# Maternal Nutritional Status Predicts Adverse Birth Outcomes among HIV-Infected Rural Ugandan Women Receiving Combination Antiretroviral Therapy

**DOI:** 10.1371/journal.pone.0041934

**Published:** 2012-08-07

**Authors:** Sera Young, Katherine Murray, Julia Mwesigwa, Paul Natureeba, Beth Osterbauer, Jane Achan, Emmanuel Arinaitwe, Tamara Clark, Veronica Ades, Albert Plenty, Edwin Charlebois, Theodore Ruel, Moses Kamya, Diane Havlir, Deborah Cohan

**Affiliations:** 1 Division of Nutritional Sciences, Cornell University, Ithaca, New York, United States of America; 2 Center for AIDS Prevention Studies, University of California San Francisco, San Francisco, California, United States of America; 3 Makerere University-University of California San Francisco Research Collaboration, Kampala, Uganda; 4 Department of Medicine, University of California San Francisco, San Francisco, California, United States of America; 5 Department of Paediatrics and Child Health, Makerere University College of Health Sciences, Kampala, Uganda; 6 Department of Obstetrics, Gynecology and Reproductive Sciences, University of California San Francisco, San Francisco, California, United States of America; 7 Department of Pediatrics, University of California San Francisco, San Francisco, California, United States of America; 8 Department of Medicine, Makerere University Medical School, Kampala, Uganda; UCL Institute of Child Health, University College London, United Kingdom

## Abstract

**Objective:**

Maternal nutritional status is an important predictor of birth outcomes, yet little is known about the nutritional status of HIV-infected pregnant women treated with combination antiretroviral therapy (cART). We therefore examined the relationship between maternal BMI at study enrollment, gestational weight gain (GWG), and hemoglobin concentration (Hb) among 166 women initiating cART in rural Uganda.

**Design:**

Prospective cohort.

**Methods:**

HIV-infected, ART-naïve pregnant women were enrolled between 12 and 28 weeks gestation and treated with a protease inhibitor or non-nucleoside reverse transcriptase inhibitor-based combination regimen. Nutritional status was assessed monthly. Neonatal anthropometry was examined at birth. Outcomes were evaluated using multivariate analysis.

**Results:**

Mean GWG was 0.17 kg/week, 14.6% of women experienced weight loss during pregnancy, and 44.9% were anemic. Adverse fetal outcomes included low birth weight (LBW) (19.6%), preterm delivery (17.7%), fetal death (3.9%), stunting (21.1%), small-for-gestational age (15.1%), and head-sparing growth restriction (26%). No infants were HIV-infected. Gaining <0.1 kg/week was associated with LBW, preterm delivery, and a composite adverse obstetric/fetal outcome. Maternal weight at 7 months gestation predicted LBW. For each g/dL higher mean Hb, the odds of small-for-gestational age decreased by 52%.

**Conclusions:**

In our cohort of HIV-infected women initiating cART during pregnancy, grossly inadequate GWG was common. Infants whose mothers gained <0.1 kg/week were at increased risk for LBW, preterm delivery, and composite adverse birth outcomes. cART by itself may not be sufficient for decreasing the burden of adverse birth outcomes among HIV-infected women.

**Trial Registration:**

Clinicaltrials.gov NCT00993031

## Introduction

As the availability of combination antiretroviral therapy (cART) for HIV-infected pregnant women broadens and perinatal HIV transmission is reduced, there are increasing numbers of HIV-exposed, uninfected children worldwide [1]. Studies to date suggest that these children have worse outcomes compared to their HIV-unexposed counterparts [2]–[6]. Preterm delivery (PTD), low birth weight (LBW), stunting, and other markers of fetal growth restriction are important predictors of neonatal mortality, post-neonatal infant mortality, and infant and child morbidity [7]–[16]. Among the many factors that predict poor gestational outcomes, maternal nutritional status before and during pregnancy has emerged as a major modifiable determinant [15], [17]–[21]. Specifically, pre-pregnancy body mass index (BMI) and gestational weight gain (GWG) have repeatedly been associated with LBW [22]–[28]. Little is known, however, about the relationship between maternal nutritional status and birth outcomes among HIV-infected women on cART, particularly in the resource-constrained settings of rural sub-Saharan Africa.

Given that nutritional status is a strong modifiable predictor of birth outcomes, we sought to characterize the baseline nutritional status of pregnant women initiating cART in rural Uganda and examine the associations between their nutritional status and adverse birth outcomes.

## Methods

### Study Design and Population

We analyzed nutritional data from an ongoing prospective clinical trial in Tororo, Uganda, evaluating malaria outcomes among women randomized to receive an HIV protease inhibitor or non-nucleoside reverse transcriptase inhibitor based cART regimen (NCT00993031, http://clinicaltrials.gov). The protocol for this trial and supporting CONSORT checklist are available as supporting information; see Checklist S1 and Protocol S1. Women with HIV-1 infection and a documented pregnancy between 12 and 28 weeks of gestation were enrolled (Figure S1). Women were excluded if they had ever used cART, had received single-dose nevirapine within 2 years, had prior dose-limited toxicity to trimethoprim-sulfamethoxazole (TS) within 2 weeks, received any contraindicated medications, had any WHO stage 4 diseases, had cardiac abnormalities, or if they had abnormal laboratory values at screening, including hemoglobin (Hb) <7.5 g/dL. All women gave written informed consent. The study protocol was approved by the Faculty of Medicine’s Research and Ethics Committee at Makerere University, the Uganda National Council of Science and Technology, and the Committee on Human Research at the University of California San Francisco.

### Study Procedures

Trained study staff collected baseline demographic data and general medical, HIV, and obstetric history. Close birth spacing was defined as <2 years between births (either live born or stillbirth) based on self-reported obstetric history. Socioeconomic status (SES) was assessed by performing principle component analysis of a series of questions about possession of a radio, telephone, television, motorcycle, or bicycle. We used the first two components of the principle component analysis which accounted for over 55% of the information contained in the 6 asset holding variables.

Maternal height was measured to the nearest 0.1 cm using a Seca 206 wall-mounted measuring tape and maternal weight was measured to the nearest 500 g using a Seca 876 mechanical scale. Gestational age was estimated based on last menstrual period (LMP) and fetal ultrasound at the screening visit. All women received a fetal ultrasound. Final pregnancy dating was based on ultrasound if the discrepancy between LMP and ultrasound was greater than 1 week in the 1^st^ trimester, 2 weeks in the 2^nd^ trimester or 3 weeks in the 3^rd^ trimester [29]. Ultrasound was used to date 50.5% of second trimester pregnancies (n = 105) and 43.4% of third trimester pregnancies (n = 53), for a total of 48.1% of the 158 pregnancies in these analyses. HIV status was documented with a positive rapid HIV antibody test (Determine, Inverness Medical Japan Co., Japan) plus a confirmatory test (Stat-Pak, Chembio Diagnostic Systems, Inc., NY, USA). All women received multivitamins containing iron and folic acid, iron supplements, prophylactic mebendazole, an insecticide-treated bed net, and were started on either zidovudine/lamivudine/efavirenz or zidovudine/lamivudine/lopinavir/ritonavir. Women were also started on daily TS if they were not already receiving TS prophylaxis prior to study enrollment.

Women returned to the study clinic every four weeks until delivery for scheduled study visits as well as when they experienced adverse events or any health conditions requiring evaluation. At scheduled monthly visits, maternal weight was measured as described above. Laboratory evaluations were regularly conducted throughout pregnancy including Hb, HIV RNA PCR, and CD4/CD8 lymphocyte subsets. Clinical progression of HIV disease was categorized according to 2007 WHO criteria [30]. Adverse events were classified according to the standardized Toxicity Table for Grading Severity of Adult and Pediatric Adverse Events [31]. Clinical malaria was defined using standardized WHO criteria as the presence of fever within the past 24 hours and a positive thick blood smear. At each scheduled visit, women were given a 5-week supply of multivitamins, antiretroviral therapy, and TS. Data were collected on socioeconomic status (SES) during a scheduled visit after enrollment.

If delivery took place in the hospital, trained study staff assessed infant anthropometry immediately after birth. Infant weight was measured to the nearest 10 g using a calibrated digital Seca 354 scale. Birth length was obtained using a locally made infant length board. Head circumference was measured to the nearest 0.1 cm using non-stretchable tape (Seca 212). If delivery occurred outside the study-affiliated hospital and infants were brought to the clinic, the anthropometric assessments were performed by study staff as described above. Only those infants who were measured within 12 hours of birth were included in this analysis. Infant HIV status at birth was determined by HIV-1 DNA PCR (Cobas Amplicor, Roche Diagnostics).

### Nutritional Measures

The following markers of maternal nutritional status were assessed: body mass index (BMI) at enrollment, baseline Hb, mean Hb during pregnancy, maternal weight at 24–28 weeks gestation [18], and maternal weight change during pregnancy. Because there are no BMI standards for pregnant women, only non-pregnant adults [32], we categorized BMI at enrollment based on tertiles (<20.43, 20.43–22.59, and >22.59). Hb concentrations were categorized into 3 groups: severe anemia (≤8.5 g/dL), mild-to-moderate anemia (8.5–10.9 g/dL), and no anemia (≥11 g/dL). Because weekly maternal weight gain should be linear in the second and third trimesters [33], weekly maternal weight change was calculated by dividing the weight change between enrollment and last weight before delivery by the number of weeks elapsed between the two. For the categorical GWG variable, weight loss was defined as a rate of weight change <0 kg/wk. Those who had a positive average weekly weight gain were categorized as <25^th^ percentile of gainers (<0.1 kg per week) or ≥25^th^ percentile (≥0.1 kg per week). Maternal weight at 7 months gestation was defined as the woman’s weight in kg between 24 and 28 weeks of gestation.

### Outcomes

Infant outcomes included LBW, small for gestational age (SGA), stunting, wasting, underweight, preterm delivery, head-sparing growth restriction, and fetal death. LBW was defined as <2500 grams. SGA newborns were those with birth weight <10th percentile for their gestational age [34], [35]. Stunting, wasting, and underweight were defined as standardized, sex-specific Z-scores of ≤−2 using length-for-age, weight-for-length and weight-for-age respectively. Z-scores were created using the WHO 2007 SAS Macro package [36]. Head-sparing growth restriction was defined as the presence of LBW, SGA, stunting, wasting, or underweight with a head circumference standardized Z-score of −1 or better [34]. Preterm delivery was defined as birth at less than 37 weeks gestation. Fetal death was defined as either miscarriage (12–20 weeks of gestation) or stillbirth (intrauterine fetal demise >20 weeks of gestation). Lastly, we created a composite dichotomous adverse birth outcome variable including LBW, SGA, stunting, wasting, underweight, preterm delivery, and fetal death.

### Statistical Analyses

We restricted the analysis to singleton pregnancies because of the well-established relationship between multiple births and LBW and preterm delivery [37]. In addition, analyses of weight change during pregnancy were restricted to those women whose weights had been measured at least twice before delivery, with their last weight evaluated within two weeks prior to date of delivery.

We calculated descriptive statistics and created scatterplots to assess distribution of the data and to inform regression modeling. Chi-square and Fisher’s exact tests were performed, as appropriate, to test for associations between categorical variables. Wilcoxon signed-rank tests were performed to compare the means of continuous characteristics by categorical variables. For all logistic and linear regression models, univariate analyses were first performed to assess relationships. Subsequently, multivariate logistic regression models were fit with dichotomous outcomes and clinically important predictors: birth spacing (<2 years) and CD4+ count at screening. Multivariate linear regression models were also built for continuous outcome variables. Birth spacing and CD4+ count were included in all models because of their clinical significance, as were any other predictors meeting the p≤0.2 threshold in univariate analysis. Multivariate model inputs were evaluated for significant multi-collinearity and where highly correlated, the variable with strongest association was retained. Model fit was assessed between model versions using differences in the −2 log likelihood and the difference in degrees of freedom between models relative to the Chi-square distribution. All analyses were conducted using SAS version 9.2 (Cary, North Carolina).

## Results

### Study Population

There were 232 women enrolled in the randomized clinical trial between December 15, 2009 and May 24, 2011. Of these, 166 delivered by May 24, 2011. Of these, 158 (95.2%) were singleton birth and were included in the analysis. Of the 158 births, 13.9% occurred outside the hospital. Birthweight was obtained within 12 hours of birth for 153 (96.8%) of the 158 women with singleton births.

The median age of participants at baseline was 29 years, and they were enrolled at a mean gestational age of 21.6 weeks (Table 1). Nearly 94% of participants were multigravidae. Over 90% of participants were diagnosed with WHO Stage 1 HIV disease and half of the women had baseline CD4 counts >350. The mean baseline BMI of participants was 21.8. Nearly 45% of participants were diagnosed with anemia (Hb <11 g/dL) prior to the initiation of cART. A diagnosis of clinical malaria during pregnancy was made for 7.6% of participants.

**Table 1:** Baseline Characteristics of Women at Enrollment (n = 158)^1,2^.

Age, Median (IQR)	29 (26, 34)
Gestational age, Mean (SE)	21.6 (0.3)
Weight in kg, Median (IQR) (n = 157)	57 (52, 62)
Height in cm, Median (IQR) (n = 156)	162 (158, 166)
BMI, Mean (SE) (n = 155)	21.8 (0.2)
BMI (n = 155)	
First tertile (<20.43)	50 (32.3%)
Second tertile (20.43–22.59)	55 (35.5%)
Third tertile (>22.59)	50 (32.3%)
Gravidity, Mean (SE)	4.8 (0.2)
Parity, Mean (SE) (n = 157)	3.4 (0.2)
Primigravid [n (%)]	10 (6.3%)
Number of living children, Mean (SE)	2.9 (0.14)
History of preterm delivery	8 (5.1%)
Years since last term birth, Mean (SE)(n = 145)	4.1 (0.2)
Education	
Less than primary	130 (82.3%)
Primary or more	28 (17.7%)
Bednet at home(n = 157)	85 (54.1%)
HIV diagnosis during index pregnancy	52 (32.9%)
WHO Stage 1	145 (91.8%)
WHO Stage 2	12 (7.6%)
WHO Stage 3	1 (0.6%)
CD4 cell count (n = 157)	
<200	22 (14%)
200–350	57 (36.3%)
>350	78 (49.7%)
Log(10) Viral Load, Mean (SE) (n = 154)	4.08 (0.1)
Hb, Mean (SE)	11.05 (0.1)
Anemia, Hb <11	71 (44.9%)

1 Data are from entire sample (n = 158) unless otherwise noted.

2 All data are represented as n (%) unless otherwise noted.

IQR: Interquartile range.

SE: standard error.

BMI: Body mass index (kg/m^2^).

### Maternal Weight Gain During Pregnancy

The median weekly GWG during study participation was 0.17 kg (interquartile range [IQR]: 0.06, 0.3). Data on median weekly GWG were unavailable for two participants who were not weighed within two weeks of delivery. Twenty-three women (14.6%) lost weight during pregnancy. The median total GWG during study participation was 3 kg (IQR: 1, 5). In addition, the median maternal weight at 7 months gestation was 58 kg (IQR: 52, 63) and the median BMI at 7 months gestation was 22 (IQR: 20.5, 24.1). Adjusting for birth spacing, baseline CD4 count, baseline weight, mean Hb during pregnancy, and Grade 3 or 4 adverse events, there were no significant predictors of gestational weight gain. (Univariate relationships and the full linear regression model can be found in Table S1).

### Obstetric and Neonatal Outcomes

There were no infants infected with HIV at birth. The prevalence of LBW was 19.6%, preterm delivery 17.7%, and fetal death 3.9% (Table 2). Stunting was the most common anthropometric marker of growth restriction seen among live-born newborns (21.1%), followed by SGA (15.1%) and underweight (15.1%). Twenty six percent of live-born infants were diagnosed with head-sparing growth restriction. Seventy three percent of participants experienced a full-term delivery of a live-born infant with normal birth weight.

**Table 2:** Obstetric and Fetal Outcomes (n = 158)^1^.

Male sex (n = 155)	83(53.6%)
Overall low birth weight (n = 153)	30 (19.6%)
Overall preterm delivery	28 (17.7%)
Full term, normal birth weight (n = 152)	111 (73.0%)
Full term, low birth weight (n = 152)	13 (8.6%)
Preterm, normal birth weight (n = 152)	11 (6.6%)
Preterm, low birth weight (n = 152)	12 (7.9%)
Miscarriage (12–20 weeks) (n = 152)	1 (0.6%)
Stillbirth (>20 weeks) (n = 152)	5 (3.3%)
Composite adverse obstetric/fetal outcome	64 (40.5%)
Live born infants only (n = 152)^1^	
Stunting (LAZ^2^≤ −2) at birth (n = 142)	30 (21.1%)
Small for gestational age (<10th percentile)	23 (15.1%)
Underweight (WAZ^3^≤ −2) at birth	23 (15.1%)
Wasting (WLZ^4^≤ −2) at birth (n = 128)	11 (8.6%)
Small head circumference (HCZ^5^≤ −2) at birth (n = 150)	10 (6.7%)
Head-sparing growth restriction (n = 150)	39 (26.0%)

1 All data are represented as n (%).

2 LAZ: length-for-age Z-score.

3 WAZ: weight-for-age Z-score.

4 WLZ: weight-for-length Z-score.

5 HCZ: head circumference Z-score.

### Risk Factors for Adverse Outcomes

#### Low birth weight

In multivariate analysis adjusting for birth spacing (<2 years) and baseline CD4 count, each kg increase in maternal weight at enrollment was associated with a 30% decreased odds of LBW (aOR 0.70, 95% CI 0.52–0.95, p = 0.022, cf. Table S2). Each cm increase in maternal height was associated with an 8% lower odds of LBW (aOR 0.92, 95% CI 0.85–1.00, p = 0.046). Moreover, women who gained <0.1 kg per week had greater than 6-fold increase in odds of LBW compared to women who gained ≥0.1 kg per week (aOR 6.18, 95% CI 1.80–21.1, p = 0.004). Lastly, each kg decrease in total maternal weight at 7 months gestation was associated with a 38% increased odds of LBW (aOR 1.38, 95% CI 1.03–1.90, p = 0.034).

#### Small-for-gestational age

Adjusting for birth spacing (<2 years), baseline CD4 count, and any GWG, each g/dL increase in mean maternal Hb between enrollment and final measurement was associated with a 52% decreased odds of SGA (aOR 0.48, 95% CI 0.29–0.80, p = 0.004, cf. Table S3).

#### Stunting

After adjusting for birth spacing (<2 years) and baseline CD4 count, risk factors for neonatal stunting included male infant sex (aOR 6.02, 95% CI 1.64–22.06, p = 0.007), gestational age at delivery (aOR 0.51, 95%CI 0.37–0.71, p<0.001) and clinical malaria diagnosed during pregnancy (aOR 0.18, 95% CI 0.03–0.97, p = 0.047, cf. Table S4).

#### Head-sparing fetal growth restriction

There were no statistically significant predictors of head-sparing growth restriction in multivariate analysis adjusting for birth spacing, baseline CD4 count and mean maternal Hb during the study, cf. Table S5.

#### Preterm delivery

Adjusting for birth spacing, baseline CD4 count and history of preterm delivery, GWG <0.1 kg per week was associated with a 4-fold increased odds of preterm delivery (aOR 3.46, 95% CI 1.18–10.15, p = 0.024, cf. Table S6).

#### Composite adverse birth outcome

Lastly, predictors of the composite dichotomous adverse birth outcome included GWG <0.1 kg per week, higher SES status, and birth from June to October (the rainy season), adjusting for CD4 count and birth spacing (Table 3). In particular, gaining <0.1 kg per week was associated with a nearly 3 fold increased odds of an adverse birth outcome (aOR 2.85, 95% CI 1.32–6.15, p<0.01).

**Table 3:** Predictors of Composite Adverse Obstetric/Fetal Outcome^1^, Univariate and Multivariate Analysis.

	Univariate Analysis				Final Multivariate Model		
	N	Outcome (%)	OR	P	aOR^2^	95% CI	P
Birth spacing <2 years	158	77 (44.4%)	1.19	0.80	0.89	0.20–3.92	0.88
Baseline CD4 count (continuous)	157	64 (40.8%)	1	0.95	1	0.99–1.00	0.84
Maternal weight at 7 months gestation^3^	155	62 (40%)	0.97	0.15	0.95	0.91–1.00	0.07
Weekly weight gain							
<0.1 kg/week	54	28 (52%)	2.12	0.03	2.85	1.32–6.15	<0.01
0.1 kg/week or greater	101	34 (33.7%)			–	1	
Higher SES^4^	148	30 (47%)	1.67	0.13	2.61	1.20–5.67	0.02
Season of birth							
June to October	56	16 (28.6%)	0.45	0.03	0.33	0.15–0.74	<0.01
November to May	102	48 (47.1%)	1		–	1	

1 Composite adverse obstetric/fetal outcome includes any of the following: low birth weight, SGA, stunting, wasting, underweight, preterm delivery, and fetal death.

2 Adjusted odds ratio using multivariate logistic regression, adjusting for all variables listed in table.

3 Per kg increment in maternal weight at 7 months gestation.

4 Indicator variable for household being in the upper quartile of either the first or second component of the SES principal component analysis based on possession of a radio, telephone, television, motorcycle, bicycle or none of the above.

OR: Odds Ratio.

P: p-value.

aOR: Adjusted Odds Ratio.

95% CI: Confidence Interval.

## Discussion

Combination antiretroviral therapy is being delivered to increasing numbers of rural HIV-infected pregnant women in developing countries [38]–[40]. Goals of treatment include protecting the health of these women and promoting the birth of HIV-uninfected, healthy infants. However, cART alone may not be sufficient to achieve these goals, particularly when these women also often face other challenges besides HIV, including food insecurity and malnutrition [41].

In our cohort of HIV-infected pregnant women in rural Uganda initiating cART, TS and prenatal care, we found evidence of significant nutritional deficiencies. These women had low BMIs upon study entry and well into their pregnancy, despite having relatively preserved CD4 cell counts. Their mean weight gain of 0.17 kg/week was far below the 0.5 kg/week recommended by the Institute of Medicine for underweight women in industrialized countries in the second and third trimester [42]. Although all infants were HIV-uninfected at delivery, adverse birth outcomes were highly prevalent and likely attributable at least in part to poor maternal nutritional status.

Interestingly, HIV severity, measured as baseline CD4 count, viral load, or WHO stage, was not predictive of adverse birth outcomes. This could have been due to our sample size and the relatively small proportion of women with severe immune suppression. Indeed, 50% of our cohort had baseline CD4 counts above 350 and the majority of the women (91.8%) were WHO stage 1.

Maternal nutritional predictors of preterm delivery and growth restriction have primarily been evaluated among HIV-infected women not receiving cART. The Pregnancy and HIV Study Group of 177 ARV-naïve women in Rwanda found that each kg increment in final weight before delivery was associated with a 6% decreased odds of LBW [43]. Villamor et al. found that low maternal weight at first prenatal visit was associated with lower mean birth weight and SGA but not preterm delivery among 1002 ARV-naïve women in Tanzania [44]. Similar to our study, the prevalence of gestational weight loss was 10%, and weight loss was associated with LBW, preterm delivery and fetal death. In Zambia, Banda et al. found infant birth weight increased by 28.3 g for every unit increase in BMI at 36 weeks of gestation; they did not assess the risk of preterm delivery or other markers of growth restriction [41]. Finally, Mehta et al. analyzed outcomes of 2294 ARV-naïve pregnant women enrolled in HIVNET 024 [27]. They found enrollment maternal BMI in the lowest tertile to be associated with LBW and preterm delivery. As observed in our study, weight gain <0.1 kg per week was associated with increased risk of LBW.

Very few studies have evaluated nutritional predictors of pregnancy outcomes among HIV-infected women on cART. Ekouevi and colleagues studied 151 pregnant women receiving cART as part of the ANRS Ditrame Plus and the MTCT-Plus Projects in Cote d’Ivoire [45]. Similar to the Pregnancy and HIV Study Group in Rwanda, these researchers found maternal BMI at delivery to be predictive of LBW. In particular, the odds of LBW was 2.43 fold higher among women with a delivery BMI <25. Conversely, Powis et al recently reported that change in BMI one month following the initiation of cART in pregnancy was not significantly associated with preterm delivery among 530 HIV-infected pregnant women in Botswana [46].

It is reasonable to postulate that pregnant women treated with cART would have improved nutritional status compared to those without access to cART. Women receiving effective cART should experience less HIV morbidity, including diarrhea and wasting, which should outweigh the toxicity of the antiretroviral agents. However, our study and the two others examining nutritional markers among pregnant women receiving cART can neither support nor refute this assumption because all women received cART, and it would be unethical to randomize to non-cART treatment regimens. Furthermore, even if cART does improve nutritional status, we demonstrate in this cohort that there remain significant nutritional deficiencies and that these are associated with poor birth outcomes.

Mechanisms to explain these poor outcomes are likely numerous and not yet fully understood. For example, head-sparing, or asymmetric, growth restriction is thought to be due to preferential blood flow to the brain in the setting of placental insufficiency [47]. Indeed, the inverse association between head-sparing growth restriction and weekly GWG suggests a nutritional basis for this placental insufficiency.

Much work is needed to determine factors that contribute to low GWG and weight loss among HIV-infected pregnant women, including the impact of initiating HAART during pregnancy versus use of HAART prior to conception. It is also necessary to develop strategies to identify those women at greatest risk for poor birth outcomes. Pre-pregnancy BMI has consistently been associated with adverse birth outcomes. However, because most women do not know their pre-pregnancy weight and do not have regular access to preconception care, this indicator is not clinically useful. In order to identify another relevant maternal anthropometric predictor of adverse fetal outcomes, Kelly and colleagues conducted a meta-analysis of 25 studies including over 111,000 births worldwide [18]. They found that low maternal weight attained at 7 months gestation was a significant risk factor for fetal growth restriction, particularly among women with below average pre-pregnancy weight. Indeed in our study, those women with low weight at 7 months gestation were at particularly high risk of LBW. Because increased GWG in the 3^rd^ trimester was associated with a diminished odds of LBW, preterm delivery, and overall adverse birth outcome, these women with low weight at 7 months may benefit from a nutritional intervention.

Preterm delivery, LBW, neonatal stunting, SGA, and wasting are strong predictors of infants’ future health trajectories [48]. With the increased availability of cART during pregnancy and breastfeeding, there is an expanding generation of HIV-exposed, uninfected children. Nutritional interventions that increase maternal weight gain during pregnancy have the potential to decrease the burden of a range of adverse birth outcomes among women infected with HIV. As such, the improvement of maternal nutritional status may be a golden opportunity to not only protect the health of the mother, but to improve birth outcomes and create a thriving generation of HIV-exposed, uninfected offspring.

There are several limitations to our study. Small sample size may have impaired our ability to find statistically significant predictors of head-sparing fetal growth restriction and other poor outcomes. Our findings may not be generalizable to other cohorts of HIV-infected pregnant women receiving cART (e.g. [38], [39], [40]) because our participants were older, nearly all were multigravidae and mean BMI was lower [38]–[40]. Further, our results may not be generalizable to those women on cART prior to conceiving. We excluded multiple births because of its known effect on adverse birth outcomes, which may further limit the generalizability of our findings. Our finding that higher SES was associated with an increased odds of an adverse birth outcome may be spurious because the SES measure was generated using principal component analysis of specific asset holding questions and not a validated poverty scale. Such a measurement could have been vulnerable to unmeasured confounding. Finally, we analyzed data from an on-going randomized trial and differences by study treatment arm cannot be addressed until study completion and data unblinding.

In conclusion, initiating cART during pregnancy among HIV–infected women in rural Uganda successfully prevented HIV transmission to their infants but did not prevent poor nutritional status during pregnancy that independently predicted poor birth outcomes. More attention is needed to characterize the scope and causes of nutritional deficiencies among this population and to design interventions that improve both the health of HIV-infected mothers and optimize the health and development of their offspring.

## Supporting Information

Figure S1 **CONSORT 2010 flow diagram for NCT00993031.**
(TIFF)

Table S1 **Univariate and multivariate linear regression models of weekly gestational weight gain.**
(DOC)

Table S2 **Univariate and multivariate logistic regression models of low birthweight.**
(DOC)

Table S3 **Univariate and multivariate logistic regression models of small for gestational age.**
(DOC)

Table S4 **Univariate and multivariate logistic regression models of stunting.**
(DOC)

Table S5 **Univariate and multivariate logistic regression models of head-sparing growth restriction.**
(DOC)

Table S6 **Univariate and multivariate logistic regression models of preterm delivery.**
(DOC)

Protocol S1 **Trial protocol.**
(DOC)

Checklist S1 **CONSORT checklist.**
(PDF)
