# Supplementary figures and images for: Maternal Nutritional Status Predicts Adverse Birth Outcomes among HIV-Infected Rural Ugandan Women Receiving Combination Antiretroviral Therapy
Source: PLoS One. 2012 Aug 7;7(8):e41934. doi: 10.1371/journal.pone.0041934 (PMC3413694; doi:10.1371/journal.pone.0041934)

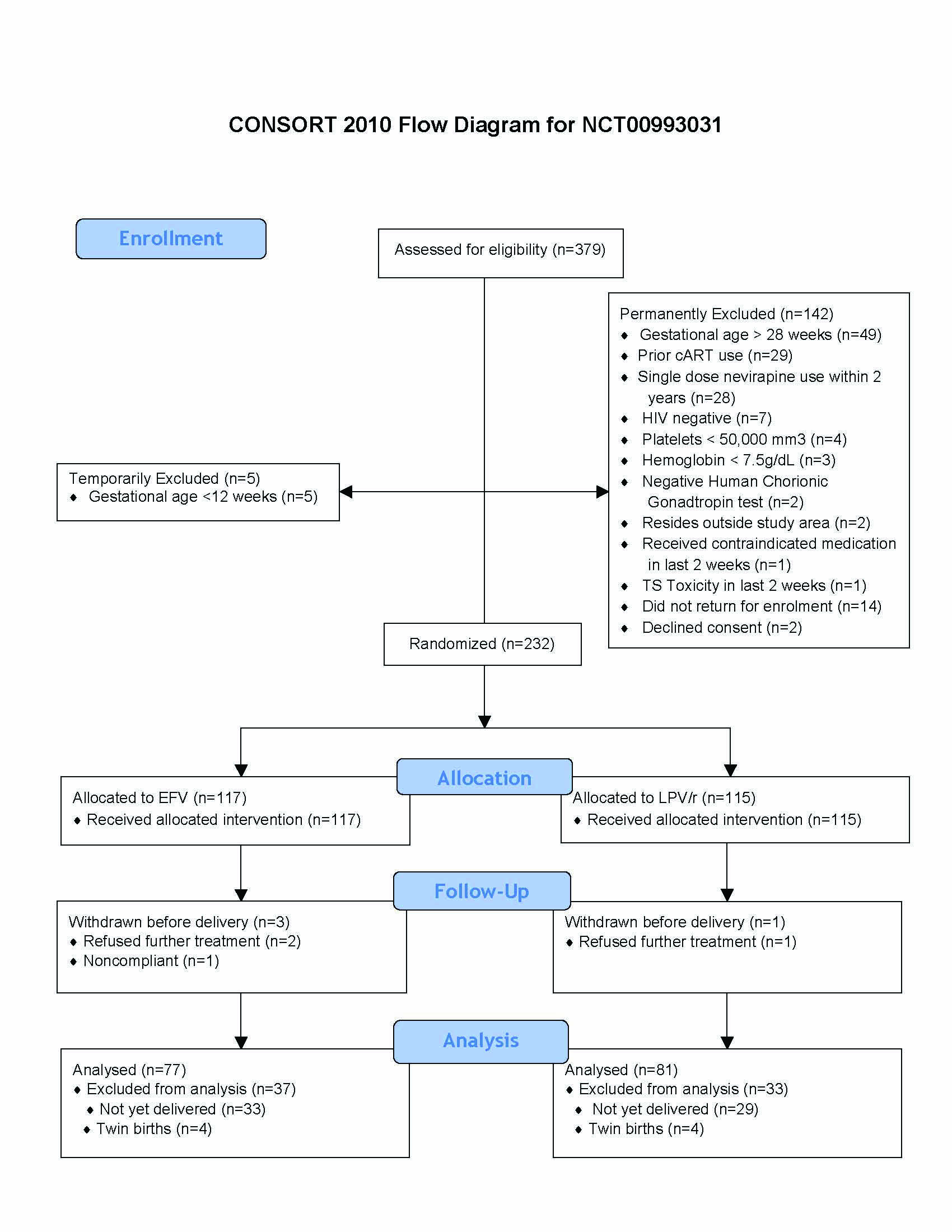

Supplement: Figure S1 — CONSORT 2010 flow diagram for NCT00993031. (TIFF) [file pone.0041934.s001.tiff]
