# Supplementary material for: Maternal Nutritional Status Predicts Adverse Birth Outcomes among HIV-Infected Rural Ugandan Women Receiving Combination Antiretroviral Therapy
Source: PLoS One. 2012 Aug 7;7(8):e41934. doi: 10.1371/journal.pone.0041934 (PMC3413694; doi:10.1371/journal.pone.0041934)
Supplement: Table S1 — Univariate and multivariate linear regression models of weekly gestational weight gain. (DOC) [file pone.0041934.s002.doc]

Table S1. Univariate and multivariate linear regression models of weekly gestational weight gain.

| Gestational Weight Gained per Week, N=146 | Univariate Model | | Final Multivariable Model | |
| --- | --- | --- | --- | --- |
|  | Beta | p-value | Beta | p-value |
| Log(10)viral Load at enrollment | 0.0107 | 0.65 |  |  |
| CD4 at screening | -0.00066 | 0.49 | -0.00005 | 0.62 |
| CD4 at screening (categorical) |  |  |  |  |
| 200-350 vs. <200 | 0.066 | 0.29 |  |  |
| >350 vs. <200 | 0.031 | 0.6 |  |  |
| WHO Stage at enrollment |  |  |  |  |
| Stage 1 vs. Stage 3 | -1.58 | 0.53 |  |  |
| Stage 2 vs. Stage 3 | -1.12 | 0.66 |  |  |
| Hemoglobin at baseline | -0.021 | 0.24 | -0.0151 | 0.43 |
| Hemoglobin at baseline |  |  |  |  |
| <8.5 vs. >11 | -0.027 | 0.81 |  |  |
| 8.5-10.9 vs. >11 | 0.066 | 0.11 |  |  |
| Mean hemoglobin throughout pregnancy | -0.0095 | 0.63 |  |  |
| Gravidity | -0.006 | 0.53 |  |  |
| Parity | -0.011 | 0.3 |  |  |
| Birth spacing (<2 y) | -0.0088 | 0.27 | -0.0058 | 0.48 |
| Maternal age at enrollment | -0.0015 | 0.69 |  |  |
| Maternal height at enrollment | -0.0036 | 0.18 |  |  |
| Maternal weight at enrollment | -0.0039 | 0.15 | -0.0036 | 0.24 |
| Maternal BMI at enrollment | -0.0037 | 0.61 |  |  |
| Maternal BMI at enrollment |  |  |  |  |
| 1st tertile vs. 3^rd^ tertile | 0.062 | 0.19 |  |  |
| 2nd tertile vs. 3^rd^ tertile | 0.065 | 0.17 |  |  |
| Less than primary school education | -0.048 | 0.36 |  |  |
| Duration of days of TS prior to enrollment | 0.00046 | 0.0002 |  |  |
| Duration of days of TS prior to enrollment |  |  |  |  |
| 1-30 vs. none | -0.02 | 0.68 |  |  |
| 31+ vs. none | -0.056 | 0.28 |  |  |
| Maternal weight at 5 months gestation | -0.0018 | 0.51 |  |  |
| Maternal weight at 7 months gestation | -0.0033 | 0.9 |  |  |
| Mean BMI at 5 months | 0.003 | 0.71 |  |  |
| Mean BMI at 7 months | 0.0076 | <0.001 |  |  |
| Season of birth |  |  |  |  |
| June to October | 0.033 | 0.43 |  |  |
| November to May | 1 | - |  |  |
| Incident clinical malaria |  |  |  |  |
| None | -0.037 | 0.62 |  |  |
| One or more episodes | 1 | - |  |  |
| 3 or 4 AE's | 0.299 | 0.23 | 0.2592 | 0.31 |
| Higher SES | 0.0037 | 0.93 |  |  |
