# Supplementary material for: Maternal Nutritional Status Predicts Adverse Birth Outcomes among HIV-Infected Rural Ugandan Women Receiving Combination Antiretroviral Therapy
Source: PLoS One. 2012 Aug 7;7(8):e41934. doi: 10.1371/journal.pone.0041934 (PMC3413694; doi:10.1371/journal.pone.0041934)
Supplement: Table S2 — Univariate and multivariate logistic regression models of low birthweight. (DOC) [file pone.0041934.s003.doc]

Table S2. Univariate and multivariate logistic regression models of low birthweight.

| Low birth weight (<2500 g), N=142 | Univariate Model | | Final Multivariable Model | | |
| --- | --- | --- | --- | --- | --- |
|  | OR | p-value | aOR | 95% CI | p-value |
| Sex of infant (male vs. female) | 1.13 | 0.78 |  |  |  |
| Log(10)viral Load at enrollment | 1 | 0.80 |  |  |  |
| CD4 at screening | 0.99 | 0.50 | 1.00 | 0.99-1.00 | 0.98 |
| CD4 at screening (categorical) |  |  |  |  |  |
| 200-350 vs. <200 | 0.89 | 0.94 |  |  |  |
| >350 vs. <200 | 0.85 | 0.82 |  |  |  |
| CD4 nadir | 0.99 | 0.61 |  |  |  |
| Hemoglobin at baseline | 0.95 | 0.78 |  |  |  |
| Hemoglobin at baseline |  |  |  |  |  |
| <8.5 vs. >11 | 1.21 | 0.86 |  |  |  |
| 8.5-10.999 vs. >11 | 0.99 | 0.88 |  |  |  |
| Mean hemoglobin throughout pregnancy | 0.75 | 0.18 |  |  |  |
| WHO Stage at enrollment |  |  |  |  |  |
| Stage 1 vs. Stage 3 | >999.99 | 0.99 |  |  |  |
| Stage 2 vs. Stage 3 | >999.99 | 0.99 |  |  |  |
| Primigravida vs. multigravida | 2.61 | 0.20 |  |  |  |
| Birth spacing | 0.96 | 0.68 |  |  |  |
| Birth spacing: <2 years vs. nullipara or > 2 years | 0.59 | 0.62 | 0.50 | 0.05-4.80 | 0.55 |
| Maternal age at enrollment | 0.99 | 0.87 |  |  |  |
| Maternal height at enrollment | 0.89 | 0.002 | 0.92 | 0.85-0.99 | 0.05 |
| Maternal weight at enrollment | 0.93 | 0.02 | 0.70 | 0.52-0.95 | 0.02 |
| Maternal BMI at enrollment | 1.01 | 0.91 |  |  |  |
| Maternal BMI at enrollment |  |  |  |  |  |
| 1st tertile vs. 3rd | 1.21 | 0.71 |  |  |  |
| 2nd tertile vs. 3rd | 1.01 | 0.87 |  |  |  |
| Less than primary school education | 1.63 | 0.46 |  |  |  |
| Weekly weight gain (1kg increments) | 0.34 | 0.29 |  |  |  |
| Weekly weight gain |  |  |  |  |  |
| < 25th percentile of gainers vs. losers | 0.83 | 0.55 |  |  |  |
| ≥ 25th percentile of gainers vs. losers | 0.38 | 0.05 |  |  |  |
| Weekly weight gain < 0.1 kg | 2.37 | 0.05 | 6.18 | 1.80-21.2 | 0.004 |
| Weekly weight gain < 0.2 kg | 1.48 | 0.39 |  |  |  |
| Weight gain vs. weight loss | 0.48 | 0.21 |  |  |  |
| Total weight gained (kg) | 0.95 | 0.42 |  |  |  |
| Unsuppressed viral load at delivery | 0.4 | 0.4 |  |  |  |
| Gestational age at enrollment | 0.96 | 0.51 |  |  |  |
| Gestational age at delivery | 0.62 | <0.0001 |  |  |  |
| Duration of days of TS prior to enrollment | 0.99 | 0.74 |  |  |  |
| Duration of days of TS prior to enrollment |  |  |  |  |  |
| 1-30 vs. none | 0.99 | 0.74 |  |  |  |
| 31+ vs. none | 0.72 | 0.54 |  |  |  |
| Duration of days of TS prior to enrollment | 0.991 | 0.11 |  |  |  |
| Maternal weight at 5 months gestation | 0.93 | 0.09 |  |  |  |
| Maternal weight at 7 months gestation | 0.94 | 0.06 | 1.38 | 1.03-1.90 | 0.03 |
| Mean BMI at 5 months | 1.02 | 0.87 |  |  |  |
| Mean BMI at 7 months | 1.05 | 0.58 |  |  |  |
| Weekly weight gain, 2nd trimester only | 1.94 | 0.30 |  |  |  |
| Weekly weight gain, 3rd trimester only | 0.5 | 0.42 |  |  |  |
| Season of birth |  |  |  |  |  |
| June to October | 0.77 | 0.57 |  |  |  |
| November to May | 1 | - |  |  |  |
| Incident clinical malaria |  |  |  |  |  |
| None | 0.59 | 0.45 |  |  |  |
| One or more episodes | 1 | - |  |  |  |
| 3 or 4 AE's | >999.99 | 0.99 |  |  |  |
| Higher SES | 0.95 | 0.91 |  |  |  |
