# Supplementary material for: Maternal Nutritional Status Predicts Adverse Birth Outcomes among HIV-Infected Rural Ugandan Women Receiving Combination Antiretroviral Therapy
Source: PLoS One. 2012 Aug 7;7(8):e41934. doi: 10.1371/journal.pone.0041934 (PMC3413694; doi:10.1371/journal.pone.0041934)
Supplement: Table S3 — Univariate and multivariate logistic regression models of small for gestational age. (DOC) [file pone.0041934.s004.doc]

Table S3. Univariate and multivariate logistic regression models of small for gestational age.

| Small for gestational age, N=139 | Univariate Model | | Final Multivariable Model | | |
| --- | --- | --- | --- | --- | --- |
|  | OR | p-value | OR | 95% CI | p-value |
| Sex of infant (male vs. female) | 0.64 | 0.34 |  |  |  |
| Log(10)viral load at enrollment | 0.97 | 0.90 |  |  |  |
| CD4 at screening | 1 | 0.57 | 1 | 0.99-1.00 | 0.35 |
| CD4 at screening (categorical) |  |  |  |  |  |
| 200-350 vs. <200 | 1.43 | 0.97 |  |  |  |
| >350 vs. <200 | 1.95 | 0.35 |  |  |  |
| CD4 nadir | 1 | 0.51 |  |  |  |
| Hemoglobin at baseline | 0.83 | 0.33 |  |  |  |
| Hemoglobin at baseline |  |  |  |  |  |
| <8.5 vs. >11 | 1.52 | 0.77 |  |  |  |
| 8.5-10.999 vs. >11 | 1.17 | 0.94 |  |  |  |
| Mean hemoglobin throughout pregnancy | 0.58 | 0.02 | 0.48 | 0.29-0.80 | 0.0044 |
| WHO stage at enrollment |  |  |  |  |  |
| Stage 1 vs. Stage 3 | >999.99 | 0.99 |  |  |  |
| Stage 2 vs. Stage 3 | >999.99 | 0.99 |  |  |  |
| Primigravida vs. multigravida | 3.08 | 0.13 |  |  |  |
| Birth spacing (< 2y) | 0.96 | 0.66 | 0.96 | 0.79-1.20 | 0.71 |
| Maternal age at enrollment | 0.94 | 0.18 |  |  |  |
| Maternal height at enrollment | 0.96 | 0.20 |  |  |  |
| Maternal weight at enrollment | 0.97 | 0.34 |  |  |  |
| Maternal BMI at enrollment | 1 | 0.98 |  |  |  |
| Maternal BMI at enrollment |  |  |  |  |  |
| 1st tertile vs. 3^rd^ tertile | 1.08 | 0.97 |  |  |  |
| 2nd tertile vs. 3^rd^ tertile | 1.11 | 0.88 |  |  |  |
| Less than primary school education | 0.47 | 0.14 |  |  |  |
| Weekly weight gain (1kg increments) | 0.524 | 0.54 |  |  |  |
| Weekly weight gain |  |  |  |  |  |
| < 25th percentile of gainers vs. losers | 0.54 | 0.69 |  |  |  |
| ≥ 25th percentile of gainers vs. losers | 0.45 | 0.30 |  |  |  |
| Weekly weight gain < 0.1 kg | 1.54 | 0.35 |  |  |  |
| Weekly weight gain < 0.2 kg | 1.48 | 0.40 |  |  |  |
| Weight gain vs. weight loss | 0.47 | 0.19 | 3.12 | 0.89-10.82 | 0.07 |
| Total weight gained (kg) | 1.01 | 0.88 |  |  |  |
| Unsuppressed viral load at delivery | 0.58 | 0.61 |  |  |  |
| Gestational age at enrollment | 0.99 | 0.98 |  |  |  |
| Gestational age at delivery | 1.34 | 0.04 |  |  |  |
| Preterm delivery | 4.52 | 0.15 |  |  |  |
| Duration of days of TS prior to enrollment | 1 | 0.86 |  |  |  |
| Duration of days of TS prior to enrollment |  |  |  |  |  |
| 1-30 vs. none | 0.71 | 0.43 |  |  |  |
| 31+ vs. none | 1.08 | 0.61 |  |  |  |
| Total duration of TS days | 1 | 0.99 |  |  |  |
| Maternal weight at 5 months gestation | 0.94 | 0.25 |  |  |  |
| Maternal weight at 7 months gestation | 0.97 | 0.35 |  |  |  |
| Maternal weight at 5 months gestation | 0.95 | 0.70 |  |  |  |
| Maternal weight at 7 months gestation | 0.99 | 0.94 |  |  |  |
| Mean BMI at 5 months | 1.25 | 0.67 |  |  |  |
| Mean BMI at 7 months | 1.08 | 0.93 |  |  |  |
| Season of birth |  |  |  |  |  |
| June to October | 0.74 | 0.53 |  |  |  |
| November to May | 1 | - |  |  |  |
| Incident clinical malaria |  |  |  |  |  |
| None | 0.88 | 0.88 |  |  |  |
| One or more episodes | 1 | - |  |  |  |
| 3 or 4 AE's | >999.99 | 0.99 |  |  |  |
| Higher SES | 0.62 | 0.34 |  |  |  |
