# Supplementary material for: Maternal Nutritional Status Predicts Adverse Birth Outcomes among HIV-Infected Rural Ugandan Women Receiving Combination Antiretroviral Therapy
Source: PLoS One. 2012 Aug 7;7(8):e41934. doi: 10.1371/journal.pone.0041934 (PMC3413694; doi:10.1371/journal.pone.0041934)
Supplement: Table S4 — Univariate and multivariate logistic regression models of stunting. (DOC) [file pone.0041934.s005.doc]

Table S4. Univariate and multivariate logistic regression models of stunting.

| Stunting (HAZ <-2) at birth, N=127 | Univariate Model | | Final Multivariable Model | | |
| --- | --- | --- | --- | --- | --- |
|  | OR | p-value | OR | 95% CI | p-value |
| Sex of infant (male vs. female) | 3.29 | 0.01 | 6.02 | 1.64-22.06 | 0.007 |
| Log(10)viral Load at enrollment | 1.67 | 0.05 |  |  |  |
| CD4 at screening | 0.99 | 0.19 | 0.99 | 0.99-1.00 | 0.25 |
| CD4 at screening (categorical) |  |  |  |  |  |
| 200-350 vs. <200 | 0.35 | 0.13 |  |  |  |
| >350 vs. <200 | 0.51 | 0.72 |  |  |  |
| CD4 nadir | 0.99 | 0.29 |  |  |  |
| Hemoglobin at baseline | 0.86 | 0.41 |  |  |  |
| Hemoglobin at baseline |  |  |  |  |  |
| <8.5 vs. >11 | 1.02 | 0.94 |  |  |  |
| 8.5-10.999 vs. >11 | 1.23 | 0.76 |  |  |  |
| Mean hemoglobin throughout pregnancy | 0.68 | 0.05 |  |  |  |
| WHO stage at enrollment |  |  |  |  |  |
| Stage 1 vs. Stage 3 | <0.001 | 0.98 |  |  |  |
| Stage 2 vs. Stage 3 | <0.001 | 0.99 |  |  |  |
| Primigravida vs. multigravida | 1.96 | 0.36 |  |  |  |
| Birth spacing | 1.001 | 0.99 | 0.95 | 0.75-1.21 | 0.71 |
| Maternal age at enrollment | 1.02 | 0.66 |  |  |  |
| Maternal height at enrollment | 0.97 | 0.36 |  |  |  |
| Maternal weight at enrollment | 0.99 | 0.66 |  |  |  |
| Maternal BMI at enrollment | 1.03 | 0.68 |  |  |  |
| Maternal BMI at enrollment |  |  |  |  |  |
| 1st tertile vs. 3^rd^ tertile | 1.03 | 0.81 |  |  |  |
| 2nd tertile vs. 3^rd^ tertile | 0.85 | 0.69 |  |  |  |
| Less than primary school education | 1.5 | 0.49 |  |  |  |
| Weekly weight gain (1kg increments) | 0.16 | 0.07 |  |  |  |
| Weekly weight gain |  |  |  |  |  |
| < 25th percentile of gainers vs. losers | 0.59 | 0.9 |  |  |  |
| ≥ 25th percentile of gainers vs. losers | 0.28 | 0.02 |  |  |  |
| Weekly weight gain < 0.1 kg | 2.44 | 0.03 |  |  |  |
| Weekly weight gain < 0.2 kg | 1.49 | 0.35 |  |  |  |
| Weight gain vs. weight loss | 0.36 | 0.07 |  |  |  |
| Total weight gained (kg) | 0.87 | 0.038 |  |  |  |
| Unsuppressed viral load at delivery | 0.33 | 0.29 |  |  |  |
| Gestational age at enrollment | 1.03 | 0.56 |  |  |  |
| Gestational age at delivery | 0.59 | <0.001 | 0.51 | 0.37-0.71 | <0.001 |
| Duration of days of TS prior to enrollment | 0.99 | 0.71 |  |  |  |
| Duration of days of TS prior to enrollment |  |  |  |  |  |
| 1-30 vs. none | 1.06 | 0.78 |  |  |  |
| 31+ vs. none | 1.43 | 0.47 |  |  |  |
| Total duration of TS days | 0.99 | 0.14 |  |  |  |
| Maternal weight at 5 months gestation | 0.99 | 0.91 |  |  |  |
| Maternal weight at 7 months gestation | 0.99 | 0.68 |  |  |  |
| Mean BMI at 5 months | 1.08 | 0.5 |  |  |  |
| Mean BMI at 7 months | 1.03 | 0.75 |  |  |  |
| Weekly weight gain, 2nd trimester only | 1.82 | 0.35 |  |  |  |
| Weekly weight gain, 3rd trimester only | 0.31 | 0.16 |  |  |  |
| Season of birth |  |  |  |  |  |
| June to October | 0.44 | 0.08 |  |  |  |
| November to May | 1 | - |  |  |  |
| Incident clinical malaria |  |  |  |  |  |
| None | 0.37 | 0.14 | 0.18 | 0.03- 0.97 | 0.047 |
| One or more episodes | 1 | - |  |  |  |
| 3 or 4 AE's | >999.99 | 0.99 |  |  |  |
| Higher SES | 1.06 | 0.9 |  |  |  |
