# Supplementary material for: Maternal Nutritional Status Predicts Adverse Birth Outcomes among HIV-Infected Rural Ugandan Women Receiving Combination Antiretroviral Therapy
Source: PLoS One. 2012 Aug 7;7(8):e41934. doi: 10.1371/journal.pone.0041934 (PMC3413694; doi:10.1371/journal.pone.0041934)
Supplement: Table S5 — Univariate and multivariate logistic regression models of head-sparing growth restriction. (DOC) [file pone.0041934.s006.doc]

Table S5. Univariate and multivariate logistic regression models of head-sparing growth restriction.

| Head Sparing Growth Restriction, N=137 | Univariate Model | | Final Multivariable Model | | |
| --- | --- | --- | --- | --- | --- |
|  | OR | p-value | OR | 95% CI | p-value |
| Sex of infant (male vs. female) | 1.27 | 0.53 |  |  |  |
| Log(10)viral Load at enrollment | 1.09 | 0.69 |  |  |  |
| CD4 at screening | 1 | 0.37 | 1 | 0.99-1.00 | 0.32 |
| CD4 at screening (categorical) |  |  |  |  |  |
| 200-350 vs. <200 | 0.81 | 0.32 |  |  |  |
| >350 vs. <200 | 1.6 | 0.15 |  |  |  |
| CD4 nadir | 1 | 0.25 |  |  |  |
| Hemoglobin at baseline | 0.8 | 0.16 |  |  |  |
| Hemoglobin at baseline |  |  |  |  |  |
| <8.5 vs. >11 | 2.03 | 0.48 |  |  |  |
| 8.5-10.999 vs. >11 | 1.11 | 0.65 |  |  |  |
| Mean hemoglobin throughout pregnancy | 0.74 | 0.09 | 0.69 | 0.47-1.03 | 0.068 |
| WHO stage at enrollment |  |  |  |  |  |
| Stage 1 vs. Stage 3 | >999.99 | 0.99 |  |  |  |
| Stage 2 vs. Stage 3 | >999.99 | 0.99 |  |  |  |
| Primigravida vs. multigravida | 2.42 | 0.2 |  |  |  |
| Birth spacing | 1.02 | 0.77 | 1 | 0.85-1.17 | 0.99 |
| Maternal age at enrollment | 0.92 | 0.03 |  |  |  |
| Maternal height at enrollment | 0.99 | 0.72 |  |  |  |
| Maternal weight at enrollment | 1.01 | 0.67 |  |  |  |
| Maternal BMI at enrollment | 1.07 | 0.36 |  |  |  |
| Maternal BMI at enrollment |  |  |  |  |  |
| 1st tertile vs. 3^rd^ tertile | 0.69 | 0.67 |  |  |  |
| 2nd tertile vs. 3^rd^ tertile | 0.68 | 0.62 |  |  |  |
| Less than primary school education | 0.73 | 0.49 |  |  |  |
| Weekly weight gain (1kg increments) | 0.22 | 0.09 |  |  |  |
| Weekly weight gain |  |  |  |  |  |
| < 25th percentile of gainers vs. losers | 0.34 | 0.45 |  |  |  |
| ≥ 25th percentile of gainers vs. losers | 0.23 | 0.019 |  |  |  |
| Weekly weight gain < 0.1 kg | 2.25 | 0.03 | 2.24 | 0.96-5.22 | 0.06 |
| Weekly weight gain < 0.2 kg | 1.46 | 0.32 |  |  |  |
| Weight gain vs. weight loss | 0.26 | 0.007 |  |  |  |
| Total weight gained (kg) | 0.88 | 0.03 |  |  |  |
| Unsuppressed viral load at delivery | 0.95 | 0.95 |  |  |  |
| Gestational age at enrollment | 1.09 | 0.1 |  |  |  |
| Duration of days of TS prior to enrollment | 0.999 | 0.67 |  |  |  |
| Duration of days of TS prior to enrollment |  |  |  |  |  |
| 1-30 vs. none | 0.87 | 0.92 |  |  |  |
| 31+ vs. none | 0.71 | 0.52 |  |  |  |
| Total duration of TS days | 0.99 | 0.15 |  |  |  |
| Maternal weight at 5 months gestation | 1.01 | 0.8 |  |  |  |
| Maternal weight at 7 months gestation | 1 | 0.95 |  |  |  |
| Mean BMI at 5 months | 1.1 | 0.43 |  |  |  |
| Mean BMI at 7 months | 1.03 | 0.71 |  |  |  |
| Weekly weight gain, 2nd trimester only | 1.05 | 0.88 |  |  |  |
| Weekly weight gain, 3rd trimester only | 0.38 | 0.2 |  |  |  |
| Season of birth |  |  |  |  |  |
| June to October | 0.7 | 0.38 |  |  |  |
| November to May | 1 | - |  |  |  |
| Incident clinical malaria |  |  |  |  |  |
| None | 1.63 | 0.54 |  |  |  |
| One or more episodes | 1 | - |  |  |  |
| 3 or 4 AE's | <0.001 | 0.99 |  |  |  |
| Higher SES | 1.3 | 0.51 |  |  |  |
