# Supplementary material for: Maternal Nutritional Status Predicts Adverse Birth Outcomes among HIV-Infected Rural Ugandan Women Receiving Combination Antiretroviral Therapy
Source: PLoS One. 2012 Aug 7;7(8):e41934. doi: 10.1371/journal.pone.0041934 (PMC3413694; doi:10.1371/journal.pone.0041934)
Supplement: Table S6 — Univariate and multivariate logistic regression models of preterm delivery. (DOC) [file pone.0041934.s007.doc]

Table S6. Univariate and multivariate logistic regression models of preterm delivery.

| Preterm Delivery <37 weeks, N=150 | Univariate Model | | Final Multivariable Model | | |
| --- | --- | --- | --- | --- | --- |
|  | OR | p-value | OR | 95% CI | p-value |
| History of preterm delivery (no vs. yes) | 1.26 | 0.83 | 1.67 | 0.15-18.80 | 0.68 |
| Sex of infant (male vs. female) | 0.75 | 0.52 |  |  |  |
| Log(10)viral load at enrollment | 2.05 | 0.015 |  |  |  |
| CD4 at screening | 0.999 | 0.39 | 0.998 | 0.99-1.00 | 0.23 |
| CD4 at screening (categorical) |  |  |  |  |  |
| 200-350 vs. <200 | 0.28 | 0.09 |  |  |  |
| >350 vs. <200 | 0.5 | 0.91 |  |  |  |
| CD4 nadir | 1 | 0.64 |  |  |  |
| Hemoglobin at baseline | 0.93 | 0.72 |  |  |  |
| Hemoglobin at baseline |  |  |  |  |  |
| <8.5 vs. >11 | 1.52 | 0.77 |  |  |  |
| 8.5-10.999 vs. >11 | 1.17 | 0.94 |  |  |  |
| Mean hemoglobin throughout pregnancy | 0.88 | 0.56 |  |  |  |
| WHO stage at enrollment |  |  |  |  |  |
| Stage 1 vs. Stage 3 | >999.99 | 0.99 |  |  |  |
| Stage 2 vs. Stage 3 | >999.99 | 0.98 |  |  |  |
| Primigravida vs. multigravida | <0.001 | 0.98 |  |  |  |
| Birth spacing | 0.98 | 0.83 |  |  |  |
| Birth spacing: <2 years vs. nullipara or > 2 years | 1.67 | 0.54 | 2.52 | 0.35-17.93 | 0.36 |
| Maternal age at enrollment | 1.03 | 0.46 |  |  |  |
| Maternal height at enrollment | 0.93 | 0.03 |  |  |  |
| Maternal weight at enrollment | 0.96 | 0.19 | 0.93 | 0.86-0.99 | 0.048 |
| Maternal BMI at enrollment | 1.05 | 0.54 |  |  |  |
| Maternal BMI at enrollment |  |  |  |  |  |
| 1st tertile vs. 3^rd^ tertile | 0.46 | 0.85 |  |  |  |
| 2nd tertile vs. 3^rd^ tertile | 0.26 | 0.11 |  |  |  |
| Less than primary school education | 2.78 | 0.18 |  |  |  |
| Weekly weight gain (1kg increments) | 0.095 | 0.04 |  |  |  |
| Weekly weight gain |  |  |  |  |  |
| < 25th percentile of gainers vs. losers | 0.41 | 0.85 |  |  |  |
| ≥ 25th percentile of gainers vs. losers | 0.21 | 0.02 |  |  |  |
| Weekly weight gain < 0.1 kg | 2.89 | 0.02 | 3.46 | 1.18-10.15 | 0.024 |
| Weekly weight gain < 0.2 kg | 1.87 | 0.20 |  |  |  |
| Weight gain vs. weight loss | 0.26 | 0.01 |  |  |  |
| Total weight gained (kg) | 0.82 | 0.01 |  |  |  |
| Unsuppressed viral load at delivery | 0.96 | 0.96 |  |  |  |
| Gestational age at enrollment | 1.03 | 0.60 |  |  |  |
| Duration of days of TS prior to enrollment | 0.99 | 0.46 |  |  |  |
| Duration of days of TS prior to enrollment |  |  |  |  |  |
| 1-30 vs. none | 2.42 | 0.09 |  |  |  |
| 31+ vs. none | 1.19 | 0.63 |  |  |  |
| Total duration of TS days | 0.98 | 0.0004 | 0.97 | 0.96-0.99 | 0.0004 |
| Maternal weight at 5 months gestation | 0.96 | 0.34 |  |  |  |
| Maternal weight at 7 months gestation | 0.97 | 0.30 |  |  |  |
| Mean BMI at 5 months | 1 | 0.99 |  |  |  |
| Mean BMI at 7 months | 1.07 | 0.43 |  |  |  |
| Weekly weight gain, 2nd trimester only | 2.4 | 0.21 |  |  |  |
| Weekly weight gain, 3rd trimester only | 0.16 | 0.05 |  |  |  |
| Season of birth |  |  |  |  |  |
| June to October | 0.44 | 0.13 |  |  |  |
| November to May | 1 | - |  |  |  |
| Incident clinical malaria |  |  |  |  |  |
| None | 0.88 | 0.88 |  |  |  |
| One or more episodes | 1 | - |  |  |  |
| 3 or 4 AE's | <0.001 | 0.99 |  |  |  |
| Higher SES | 2.17 | 0.10 |  |  |  |
