# Supplementary material for: Maternal Nutritional Status Predicts Adverse Birth Outcomes among HIV-Infected Rural Ugandan Women Receiving Combination Antiretroviral Therapy
Source: PLoS One. 2012 Aug 7;7(8):e41934. doi: 10.1371/journal.pone.0041934 (PMC3413694; doi:10.1371/journal.pone.0041934)
Supplement: Protocol S1 — Trial protocol. (DOC) [file pone.0041934.s008.doc]

**“PREVENTION OF MALARIA AND HIV DISEASE IN TORORO (PROMOTE)”**

**A UCSF/MAKERERE UNIVERSITY COLLABORATION**

**“PROMOTE-PIs (Pregnant women and Infants)”**

**PROTEASE INHIBITORS TO REDUCE MALARIA MORBIDITY IN HIV-INFECTED PREGNANT WOMEN**

**VERSION 4.0**

Sponsored by:

The National Institute of Child Health and Human Development (NICHD)

Provision of Lopinavir/ritonavir by

Abbott Laboratories

Study Chair and Principal Investigator: Diane V. Havlir, MD

University California San Francisco

Study Vice-Chairs: Deborah Cohan, MD, MPH

University California San Francisco

Pius Okong, MMed, PhD

Ugandan Ministry of Health

Principal MU Investigator: Moses Kamya, MBChB, MMed, MPH

Makerere University

**TABLE OF CONTENTS**

Page

[PROTOCOL TEAM ROSTER 7](#__RefHeading___Toc289703951)

[GLOSSARY 10](#__RefHeading___Toc289703952)

[SCHEMA 12](#__RefHeading___Toc289703953)

[1.0 INTRODUCTION 14](#__RefHeading___Toc289703954)

[1.1 Background 14](#__RefHeading___Toc289703955)

[**Table 1. Prevalence of maternal anemia according to HIV and malaria status at delivery** 17](#__RefHeading___Toc289703956)

[**Table 2. Low birth weight according to maternal HIV and malaria status at delivery** 18](#__RefHeading___Toc289703957)

[**Table 3. Risk of placental malaria among HIV-infected women receiving malaria IPT** 20](#__RefHeading___Toc289703958)

[1.2 Rationale 26](#__RefHeading___Toc289703959)

[1.3 Antiretroviral Therapy 28](#__RefHeading___Toc289703960)

[**1.3.1**  **ART Programs in Uganda** 28](#__RefHeading___Toc289703961)

[**1.3.2**  **ART agent: Efavirenz** 28](#__RefHeading___Toc289703962)

[**1.3.3**  **ART agent: Lopinavir/ritonavir (Aluvia)** 29](#__RefHeading___Toc289703963)

[**1.3.4**  **ART agent: Zidovudine (ZDV)** 29](#__RefHeading___Toc289703964)

[**1.3.5**  **ART agent: Stavudine (d4T)** 30](#__RefHeading___Toc289703965)

[**1.3.6**  **ART agent: Lamivudine (3TC)** 30](#__RefHeading___Toc289703966)

[**1.3.7** **ART agent: Tenofovir (TDF)** 30](#__RefHeading___Toc289703967)

[**1.3.8** **ART agent: Emtricitabine (FTC)** 31](#__RefHeading___Toc289703968)

[2.0 STUDY OBJECTIVES 31](#__RefHeading___Toc289703969)

[2.1 Primary Objective 31](#__RefHeading___Toc289703970)

[2.2 Secondary Objectives 31](#__RefHeading___Toc289703971)

[3.0 STUDY DESIGN 33](#__RefHeading___Toc289703972)

[**Table 4. Treatment arms** 33](#__RefHeading___Toc289703973)

[4.0 SELECTION AND ENROLLMENT OF SUBJECTS 34](#__RefHeading___Toc289703974)

[4.1 Inclusion criteria 34](#__RefHeading___Toc289703975)

[4.2 Exclusion criteria 34](#__RefHeading___Toc289703976)

[4.3 Identification and recruitment of study participants 35](#__RefHeading___Toc289703977)

[4.4 Screening of potential study participants 36](#__RefHeading___Toc289703978)

[4.5 Study informed consent 37](#__RefHeading___Toc289703979)

[4.6 Enrollment and Baseline Evaluation 37](#__RefHeading___Toc289703980)

[5.0 STUDY TREATMENT 38](#__RefHeading___Toc289703981)

[5.1 Treatment Group Assignments 38](#__RefHeading___Toc289703982)

[5.2 Treatment Allocation 39](#__RefHeading___Toc289703983)

[5.3 Study Drug Dosing and Formulations 39](#__RefHeading___Toc289703984)

[**5.3.1** **Maternal Drug Administration and Duration** 39](#__RefHeading___Toc289703985)

[**Table 5. Study drug formulations** 39](#__RefHeading___Toc289703986)

[**5.3.2 Infant Drug Regimens, Administration and Duration** 41](#__RefHeading___Toc289703987)

[5.4 Missed Doses of Study Drugs for both Women and Infants 42](#__RefHeading___Toc289703988)

[5.5 Drug Supply, Distribution, and Pharmacy 42](#__RefHeading___Toc289703989)

[**5.5.1** **Study drug supply** 42](#__RefHeading___Toc289703990)

[**5.5.2** **Study drug distribution** 42](#__RefHeading___Toc289703991)

[**5.5.3** **Agent accountability** 42](#__RefHeading___Toc289703992)

[6.0 STUDY EVALUATIONS AND CLINICAL MANAGEMENT 43](#__RefHeading___Toc289703993)

[6.1 Schedule of evaluations and interventions for women 43](#__RefHeading___Toc289703994)

[**6.1.1** **Antepartum visits** 43](#__RefHeading___Toc289703995)

[**6.1.2** **Delivery visit** 43](#__RefHeading___Toc289703996)

[**6.1.3** **Postpartum visits** 44](#__RefHeading___Toc289703997)

[**6.1.4** **Standard Prenatal Care in Uganda** 44](#__RefHeading___Toc289703998)

[**6.1.5. Nutritional visits** 45](#__RefHeading___Toc289703999)

[6.2 Schedule of evaluations and interventions for infants 45](#__RefHeading___Toc289704000)

[**6.2.1** **Delivery visit** 45](#__RefHeading___Toc289704001)

[**6.2.2** **Postpartum visits** 46](#__RefHeading___Toc289704002)

[**6.2.3** **Standard Care of Newborns in Uganda** 46](#__RefHeading___Toc289704003)

[**6.2.4. Study visits at 18 months and 24 months postpartum after study discontinuation** 46](#__RefHeading___Toc289704004)

[6.3. Diagnosis and treatment of concurrent illnesses 47](#__RefHeading___Toc289704005)

[**6.3.1** **Malaria** 47](#__RefHeading___Toc289704006)

[**6.3.1.1** **Diagnosis of malaria** 47](#__RefHeading___Toc289704007)

[**6.3.1.2** **Management of malaria** 47](#__RefHeading___Toc289704008)

[**6.3.1.3** **Diagnostic Testing for Malaria** 48](#__RefHeading___Toc289704009)

[**Table 6. Categories of placental malaria** 49](#__RefHeading___Toc289704010)

[**6.3.2** **Tuberculosis (TB)** 50](#__RefHeading___Toc289704011)

[6.4 Medical care outside of the study clinic 50](#__RefHeading___Toc289704012)

[6.5 After hours visits 50](#__RefHeading___Toc289704013)

[6.6 Co-enrollment Guidelines 51](#__RefHeading___Toc289704014)

[7.0 TOXICITY MANAGEMENT 51](#__RefHeading___Toc289704015)

[7.1 Toxicity management for women by toxicity grade 52](#__RefHeading___Toc289704016)

[7.2 Toxicity management for women by diagnosis 53](#__RefHeading___Toc289704017)

[**7.2.1** **Nausea/vomiting** 53](#__RefHeading___Toc289704018)

[**7.2.2** **Diarrhea** 54](#__RefHeading___Toc289704019)

[**7.2.3** **Zidovudine intolerance** 54](#__RefHeading___Toc289704020)

[**7.2.4** **Rash** 55](#__RefHeading___Toc289704021)

[**7.2.5 Electrocardiogram (ECG) abnormality** 55](#__RefHeading___Toc289704022)

[7.3 Toxicity management for women by laboratory abnormality 55](#__RefHeading___Toc289704023)

[**7.3.1** **Anemia** 55](#__RefHeading___Toc289704024)

[**7.3.2** **Hepatotoxicity** 55](#__RefHeading___Toc289704025)

[**7.3.3** **Hyperglycemia** 56](#__RefHeading___Toc289704026)

[**7.3.4** **Neutropenia** 56](#__RefHeading___Toc289704027)

[**7.3.5** **Proteinuria** 56](#__RefHeading___Toc289704028)

[**7.3.6** **Fetal or infant demise after study entry** 56](#__RefHeading___Toc289704029)

[7.4 Toxicity management for infants by toxicity grade 57](#__RefHeading___Toc289704030)

[7.5 Subject management plan 59](#__RefHeading___Toc289704031)

[**7.5.1** **Criteria for virologic failure** 59](#__RefHeading___Toc289704032)

[**7.5.2** **Management of virologic failure** 59](#__RefHeading___Toc289704033)

[**7.5.3** **Subsequent pregnancy management** 59](#__RefHeading___Toc289704034)

[7.6 Allowed medications 59](#__RefHeading___Toc289704035)

[7.7 Disallowed medications 60](#__RefHeading___Toc289704036)

[7.8 Criteria for study discontinuation 60](#__RefHeading___Toc289704037)

[7.9 Management of antiretroviral medication upon study discontinuation or end of study 60](#__RefHeading___Toc289704038)

[8.0 MONITORING OF ADVERSE EVENTS AND MANAGEMENT 61](#__RefHeading___Toc289704039)

[8.1 Monitoring and Reporting of Adverse Events 61](#__RefHeading___Toc289704040)

[**8.1.1** **Definitions** 61](#__RefHeading___Toc289704041)

[**8.1.2** **Identification of Adverse Events** 61](#__RefHeading___Toc289704042)

[**8.1.3** **Reporting of Adverse Events** 62](#__RefHeading___Toc289704043)

[**Table 7. Guidelines for reporting adverse events** 62](#__RefHeading___Toc289704044)

[• Event that changes the risk/benefit ratio of the study. 63](#__RefHeading___Toc289704045)

[9.0 STATISTICAL CONSIDERATIONS 63](#__RefHeading___Toc289704046)

[9.1 General design issues 63](#__RefHeading___Toc289704047)

[9.2 Outcome measures 65](#__RefHeading___Toc289704048)

[**9.2.1** **Primary outcome measure** 65](#__RefHeading___Toc289704049)

[**9.2.2** **Secondary outcome measures** 65](#__RefHeading___Toc289704050)

[**9.2.3** **Pharmacokinetics in women and infants** 66](#__RefHeading___Toc289704051)

[9.3 Randomization and stratification 67](#__RefHeading___Toc289704052)

[9.4 Sample size and accrual 67](#__RefHeading___Toc289704053)

[**Table 8. Range of protective efficacies detectable given an evaluable sample size of 215 per arm** 68](#__RefHeading___Toc289704054)

[**Table 9. Estimated accrual per year** 68](#__RefHeading___Toc289704055)

[9.5 Monitoring 68](#__RefHeading___Toc289704056)

[**9.5.1** **Data Safety Monitoring Board** 68](#__RefHeading___Toc289704057)

[**9.5.2** **Interim analysis and stopping guidelines** 69](#__RefHeading___Toc289704058)

[**Table 10. Schedule of 2 interim analyses and final analyses: boundaries to monitor study outcomes*** 69](#__RefHeading___Toc289704059)

[9.6 Analysis 69](#__RefHeading___Toc289704060)

[**9.6.1** **Primary analysis** 69](#__RefHeading___Toc289704061)

[**9.6.2** **Secondary analysis** 70](#__RefHeading___Toc289704062)

[10.0 DATA COLLECTION AND MONITORING 74](#__RefHeading___Toc289704063)

[10.1 Record Keeping 74](#__RefHeading___Toc289704064)

[10.2 Data Quality Assurance and Monitoring 74](#__RefHeading___Toc289704065)

[11.0 HUMAN SUBJECTS 74](#__RefHeading___Toc289704066)

[11.1 Treatment and Compensation for Injury 74](#__RefHeading___Toc289704067)

[11.2 Costs to the Subjects 74](#__RefHeading___Toc289704068)

[11.3 Reimbursement of Subjects 75](#__RefHeading___Toc289704069)

[11.4 Institutional Review Board (IRB) Review and Informed Consent 75](#__RefHeading___Toc289704070)

[11.5 Study Discontinuation 75](#__RefHeading___Toc289704071)

[12.0 PUBLICATION OF RESEARCH FINDINGS 76](#__RefHeading___Toc289704072)

[13.0 BIOHAZARD CONTAINMENT 76](#__RefHeading___Toc289704073)

[14.0 REFERENCES 76](#__RefHeading___Toc289704074)

[APPENDICES 93](#__RefHeading___Toc289704075)

[Appendix I. Schedule of Clinical and Laboratory Evaluations for Women 93](#__RefHeading___Toc289704076)

[Appendix II. Schedule of Clinical and Laboratory Evaluations for Infants^8^ 98](#__RefHeading___Toc289704077)

[Appendix III. Precautionary Medications 100](#__RefHeading___Toc289704078)

[Appendix IV. Patient Information Sheet 102](#__RefHeading___Toc289704079)

[Appendix V. Determination of Gestational Age 103](#__RefHeading___Toc289704080)

[Appendix VI. Informed Consent to be Screened for a Clinical Study 104](#__RefHeading___Toc289704081)

[Appendix VII. Research Participation Informed Consent Form 107](#__RefHeading___Toc289704082)

[Appendix VIII. Future Use of Biological Specimens Informed Consent 123](#__RefHeading___Toc289704083)

[Appendix IX. Uganda Ministry of Health Guidelines for Routine Care of Pregnant and Postpartum Women, and Newborns 126](#__RefHeading___Toc289704084)

[Appendix X. DAIDS Toxicity Table (Dec 2004, clarification Aug 2009) 128](#__RefHeading___Toc289704085)

[Appendix XI. Supplemental Toxicity Table for Grading Severity of Adult and Pediatric Cutaneous/Skin Rash/Dermatitis Adverse Experiences 147](#__RefHeading___Toc289704086)

[Appendix XII. Pharmacokinetics using Plasma and Hair Collection from Woman and Infants 148](#__RefHeading___Toc289704087)

[Appendix XIII. Radio Announcement Script 150](#__RefHeading___Toc289704088)

[Appendix XIV. Food insecurity, and water and sanitation questions for Nutrition Interview (Version March 31, 2011) 151](#__RefHeading___Toc289704089)

[Appendix XV. Nutrition questions for focus group discussion or open-ended interviews (Version March 31, 2011) 154](#__RefHeading___Toc289704090)

[Appendix XVI. Informed Consent to Participate in a Nutrition Interview or Focus Group 156](#__RefHeading___Toc289704091)

[Appendix XVII. Informed Consent to Participate in SubStudy after Main Study Participation 159](#__RefHeading___Toc289704092)

[Appendix XVIII. DMID Pediatric Toxicity Tables 163](#__RefHeading___Toc289704093)

**PROTOCOL TEAM ROSTER**

**Investigators:**

Study Chair and Principal Investigator:

Diane V. Havlir, MD, University of California, San Francisco

Address: San Francisco General Hospital, 995 Potrero Avenue,

Building 80, Ward 84, UCSF Box 0874, San Francisco, CA 94110, U.S.A.

Phone Number:  01-415-476-4082, ext 400

Fax Number:      01-415-502-2992

Email:  [dhavlir@php.ucsf.edu](mailto:dhavlir@php.ucsf.edu)

Study Vice-Chairs:

Deborah Cohan, MD MPH, University of California, San Francisco

Address: San Francisco General Hospital, 1001 Potrero Avenue,

Ward 6D-22, UCSF Box 0842, San Francisco, CA 94110, U.S.A.

Phone Number: 01-415-206-3658

Fax Number: 01-415-206-3112

Email: [cohand@obgyn.ucsf.edu](mailto:cohand@obgyn.ucsf.edu)

Pius Okong, MMed, PhD, Ugandan Ministry of Health

Address: St. Francis Hospital Nsambya, P.O. Box 7146, Kampala, Uganda

Phone Number: +256-41-267012

Fax Number: +256-41-267870

Email: [pio_okong@yahoo.co.uk](mailto:pio_okong@yahoo.co.uk)

Principal MU Investigator:

Moses R. Kamya, MBChB, MMed, MPH Makerere University, Kampala, Uganda

            Address:  Department of Medicine, P.O. Box 7072, Kampala, Uganda

            Phone Number: +256-414-533200

            Fax Number: +256-414-540524

            Email: [mkamya@infocom.co.ug](mailto:mkamya@infocom.co.ug)

Study Investigators:

Jane Achan, MBChB, MPed, Makerere University, Kampala, Uganda

Address: MU-UCSF Research Collaboration,

P.O. Box 7475, Kampala, Uganda

Phone Number: +256-414-530692

Fax Number: +256-414-540524

Email:  [achanj@yahoo.co.uk](mailto:achanj@yahoo.co.uk)

Veronica Ades, MD, MPH, University of California, San Francisco

Address: San Francisco General Hospital, 1001 Potrero Avenue,

UCSF Box 0842, San Francisco, CA 94110, U.S.A.

Phone Number: 01-415-206-3658

Fax Number: 01-415-206-3112

Email: [adesv@globalhealth.ucsf.edu](mailto:adesv@globalhealth.ucsf.edu)

Edwin D. Charlebois, MPH, PhD University of California, San Francisco

Address:  50 Beale St., 13^th^ Floor, San Francisco, CA 94105, U.S.A.

Phone Number: 01-415-597-9301

Fax Number:  01-415-597-9213

Email:  [edwin.charlebois@ucsf.edu](mailto:echarlebois@psg.ucsf.edu)

Tamara Clark, MPH, University of California, San Francisco

            Address:  UCSF, Parnassus Avenue Box 0811, San Francisco, CA  94143, U.S.A.

Phone Number: 01-415-206-8790

Fax Number: 01-415-648-8425

            Email:   [tclark@medsfgh.ucsf.edu](mailto:gdorsey@medsfgh.ucsf.edu)

Grant Dorsey, MD, PhD, University of California, San Francisco

            Address:  UCSF, Parnassus Avenue Box 0811, San Francisco, CA  94143, U.S.A.

Phone Number: 01-415-206-4680

Fax Number: 01-415-648-8425

            Email:   [gdorsey@medsfgh.ucsf.edu](mailto:gdorsey@medsfgh.ucsf.edu)

Anne Gasasira, MBChB, MPH, Makerere University, Kampala, Uganda

Address: MU-UCSF Research Collaboration,

P.O. Box 7475, Kampala, Uganda

Phone Number: +256-414-530692

Fax Number: +256-414-540524

Email:  [agasasira@berkeley.edu](mailto:agasasira@berkeley.edu), [agasasira@gmail.com](mailto:agasasira@gmail.com)

Lena Kim, MD, Fellow, Division of Maternal-Fetal Medicine, Department of Obstetrics,

Gynecology and Reproductive Sciences

Address: UCSF, 500 Parnassus Avenue, M1495B, Campus Box 0132, San Francisco, CA 94143-0132

Phone Number: 01-415 353 1256

Fax Number: 01-415 476 1811

Email: [kimlh@obgyn.ucsf.edu](mailto:kimlh@obgyn.ucsf.edu)

Philip Rosenthal, MD, University of California, San Francisco

Address:  UCSF, Parnassus Avenue Box 0811, San Francisco, CA  94143, U.S.A.

Phone Number: 01-415-206-8845

Fax Number: 01-415-648-8425

Email: [prosenthal@medsfgh.ucsf.edu](mailto:prosenthal@medsfgh.ucsf.edu)

Theodore Ruel, MD, Assistant Professor, Division of Infectious Disease, Dept Pediatrics

Address: UCSF, 500 Parnassus Avenue, MU4E, Campus Box 0136, San Francisco, CA 94143-0136

Phone Number: 01-415 476 9198

Fax Number: 01-415 476 1343

Email:  [ruelt@peds.ucsf.edu](mailto:ruelt@peds.ucsf.edu)

Sera Young, PhD

Address: Cornell University, Division of Nutritional Sciences, Savage Hall, Ithaca, NY 14853

Phone Number: 607 351 0172

Fax Number: 607 255 1033

Email: sly3@cornell.edu

OTHER KEY PERSONNEL

| Name | Title | Institution |
| --- | --- | --- |
| Dr. Julia Mwesigwa | Senior Study Coordinator | IDRC, Uganda |
| Dr. Paul Natureeba | Junior Study Coordinator | IDRC, Uganda |
| Dr. Bridget Nzarubara | Regulatory Affairs Manager | IDRC, Uganda |
| Beth Osterbauer | Project Coordinator | UCSF |
| Barnabas Natamba | Nutritionalist | Cornell University |
| Dr. Rebecca Stoltzfus | Nutritionalist | Cornell University |

**GLOSSARY**

ACTG AIDS Clinical Trials Group

AACTG Adult AIDS Clinical Trials Group

AE Adverse event

AL Artemether-lumefantrine

ALT Alanine transaminase (SGPT)

ART Antiretroviral therapy

ARV Antiretroviral

AS Artesunate

AST Aspartate aminotransferase (SGOT)

AUC Area under the curve

AZ Azithromycin

BID Twice a day

CAB Community Advisory Board

CBC Complete blood count

CDC Center for Disease Control

CFAR Center for AIDS Research

CHR Committee for Human Research

CI Confidence Interval

Cmax Maximum plasma concentration of a drug

Cmin Trough serum concentration of a drug

COC Combined oral contraceptive pills

CQ Choloroquine

Cr Creatinine

CRF Case report form

DAIDS Division of AIDS

DMC Data Management Centre

d4T Stavudine

DMPA Depot medroxyprogesterone acetate

DSMB Data and Safety Monitoring Board

EIA Enzyme-linked immunoassay

EFV Efavirenz

FTC Emtricitabine

H Hour

HAART Highly active antiretroviral therapy

Hg Hemoglobin

HIV Human Immunodeficiency Virus

IUD Intrauterine device

IPT Intermittent preventive therapy (for malaria)

IRB Institutional review board

ITN Insecticide treated net

IUFD Intrauterine fetal demise (> 20 weeks gestation)

IUGR Intrauterine growth restriction

LBW Low birth weight (<2500g)

LMP Last menstrual period (first day)

LPV Lopinavir

LPV/r Lopinavir/ritonavir

MOH Ministry of Health

MTCT Maternal to child transmission

MU Makerere University

NDA Uganda National Drug Authority

NICHD National Institute of Child Health and Human Development

NIH National Institutes of Health

NNRTI Nonnucleoside reverse transcriptase inhibitor

NRTI Nucleoside reverse transcriptase inhibitor

NVP Nevirapine

OR Odds ratio

PACTG Pediatric AIDS Clinical Trials Group

PCR Polymerase chain reaction

POP Progesterone-only pills

RDT Rapid diagnostic test

PI Protease inhibitor

PMTCT Prevention of mother to child transmission

PO Oral

PTD Preterm delivery (<37 weeks gestation)

Q Every

QD Once every day

RDT Rapid diagnostic test

RPR Rapid plasma regain test

RR Relative Risk

RTV Ritonavir

SD Single dose

SP Sulphadoxine-pyrimethamine

3TC Lamivudine

TASO The AIDS Support Organization

TB Tuberculosis

TDF Tenofovir

TDH Tororo District Hospital

TDM Therapeutic Drug Monitoring

TS Trimethoprim-sulfamethoxazole

UCSF University of California, San Francisco

UNCST Ugandan National Council for Science and Technology

VDRL Venereal Disease Research Laboratory

WHO World Health Organization

WIHS Women's Interagency HIV Study

ZDV Zidovudine

**SCHEMA**

PROTEASE INHIBITORS TO REDUCE MALARIA MORBIDITY IN HIV-INFECTED PREGNANT WOMEN

DESIGN: Randomized Controlled Trial, open-label

SAMPLE SIZE: 500 women and their infants

POPULATION: HIV-infected pregnant women, ≥16 years old, at 12-28 weeks gestation living in a malaria endemic area who are naïve to antiretroviral therapy (ART) or received single-dose nevirapine (alone or with zidovudine, zidovudine/lamivudine or other abbreviated monotherapy or dual therapy for prevention of mother-to-child transmission of HIV [PMTCT]) more than 24 months prior to enrollment.

STRATIFICATION: Gravidity at study entry (G1 versus G2+) and gestational age at the time of enrollment (<24 weeks versus ≥ 24 weeks)

REGIMEN:

| Treatment Arm | Medications* |
| --- | --- |
| Group A | ZDV 300mg/3TC 150mg/ LPV 200mg/r 50mg |
| Group B | ZDV 300mg/3TC 150mg/ EFV 600mg |

*ZDV = zidovudine, 3TC = lamivudine, EFV=efavirenz, LPV = lopinavir, r = ritonavir

All newborns will recieve nevirapine syrup after birth for 6 weeks.

TREATMENT DURATION: Women: Study entry until 1 week following

discontinuation of breastfeeding, (but no longer than 1 year + 1 week postpartum)

STUDY DURATION: Women: Study entry until 6 weeks following the

cessation of breastfeeding but not beyond 58 weeks of age

Infants: Birth until 6 weeks following the cessation of breastfeeding but not beyond 58 weeks of age

HYPOTHESIS AND STUDY OBJECTIVES:

Protease inhibitor-based antiretroviral therapy (ART) will reduce malaria-related morbidity in pregnant and breastfeeding women.

Primary Objective:

1. To determine if the use of PI-based ART versus non-PI ART among HIV-infected pregnant women leads to reduced rate of placental malaria as measured by Giemsa-stained smear or polymerase chain reaction (PCR) of placental blood.

Secondary Objectives:

1. To determine if the use of PI-based ART versus non-PI ART among HIV-infected pregnant women leads to reduced rate of placental malaria as measured by alternate diagnostic modalities (rapid diagnostic test (RDT; immunochromatographic lateral flow test for histidine rich protein 2 [HRP2] and plasmodium lactate dehydrogenase [pLDH]) for malaria parasites in placental blood; placental histopathology).

2. To determine if the use of PI-based ART versus non-PI ART among HIV-infected pregnant women leads to reduced rate of maternal malaria and severe anemia.

3. To determine if the use of PI-based ART versus non-PI ART among HIV-infected pregnant women leads to reduced obstetrical/fetal/neonatal morbidity measured by a composite clinical outcome of infant low birth weight (LBW, <2500gm), still birth (intrauterine fetal demise > 20 weeks gestation), late spontaneous abortion (miscarriage 12-20 weeks gestation), preterm delivery (< 37 weeks, < 34 weeks, continuous variable), and neonatal death (death of liveborn infant within 28 days of birth).

4. To compare the maternal and infant safety and tolerance profile of PI-based versus non-PI ART during pregnancy and breastfeeding

5. To compare the incidence of pre-eclampsia between PI-based versus non-PI ART among HIV-infected pregnant women.

6. To compare the virologic and immunologic efficacy of PI-based versus non-PI ART in pregnant and breastfeeding women, including:

1. Prevalence of maternal HIV RNA suppression of <400 copies/mL and of <50 copies/mL at delivery and 24 weeks after the start of the treatment regimen.
2. Change in maternal CD4 cell counts and % CD4 from ART initiation to delivery and from delivery to the cessation of breastfeeding.
3. Development of one or more new maternal HIV antiretroviral resistance mutations measured at delivery, 24 weeks and 1 year postpartum.
4. Incidence of maternal to child transmission of HIV, measured by infant HIV DNA PCR at delivery, DNA PCR at each follow-up visit and at 58 weeks of life or 6 weeks following the cessation of breastfeeding (whichever comes first)

7. To assess PI-based and non-PI based ART exposure in HIV-infected women and in their infants by measuring ART levels in paired hair and blood samples (women at 30-34 weeks gestation and 12 weeks postpartum; infants at delivery, 12 weeks and 24 weeks of life).

8. To assess the association between nutritional status and obstetrical, infant and HIV-related outcomes, including ART levels among women.

**1.0 INTRODUCTION**

**1.1 Background**

**HIV and Malaria Interactions**

HIV and malaria constitute overlapping and synergistic epidemics among pregnant women in sub-Saharan Africa.^1^ Twenty-two million people are living with HIV in sub-Saharan Africa, and 61% of these are women. In some areas of sub-Saharan Africa, up to 40% of pregnant women are infected with HIV.^2^ HIV-infection is associated with worse maternal outcomes, including spontaneous abortion, stillbirth, anemia, chorioamnionitis, postpartum endometritis and mortality during pregnancy and the postpartum period.^3-10^ In the absence of ART administered to the pregnant woman, the cumulative risk of in-utero and intrapartum HIV mother to child transmission (MTCT) approaches 25%.^11-14^ Prolonged breastfeeding is associated with an additional 15% risk of MTCT.^14-21^ Worldwide, 530,000 children became infected with HIV in 2006, nearly all through MTCT.^2^ Infants born to HIV-infected mothers have low birth weight (LBW) and higher prevalence of intrauterine growth restriction (IUGR) and mortality compared to infants born to HIV-uninfected mothers.^5 22^ Even in the absence of HIV acquisition, HIV-exposed infants fare worse than those born to HIV-uninfected women, with an increased risk of serious morbidity and death.^22 23^

Malaria is one of the most prevalent infectious diseases in the world, with an estimated 515 million cases in 2002 and 70-90% of cases occurring in sub-Saharan Africa.^24 25^ Numerous studies have found that over 25% of pregnant women in Africa have evidence of placental malaria and/or peripheral parasitemia.^26-29^ Infection with *P. falciparum* during pregnancy is associated with poor maternal and infant outcomes, including maternal anemia, severe and/or cerebral malaria, maternal mortality, stillbirth, spontaneous abortion, IUGR, preterm delivery, infant anemia, congenital malaria, decreased transplacental passage of protective maternal antibodies and infant mortality.^27 28^ The WHO estimates that 10,000 women and 200,000 infants die annually due directly to maternal infection with *P. falciparum,* with an additional 573,000 African infants born with low birth weight due to placental malaria.^27 29-31^

HIV-infected adults are at increased risk of clinical malaria, demonstrating not only the geographic overlap, but the synergy between the HIV and malaria epidemics.^32-34^ There are an estimated 1 million pregnant women co-infected with HIV and malaria in sub-Saharan Africa. The overlapping and synergistic morbidities of these two diseases produce enormous health and economic burdens. Strategic interventions in HIV-infected pregnant women to prevent malaria and HIV transmission represent a crucial step in improving both maternal and infant outcomes.^35^

**Malaria and Pregnancy**

Malaria during pregnancy is associated with poor maternal, obstetrical and infant outcomes. Malaria affects pregnant women living in both low and high transmission areas. In low transmission areas, primigravid and multigravid women are both at risk for severe and complicated malaria. In high transmission areas, primigravid experience the most severe episodes, but the major toll of malaria is its effect on maternal anemia and poor infant outcomes.^36^ In pregnant women living in high transmission areas, malaria magnifies the risk of anemia, contributing indirectly or directly to significant morbidity and mortality. In sub-Saharan Africa, malaria is estimated to account for 2-15% of cases of maternal anemia ^27^ and 10,000 maternal deaths due to anemia each year.^28^ In a recent survey of 553 Ugandan health care facilities with an overall maternal mortality rate of 67.1/10,000 live births, malaria was attributed as a direct or indirect cause of maternal mortality in 65% of cases.^37^ Moreover, it is estimated that malaria may account for up to 60% of spontaneous abortions in Uganda.^38-40^

In addition to its profound effects on pregnant women, malaria infection during pregnancy has severe and detrimental consequences for the fetus and infant. Malaria infection during the third trimester appears to have the most important effect on LBW and poor fetal outcomes, but infections earlier during pregnancy may also contribute.^41-43^ In a Zimbabwean cohort of 986 HIV-infected and uninfected infants, maternal *P. falciparum* infection was associated with a 10-fold increased odds of delivering a LBW infant.^44^ LBW is the strongest risk factor for neonatal and infant death,^45^ and malaria is thought responsible for 62,000-363,000 infant deaths yearly associated with LBW.^46^ Importantly, an estimated 30% of preventable cases of LBW are attributed to malaria.^47^ Moreover, infants born to women with malaria are at approximately 2-3 times the risk of having anemia.^48-50^ Maternal *P. falciparum* infection also appears to have an adverse effect on neonatal immunity.^51-54^ In summary, maternal infection with *P. falciparum* is responsible for profound and preventable morbidity among pregnant women and their infants living in endemic areas.

Placental malaria is an important mediator of the adverse effects associated with malaria. Erythrocytes infected with *P. falciparum* commonly circulate in the placenta, leading to important impacts on fetal health.^26^ In stable malaria transmission areas, women with placental malaria have approximately twice the risk of delivering a LBW infant compared to women without placental malaria.^29 55^ Placental malaria is associated with stillbirth, regardless of parity (OR 2.2, 95% CI 1.5–3.2; *P* < 0.001).^56^ In a Zimbabwean cohort adjusting for maternal HIV status, women with malaria were at a 4.7 increased odds of experiencing a stillbirth compared to those without malaria.^44^ Placental malaria is associated with a diminished transfer of protective maternal antibodies against measles, tetanus and *S. pneumoniae*.^51-54^ Placental malaria also appears to be associated with decreased infant cellular and antibody responses to *P. falciparum* epitopes and, in turn, increased risk of malaria infection in infants.^1 57-63^ In the course of evaluating new preventive strategies for malaria in pregnant women, placental malaria has emerged as a primary indicator of efficacy because of its high correlation with clinical outcomes. In addition, unlike LBW, changes in placental malaria burden are generally attributable to anti-malarial interventions, whereas many factors can affect LBW.

Placental malaria is most commonly measured as either malarial parasitemia in placental thick blood smears or by histologic assessment of placental tissue for parasites.^64-72^ Peripheral blood smears are insensitive for placental malaria.^66^ The most consistently utilized test for placental malaria, and that most demonstrably predictive of poor infant outcomes, is placental blood smears. Preliminary data we have collected among 517 HIV-infected and HIV-uninfected women delivering at Tororo District Hospital suggest that there is an association between placental malaria diagnosed by PCR and low birth weight. Therefore, for this study, we will utilize the presence of *P. falciparum* in placental blood by either blood smear or PCR as our primary outcome. Histological assessment of placental parasitemia is more technically demanding, but adds an important second measure, including characterization of the acuteness of placental infection. We will also perform histological assessment of placentas from study subjects. The maternal, obstetrical and neonatal manifestations of placental malaria appear to vary according to placental findings, with chronic placental infection most associated with anemia and low birth weight, and acute infection associated with preterm delivery.^68 70 73^ The most detailed study in this area showed strong associations between an active-chronic infection histology and both low birth weight and maternal anemia.^68^ In summary, placental malaria is an established marker for poor infant outcomes and will be used as the primary measure of efficacy in the proposed trial.

HIV magnifies the adverse sequelae of malaria among pregnant women and their infants. HIV-infected pregnant women have significant alterations in both cellular and humoral immunity to malaria. ^74 75^ As a result, HIV-infected women, regardless of parity, experience worse sequelae from malarial infection and are at increased risk of clinical and placental malaria compared to HIV-uninfected women. Numerous studies conducted in both stable and unstable malaria transmission areas have found that, among pregnant women with malaria, those with HIV are more likely to experience anemia (Table 1).^76-80^ In addition, women with HIV are more likely to experience increased malaria parasite density as compared to HIV-negative women and this, in turn, is associated with worse anemia.^79^

**Table 1. Prevalence of maternal anemia according to HIV and malaria status** at delivery

| (Adapted from ter Kuile^1^) | | | | | | | | | | | | |
| --- | --- | --- | --- | --- | --- | --- | --- | --- | --- | --- | --- | --- |
| Study | van Eijk ^78^ | | | | Ayisi ^77^ | | | | Rogerson (unpublished) | | | |
| Total N (% HIV+) | 4608 (25%) | | | | 2466 (24.3%) | | | | 454 (27.3%) | | | |
|  | Primigravida | | Multigravida | | Primigravida | | Multigravida | | Primigravida | | Multigravida | |
|  | N | % | N | % | N | % | N | % | N | % | N | % |
| No HIV/malaria | 1233 | 11.8 | 1889 | 13.6 | 607 | 10.7 | 708 | 12.1 | 89 | 20.3 | 175 | 13.1 |
| HIV alone | 286 | 18.9 | 601 | 21.8 | 147 | 19.0 | 229 | 14.0 | 51 | 34.2 | 114 | 26.3 |
| Malaria alone | 368 | 18.2 | 273 | 14.3 | 206 | 13.6 | 119 | 14.3 | 80 | 35.6 | 69 | 23.2 |
| HIV/malaria co-infection | 172 | 23.8 | 194 | 34.0 | 81 | 23.5 | 76 | 32.9 | 19 | 72.7 | 28 | 35.7 |

Moreover, pregnant women with both HIV and malaria are more likely to die than women with either HIV or malaria alone. One study of pregnant women in Zimbabwe found 50% maternal mortality for women with HIV-malaria co-infection as compared to 37.8% of women with HIV alone and 29% of women with clinical malaria alone.^44^ In addition to these poor maternal outcomes, HIV-infected women with malaria parasitemia experience an unacceptably high prevalence of preterm delivery and IUGR.^44 77 81^ In a study of 275 HIV-infected pregnant women in Tanzania, preterm delivery was experienced by 26.7% of women with maternal parasitemia at delivery as compared to 12.8% without parasitemia (p=0.02).^81^

Maternal HIV infection also magnifies the poor infant outcomes associated with maternal malaria, asymptomatic parasitemia and placental malaria. Among women with HIV, maternal parasitemia at delivery and placental parasitemia have been found to increase the odds of LBW by 2.2-2.5 (Table 2).^77 82^ Similarly, parasitemia during pregnancy has been found to nearly triple the risk of HIV-infected women delivering a baby with LBW.^81^ Villamor and colleagues found that among HIV-infected women in Tanzania, maternal parasitemia during pregnancy was a risk factor for LBW with an adjusted relative risk of 2.7. Similarly, maternal parasitemia, particularly at densities of 10^6^ cells/μL and greater, was associated with a 2.2-2.4 fold increased odds of LBW in another cohort of HIV-infected women in Tanzania.^82^ Infants born to women with both HIV and malaria are also vulnerable to death, even in the absence of MTCT, as compared to those born to women infected with HIV alone or malaria alone. Bloland found that infants born to women with both HIV and placental malaria infection had over 9 times the odds of dying compared to infants of mothers without HIV or malaria, 4.5 increased odds as compared to infants of mothers with placental malaria only, and between 2.7 and 7.7 increased odds as compared to infants of mothers with HIV only.^83^ Moreover, infant mortality was highest among Zimbabwean babies born to HIV-infected women with clinical malaria during pregnancy (14.8%) as compared to those born to HIV-infected women without malaria (9.6%) or those born to HIV-uninfected women with malaria (2.2%).^44^ In Villamor’s study of 275 HIV-infected pregnant women in Tanzania, maternal parasitemia at delivery was associated with 4 times the risk of infant mortality (8.9% if parasitemia vs. 2.3% without parasitemia, p=0.03)^81^ Data are conflicting on whether malaria increases the risk for HIV transmission.^84-89^ All women in this study will receive ART during the highest periods of HIV transmission, hence we expect low prevalence of MTCT, and our study will not be able to address the effect of different malaria prevention strategies on MTCT.

**Table 2. Low birth weight according to maternal HIV and malaria status** at delivery

|  | Ayisi^90^ | | | | Rogerson (unpublished) | | | |
| --- | --- | --- | --- | --- | --- | --- | --- | --- |
| Total N (% HIV+) | 2466 (24.3%) | | | | 454 (27.3%) | | | |
|  | Primigravida | | Multigravida | | Primigravida | | Multigravida | |
|  | N | % | N | % | N | % | N | % |
| No HIV/malaria | 676 | 4.1 | 809 | 2.7 | 30 | 21.0 | 79 | 5.1 |
| HIV alone | 160 | 7.5 | 256 | 3.9 | 38 | 23.7 | 176 | 8.0 |
| Malaria alone | 226 | 9.3 | 129 | 2.3 | 59 | 25.4 | 54 | 13.0 |
| HIV/malaria co-infection | 91 | 14.3 | 84 | 4.8 | 14 | 35.7 | 24 | 16.7 |

The vulnerability HIV-infected women experience not only occurs during pregnancy but also postpartum.^91 92^ Diagne and colleagues followed all residents of a Senegalese village prospectively for malaria exposure, parasitemia and malaria-associated morbidity.^92^ Malaria prevalence increased significantly during the second and third trimesters and peaked at 75.1 episodes per 1000 person-months in the 60 days following delivery.

HIV-infected women appear at even higher risk of postpartum malaria. Ladner and colleagues followed 228 HIV-infected and 229 HIV-uninfected pregnant women in Rwanda.^42^ They found an increased prevalence of peripheral parasitemia at the initial prenatal enrollment visit among HIV-infected women (8.0%) compared to HIV-uninfected women (3.5%, p<0.04). Moreover, the cumulative incidence during the antepartum period through 6 months postpartum was 6.2 per 100 women-months among those with HIV compared to 3.5 among those without HIV (RR 1.7, p=.0001). While malaria incidence peaked among the HIV-infected women in the latter part of pregnancy (10.7 per 100 women-months), the difference in malaria incidence between HIV-infected and uninfected women was most dramatic in the postpartum period. Between 3-6 months postpartum, malaria incidence was 7.3 per 100 women-months among women with HIV compared to 3.0 among HIV-uninfected women (RR 2.5, p=.0001). In summary, the increased risk of malaria for pregnant women is increased in the setting of HIV and continues postpartum. Thus, malaria preventive efforts aimed at HIV-infected women should be directed during pregnancy and during the post-partum period.

**Anti-Malarial Prophylactic Interventions**

Prophylactic interventions can decrease the risk of malaria during pregnancy, but the burden remains substantial in endemic areas, particularly among HIV-infected women. Anti-malarial prophylactic interventions have been used with varying success among pregnant women in malaria-endemic areas. In the past, the standard of care included weekly chloroquine (CQ) prophylaxis, though this intervention ultimately failed to significantly reduce the impact of malaria in pregnancy due to increasing *P. falciparum* resistance and poor adherence.^93-99^ Intermittent preventive therapy (IPT) with 2-doses of sulfadoxine-pyrimethamine (SP) administered during the second and third trimesters was shown to be superior to CQ at preventing placental malaria and reducing the risk of LBW and maternal anemia, and has become standard of care in most malaria-endemic areas.^100-114^ Insecticide treated nets (ITNs) have also been found to reduce the risk of malaria and its complications among pregnant women. A systematic review of randomized trials found that ITNs were associated with a 23% decreased risk of placental malaria, 33% decreased risk of fetal loss (combined outcome of fetal death/stillbirth and spontaneous abortion), and 23% decreased risk of LBW.^115^ IPT and ITNs had the highest protection when used in combination.^116^ However, these two interventions, although clearly beneficial, fall far short of eliminating risks of malaria during pregnancy.

Among HIV-infected pregnant women, only IPT with SP has been evaluated in randomized studies (Table 3). Filler and colleagues evaluated the efficacy of monthly versus 2-dose SP and found that HIV-infected women receiving 2 doses of SP-IPT were significantly more likely than those receiving monthly SP-IPT to have placental parasitemia (21.5% vs. 7.8%; RR 0.36, 95% CI 0.17-0.79).^117^ Nonetheless, there were no significant differences in the risk of LBW, spontaneous abortion and stillbirth among HIV-infected women randomized to receive monthly versus 2-dose IPT, although the combined risk of these adverse outcomes remained unacceptably high (29.2% if 2-dose IPT versus 28.8% if monthly SP). In a smaller observational cohort, Parise similarly found that 2-dose SP-IPT was inferior at preventing placental parasitemia for HIV-infected women (25% vs. 7%; p=0.007), but that monthly SP-IPT effectively reduced the placental malaria burden to less than 10%, regardless of maternal HIV status.^118^ In this study as well, the prevalence of LBW among HIV-infected women was high despite the use of 2-dose (13.2%) or monthly (10.7%) IPT. In contrast, Hamer recently demonstrated in a randomized controlled trial no difference in risk of placental malaria, as defined by histopathology, among HIV-infected women in Zambia regardless of whether they had received monthly or 2-dose SP-IPT.^119^ 26-29% of these women experienced placental malaria despite receiving IPT. While there was a trend towards lower risk of placental parasitemia among those receiving monthly versus 2-dose SP (2% versus 4%, RR 0.55; 95% CI 0.17-1.79), the study was conducted in an area of low malaria transmission and was underpowered to determine statistical significance. In summary, in areas of high transmission similar to the site for our proposed study, risks for placental malaria and LBW have remained substantial even with the use of SP-IPT and ITNs.

**Table 3. Risk of placental malaria among HIV-infected women receiving malaria IPT**

| **Study** | **N** | **Placental malaria definition** | **Intervention** | **Prevalence placental malaria** |
| --- | --- | --- | --- | --- |
| Filler ^117^ | 266 | Placental smear | 2 dose vs. monthly SP | 21.5% 2-dose vs. 7.8% monthly |
| Hamer ^119^ | 456 | Histology  Placental smear | 2-dose vs. monthly SP | Histology: 29% 2-dose vs. 26% monthly  Smear: 4% 2-dose vs. 2% monthly |
| Kalilani ^120^ | 141* | Histology  Placental smear | SP vs. SP/AZ vs. SP/AS | Histology*: 45-50%, for all regimens  Smear*: 16.1% SP, 27.3% SP/AZ, 11.4% SP/AS |
| Inion ^87^ | 372 | Histology | 2-dose SP | 9.1% |
| Parise ^118^ | 67 | Placental smear | 2-dose vs. monthly SP | 25.6% 2-dose vs. 7.1% monthly |
| van Eijk ^112^ | 139 | Placental smear | 1-dose vs. ≥2-dose SP | 7.1% 1-dose vs. 12.7% 2-dose |

AZ=azithromycin; AS=artesunate; * 29% of women HIV-infected and did not stratify results by maternal HIV status

Daily trimethoprim-sulfamethoxazole (TS) has been associated with improved HIV-associated morbidity and mortality, including studies of pregnant women.^121-125^ Studies in Africa showed a benefit across a wide range of CD4 cell counts. At present, the WHO recommends daily TS for adults and adolescents, including those who are pregnant, with CD4 counts below 350, WHO stage 2 if no CD4 is available, and/or WHO stage 3 or 4.^30^ ^126 127^ Some countries, such as Uganda, have extended the use of TS to HIV-infected persons for all ranges of CD4 cell counts based on the data referenced above. The Uganda Ministry of Health (MOH) recommends daily TS for all pregnant HIV-infected women, regardless of CD4 count.^128^ In our review of the literature, daily TS has not been evaluated or compared to SP-IPT as a malaria preventive intervention, even though it is standard of care worldwide. In addition, it is recommended that SP-IPT not be given to those already receiving daily TS due to an increased risk of toxicity.^129^ A comparative trial of SP-IPT vs. daily TS is not now feasible, unless it is limited to women with high CD4 cell counts (≥350) in a country not already recommending TS for these women. Clearly, daily TS and SP-IPT offer distinct approaches to malaria prevention. TS provides continuous sub-therapeutic anti-malarial exposure with a relatively weak anti-malarial. IPT with SP, the more proven regimen, provides infrequent full therapeutic doses of a more potent anti-malarial. By necessity, all Ugandan HIV-infected pregnant women will receive TS, and not SP-IPT. In any event, available data demonstrate that neither daily TS nor IPT-SP are enough to adequately prevent malaria morbidity in high transmission areas. As an additional concern, while not well-studied, standard daily folic acid supplementation in pregnant women may abrogate the efficacy of daily TS or IPT-SP in preventing placental malaria.^130-132^ The limitations of TS and SP-IPT highlight the importance of our proposed trial, which will explore an independent means of decreasing malaria incidence with PI-based ART.

Studies of pregnant women living in high malaria transmission areas demonstrate continued unacceptably high levels of placental malaria and poor infant outcomes such as LBW, even with the use of IPT and ITNs. There are no available data on the effect of TS on placental malaria. However, data from Tororo (our proposed study site), shows that even with the use of TS, ITNs, and either ART or prevention of MTCT (PMTCT) regimens in HIV-infected pregnant women, rates of poor maternal and infant outcomes remain high. Our preliminary data from Tororo also show continued high incidence of malaria in children despite TS and ITNs. Finally, our data from Tororo demonstrate very high prevalence of antifolate resistance-conferring mutations in infecting malaria parasites. High and increasing levels of resistance to TS may explain the persistently high incidence of malaria among children receiving this intervention and the continued high rates of poor maternal and infant outcomes among pregnant women receiving daily TS. Given the vulnerability of HIV-infected pregnant women and their infants to the detrimental effects of malaria, new strategic interventions merit urgent evaluation among HIV-infected pregnant women living in high malaria transmission areas.

**HIV Protease Inhibitors**

HIV protease inhibitors (PIs) inhibit *P. falciparum* in vitro.^133-138^ HIV PIs target the HIV protease, a member of the aspartic protease family. *P. falciparum* expresses a number of aspartic proteases, known as plasmepsins. Skinner-Adams demonstrated that saquinavir, ritonavir, and indinavir inhibited *P. falciparum* in vitro.^138^ Parikh extended these observations showing that lopinavir was the most potent HIV PI against *P. falciparum*, and that in vitro inhibitory concentrations were 10 fold below plasma concentrations achieved in vivo with lopinavir/ritonavir boosted combinations.^133^ In addition, Parikh demonstrated inhibition of purified plasmepsin II by lopinavir. Andrews confirmed activity of lopinavir/ritonavir in a murine model of malaria and provided additional supporting data that inhibition of plasmepsins by protease inhibitors is the mechanism of action of PIs against *P. falciparum*.^136^ Redmond evaluated serum from HIV-infected persons receiving PIs, and demonstrated that *P. falciparum* was inhibited by serum from patients receiving PIs, but not non-nucleoside reverse transcriptase inhibitors (NNRTIs).^135^ Thus, in vitro assays, animal models, and human sera experiments all indicate that HIV PIs inhibit *P. falciparum* at levels achievable in HIV-infected persons and support their evaluation as an innovative preventive measure against malaria among HIV-infected individuals.

Protease inhibitors can be safely used in pregnant women**.** PI-based ART regimens are considered first line regimen based on their antiviral potency, and they are generally well-tolerated by pregnant women. A UK-based retrospective study evaluated 104 pregnant women receiving LPV/r and found that despite 26% of women experiencing nausea/vomiting, 19.2% Grade 1-2 hepatic toxicity, and 3.8% Grade 3 hepatic toxicity, 92% of women continued the regimen until delivery.^139^ As such, PI-based ART has become standard for pregnant women in the resource-rich setting, and it is now being increasingly evaluated for use during pregnancy in the resource-limited setting.^140 141^ The WHO recently updated its recommendations related to antiretroviral use in pregnancy and added LPV/rit as a 1^st^ line option for women with CD4 count above 350.^142^

There are safety issues related to the use of PIs in pregnant women and their exposed infants that merit comment: 1) preterm delivery (PTD), 2) low birth weight (LBW), 3) gestational diabetes and 4) pre-eclampsia.^143^ While some cohort studies have demonstrated an association between ART and PTD,^144-149^ this has not been corroborated in other large multi-center studies.^143 150-152^ A retrospective analysis of HIV-exposed infants followed as part of the Pediatric Spectrum of HIV Disease cohort found a significant decline in the prevalence of preterm delivery corresponding to an increase in antiretroviral use, including combination regimens, among pregnant women in the US.^153^ The most definitive review of this topic is a recent meta-analysis which found only ART initiated prior to conception or in the first trimester to be associated with preterm delivery.^154^

Similarly, the largest and most recent studies evaluating birth outcomes among HIV-infected pregnant women have concluded that HIV PIs are not a risk factor for LBW. In the WITS cohort of 2543 pregnant women, there was no association between PI-based ART and LBW.^152^ The Pediatric Spectrum of HIV Disease cohort found a significantly decreased prevalence of LBW, from 35% to 21%, with the increased use of ART from 1989 to 2004, and PI-based ART, in particular, was not associated with LBW.^153^ Lastly, the NISDI cohort involving 681 women demonstrated no association between PI use and LBW in a multivariate analysis.

Nearly all studies investigating the risk of gestational diabetes among women on PIs have not found an association.^152^ In a recent multi-center, prospective cohort study designed specifically to evaluate risk of gestational diabetes and PI use, Hitti and colleagues found no such association between PI use and glucose intolerance or insulin resistance in pregnancy.^152 155^ Thus, despite early concern for the safety of PIs in pregnant women, a large body of data supports their use in this population.

There are conflicting data evaluating the risk of pre-eclampsia among HIV -infected women and the association between ART and pre-eclampsia. A British study found an significantly increased risk of pre-eclampsia among HIV-infected women on HAART(10.5%) compared to women without any antiretroviral exposure (0%), though there was no difference in the risk of pre-eclampsia between HIV-infected versus HIV-uninfected women. ^156^ A Spanish cohort study found that HIV-positive women experienced a significantly increased risk of pre-eclampsia compared with HIV-negative women (adjusted OR, 4.9; 95% CI, 2.4-10.1). ^157^ In addition, among those with HIV, use of HAART prior to pregnancy was associated with an increased risk of pre-eclampsia (adjusted OR, 8.9; 95% CI, 1.7–45.5). On the other hand, other studies have failed to demonstrate an association between HIV and pre-eclampsia. A cross-sectional study in the US found no difference in prevalence of preeclampsia-associated hospitalizations between HIV+ and HIV-negative women. ^4^ Furthermore, there was no significant difference in the rate of pre-eclampsia between women delivering in the pre-HAART and HAART eras. A retrospective cohort in South Africa of 2600 women found no difference in the risk of pre-eclampsia between HIV+ and HIV-negative women, though there were no data on CD4 count and the proportion of HIV+ women on ART. ^158^ A study from South Africa also found no difference in the prevalence of pregnancy-induced hypertension or eclampsia between women infected or uninfected with HIV, though it was not clear from the data what proportion of HIV+ women received antiretrovirals. ^159^ Other studies have, on the contrary, found a decreased risk of pre-eclampsia or eclampsia among HIV-infected women. A Brazilian case-control study found that HIV-positive women, most of whom had viral suppression on HAART, had a significantly lower risk for pre-eclampsia (0.8%) than HIV-negative controls (10.6%).^160^ A retrospective cohort study of HIV-infected and HIV-uninfected pregnant women admitted to a high-risk obstetrical unit in South Africa found no difference in the prevalence of pre-eclampsia but a significantly lower risk of eclampsia among HIV+ (4.7%) compared to HIV-uninfected women (17.1%, p-value 0.04). ^161^

As discussed above, women – particularly those with HIV – have an on-going susceptibility to malaria during the postpartum period and thus may benefit from continued malaria preventive therapy. Our study will be able to determine whether the use of PIs reduces this risk. Our study also comes at an opportune time to contribute data to other studies focusing on optimal postpartum ART regimens for PMTCT of HIV in breastfeeding women. Uganda has adapted the WHO policy recommending that HIV-infected women exclusively breastfeed for 6 months unless living in areas where replacement feeding is acceptable, feasible, affordable, sustainable and safe.^162^

The WHO has recently updated its recommendations related to infant feeding in the setting of maternal HIV and now recommends exclusive breastfeeding for the first 6 months of life with the introduction of complementary foods at that time. ^163^ The WHO recommends continuing breastfeeding until 1 year of age. Several observational cohorts have demonstrated remarkably low postnatal MTCT in the setting of lactational ART.^164^ The DREAM cohort in Mozambique provides nevirapine (NVP) based ART to all pregnant women regardless of CD4 count and continues the ART during 6 months of lactation. Preliminary findings include 1.2% MTCT at 1 month of age and only an additional 0.8% MTCT by 6 months of age. The MITRA PLUS cohort in Tanzania has preliminarily found a cumulative MTCT prevalence of 5% at 6 months among women receiving NVP-based ART during late pregnancy and for a minimum of 6 months postpartum.^165^ A small observational study in Rwanda similarly found a low risk of MTCT in the setting of third-trimester and lactational efavirenz. ^166^ Several trials have also found very low risk of HIV transmission in the setting of HAART use in pregnancy and during 6 months of lactation. Most notably, the Mma Bana study in Botswana was a randomized controlled trial of ZDV/3TC/abacavir vs. ZDV/3TC/LPV/rit among pregnant women with CD4 count 200 or above. ^167^ The study also followed pregnant women with CD4 counts below 200 who received ZDV/3TC/NVP. The cumulative risk of transmission through the 6 month breastfeeding period was 1.8%, 0.4% and 0.6% for infants born to women on the abacavir, LPV/rit and NVP-based regimens, respectively.

Given the on-going risk of malaria postpartum, and the increasing body of literature demonstrating the benefit of lactational antiretrovirals, the use of PIs postpartum will likely be associated with improved HIV and malaria outcomes for both women and their infants.

Given that women will be followed in this study postpartum and one arm of the study includes the potentially teratogenic medication EFV, the drug-drug interactions between hormonal contraception and antiretroviral medications warrant further discussion. In particular, when taken in conjunction with ethinyl estradiol/norethindrone acetate, EFV has been associated with an increase in ethinyl estradiol levels (area under the curve [AUC] increased by 37%) with no significant change in Cmax. ^168^ There are no apparent changes in norethindrone acetate or EFV levels and the increased levels of ethinyl estradiol are of uncertain clinical significance. On the other hand, when taken in conjunction with ethinyl estradiol/norgestimate, EFV has been associated with decreased levels of norgestimate (AUC decreased by 64%, Cmax decreased by 46%, Cmin decreased by 82%) without significant effects on the levels of ethinyl estradiol. ^169^ Lopinavir/ritonavir also appears to have interactions with both ethinyl estradiol (AUC decreased by 42%, Cmax decreased by 41%, Cmin decreased by 58%) and norethindrone acetate (AUC decreased by 17%, Cmax decreased by 16%, Cmin decreased by 32%), ^170^ These pharmacokinetic charges are thought due to ritonavir’s induction of CYP450, though, are of unclear clinical significance. As opposed to the pharmacokinetic changes seen with concurrent antiretrovirals and combined hormonal contraception, there do not appear to be significant pharmacokinetic changes in or altered clinical effectiveness of either depot medroxyprogesterone acetate (DMPA) or EFV when these 2 medications are given concurrently.^171 172^

It is rational to evaluate the strategic use of HIV protease inhibitors in resource limited settings. At present, although HIV PIs play a pivotal role in first and second line therapy in the US, they are used to a very limited extent in resource limited settings. In contrast to NNRTIs, HIV PIs require a higher pill burden, a cold chain for storage and higher cost. In addition, unlike NNRTIs, there are no co-formulated preparations, where all 3 components of an ART regimen have been packaged into a single tablet. Some of these barriers to wider PI usage in Africa have already begun to be dismantled. For example, there is now a preparation of co-formulated LPV/r that is heat stable, which we propose to evaluate in this study. Price reductions to ART have been dramatic in recent years, and may continue for PIs, particularly if they are shown to have benefit for multiple diseases. It should be duly noted that ART never would have been deployed in Africa had the market costs at the time been considered a prohibitory factor. Finally, the long term consequences of drug resistance remain a concern for HIV strategies, and PIs offer the advantage of a high genetic barrier to HIV drug resistance. For settings where therapy interruptions are anticipated or planned, such as pregnancy among immunocompetent women, the shorter pharmacologic half-life of PIs could be viewed as advantageous.

**Antiretroviral exposure in pregnancy and in infants**

A secondary objective of this study is to examine PI and NNRTI exposure in HIV-infected women and infants during important transitions and to assess if exposure correlates with certain key outcomes and toxicities of PMTCT. In HIV-infected women, we will compare LPV/r and EFV levels in participants on PI- versus non-PI based ART, respectively, during the third trimester and after delivery to assess if exposure differs significantly in those two periods. Original pharmacokinetic studies of the previously-available LPV/r capsules (dosed at 3 capsules twice daily) during the third trimester indicated that LPV levels during the final trimester were significantly lower than during the postpartum period and in nonpregnant adults^173^, but pharmacokinetic studies using the new tablet formulation of LPV/r (dosed at 2 tablets twice daily) during pregnancy are still underway.^174^ Data on whether EFV exposure is altered during the third trimester of pregnancy are not yet available. Data from this study would provide LPV/r or EFV exposure measurements in HIV-infected pregnant women during the third trimester and compare those levels to those obtained during the postpartum period. Exposure will be assessed by measuring single plasma levels of the ARV of interest, as well as levels of the drug in small hair samples from the women.

In infants, the degree of *in utero* exposure to PIs or NNRTIs has not been systematically evaluated up to this point since a long-term assessment of exposure has not yet been available. Measuring ART levels in hair provides information on an average level of exposure to medications over weeks to months (see below) and hair levels of medications in infants at delivery will likely reflect exposure during the third trimester since fetal hair begins to grow only in the 3^rd^ trimester. We will assess hair levels of LPV/r or EFV in infants born to HIV-infected women in our study on PI or non-PI based ART, respectively.

The exposure of breastfeeding infants to PIs or NNRTIs administered to their mothers during lactation has also not been systematically evaluated, although preliminary studies suggest that levels of these agents in maternal plasma are not correlated with plasma levels in infants.^166^ Since the infants in our study will potentially be exposed to PI- or non-PI based ART for the duration of breastfeeding, we will measure plasma and hair levels of LPV/r and EFV in the infants at 12 and 24 weeks of life in order to assess their level of exposure to these agents through breastfeeding.

**Hair levels to assess ARV exposure**

The concentration of medications in hair reflects drug uptake from the systemic circulation over weeks to months. Therefore, drug levels in hair provide an advantage over single plasma drug concentrations in estimating an average level of medication exposure.^175^

Although commonly used as surrogates for antiretroviral exposure, adherence measures are imperfect indicators of the amount of drug that eventually reaches the site of viral activity. Not only are methods of evaluating adherence limited by patient recollection and accuracy^176^, but adherence measurements do not account for interindividual biologic variability in the amount of drug that is absorbed and eliminated.^177-179^ Measurement of plasma drug levels as a method of therapeutic drug monitoring (TDM) has had inconsistent success in predicting treatment outcomes^180-183^, probably because drug levels vary significantly from day to day within an individual.^184^ Other limitations of TDM using blood levels include “white-coat” effects^185^, in which adherence just prior to medical appointments improves above average, and its dependence on the patient’s ability to accurately report the timing of recent doses.

Many drugs are incorporated into hair as it grows, but use of hair specimen testing has largely been limited to forensic applications.^186^ Recently, the potential utility of hair specimen assays in the assessment of exposure to chronically administered medications has been reported^175 187 188^, including testing for indinavir^189-192^, LPV/r, atazanavir, efavirenz and nevirapine. ^193^ A collaborating group from the Women’s Interagency HIV Study (WIHS) showed that hair concentrations of protease inhibitors were the strongest independent predictor of virologic success in women initiating LPV/r or atazanavir-based therapy.^194 195^ A similar study showed EFV hair levels to be the strongest predictor of virologic success in a cohort of treatment-experienced women initiating EFV-based HAART ^196^ Given the advantage of hair monitoring for ARV levels in assessing long-term exposure to ART agents and the recent availability of laboratory methods to measure these agents in hair, we will use both plasma and hair levels of LPV/r and EFV to assess exposure in mothers and infants in our study.

**1.2 Rationale**

This study is designed to compare a PI containing antiretroviral regimen

(zidovudine, lamivudine, and lopinavir/ritonavir) to a non-PI regimen

(zidovudine, lamivudine, and efavirenz), with the goal of reducing the morbidity of HIV and malaria among women at all CD4 counts and their children.

Although the provision of ART to all HIV-infected pregnant and postpartum

women regardless of CD4 count differs from the current Uganda Ministry of

Health (MOH) standard of short-course antepartum zidovudine plus single-dose

nevirapine in labor to women who do not otherwise qualify for ART, the Uganda

MOH supports our study design. The proposed study to improve outcomes in

HIV-infected pregnant women and their children is designed in collaboration with

Ugandan health care authorities, clinicians, and public health officials. Our study

supports the Uganda MOH priorities to address HIV, malaria and maternal child health. Of importance to public health officials is that we are testing our hypothesis and conducting our trial in rural Uganda in a high malaria transmission

area where the results would ultimately be applied. We are testing our

intervention in the context of Uganda MOH and WHO guidelines, which recommend daily TS, ITNs, and exclusive breastfeeding for the first 6 months postpartum for HIV-infected pregnant and postpartum women.

Zidovudine and lamivudine are common to both arms of the study and are

nucleoside analogs that are widely used in pregnancy and are well-tolerated and safe for both pregnant women and their exposed infants. Because the protocol includes women of all CD4 counts, efavirenz is the non-PI of choice given its potency, minimal known risk when given after the first trimester in pregnancy and listing as an alternative medication for pregnant women in the Ugandan national HIV treatment guidelines.

Efavirenz will be the NNRTI used for this study. Efavirenz-based antiretroviral regimens are considered a 1^st^ line option for non-pregnant adults in many countries in sub-Saharan Africa, including Uganda. In addition, efavirenz is listed as an alternative regimen in pregnancy after the 1^st^ trimester according to the Ugandan Ministry of Health. The recently updated WHO antiretroviral guidelines recommend EFV as a 1^st^-line option in pregnancy for women at all CD4 counts.^142^ Similarly, the US Perinatal HIV Treatment Guidelines state that the use of efavirenz “can be considered if, after consideration of other alternatives, this is the best choice for a specific woman.” We have specifically chosen efavirenz as the NNRTI of choice in this study, because our goal is to enroll women at all CD4 counts and there is a well documented risk of severe hepatotoxicity with nevirapine (the other NNRTI available) among HIV-infected adults with CD4 counts above 250. While there are animal and human data linking first-trimester use of efavirenz and neural tube defects in exposed offspring, there is no evidence of harm among pregnant or breastfeeding women, fetuses or infants exposed to efavirenz during the second/third trimester or breastfeeding period.

Rationale for the study is summarized below:

- Pregnant and postpartum women with HIV are at increased risk of malaria.
- Malaria is associated with maternal and obstetric morbidity and mortality and poor infant outcomes, all of which are amplified in the setting of HIV.
- Malaria burden is substantial even with current prophylactic interventions; resistance to antifolates is increasing, jeopardizing preventive interventions with TS.
- We need additional tools to decrease risk of malaria in pregnant and postpartum women.
- HIV protease inhibitors are active in vitro against *P. falciparum.*
- PI-based ART is effective, safe and well-tolerated during pregnancy.
- HIV PIs may thus prove to have simultaneous protective effect against HIV and malaria.
- Strategic use of HIV PIs can improve maternal and fetal outcomes among HIV populations living in malaria endemic areas.
- As logistical barriers to the use of PIs decrease in sub-Saharan Africa, additional costs related to PI use may be off-set by the preventive benefit of PIs against malaria and perinatally-transmitted HIV, and costs may be limited by targeting PI use to the high risk periods of pregnancy and breastfeeding.

**1.3 Antiretroviral Therapy**

This study will compare two different ART regimens. Pregnant women enrolled

in the study will start either an ART regimen containing the HIV protease inhibitor lopinavir/ritonavir +2 NRTIs or a non-PI containing ART regimen (EFV+2 NRTIs). A summary of the current ART access situation in Uganda and a brief synopsis of the ART drugs women will receive while participating in the study are provided below.

**1.3.1 ART Programs in Uganda**

Uganda has been at the forefront of providing access of ART to HIV infected adults and children in Africa. Over the past five years, the Ministry of Health (MOH) in collaboration with the World Health Organisation (WHO), World Bank Multi-country AIDS Program (MAP), Global Fund to Fight AIDS, Tuberculosis and Malaria (GFATM), the United States President's Emergency Plan for AIDS Relief (PEPFAR) and other partners have supported expansion of HIV-treatment programs in Uganda.^197^ The increasing availability of cheaper generic drug formulations has permitted expanded HIV-treatment programs in Uganda. Since the government of Uganda launched the universal access to free antiretroviral drugs in 2004, 313 public and private facilities have been accredited to provide antiretroviral therapy (ART) by the end of March 2008, and, of the 300,000 people estimated to need treatment, 135,000 are already accessing ART countrywide. Efavirenz and nevirapine are the available and recommended NNRTIs in Uganda. For first line therapy, ZDV or d4T plus 3TC are used for the NRTI backbone.

**1.3.2 ART agent: Efavirenz**

Efavirenz is an NNRTI that is active against HIV-1. It is used for treatment of HIV in children and adults. When given in combination therapy, efavirenz-based regimens are successful in achieving sustained viral suppression in adherent patients. A single point mutation in reverse transcriptase can confer high level drug resistance. There is cross resistance between efavirenz and nevirapine. Efavirenz is generally well tolerated. Many persons experience mild alterations in sensorium which resolve after 2-4 weeks of therapy. Rash has been reported in up to 10%, but is rarely severe. Efavirenz is typically dosed at bedtime to minimize central nervous system side effects. While there are animal and human data linking first-trimester use of efavirenz and neural tube defects in exposed offspring, there is no evidence of harm among pregnant or breastfeeding women, fetuses or infants exposed to efavirenz during the second/third trimester or breastfeeding period.^166 198-206^ Dosing of efavirenz for use in this study is found in section 5.0.

**1.3.3 ART agent: Lopinavir/ritonavir (Aluvia)**

The coformulation of lopinavir + ritonavir (lopinavir/ritonavir) received FDA approval in 2000 for treatment of HIV in adults and children age 6 months or older. Regimens containing lopinavir/ritonavir have been shown to be effective as initial therapy. Lopinavir/ritonavir, used with stavudine and lamivudine as initial treatment, was significantly more effective in achieving virologic suppression than the combination of nelfinavir + stavudine + lamivudine (HIV RNA <50 copies/mL in 67% vs. 52% at 48 weeks; p < .001).^207^ In a study comparing lopinavir/ritonavir with ritonavir-boosted fosamprenavir, the 2 boosted protease inhibitors, given in combination with abacavir + lamivudine, provided comparable rates of virologic suppression and similar increases in CD4 cell counts. At 48 weeks, by intent-to-treat analysis, the proportion of subjects with HIV RNA <50 copies/mL was 65% in the lopinavir/ritonavir group and 66% in the fosamprenavir + ritonavir group. CD4 count increases were 191 cells/µL and 176 cells/µL, respectively.^208^

In a randomized comparison between lopinavir/ritonavir and efavirenz, each given with 2 nucleoside analogues, lopinavir/ritonavir recipients had lower rates of virologic suppression to <50 copies/mL at 48 weeks (77% for lopinavir/ritonavir and 89% for efavirenz recipients; p = .003), though greater increases in CD4 cell counts (285 and 241 cells/µL, respectively; p = .01). A third treatment group was given efavirenz + lopinavir/ritonavir, without nucleoside analogues; 83% of subjects in this group had virologic suppression to <50 copies/mL.^209^ The most common symptomatic side effects of lopinavir/ritonavir are diarrhea and nausea. Common laboratory abnormalities include elevated cholesterol and triglyceride levels; elevated liver transaminases are also observed. As with other protease inhibitors, lopinavir/ritonavir may contribute to abnormalities of body fat distribution. Lopinavir/ritonavir is considered safe in pregnancy and is the preferred PI-based regimen for pregnant women in the United States.^198 206^ Dosing of LPV/r for use in this study is found in section 5.0.

**1.3.4 ART agent: Zidovudine (ZDV)**

Zidovudine was the first agent approved for the treatment of HIV and has been used for over a decade in adults and children. ZDV is a thymidine analogue that inhibits HIV reverse transcriptase. ZDV requires intracellular phophorylation to the active triphospate form. The most common clinical symptomatic toxicities associated with zidovudine include headache, fatigue, malaise, myalgia and, nausea. The most common laboratory toxicities associated with zidovudine are anemia and neutropenia. Other less common side effects are nail pigmentation, myopathy, hepatitis, and rarely, lactic acidosis. These effects may require dose modification or substitution of another NRTI such as tenofovir or stavudine. ZDV will be administered with lamivudine as part of the NRTI backbone of a treatment regimen. ZDV is antagonistic with stavudine, and patients should not receive both drugs concomitantly. Resistance to ZDV evolves over a period of months, and requires multiple mutations in reverse transcriptase. Resistance to ZDV can reduce susceptibility to other thymidine analogues. Zidovudine is considered safe in pregnancy and a preferred NRTI for pregnant women in the United States. ^198 206^ Dosing of zidovudine for use in this study is found in section 5.0.

**1.3.5 ART agent: Stavudine (d4T)**

Stavudine may be used among women in this study who are not candidates for zidovudine because of severe anemia or other toxicity. Stavudine is a thymidine analogue, which is used as combination ART in both adults and children. In early studies of stavudine, HIV RNA levels were reduced by over 0.5 log in treatment naïve adults who received monotherapy.^210^ Prior zidovudine treatment was associated with a reduction in antiviral response to stavudine.^211^ One of the major toxicities associated with stavudine is peripheral neuropathy. In adults, peripheral neuropathy is a dose -related toxicity. Lactic acidosis is another side effect which is rare, but can be fatal, and has been seen among pregnant women. ^206^ Asymptomatic low level lactate elevations are common in pediatric and adult patients receiving stavudine, but these have not been shown as predictive of subsequent lactic acidosis.^212^ Body habitus changes, specifically lipoatrophy and facial wasting, have been noted in both adults and children receiving stavudine as a part of combination therapy regimens.^213^ These changes occur over time, and studies in adults demonstrate that regimens using tenofovir produce less lipoatrophy changes than regimens using stavudine. In adult studies, lipatrophy also appears more severe with stavudine as compared to zidovudine. Substitution of another agent for stavudine may prevent progression of lipoatrophy among those with this side effect, but reversibility may require years or may not occur. Dosing of stavudine for use in this study is found in section 5.0.

**1.3.6 ART agent: Lamivudine (3TC)**

Lamivudine is a cytidine analogue that inhibits reverse transcriptase and is active against HIV-1 and HIV-2. It is also active against hepatitis B virus. It is widely used in combination ART for HIV treatment in adults and children. A single point mutation at codon 184 confers resistance to 3TC and can be selected rapidly when viral suppression is not achieved. Lamivudine is extremely well tolerated. Rarely, it has been associated with pancreatitis and peripheral neuropathy. It is difficult to attribute the other toxicities reported to occur with lamivudine such as lactic acidosis, headache, fatigue, and gastrointestinal symptoms, because these have occurred in the setting of other NRTI agents that are known to produce these toxicities. Lamivudine is considered safe in pregnancy and a preferred NRTI for pregnant women in the United States.^198 206^ Dosing of lamivudine for use in this study is found in section 5.0.

**1.3.7 ART agent: Tenofovir (TDF)**

Tenofovir may be used among women in this study who are not candidates for zidovudine because of severe anemia or other toxicity. Tenofovir is an adenosine analogue that inhibits reverse transcriptase and is active against HIV as well as hepatitis B virus. It is widely used in combination ART for HIV treatment in adults. Tenofovir is extremely well tolerated. Rarely, tenofovir has been associated with lactic acidosis and severe hepatomegaly with steatosis. Tenofovir may also be associated with decreased bone mineral density or renal insufficiency. In addition, tenofovir may be associated with severe acute exacerbation of hepatitis B virus, particularly if an individual has active viral hepatitis B infection and subsequently stops taking TDF. Tenofovir is considered safe in pregnancy. Dosing of tenofovir for use in this study is found in section 5.0.

**1.3.8 ART agent: Emtricitabine (FTC)**

Emtricitabine may be used among women in this study who are prescribed tenofovir because of zidovudine-associated toxicity. Emtricitabine is very similar to lamivudine and may be used in this study as a co-formulated once-daily tablet with tenofovir as dictated by pharmacy supplies and as clinically indicated. Emtricitabine is a cytidine analogue that inhibits reverse transcriptase and is active against HIV as well as hepatitis B virus. It is widely used in combination ART for HIV treatment in adults. Emtricitabine is extremely well tolerated. Rarely, emtricitabine has been associated with lactic acidosis and severe hepatomegaly with steatosis. In addition, emtricitabine may be associated with severe acute exacerbation of hepatitis B virus, particularly if an individual has active viral hepatitis B infection and subsequently stops taking FTC. Emtricitabine is considered safe in pregnancy. Dosing of emtricitabine for use in this study is found in section 5.0.

**2.0 STUDY OBJECTIVES**

**2.1 Primary Objective**

To determine if the use of PI-based ART versus non-PI ART among HIV-infected pregnant women leads to reduced rate of placental malaria as measured by Giemsa-stained smear or PCR for malaria parasites of placental blood.

**2.2 Secondary Objectives**

1) To determine if the use of PI-based ART versus non-PI ART among HIV-infected pregnant women leads to reduced rate of placental malaria as measured by alternate diagnostic modalities (rapid diagnostic test (RDT; immunochromatographic lateral flow for histidine rich protein 2 and plasmodium lactate dehydrogenase [pLDH])) for malaria parasites in placental blood; placental histopathology).

2) To determine if the use of PI-based ART versus non-PI ART among HIV-infected pregnant women leads to reduced rate of maternal malaria and severe anemia.

3) To determine if the use of PI-based ART versus non-PI ART among HIV-infected pregnant women leads to reduced morbidity measured by a composite clinical outcome of infant low birth weight, still birth (intrauterine fetal demise > 20 weeks gestation), late spontaneous abortion (miscarriage 12-20 weeks gestation), preterm delivery (<37 weeks gestation), and neonatal death (death of a liveborn infant within first 28 days of life).

4) To compare the maternal and infant safety and tolerance profile of

PI-based versus non-PI ART during pregnancy and breastfeeding

5) To compare the incidence of preeclampsia between PI-based versus non-PI ART among HIV-infected pregnant women.

6) To compare the virologic and immunologic efficacy of PI-based versus non-PI ART in pregnant and breastfeeding women, including:

i) Prevalence of maternal HIV RNA suppression of <400 copies/mL and of <50 copies/mL at delivery, 24 weeks after the start of the treatment regimen.

ii) Change in maternal CD4 cell counts and % CD4 from ART initiation to delivery and from delivery to the cessation of breastfeeding.

iii) Development of one or more new maternal HIV antiretroviral resistance mutations measured at delivery, 24 weeks and 1 year postpartum.

iv) Incidence of maternal to child transmission of HIV, measured by infant HIV DNA PCR at delivery, DNA PCR at follow-up visits and 58 weeks of life or 6 weeks following the cessation of breastfeeding if that occurs prior to 1 year of life.

7) To assess PI-based and non-PI based ART exposure in HIV-infected women and their infants by measuring ART levels in plasma and hair samples (women at 30-34 weeks gestation and 12 weeks postpartum; infants at delivery, 12 weeks and 24 weeks of life).

8) To assess the association between nutritional status and obstetrical, infant and

HIV-related outcomes, including ART levels among women.

**3.0 STUDY DESIGN**

This study is an open-label, single site, randomized controlled trial comparing PI-based ART to non-PI based ART for HIV-infected pregnant and breastfeeding women of all CD4 cell counts at high risk of malaria. The study is designed to test the hypothesis that pregnant women receiving a PI-based ART regimen will have lower risk of placental malaria compared to pregnant women receiving a non-PI based ART regimen. The primary study endpoint of the study is placental malaria.

The study site will be the Tororo district hospital campus situated in Eastern Uganda, an area of high malaria transmission. Using convenience sampling, we will enroll 500 HIV-infected pregnant women from the Tororo community. Eligible women between 12-28 weeks gestation will be randomized at enrollment to receive either a PI- based or an NNRTI-based ART regimen (Table 4) after stratification by gravidity (G1 versus G2+) and gestational age (<24 weeks versus ≥ 24 weeks at enrollment).

Table 4. Treatment arms

| Treatment Arm | Medications* |
| --- | --- |
| Group A | ZDV 300mg/3TC 150mg/ LPV 200mg/r 50mg |
| Group B | ZDV 300mg/3TC 150mg/ EFV 600mg |

*ZDV = zidovudine, 3TC = lamivudine, EFV = efavirenz, LPV = lopinavir, r = ritonavir

At enrollment, all study participants will receive a long lasting ITN and, as available, a basic care package including a safe water vessel, multivitamins and condoms, as per current standard of care for HIV-infected pregnant women in Uganda, if they have not already received these interventions from the referral site. Two ITNs will be provided for each mother-infant pair. Participants will receive all routine and acute medical care at a designated study clinic open 7 days a week from 8 a.m. to 5 p.m. If medical care is needed after hours, participants will be instructed to come to Tororo District Hospital premises (where the study clinic is located) and request that the study physician on-call be contacted. They will be followed up from the time of enrollment during pregnancy and through the cessation of breastfeeding; seen monthly for routine assessments and laboratory evaluations. Following delivery, the infants of enrolled women will be followed until 6 weeks following the cessation of breastfeeding but not beyond 58 weeks of life. Study participants will be followed closely for adverse events potentially due to study drugs and for malaria and HIV treatment outcomes. During the follow-up period, all patients presenting to the clinic with a new episode of fever will undergo standard evaluation (history, physical examination and Giemsa-stained blood smear) for the diagnosis of malaria.

**Women**

Women will receive the study treatment from the time of study entry and randomization (12-28 weeks gestation) until 1 week following the cessation of breastfeeding (but no longer than 1 year + 1 week postpartum).

If a subject experiences a toxicity endpoint, ART will be changed to provide antiviral activity prior to delivery.

Exclusive breastfeeding will be encouraged until 24 weeks postpartum which is the standard of care in Uganda. As per updated WHO guidelines, women will be encouraged to introduce food at 6 months of life and continue breastfeeding until 1 year of life. Women will be counseled to wean over the course of 1 month and continue antiretrovirals for at least 1 week following weaning. Furthermore, if an infant is found to be HIV-infected, Uganda MOH and WHO guidelines recommend the continuation of breastfeeding until 2 years of life and daily TS.

All women will receive daily oral trimethoprim/sulfamethoxazole (TS) per Ugandan MOH guidelines. The schedule for clinical and laboratory evaluation for women is outlined in Appendix I.

**Infants**

Per Ugandan MOH guidelines, all newborns will receive nevirapine syrup (10mg/ml) starting within 12 hours after birth for 6 weeks, daily oral TS from 6 weeks of life until 6 weeks following the cessation of breastfeeding, and their mothers will be instructed on ITN use for their infants.

The schedule for clinical and laboratory evaluation for infants is outlined in Appendix II.

**4.0 SELECTION AND ENROLLMENT OF SUBJECTS**

**4.1 Inclusion criteria**

1. Age > 16 years (if <18 years old, living independently from parents)
2. Documentation of HIV status must come from two assays. Assays include DNA PCR, HIV RNA, Western blot, or rapid HIV antibody test
3. Confirmed pregnancy by positive serum or urine pregnancy test or ultrasound
4. Estimated gestational age between 12 and 28 weeks (based on first day of last menstrual period with physical exam confirmation and ultrasound confirmation) at time of enrollment
5. Residency within 30 km of the study site
6. Willing to provide informed consent

**4.2 Exclusion criteria**

1. Current or prior use of HAART
2. Exposure to single-dose NVP (alone or with zidovudine or zidovudine/lamivudine or other abbreviated monotherapy or dual therapy for PMTCT) less than 24 months prior to enrollment
3. Prior dose-limited toxicity to TS within 14 days of study enrollment
4. Receipt of any contraindicated medications within 14 days of study enrollment (See Appendix III.)
5. Active tuberculosis or other WHO Stage 4 diseases
6. Screening laboratory values:
   1. Hemoglobin: <7.5 g/dL*
   2. Absolute neutrophil count (ANC): <750/mm^3^
   3. Platelet count: <50,000/mm^3^
   4. ALT: >225 U/L (>5.0x ULN)
   5. AST: >225 U/L (>5.0x ULN)
   6. Bilirubin (total): > 2.5x ULN
   7. Creatinine: > 1.8x ULN
7. Known cardiac conduction abnormalities or structural heart defect

* Women found to have a hemoglobin <7.5 at screening may receive iron and folic acid and/or a blood transfusion at the physician’s discretion. If a repeat hemoglobin is ≥7.5 g/dL, the woman may be considered for study inclusion.

NOTE: A woman will be excluded from study participation during the current pregnancy if she goes into labor, experiences ruptured membranes or develops active tuberculosis or a WHO stage 4 condition following study enrollment but prior to study drug initiation.

**4.3 Identification and recruitment of study participants**

Participants may be recruited through multiple referral mechanisms including those described below:

Tororo District

- Tororo District Antenatal Clinics: HIV testing is offered to all pregnant women presenting to the antenatal clinics of Tororo district. We will approach women known to be HIV-infected or identified as HIV-infected through voluntary counseling and testing.
- HIV Testing Service: Tororo District Hospital also offers HIV testing services to individuals referred from outpatient clinics or other health facilities in the community.  Participants will also be recruited from these testing units.

The AIDS Service Organization (TASO)

TASO has been providing care to HIV infected children and adults in Tororo since 1989. Pregnant women aged 16 years or older will be referred to the study clinic to be screened for eligibility.

Tororo community household testing

Two ongoing studies conducted by the CDC in Tororo are actively involved in household testing; these include the Home-Based AIDS Care Project (HBAC) and the Pre Exposure Prophylaxis Project (PrEP).  In these studies, all household members are counseled and tested for HIV. Trial staff will refer HIV-infected pregnant women to our study for screening.

HIV-infected pregnant women will be approached about participating in the study and will be provided an information sheet about the requirements of the study (Appendix IV). If the woman is initially agreeable to screening for participation in the study, she will either be escorted to the study clinic or will be given an appointment to return at a later date.

Public announcements

General public announcements will be made both in local markets and over the radio (see appendix XIII). General information about the study will be given and information sheets (Appendix IV) made available. Interested women will be referred to our study clinic at Tororo District Hospital for screening.

**4.4 Screening of potential study participants**

Pregnant women referred to the study clinic will be screened for study eligibility. All interviews will be conducted in the appropriate language; translators will be used if necessary. During the screening process, the study physicians will conduct a medical history and assess for eligibility criteria of the pregnant woman (including age, last menstrual period, residency within 30 km of the study site, evidence of labor or ruptured membranes, prior continuous ART use, prior NVP exposure, history of TS toxicity, use of contraindicated medication, evidence of active tuberculosis or other WHO stage 4 disease). Eligible participants will undergo a dating obstetrical ultrasound. Study staff will use crown-rump length for gestational ages < 12 weeks and combined biometrics (biparietal diameter, head circumference, abdominal circumference and femur length) for pregnancies > 12 weeks. Gestational age will be estimated accounting for both LMP and ultrasound dating (see Appendix V).^214^

If the initial screening criteria are met, the woman will be asked to sign the screening consent form (see Appendix VI). If the woman is unable to read or write, her fingerprint will substitute for a signature, and a signature from a witness to the informed consent procedures will be obtained. The screening consent will permit obtaining blood for a rapid HIV antibody testing and other pre-enrollment labs (CBC, AST, ALT, creatinine, bilirubin, pregnancy test). A urine or serum pregnancy test may be optional if an ultrasound done the day of screening demonstrates an intrauterine pregnancy. Blood from those women found to have a positive rapid HIV antibody test will also be tested for HIV RNA and CD4/CD8 count. Laboratory and clinical assessments are shown in Appendix I. Women who pass initial screening will be asked to return to the clinic 3 to 7 days later to review the results of their screening laboratory tests. Upon completion of the screening process, those women who have passed the initial screening may have the location of their homes recorded.

If the woman is undecided about consenting to screen for the study at the initial visit, she will be allowed to return at a later visit to make a final decision about screening for study participation, as long as she will be between 12 and 28 weeks gestation at the time of enrollment. If the screening interview determines the woman is not eligible for the study, she will be referred to a local provider for care.

**4.5 Study informed consent**

Pregnant women who pass initial screening will be asked to return to the clinic 3 to 7 days later to review the results of their screening laboratory tests. If all eligibility criteria have been met, study staff will conduct the informed consent discussion in the study clinic with the subject. Informed consent for research participation will be conducted in the appropriate language and a translator will be used if necessary. The study will be described and consent obtained in one of 5 languages (Jopadhola, Teso, Swahili, Luganda, or English). The consent forms will be translated into each language and back-translated into English to check for any loss or change of meaning. The consent form will cover study participation of both the subject and her future infant, and will be read aloud to the study subject by a trained study staff member. This consent will include a discussion of the voluntary nature of research, the specific goals of the study, the nature of randomization, the study procedures including the receipt of ART, the potential risks and benefits of study participation, the importance of follow-up at our study site for all febrile episodes and scheduled study visits, placental collection and the recommendation for delivery at Tororo District Hospital. Following the informed consent discussion, women will be asked by the study physicians to sign a written consent form (Appendix VII). In addition, study physicians will conduct informed consent discussion and written informed consent for future use of biological specimens (Appendix VIII) conducted in the appropriate language; translators will be used if necessary. Women may be enrolled into the study even if she declines consent for future use of biological specimens for her and her child.

**4.6 Enrollment and Baseline Evaluation**

Women who fulfill eligibility criteria and who provide informed consent will be assigned a study number in ascending order as enrolled into the study. Women will receive a long lasting ITN and, as available, a basic care package including a safe water vessel, multivitamins and condoms, and daily TS will be initiated if they have not already received these as part of routine prenatal care. Baseline evaluations will be conducted (see Appendix I). Counseling in line with the Ugandan Ministry of Health and WHO guidelines, will be done with women regarding HIV-status, breastfeeding practices, ITN use, and TS and ART adherence. Women will be registered in the Antiretroviral Pregnancy Registry.

Women who are not eligible for the study will be referred to the appropriate local care facility. Pregnant women with life-threatening screening laboratory values will be referred to the Tororo District Hospital Antenatal Ward in-patient unit for further medical evaluation.

After the informed consent is given and all eligibility criteria are met, the study team will facilitate study treatment initiation at this visit with the goal of initiating ART among study participants as early as possible, while being mindful of safety. Women will be randomized (section 5.1) to a study treatment group and start HIV and ART counseling at this visit. The day study treatment begins will start the routine assessment schedule (section 6.0). This may be the day of enrollment if counselors agree it is appropriate. Counseling may take up to one week.

On the day of enrollment women will undergo a series of evaluations (see Appendix I) including a history and complete physical examination. Women will have blood collected by venipuncture for routine baseline laboratory testing if the screening visit laboratory testing was performed more than 2 weeks prior to the enrollment visit. Additional labs will also be drawn at the day of study treatment initiation, including fasting metabolic panel and repeat HIV RNA-PCR. A home visitor will escort the study participant home, at the enrollment visit or a later visit at a time most convenient to the participant, to record where the household is located both by hand on a Household Locator form and by GPS.

**5.0 STUDY TREATMENT**

**5.1 Treatment Group Assignments**

Study participants will be randomized to one of the two treatment groups using a 1:1 scheme. Study participants will be randomized to begin therapy when they reach at least 12 weeks gestation but no later than 28 weeks gestation. Treatment group assignment will be made according to 4 randomization lists stratified by the woman’s gravidity (G1 vs. G2^+^) and gestational age (<24 weeks vs. ≥ 24 weeks). Randomization lists will be computer generated by a member of the project who will not be directly involved in the conduct of the study. The randomization lists will include consecutive treatment numbers with corresponding random treatment assignments. Randomized codes will correspond to the 2 treatment groups using permuted variable sized blocks of 2 and 4. Sealed copies of the original randomization lists and documentation of the procedure used to generate the lists will be stored in the project administrative offices in San Francisco and Kampala. Prior to the onset of the study, two sets of sequentially numbered, opaque, sealed envelopes will be prepared. Each envelope will be marked on the outside with the treatment group and gestational age stratum (G1 vs. G2^+^, <24 weeks vs. ≥ 24 weeks) and treatment allocation number. The inside of the envelope will contain a piece of paper with the treatment allocation number and treatment group assignment along with a piece of carbon paper.

**5.2 Treatment Allocation**

Study participants will be referred to a study nurse responsible for the allocation of study drug treatment. The study nurse will assign intervention groups as follows:

1. Select next available envelope
2. Note the treatment group stratum and treatment number on the outside of the envelope
3. Write date, time, and study number on the outside of the envelope
4. Open envelope
5. Remove form containing code for treatment group and date, time, and study number (transferred to form via carbon paper inside of envelope)
6. Store form in file box in study clinic
7. Record onto the treatment allocation master list the study number, enrollment date, treatment assignment code, and study medications to be given.
8. Record the assigned treatment in the patient’s file.

**5.3 Study Drug Dosing and Formulations**

**5.3.1 Maternal Drug Administration and Duration**

Maternal treatment regimen from study randomization until 1 year postpartum, or until 1 week following the cessation of breastfeeding if < 1 year postpartum.

**Group A:**

Zidovudine 300mg PO BID

Lamivudine 150mg PO BID

Lopinavir 200mg/Ritonavir 50mg 2 tablets PO BID, increased to 3 tablets PO BID starting at 30 weeks gestation. Postpartum dosing will be 2 tablets BID.

**Group B:**

Zidovudine 300mg PO BID

Lamivudine 150mg PO BID

Efavirenz 600mg PO once daily

**Table 5. Study drug formulations**

| **Drug** | **Formulation** | **Dosing** | **Storage** |
| --- | --- | --- | --- |
| Efavirenz (EFV) | 600 mg tablets | 1 tablet orally once per day | Store at 25 C (77 F). Excursions permitted between 15 C to 30 C (59 F to 86 F) |
| Emtricitabine | 200 mg tablets | 1 tablet orally once per day | Store at 25 C (77 F); excursions permitted to 15 C–30 C (59 F–86 F) |
| Lamivudine (3TC) | 150 mg tablets | 1 tablet orally twice per day | Store at 25 C (77 F). Excursions permitted between 15 C and 30 C (59 F and 86 F) |
| Lopinavir/ritonavir (LPV/r) | 200 mg/50 mg tablets | 2 tablets orally twice per day prior to 30 weeks gestation and postpartum  3 tablets twice per day (30 weeks gestation until delivery) | Store at 20 C to 25 C (68 F to 77 F); excursions permitted to 15 C to 30 C (59 F to 86 F). Exposure of tablets to high humidity outside the original container for longer than 2 weeks is not recommended |
| Stavudine (d4T) | 30 mg tablets | 1 tablet orally twice per day | Store in tightly closed containers at 25 C (77 F). Excursions permitted between 15 C to 30 C (59 F to 86 F) |
| Tenofovir (TDF) | 300 mg tablets | 1 tablet orally once per day | Store at 25 C away from light and moisture. Excursions permitted between 15 C and 30 C) |
| Emtricitabine (FTC)/Tenofovir (TDF) co-formulation | 200mg/300mg co-formulated tablets | 1 tablet orally once per day | Store at 25 C (77 F), excursions permitted to 15–30 C (59–86 F) |
| Zidovudine (ZDV) | 300 mg tablets | 1 tablet orally twice per day | Store between 15 C and 25 C (59 F and 77 F) |
| Zidovudine (ZDV)/Lamivudine (3TC) co-formulation | 300 mg/150 mg co-formulated tablets | 1 tablet orally twice per day | Store between 2 C and 30 C (36 F to 86 F). |

Emtricitabine 200mg can be taken orally and may be taken with or without food. There are no dietary requirements or restrictions. Emtricitabine is taken once a day. A co-formulation of FTC/TDF (200mg /300mg) as a single tablet once a day may be prescribed as an alternative to Zidovudine/Lamivudine for women with anemia (Hg < 8g/dL) or other dose limiting toxicity to zidovudine (or stavudine) per DAIDS and/or Ugandan MOH guidelines.

Efavirenz 600mg can be taken orally and may be taken with or without food. There are no dietary requirements or restrictions. Efavirenz will be taken once a day, preferably just prior to sleep.

Lamivudine 150mg can be taken orally and may be taken with or without food. There are no dietary requirements or restrictions. Lamivudine will be taken BID

Lopinavir/ritonavir is co-formulated and each tablet contains 200mg lopinavir and 50mg of ritonavir. The lopinavir/ritonavir will be taken as 2 tablets BID with food until 30 weeks gestation. The dose will be increased to 3 tablets BID at 30 weeks gestation until delivery. Immediately postpartum, the dose will be decreased back to 2 tablets BID until the completion of the study.

Stavudine may be used as an alternative for women with anemia (Hg < 8g/dL) or other dose limiting toxicity to zidovudine per DAIDS and/or Ugandan MOH guidelines. The dose of stavudine is 30mg PO BID, regardless of weight and may be taken with or without food.

Tenofovir may be used as an alternative for women with anemia (Hg < 8g/dL) or other dose limiting toxicity to zidovudine per DAIDS and/or Ugandan MOH guidelines. The dose of tenofovir is 300mg once daily and may be taken with or without food. As noted above, tenofovir may be prescribed as a fixed-dose co-formulation with emtricitabine (200mg/300mg). This once-daily fixed-dose co-formulation can be taken orally and may be taken with or without food.

Zidovudine 300 mg can be taken orally and may be taken with or without food. There are no dietary requirements or restrictions. The zidovudine will be taken BID.

Zidovudine (ZDV)/Lamivudine (3TC) co-formulation will be administered as a fixed-dose combination when feasible. Fixed-dose ZDV/3TC (300 mg/ 150 mg) can be taken orally and may be taken with or without food. There are no dietary requirements or restrictions. Fixed-dose ZDV/3TC will be taken BID.

- - 1. **5.3.2 Infant Drug Regimens, Administration and Duration**

As per Ugandan MOH guidelines, all infants will receive HIV prophylaxis with nevirapine syrup starting after birth for 6 weeks. Additional or alternative ART may be prescribed at the discretion of the local investigator or pediatrician as needed. Infants will also receive daily TS elixir from 6 weeks of life until 6 weeks following the cessation of breastfeeding per Ugandan MOH guidelines. The elixir concentration is 40mg (trimethoprim)/ 200mg (sulfamethoxazole)/5ml suspension, and the infants will receive 2.5cc daily.

**5.4 Missed Doses of Study Drugs for both Women and Infants**

**Missed dose due to vomiting:** If the episode of vomiting occurs < 2 hours post administration, the mothers will be counseled to repeat the dose. Where the episode of vomiting occurs >2 hours later, the women will be counseled to not repeat the dose unless whole pills are observed in the vomitus. In infants, if the episode of vomiting occurs >2 hours later, women will be counseled to not repeat the dose.

**Missed dose due to other reasons:** If the dose was missed < 6 hours past the scheduled time of drug administration, women will be counseled that the dose should be taken as soon as remembered and the next dose may be delayed as needed to improve spacing of doses. However, if > 6 hours has elapsed since the scheduled time of drug administration, then women will be counseled to skip the missed dose and take the next scheduled dose at the next regularly scheduled time.

**5.5 Drug Supply, Distribution, and Pharmacy**

**5.5.1 Study drug supply**

Abbott Laboratories will supply the LPV/r for the duration of the study. Study medications being used in accordance with national policy will be provided by the Uganda Ministry of Health. The source of funding for these medications will be the Global Fund to fight AIDS, TB and malaria. Antiretroviral agents provided through PEPFAR will not be used in this study. The remainder of the ART medications not supplied by the Uganda MOH or Abbott will be purchased by the study.

**5.5.2 Study drug distribution**

At the time of ART initiation, pregnant women will be provided with a 5 week supply of medications and re-supplied at every 4 week visit throughout the trial. Antiretroviral drugs will be provided to the participants for the duration of the study period.

**5.5.3 Agent accountability**

The study pharmacist will maintain complete records of all study drugs received in the study pharmacy. Lot number and number of pills given to each participant at each visit will be recorded. Patients will be requested to return all empty drug bottles and to bring any bottles in use to the clinic at follow-up visits. A registry of all study medication, current product labels, and Certificates of Analysis, provided by suppliers will be maintained within the regulatory binder for the study. The date received, lot number, expiration date, and date used will be recorded for each of the study medications. Monthly inventory of all study medications will be conducted and a record log of investigational medications will be kept at the study clinic. All unused drugs will be returned to the Tororo District hospital drug stores after the study is completed or terminated.

**6.0 STUDY EVALUATIONS AND CLINICAL MANAGEMENT**

**6.1 Schedule of evaluations and interventions for women**

See Appendix I for detailed table of evaluations and interventions for women.

**6.1.1 Antepartum visits**

Antepartum study visits will occur every 4 weeks until delivery and include a history, targeted physical exam, assessments of medication adherence and phlebotomy for laboratory testing. In addition, there will be a study visit at 30-34 weeks gestation for all participants in order to obtain metabolic laboratory assays and paired blood and hair samples to measure ARV levels. Women will receive a supply of ART and TS at each visit, detailed instructions on how to take medications, information regarding common and serious side-effects, and comprehensive adherence counseling. Women can be seen for these visits within 7 days before or after the scheduled visit date. For women who miss a study visit appointment, study staff will contact the woman within 7 days of the missed appointment to bring to the clinic to ensure a consistent supply of ART. If a woman is not able to come to the clinic at all, the study staff will administer the history, targeted physical exam and obtain blood for laboratory evaluation at home.

**6.1.2 Delivery visit**

Addressing the primary aim of this study requires the evaluation of placental blood and tissue collected at delivery. For women who deliver at the hospital, hospital-based study staff will perform specimen collection and processing as below. For women who are unable to travel to the hospital, a study staff member will go to the participant’s home and assist in transporting the placenta to TDH in order to obtain placental specimens as described in the study’s standard operating procedures.

Study staff will document details of the delivery, including date and time of delivery, type of delivery, estimated blood loss and any maternal, obstetrical complications. In addition, staff will elicit the time of the last dose of ART prior to delivery. At the time of delivery, staff will also obtain maternal blood for HIV RNA, CBC, CD4 count, AST, ALT, and malaria testing. Women who deliver outside of the hospital will be encouraged to come to the hospital with their infant on the day of delivery for medical evaluation and collection of study specimens.

Placentas will be evaluated for placental malaria using 4 assays: a) placental blood parasitemia by Giemsa-stained blood smear, b) malaria RDTs (one HRP2, one pLDH) of placental blood, c) PCR of placental blood for malaria parasites, and d) placental histopathology. Within 30 minutes after delivery in the hospital, the placenta will be cleaned of blood clots, placed umbilical cord side down, and gently blotted with a paper towel. Placental blood samples and biopsies will then be collected as described in the study’s standard operating procedures.

**6.1.3 Postpartum visits**

Postpartum visits will occur every 2 weeks until the 8^th^ week, and then every 4 weeks until 24 weeks following delivery. After 24 weeks postpartum until 1 year postpartum, women will come to the study clinic every 4 weeks for pregnancy testing and dispensing of antiretroviral medication. Between 24 weeks and 1 year postpartum, women will have study visits every 3 months with targeting physical exams and laboratory evaluation. Women will receive a 5 weeks supply of antiretroviral medication at each of the dispensing visits every 4 weeks. Uganda MOH guidelines also recommend visits at 24 hours and 1 week postpartum, and these will be performed by clinical TDH staff, subject to current availability. Study staff will conduct a detailed history to assess for ART and TS adherence (number of missed doses since the last study visit), use of concomitant medications, adverse events, illnesses including febrile episodes or other symptoms of malaria, and breastfeeding patterns. Women can be seen for these visits within 7 days before or after the scheduled visit date. For women who miss a study visit appointment, study staff will contact the woman within 7 days of the missed appointment to bring to the clinic to ensure a consistent supply of ART. Women will also have a visit at 58 weeks postpartum. This visit will include an adherence questionnaire (for those women who are continuing on ART off-study, dispensed by a local facility) as well as an HIV RNA PCR.

All women (and their sexual partners when available and as appropriate) will undergo comprehensive contraceptive counseling. The contraceptive counseling will review the risks, benefits and side-effects of all available methods including condoms (male and female), combined oral conceptive pills (COC), progesterone only pills (POP), DMPA, postpartum tubal ligation (sterilization), hormonal implants, and intrauterine devices (IUD). The consent form will review the study’s requirement that women have a postpartum tubal ligation or use either an IUD or 2 contraceptive methods, including at least 1 non-hormonal method. While not mandated, women randomized to receive EFV will be encouraged to use the IUD given its superior efficacy. All contraceptives will be supplied to study participants by Tororo District Hospital. Given the teratogenic potential of EFV, all study participants will undergo monthly pregnancy testing starting at 8 weeks postpartum.

Evaluations and interventions for the mother are listed in detail in Appendix I.

**6.1.4 Standard Prenatal Care in Uganda**

Throughout the antepartum, intrapartum, and postpartum time periods, women will receive standard care as designated by the Uganda MOH Guidelines (Appendix IX). Standard antenatal care includes screening and treating as needed for syphilis and syndromic management of sexually transmitted infections. Routine antenatal medications also include iron, folic acid, multivitamins, and mebendazole. Each antenatal visit also includes blood pressure assessment and urine dipstick for proteinuria. HIV testing will be encouraged for participants’ partners; all partners found to be HIV-infected will be referred locally for care. In addition, hospital staff conducts on-going safer sex counseling for discordant couples. Standard delivery care for in-hospital births will include labor management by the midwifery staff and management of obstetrical complications. All postpartum women receive vitamin A supplementation (200,000 IU) immediately following delivery. Women are typically evaluated at 1 week and 6 weeks postpartum. These visits include a history and physical exam including evaluation for opportunistic infections, and follow-up on any obstetrical complications that occurred. Contraceptive counseling, nutritional assessment and infant feeding and support are provided as well.

### **6.1.5. Nutritional visits**

PROMOTE-PI participants may be asked to take part in optional activities about food and nutrition. The purpose of these activities is to inform the design and implementation of a nutritional support intervention for PROMOTE participants. We will plan to ask the participants a short list of questions about food security and home hygiene that should take less than 20 minutes (Appendix XIV). In addition, we plan to ask a subset of participants for their consent to participate with in-depth questioning. These interviews will take no more than 2 hours and will occur either in a focus group, one-on-one setting, or both, based on participants' preference and availability. We will collect information about food preferences, beliefs, practices, nutritional challenges, and opinions about potential micronutrient supplements (micronutrient sprinkles, tablets, and syrups). The focus group sessions will be digitally recorded to facilitate later transcription and translation. Participation is entirely voluntary and a separate consent form will be completed for those who chose to participate in a focus group discussion and/or in-depth interview (See Appendix XVI). The specific number of participants to be asked to participate in individual interviews and/or the focus groups will be depend on resources and participant visit scheduling.

**6.2 Schedule of evaluations and interventions for infants**

See Appendix II for detailed table of evaluations and interventions for infants.

**6.2.1 Delivery visit**

For women who deliver in the hospital, study staff will document Apgar score, birth weight with a calibrated scale, and any neonatal complications. Study staff will also obtain an infant blood sample for HIV DNA, thick smear, CBC, AST/ALT and ARV level. Study staff will also obtain a small swatch of infant hair. The infant will initiate nevirapine (NVP) prophylaxis within the first 12 hours of life 6 weeks of life per Uganda MOH guidelines. Women will be given a 6weeks supply of NVP syrup and will be taught how to administer the NVP prophylaxis properly. For homebirths, a study staff member will drive to the study subject’s home to assist with collection of the placenta. Women who deliver at home will be encouraged to come to the hospital with the infant on the day of delivery for clinical evaluation and to complete study procedures.

**6.2.2 Postpartum visits**

Postpartum visits will occur at 2, 6, 8, 12, 16, and 24 weeks of life in conjunction with maternal visits. Between 24 weeks and 1 year of life, infants will have study visits every 3 months. At the first postpartum visit, study staff will conduct a detailed history of the mother regarding NVP adherence for their newborn.Infant daily TS prophylaxis will be started 6 weeks postpartum and continue until 6 weeks following the cessation of breastfeeding per Uganda MOH guidelines. If an infant is found to be infected with HIV, staff will recommend that the infant continues TS, as per Uganda MOH guidelines. In follow-up postpartum visits after the 6^th^ week, study staff will conduct a detailed history of the mother regarding infant TS treatment adherence and ITN use. At all postpartum visits, study staff will obtain information from maternal history regarding infant adverse events. Study staff will perform targeted physical exams at every visit, and will monitor growth and development at weeks 2, 12, and 24 and 1 year of life. Infants can be seen for these visits within 7 days before or after the scheduled visit date. For infants who miss a study visit appointment, study staff will contact the mother within 7 days of the missed appointment to bring her child to the clinic.

**6.2.3 Standard Care of Newborns in Uganda**

Uganda MOH guidelines (Appendix IX) also recommend visits at 24 hours and 1 week postpartum, and TDH staff may conduct these visits as clinically indicated. These visits are not considered study visits. Newborns will receive NVP and TS per Uganda MOH guidelines. Also in keeping with Uganda MOH guidelines, immediate postpartum infant care will include polio and BCG immunization, ophthalmic tetracycline, and vitamin K. Also, women will receive extensive counseling in support of exclusive breastfeeding for 6 months, introduction of complementary food at 6 months, continuation of breastfeeding until 1 year and slow wean over the course of 1 month.^128^ ^163^ Infants will undergo immunization visits at 6, 10, 14 weeks and 6 months of life. Women with infants found to be infected with HIV will be encouraged to continue breastfeeding for as long as possible. The mothers of these HIV+ infants will be given the opportunity to enroll their child in a clinical trial comparing PI-based ART and non-PI ART in HIV-infected children. If they decline, they will be referred for infant HIV care from a local provider. All clinical records will be provided to the local provider.

**6.2.4. Study visits at 18 months and 24 months postpartum after study discontinuation**

PROMOTE-PI participants will be released back to their referring organization or provided an introduction to the organization of their choice for management after study participation ends. However, participants will be approached by study staff to consent for a substudy consisting of 2 visits at approximately 18 months and 24 months postpartum, while the PROMOTE-PIs study is still active. These 18 month and 24 month visits will be a basic assessment of both maternal and infant health since the last visit and will also assess child feeding patterns and basic developmental milestones. With consent from the women, study staff will alsoobtain blood specimens for blood smear, filter paper and stored blood/plasma from both mother and infant for future use. The informed consent for this substudy is found in Appendix XVI.

**6.3. Diagnosis and treatment of concurrent illnesses**

At the time of enrollment, women will be instructed to come to the study clinic for all medical care for themselves and their babies and to avoid the use of any outside medications. The study clinic will remain open 7 days a week from 8 a.m. to 5 p.m., including holidays and weekends.

**6.3.1 Malaria**

We will utilize a passive case detection system to diagnose clinical malaria. At each visit for a new medical problem, mothers and infants who are febrile (tympanic temperature > 38.0˚C) or report history of fever in the past 24 hours will have blood obtained urgently by finger-prick or heel stick for a thick blood smear. If the thick blood smear is positive, the patient will be diagnosed with malaria.

**6.3.1.1 Diagnosis of malaria**

All episodes of malaria will be classified as uncomplicated or complicated according to standardized WHO criteria:

**Uncomplicated malaria (all of the following)**

1) Fever (> 38.0ºC tympanic) or history of fever in the previous 24 hours

2) Positive thick blood smear

3) Absence of complicated malaria

**Complicated malaria (any of the following)**

1) Evidence of severe malaria and parasitemia

2) Danger signs present (WHO definitions) and parasitemia

3) Parasite density > 500,000/µl

**6.3.1.2 Management of malaria**

Uncomplicated malaria in pregnant women and infants > 4 months of age will be treated with artemether-lumefantrine (twice daily for 3 days), the recommended first-line treatment in Uganda. Several studies have demonstrated safety and effectiveness of AL against drug resistant *P. falciparum* infections in infants, children and adults, including pregnant women.^22 107 215-222^ For cases of treatment failure, women may receive quinine at the discretion of the treating physician. Infants with uncomplicated malaria under the age of 4 months or less than 5 kg with uncomplicated malaria will be treated with standard dose of quinine (3 times a day for 7 days). The first dose of antimalarial therapy will be given by a study nurse in clinic and directly observed. The rest of the antimalarial treatments will be given to mothers with instructions for administration at home. Any patient who vomits the first dose of antimalarial medication at the study clinic within 30 minutes of administration will be retreated with a second dose. Any patient who vomits repeatedly (> 3 times) will be recorded as having complicated malaria and referred to Tororo District Hospital for standard treatment with parenteral quinine.

Any patient (women or infants) who is diagnosed with severe malaria or danger signs will be referred to TDH for consideration for admission and treatment with parenteral quinine. Patients not admitted to TDH will receive standard doses of oral quinine through the study clinic. Pregnant women with complicated malaria or infants < 4 months of age will be treated with quinine (10 mg/kg three times a day x 7 days) in accordance with Ugandan guidelines.

On the day of malaria diagnosis, patients (mothers or infants) will undergo a standardized clinical history and physical examination and have blood samples obtained by venipuncture for thick and thin blood smear and filter paper collection and CBC and ALT tests. ECGs will be done for all women on Days 0 and 3 of malaria follow up. The clinical management team will be consulted for all cases of abnormal ECG findings. Molecular genotyping methods will be used to distinguish recrudescence (recurrent malaria due to parasite strains present at the time of the previous episode of malaria) from new infections (recurrent malaria due to newly infecting parasite strains) by methods validated in our laboratory at UCSF ^223^. Incident episodes of malaria will only be considered those due to new infections. Asymptomatic parasitemia will not be treated in accordance with country guidelines.

Of note, women will not be excluded from study enrollment based on acute malaria infection. However, the study will require that they complete full malaria treatment as described here prior to study enrollment.

**6.3.1.3 Diagnostic Testing for Malaria**

**Blood smears:** Thick and thin blood smears will be stained with 2% Giemsa and read by experienced laboratory technologists. Parasite densities will be calculated by counting the number of asexual parasites per 200 leukocytes (or per 500 leukocytes, if the count is <10 asexual parasites/200 leukocytes), assuming a leukocyte count of 8,000/μl. A blood smear will be considered negative when the examination of 100 high power fields does not reveal asexual parasites. Thin smears will be used for parasite species identification. Urgent thick smears will be read in our study clinic for initial diagnosis. All slides will be read by a second microscopist and a third reviewer will settle any discrepant readings.

**PCR**: Each time a thick blood smear is obtained; blood will also be collected onto filter paper. Samples will be collected by venipuncture or by finger prick sampling. Blood will be placed onto filter paper in approximately 25 μl aliquots per blood spot (4 blood spots per sample). The samples will be labeled with study numbers and dates, air-dried, and stored in small, sealed sample bags at ambient temperature or 4°C with desiccant. Molecular studies will include the extraction of DNA from filter paper and followed by characterization of parasite genetic polymorphisms using standard molecular procedures including PCR, DNA hybridization, and/or restriction enzyme digestion. For all repeat episodes of malaria, molecular genotyping methods, similar to those described for polymorphism analysis, will be used to distinguish recrudescent from new infections. Additional molecular studies will include analyses of polymorphisms in parasite genes for mutations that may impact on malaria infection and response to antimalarial therapy. Molecular studies will be performed only for research purposes and will have no impact on the clinical management of study patients.

**RDT**: One HRP2 and one pLDH RDT will be prepared and read within 24 hours of collection, using placental blood from the EDTA tube. At the end of the manufacturer-recommended development time for each test, the RDT result will be recorded in a log. An RDT result will be considered positive if both the control line and the test line are visible after the development time. A result will be considered negative if the control line is visible, but no test line appears. The result will be considered invalid if no control line is visible, regardless of whether or not a test line appears. To minimize observational bias, study team staff reading blood smears will be blinded to the RDT results. Each RDT will be read by two study staff members, and a third reviewer will settle any discrepant readings.

**Histology**: Biopsies from each placenta will be done by a laboratory technician or TDH labor ward nurse. Biopsies will be fixed then embedded in paraffin wax, sectioned into 3 μM slices using a rotary microtome, fixed to glass slides, and dehydrated in sequential ethanol baths. Separate slides will then be stained in 0.1% hematoxylin and 1% eosin and/or in 2% Giemsa as appropriate. For assessment of histologic evidence of placental malaria, pathologists who are blinded to all clinical information will assess placentas using a systematic method of analysis. Placentas will be graded into 5 categories, as described in Table 6. The presence of intervillous parasite-infected erythrocytes and of pigment in monocyte/macrophages or fibrin will be noted. Percentages of intervillous erythrocytes infected with parasites and of monocyte/macrophages containing malarial pigment will be counted. Placental specimens will be examined by two experts, and any discrepant readings will be resolved by a third reader or consensus reached by two expert readers. If histology varies in different portions of a placenta, the reading with the highest intensity of malaria infection will be used.

**Table 6. Categories of placental malaria**

| **Category** | **Histology** |
| --- | --- |
| Not infected | Normal placenta without parasites or pigment (hemozoin) |
| Active-acute infection | Parasites; Minimal pigment accumulation |
| Active-subacute infection | Parasites; Pigment in fibrin, but not monocytes |
| Active- chronic infection | Parasites; Pigment in monocytes (with or without pigment in fibrin) |
| Past infection | No parasites; Pigment in cells or fibrin |

In addition to evaluating placentas for evidence of malaria, our team of histopathologists will conduct a standardized pathologic evaluation of the placenta. ^224^

**6.3.2 Tuberculosis (TB)**

We will use a standard clinical algorithm to screen participants for TB. For patients with active TB, HIV-TB drug interactions, particularly for women on PI-based ART, will be addressed on a case-by-case basis by clinic and study staff. In particular, we will either make rifabutin available or give rifampicin with additional ritonavir to those women diagnosed with active TB.

**6.4 Medical care outside of the study clinic**

Routine medical care, including medications, will be provided in the study clinic free of charge, and patients will be reimbursed for costs of any transportation to and from our clinic. In addition, reimbursement for the cost of tests and drugs made by referrals, as recommended by the study physicians, to other clinics and services as well as after hours visits at Tororo District Hospital will be provided. We anticipate reimbursing the cost of most diagnostic tests (including laboratory tests, X-rays, and ultrasounds) and medications resulting from these referrals, using available funds. However, reimbursement of all diagnostic tests and treatment recommended outside the study clinic cannot be guaranteed in all circumstances because neither Makerere University, the University of California, San Francisco, nor the funding agency have a program to cover these costs. Decisions on reimbursement will be made by the study coordinator and the investigators, in conjunction with the funding agency if necessary.

## 6.5 After hours visits

Women will be encouraged to visit the Tororo District Hospital when urgent care is needed for her or her child outside of study clinic hours. Women will be instructed to inform hospital personnel of their involvement in the study at the time of registration and to visit the study clinic on the following day.

If a patient is diagnosed with uncomplicated malaria he or she will receive treatment from a hospital supply of artemether-lumefantrine and the doctors will be instructed to refer patients to our study clinic when it opens at 8 am the following day. If a patient is diagnosed with severe malaria, he or she will receive quinine following standard treatment guidelines. If the patient is under the age of 4 months and diagnosed uncomplicated malaria, they will be treated with quinine. Patients with non-malarial illnesses will be managed at the discretion of the Tororo District Hospital staff. Upon discharge, patients will receive follow-up at the study clinic as outlined above. Study personnel will visit the Tororo District Hospital daily to inquire about visits from study subjects and facilitate follow-up in the study clinic.

**6.6 Co-enrollment Guidelines**

The pregnant women from this study may co-enroll in observational studies, and they will be enrolled in the Antiretroviral Pregnancy Registry [http://www.apregistry.com](http://www.apregistry.com/)). They may not be co-enrolled in protocols that utilize study drugs contraindicated in section 7.7. The infants from this study may be recruited into two other program-related interventional studies depending on their HIV status. HIV-infected children will be eligible for a PI-based ART versus non-PI ART trial and the HIV-negative children will be eligible for a malaria drug prophylaxis trial. Furthermore, blood samples from women or children who develop malaria from any of the three clinical trials will be used for laboratory analysis of malaria parasite drug resistance. Co-enrollment in other studies will be determined on a case-by-case basis by the protocol team.

**7.0 TOXICITY MANAGEMENT**

The Division of AIDS (DAIDS) standardized Toxicity Table for Grading Severity of Adult and Pediatric Adverse Events (DAIDS AE Grading Table), Version 1.0, December, 2004; clarification AUGUST 2009 [http://rcc.tech-res-intl.com] will be used to screen for eligibility and to evaluate adverse events for pregnant and postpartum women (Appendix X). The one exception to using the DAIDS Toxicity Table is the Supplemental Toxicity Table for Grading of Severity of Cutaneous/Skin Rash/Dermatitis Adverse Experiences (Appendix XI) which will be used to report skin manifestations. (Adapted from PACTG P1039.) Study staff will receive extensive training in the identification and management of adverse events. In addition, women will be informed by the study team about potential adverse events associated with the prescribed ARTs and will be encouraged to present to the study clinic for all potential adverse events. The study clinic will remain open 7 days a week so that participants may present with potential adverse events as they arise.

In general, if Grade 3 or 4 toxicity is documented following the dosing of any antiretrovirals, study staff will assess for alternate explanations for clinical and laboratory abnormalities. If the grade 3 or 4 toxicity is judged to be study drug-related, study medications will be switched or temporarily held while the evaluation proceeds. For laboratory abnormalities, repeat laboratory testing will be performed within 72 hours. Study staff will follow participants with abnormal clinical findings and laboratory values at least weekly until toxicity resolves to less than or equal to Grade 2 or no further improvement is anticipated.

If a Grade 3 or 4 toxicity is reported but repeat evaluation shows Grade 1 or normal values (Grade 0), the site will notify the study team of the spurious nature of the event and it will be corrected in the clinical data base. Treatment will not necessarily be suspended if toxicities are caused by underlying infections or processes, or by drugs other than the study regimen.

Pregnant women will undergo additional clinical evaluation to rule out preeclampsia or other obstetrical complications that may contribute to lab abnormalities or signs or symptoms of clinical toxicity.

**7.1 Toxicity management for women by toxicity grade**

**Grade 1 and 2 Toxicities**:

- Continue Study Regimen
- If toxicity is suspected, the study subject should be evaluated immediately. Liver function tests, CBC with differential, creatinine, and creatinine phosphokinase should be tested as clinically indicated and the subject should be extensively counseled to re-present to the study clinic immediately if there is any worsening of symptoms.

**Grade 3 Toxicity:**

- In the setting of anemia, evaluate for causes other than drug toxicity. If toxicity is > Grade 2, zidovudine may be reduced to 200 mg PO BID or changed to tenofovir or stavudine without treatment interruption. Women who are switched to stavudine may have an increased risk of lactic acidosis and will be monitored closely for this complication.
- For other Grade 3 toxicity, the study regimen may be switched or stopped at the discretion of the study physician or continued while awaiting results of repeat laboratory tests. If repeat laboratory test confirms Grade 3 toxicity, and this toxicity is judged to be study drug related, stop the study regimen and repeat laboratory tests weekly until the toxicity resolves to < Grade 2. Study staff are encouraged to discuss toxicity management with the the clinical management team, which should be notified of regimen changes or modifications and, as necessary, consulted for advice.
- Participants with Grade 3 asymptomatic laboratory abnormalities may continue study treatment after discussion with the clinical management team only if the study staff have compelling evidence that the toxicity is NOT related to the study treatment.
- If the toxicity resolves to < Grade 2, re-start the study regimen. However, if > Grade 3 toxicity judged to be study drug-related recurs after restarting the study regimen, the study subject will have reached a study endpoint and will be off study treatment permanently.
- If the toxicity judged to be study drug-related does not resolve to < Grade 2 within 14 days, the subject will have reached a study endpoint and will be permanently discontinued from the study treatment.

Any study subject who is unable to continue on their study regimen due to reaching a toxicity study endpoint will continue to obtain necessary evaluations from our study team until resolution of the toxicity. Furthermore, the study subject will be referred to the local HIV clinic to continue necessary HIV care.

**Grade 4 Toxicity:**

- Participants who develop a symptomatic Grade 4 toxicity will have all study treatment stopped until resolution of the toxicity to a ≤ Grade 2, but substitution will be allowed at the discretion of clinical management team.
- Alternative study-provided or non-study provided medications should replace the study treatment thought to be most likely related to the toxicity, once HAART is restarted.
- For any Grade 4 laboratory test, repeat laboratory tests within 72 hours of receiving the laboratory results to confirm Grade 4 toxicity. If a life-threatening toxicity is confirmed by the on-site study staff and judged to be study drug-related, medications will be immediately discontinued and the subject will have reached a toxicity endpoint and participation in the study will be discontinued.
- Participants with Grade 4 asymptomatic laboratory abnormalities may continue study treatment after discussion with the protocol team only if the study staff have compelling evidence that the toxicity is NOT related to the study treatment and with permission of the clinical management team.
- If medications are stopped, they may be restarted if the subject has resolution of the toxicity to < Grade 2 within 14 days. Prior to restarting the study regimen, the clinical management team will need to give its approval. If toxicity recurs at ≥ Grade 3 and the toxicity is judged to be study drug-related, the study subject will have reached a study endpoint and will be permanently off the study regimen.
- If the toxicity does not resolve to < Grade 2 within 14 days and is thought to be study drug-related, the subject will have reached a study endpoint and will be off the study treatment permanently.

Any study subject who is unable to continue on their study regimen due to reaching a toxicity study endpoint will continue to obtain necessary evaluations from our study team until resolution of the toxicity. Furthermore, the study subject will be referred to the local HIV clinic to continue necessary HIV care.

**7.2 Toxicity management for women by diagnosis**

**7.2.1 Nausea/vomiting**

For cases of Grade 1 or 2 nausea or vomiting, study treatment will continue without interruption. Participants with Grade 1 and 2 nausea or vomiting may be treated symptomatically with permitted oral or rectal anti-emetic medication. If Grade 2 nausea and/or vomiting that persists despite symptomatic treatment is determined to be due to ZDV, the dose of ZDV should be reduced or the regimen may be switched to tenofovir or stavudine . Persistent nausea or vomiting despite ZDV dose reduction will lead to discontinuation of ZDV and switching of the regimen. For participants who experience nausea and vomiting related to the initiation of LPV/r, dose adjustment may be considered after consultation with the clinical management team.

Participants with Grade 3 or 4 nausea or vomiting judged to be study drug-related will discontinue all study drugs until the toxicity returns to Grade 2 or less and be treated symptomatically. Therapy should be resumed using the full doses of study drugs except for a dose reduction of ZDV to 200 mg BID or switching ZDV to tenofovir or stavudine. If Grade 3 or 4 nausea and vomiting recur upon the resumption of study medications despite symptomatic treatment and these symptoms are thought to be study drug-related, the study regimen should again be stopped or alternative antiretroviral agents should be considered. Study staff will consider the diagnosis of lactic acidosis syndrome, hyperemesis gravidarum or preeclampsia and other obstetrical complications and evaluate subjects for these events if clinically indicated.

**7.2.2 Diarrhea**

Participants with Grade 1 or 2 diarrhea will continue ART without interruption. Subjects with diarrhea of any Grade may be treated symptomatically with permitted anti-motility agents. For Grade 3 diarrhea judged to be study drug-related that is unresponsive to anti-motility agents and for which an alternative etiology (e.g., infectious diarrhea) is not established, all study medications will be interrupted until resolution of diarrhea to Grade 2 or less. If Grade 3 or 4 diarrhea recurs upon the resumption of study medications and this toxicity is judged to be study drug-related, all study medications will be interrupted and alternative antiretroviral therapy will be selected after consultation with the clinical management team.

**7.2.3 Zidovudine intolerance**

Subjects with zidovudine intolerance may undergo a dose reduction from the standard dose of 300mg PO BID to 200mg PO BID. Alternatively, participants may be switched to tenofovir or stavudine. A return to full dose should be considered if the intolerance resolves.

Zidovudine may also be substituted with tenofovir or stavudine in subjects with > Grade 3 headache, fatigue, or nausea/vomiting thought to be drug related and not responsive to dose reduction. Stavudine dosing is 30mg PO BID, regardless of weight. ^225^ Changing zidovudine to tenofovir or stavudine will not result in discontinuation or changing of the other antiretrovirals in the subjects’ study regimen. Since stavudine may be associated with an increased risk of lactic acidosis, women who are switched to stavudine will be monitored closely for this complication.

**7.2.4 Rash**

For Grade 1 or 2 rash, study treatment will continue without interruption. Participants with a Grade 1 or 2 rash may be treated symptomatically with permitted antipyretic, antihistamine, and/or non-steroidal anti-inflammatory medications (if postpartum), but should be monitored closely by study staff. Subjects with Grade 3 or 4 rash thought to be study drug-related will have all study medications held or switched unless the rash is determined to be unrelated to study medications. Once the rash resolves to Grade ≤ 2, the clinical management team will determine alternative regimens.

### **7.2.5 Electrocardiogram (ECG) abnormality**

If the QTc intervial is found to be prolonged (Grade 1 or higher) on Day 0, the participant will not be given AL and will instead receive an alternative anti-malarial treatment. If a participant develops QTc prolongation on Day 3 ECG (Grade 1 or higher), the participant will not receive AL for any subsequent malaria treatment and will instead receive alternative anti-malaria therapy. All cases of QTc prolongation regardless of grade will be discussed by the clinical management team to determine appropriate follow-up.

**7.3 Toxicity management for women by laboratory abnormality**

**7.3.1 Anemia**

In order to minimize the risk of severe anemia, women with a baseline hemoglobin <7.5 g/dL are excluded from the trial. In the setting of anemia, evaluate and treat for causes other than drug toxicity. If toxicity is > Grade 2, zidovudine may be reduced to 200 mg PO BID or changed to tenofovir or stavudine without treatment interruption. Women who are switched to stavudine may have an increased risk of lactic acidosis and will be monitored closely for this complication.

**7.3.2 Hepatotoxicity**

For subjects with Grade 3 AST and/or ALT elevations, study medications may be continued at the discretion of the clinical management team. Careful assessments will be done to rule out the use of alcohol or hepatotoxic herbs, non-study medication-related drug toxicity, viral hepatitis, or preeclampsia or other obstetrical complication as the etiology of the Grade 3 elevation. The possibility of lactic acidosis syndrome will also be explored. In the event of Grade 4 AST and/or ALT elevations judged to be study drug-related, all study medications will be discontinued until the toxicity returns to Grade 2 or less. If the Grade 4 elevation in AST or ALT recurs and is judged to be study drug-related, alternative ART therapy will be selected at the discretion of the clinical management team.

**7.3.3 Hyperglycemia**

If a subject is diagnosed with hyperglycemia, she will be continued on her treatment regimen and managed clinically. Metabolic assays, including fasting glucose and insulin, will be performed on stored specimens obtained at study entry, 30-34 weeks gestation and 24 weeks post-partum. These results will not be available in real-time.

**7.3.4 Neutropenia**

If > Grade 2 toxicity, zidovudine may be reduced to 200 mg PO BID or changed to tenofovir or stavudine.

**7.3.5 Proteinuria**

If > Grade 1 toxicity and pregnant, evaluate the subject for preeclampsia. Proteinuria could be confirmed with a 24 hour urine protein collection if available. The study regimen will be continued if proteinuria is found to be due to preeclampsia or other non-study drug etiology. If Grade 3 toxicity without preeclampsia or Grade 4 toxicity is present and this toxicity is judged to be study drug-related, the antiretroviral treatment regimen will be stopped or switched until proteinuria resolves to ≤ Grade 2 and restarted at that time if resolution occurs within 14 days. If Grade 3 or 4 toxicity recurs and is judged to be study drug-related, the study subject will have reached a study endpoint.

**7.3.6 Fetal or infant demise after study entry**

The mother will continue to be followed on study. Antiretroviral therapy may be continued at the discretion of the study team. Furthermore, the study team may choose to switch antiretroviral therapy for women who desire to get pregnant again while on-study, particularly those women receiving EFV. An autopsy is recommended whenever feasible. In addition, the following must be documented:

1. Ultrasound or fetal assessment(s) performed as clinically indicated to diagnose fetal demise.
2. Assessment of antepartum complications which may have caused fetal demise (including but not limited to: placenta previa, abruptio placenta, unexplained vaginal bleeding, uterine/abdominal trauma, intrauterine growth retardation, oligohydramnios, polyhydramnios, maternal/fetal blood incompatibility, maternal infection).
3. Obstetrical exam (fundal height, gestational age assessment, and cervical examination, if indicated).
4. Complete narrative of pregnancy course and assessment of relationship of fetal demise to study regimen sent to the team via e-mail. In the case of infant demise, a complete narrative of pregnancy and postpartum course for both mother and infant and assessment of relationship of infant demise to study regimen shall be sent to the team via email.
5. A copy of placental pathology and autopsy report is also requested (if autopsy performed).

**7.4 Toxicity management for infants by toxicity grade**

To report adverse events for infants, we will use the the DMID Pediatric Toxicity Table Nov 2007 (Appendix XVIII). The only exception to using the DMID Pediatric Toxicity Table is the DAIDS Supplemental Toxicity Table for Grading of Severity of Cutaneous/Skin Rash/Dermatitis Adverse Experiences (Appendix X) which will be used to report skin manifestations (Adapted from PACTG P1039.) Infants with any signs or symptoms of toxicity will be monitored and followed until the toxicity resolves.

For all toxicities except skin rash:

**Grade 1 and 2 toxicity:**

- Continue nevirapine and TS.

**Grade 3 toxicity:**

- Continue nevirapine and TS but repeat laboratory testing within 72 hours of receiving the laboratory results. If the repeat value is Grade 3 or higher and the toxicity is judged to be NVP or TS-related, NVP will be switched or temporarily held and/or TS will be held while the evaluation proceeds in consultation with the clinical management team.
- We will continue to follow the infant until the toxicity has resolved.

**Grade 4 toxicity:**

- Repeat laboratory testing within 72 hours of receiving the laboratory results to confirm. If repeat value is > Grade 3 and the toxicity is judged to be NVP or TS-related, NVP will be switched or temporarily held and/or TS will be held while the evaluation proceeds in consultation with the clinical management team. We will continue to follow the infant until the toxicity has resolved.

For skin rash toxicity:

For any grade rash, treatment should be provided according to the judgment of the study clinician. Pruritis or rash with pruritis and minor accompanying symptoms may be managed with antihistamines, antipyretics and/or non-steroidal anti-inflammatory medications. NVP has been rarely associated with the development of severe rash, including SJS and Toxic Epidermal Necrolysis (TEN).  The presence of constitutional symptoms makes the rash Grade 3 or 4.

The Supplemental Toxicity Table for Grading Severity of Adult and Pediatric Cutaneous/Skin Rash/Dermatitis Adverse Experiences (Appendix XI) and the Rash Management Guidelines MUST be used for grading rash toxicities.  Management of rash toxicities will be as follows.

**Grade 1 (erythema with or without pruritis):**

- Continue NVP (if receiving NVP) and/or TS.  If rash does not resolve within 14 days of onset, a study team discussion will be held to determine further management.

**Grade 2A (diffuse erythematous macular or maculopapular rash or dry:** desquamation with or without pruritis but without constitutional findings, or target lesions without blister/vesicle or ulceration in lesion)

- Continue NVP (if receiving NVP) and/or TS. If rash does not resolve within 14 days of onset, a study team discussion will be held to determine further management.

**Grade 2B (urticaria):**

- Continue TS.
- If receiving NVP and a definitive, alternative explanation for the rash/skin reaction cannot be determined, NVP will be switched or temporarily held while evaluating the event..
- If NVP is temporarily held:
  - If NVP cannot be ruled out as the cause of the rash/skin reaction within 1 week of discontinuing NVP or if the rash/skin reaction does not improve by at least 1 grade within 1 week of temporarily holding NVP, NVP will be permanently discontinued.
  - If the rash/skin reaction is definitely due to an alternative diagnosis, NVP may be reintroduced after consultation with the clinical management team AND after the rash has resolved to Grade1 or lower
- We will continue to follow the infant until the toxicity has resolved.

**Grade 3** **toxicity:**

- If there is no other explanation for the rash and the child is receiving NVP, NVP will be permanently discontinued.
- TS will be temporarily held.  TS can be restarted after consultation with the clinical management team AND  after the rash has resolved to Grade 1 or lower.
- We will continue to follow the infant until the toxicity has resolved.

**Grade 4 toxicity:**

- If there is no other explanation for the rash both NVP (if still receiving) and TS will be permanently discontinued.
- We will continue to follow the infant until the toxicity has resolved.

**7.5 Subject management plan**

There will be a clinical management team consisting of the principal and co-principal investigators. Clinical management questions will be posed to the team and responses will be available within 24 hours.

**7.5.1 Criteria for virologic failure**

A subject will meet criteria for virologic failure if she has any one of the following, confirmed by a second assay on a different day within the following thirty days:

1) < 1 log reduction in HIV RNA PCR at 8 weeks after study entry

2) If baseline viral load started at < 5000 copies/mL, HIV RNA PCR >

400 copies/mL at 8 weeks after study entry

**7.5.2 Management of virologic failure**

Onsite study staff will query the clinical management team in cases of virologic failure to determine if treatment changes or alterations in breastfeeding recommendations are indicated. In addition to administering adherence questionnaires at each study visit, study staff will also conduct adherence counseling as needed. At the discretion of the clinical management team, intrapartum and infant antiretroviral prophylaxis or breastfeeding recommendations may be guided by maternal viral load or other maternal conditions such as poor adherence, even if the criteria for virologic failure per Section 7.5.1. are not met.

**7.5.3 Subsequent pregnancy management**

Trained study staff will extensively counsel women in both the antepartum and postpartum setting on postpartum contraception and safe sex. The informed consent form will review the requirement for either (1) 2 forms of contraception (including at least one non-hormonal method) or (2) the use of an intrauterine device (IUD) or tubal ligation postpartum. Women will undergo pregnancy testing during the postpartum period (see Appendix I). Women who become pregnant during the postpartum study period will continue to be followed until 24 weeks postpartum from the index pregnancy. These women will continue on ART through the subsequent pregnancy, though those women randomized to EFV will be switched to a non-EFV containing regimen once the subsequent pregnancy is diagnosed.

**7.6 Allowed medications**

- Medications prescribed by the subject’s primary provider for the management of pregnancy and associated conditions, with the exception of the medications listed in section 7.7.
- Medications prescribed by the subject’s primary provider for the management of medical illnesses, with the exception of the medications listed in section 7.7.
- Inhaled and topical steroids (except intranasal or inhaled fluticasone)
- Betamethasone or dexamethasone, intramuscular injection, for the induction of fetal lung maturity
- Ergot alkaloids to be used as a uterotonic agent in the case of postpartum hemorrhage **only if no other uterotonic options are available**.

**7.7 Disallowed medications**

- Sulphadoxine-pyrimethamine
- Anabolic steroids
- Cytokine inhibitors
- Cytokines (e.g., interleukins such as IL-2 and interferon)
- Systemic chemotherapeutic agents
- Grapefruit and grapefruit juice
- Chronic or recurrent use of glucocorticoids, prednisone, or systemic corticosteroids for longer than 14 days
- Intranasal fluticasone and inhaled fluticasone

**7.8 Criteria for study discontinuation**

- The subject (or legal guardian in the case of infant participant) refuses further treatment and/or follow-up evaluations
- The study staff or participant’s medical provider determines that further participation in the study would be detrimental to the subject’s or her fetus’ or infant’s health and/or well being
- The subject is noncompliant with the study requirements in a manner that is either detrimental to the subject’s health or interferes with the validity of the study results.

**7.9 Management of antiretroviral medication upon study discontinuation or end of study**

The discontinuation of antiretroviral therapy has numerous potential implications including the development of resistance, particularly when discontinuing an NNRTI-based regimen, and the risk of HIV transmission to an infant from a mother discontinuing antiretroviral therapy while still breastfeeding. Study staff will follow the Standard Operating Procedure (SOP) manual in referring all study participants to local HIV care at the time of study discontinuation or study end. The SOP delineates the logistics of transferring women and infants into care and includes recommendations in the management of women following antiretroviral discontinuation. For those women randomized to the EFV-containing arm who are not eligible for ARVs according to the Ugandan MOH guidelines, the SOP describes specific management recommendations including the use of an NRTI tail following the discontinuation of EFV. For those women who continue to breastfeed past the 24 week postpartum study endpoint, the SOP describes specific management recommendations including the continuation of antiretroviral therapy throughout the breastfeeding period, even for those women who would not otherwise qualify for antiretroviral therapy by WHO and Ugandan Ministry of Health guidelines.

**8.0 MONITORING OF ADVERSE EVENTS AND MANAGEMENT**

**8.1 Monitoring and Reporting of Adverse Events**

**8.1.1 Definitions**

An adverse event is defined as "any untoward medical occurrence in a patient or clinical investigation subject administered a pharmaceutical product that does not necessarily have a causal relationship with this treatment" (ICH Guidelines E2A). An adverse event can further be broadly defined as any untoward deviation from baseline health, which includes:

- Worsening of conditions present at the onset of the study
- Deterioration due to the primary disease
- Intercurrent illness
- Events related or possibly related to concomitant medications

(International Centers for Tropical Disease Research Network Investigator Manual, Monitoring and Reporting Adverse Events, 2003).

- - 1. **Identification of Adverse Events**

At each scheduled and unscheduled visit to the clinic, study clinicians will assess patients according to a standardized clinical record form. A severity grading scale, based on toxicity grading scales developed by the NIH Division of AIDS Toxicity Tables, will be used to grade severity of all symptoms, physical exam findings, and laboratory results (Appendix X). Any new event will be considered an adverse event.

Data will be captured on the incidence of all adverse events, regardless of severity. For each adverse event identified and graded as moderate, severe or life threatening and felt to be possibly, probably or definitely related to study drugs, an adverse event report form will be completed. In addition, an adverse event form will be completed for all serious adverse events and unexpected events, regardless of severity. An adverse event report form will not be completed for events classified as mild (unless they are serious or unexpected), as mild symptoms are common and difficult to distinguish from signs and symptoms due to malaria and other common illnesses. The following information will be recorded for all adverse experiences that are reported:

1. Description of event
2. Date of event onset
3. Date event reported
4. Maximum severity of the event
5. Maximum suspected relationship of the event to study drugs
6. Whether the event is a serious adverse event
7. Initials of the person reporting the event
8. Outcome
9. Date event resolved

**8.1.3 Reporting of Adverse Events**

Guidelines for reporting of adverse events provided by NICHD, UCSF Committee for Human Research, the Ugandan National Council for Science and Technology, Makerere University IRB, and Abbott Laboratories will be followed as summarized in Table 7.

**Table 7. Guidelines for reporting adverse events**

| **Institution** | **Type of Adverse Events** | **When to Report** |
| --- | --- | --- |
| **NICHD** | - Definitely, Probably, or Possibly related **AND** Serious^*^ or Unexpected^±^ | - Within 10-working days of awareness |
| **UCSF-CHR** | - Definitely, Probably, or Possibly related **AND** Serious^*^ or Unexpected^±^ | - Within 10-working days of awareness |
| **UNCST** | - All Serious^*^ or Unexpected^±^ events irrespective of relationship | - Within 7-calendar days of awareness - All other reportable events within 15-calendar days of awareness |
| **MU**  **SOMREC** | - All Serious^*^ or Unexpected^±^ events irrespective of relationship | - Fatal or life-threatening events within 3 working days of awareness - All other SAEs within 10 working days of awareness |
| **NDA** | - All serious and Unexpected events irrespective of relationship | - Within 7-calendar days of awareness |
| **Abbott Laboratories** | - Definitely, Probably, or Possibly related **AND** Serious^*^ or Unexpected^±^ - Only AEs in the PI-group | - Within 72 hours of awareness |
| **FDA** | - Definitely, Probably or Possibly related **AND BOTH** Serious^*^ AND Unexpected^±^ | - For fatal or life-threatening events, by telephone or fax within 7 calendar days of first awareness - All other reportable events within 15 calendar days of first awareness |

****Serious Adverse Event (SAE)*** is any AE that results in any of the following outcomes:

• Death,

• Life-threatening adverse experience,

• Inpatient hospitalization or prolongation of existing hospitalization,

• Persistent or significant disability/incapacity,

• Congenital anomaly/birth defect, or cancer, or

• Any other experience that suggests a significant hazard, contraindication, side effect or precaution that **may require medical or surgical intervention** to prevent one of the outcomes listed above,

• Event occurring in a gene therapy study

• Event that changes the risk/benefit ratio of the study.

***±Unexpected Adverse Event.*** An adverse event is defined as being unexpected if the event exceeds the nature, severity, or frequency described in the protocol, consent form and investigator brochure (when applicable). An unexpected AE also includes any AE that meets any of the following criteria:

• Results in subject withdrawal from study participation,

• Due to an overdose of study medication, or

• Due to a deviation from the study protocol

**9.0 STATISTICAL CONSIDERATIONS**

**9.1 General design issues**

This will be an open-label, two-arm, single-center randomized clinical trial designed to test the hypothesis that PI-based ART will reduce placental malaria compared to non-PI regimens in HIV-infected pregnant women. Secondary objectives are to compare the rates of LBW, stillbirth, spontaneous abortion, preterm delivery and neonatal death as a composite clinical outcome, maternal malaria, maternal anemia, maternal and infant grade 3 or 4 toxicity, preeclampsia, HIV RNA suppression and CD4 count at delivery and 24 weeks postpartum, HIV genotypic drug resistance, and perinatal HIV infection as well as to assess pharmacokinetic properties of the medication in women and infants.

The primary analysis for this study will be based on the prevalence of placental malaria at the time of delivery in each treatment arm, defined by positive thick smears or PCR of placental blood. We calculated the sample size for this study to show an effect size of 48.5% reduction in placental malaria with a 0.05 level of statistical significance (two-sided) and power of 80%.

The target population for this study is HIV-infected pregnant women between 12-28 weeks gestation who are 16 years of age or older living in the Tororo district of Uganda, a malaria-endemic area. We will enroll primigravid and multigravid women at all CD4 cell counts. The study population will include both antiretroviral naïve women and women who received single-dose nevirapine (alone or with ZDV or ZDV/3TC or other abbreviated monotherapy or dual antiretroviral therapy as part of PMTCT) more than 24 months prior to enrollment. Women with current or prior HAART or prior NVP exposure within 24 months of enrollment (alone or with ZDV or ZDV/3TC or other abbreviated monotherapy or dual antiretroviral therapy as part of PMTCT) will be excluded from the trial. Women will continue on their treatment regimen until 24 weeks postpartum or until cessation of breastfeeding if that occurs first. All women will receive insecticide treated nets and intermittent preventative therapy with trimethoprim-sulfamethoxazole (TS). As per current WHO guidelines, women will be counseled to exclusively breastfeed for 6 months, introduce complementary foods at 6 months of life, continue breastfeeding until 1 year of life and wean over the course of 1 month. ^163^ All infants will receive 6 weeks of nevirapine starting at birth, and TS from 6 weeks of life until 6 weeks after weaning from breast milk [128].

Blinding would be the preferred approach to the execution of this study to avoid systematic bias, but is not possible due to the large pill burden and logistical constraints. Potential sources of bias with an open-label study include increased attribution of adverse events to a specific study regimen and introduction of biased co-interventions by providers if they perceive the study regimen as suboptimal. In order to minimize these biases, we will utilize standardized protocols for the identification and management of adverse events. In order to minimize ascertainment bias, we will use a standardized schedule of evaluation in addition to blinding laboratory personnel from study drug assignment. Objective measures such as LBW, stillbirth and spontaneous abortion are less vulnerable to bias, and we will use calibrated scales and specific definitions of all outcomes.

Over the course of conducting this trial, there may be new information which affects the conduct of our study. For instance, the recommended duration of breastfeeding for HIV-infected women in Uganda may change. Optimal dosing of PIs in pregnant women is still under investigation. As data arise to better guide management, we will modify our study as appropriate, maintaining as much as possible our overarching aim of evaluating the use of PIs among pregnant and breastfeeding women as a means of preventing the poor maternal and infant sequelae associated with HIV-malaria co-infection in an area of high malaria transmission.

For the pharmacokinetic portion of the study, we will initially evaluate only a subset of plasma and hair ART levels in women and their infants, although hair and plasma will be collected on all of the study participants. Stek et al^173^ calculated a sample size of 25 women to be adequate to detect statistically significant differences in LPV exposure during pregnancy and postpartum conditions. For the comparison of third-trimester versus postpartum lopinavir exposure, the comparisons will be made at the within-subject level, using 90% confidence limits for the geometric mean ratio of hair LPV or EFV level in pregnant versus non-pregnant conditions. When the true geometric mean of the ratio (the antilog of the true mean of the log ratios) of the pharmacokinetic hair exposure parameters for pregnant and non-pregnant conditions has a value of 1, this indicates equal geometric mean pharmacokinetic exposure parameters for the pregnant and non-pregnant conditions. If the 90% confidence intervals (CIs) are entirely outside the limits, the pharmacokinetic exposure parameters for the pregnant and non-pregnant conditions are considered different, and a dose modification might be indicated. If, on the other hand, the 90% confidence limits are entirely within the limits, the drug exposure is likely to produce a similar antiviral effect for both the pregnant and the non-pregnant conditions, and no dose modification is needed. If the 90% confidence interval overlaps with the limits, these data alone do not support any conclusions. The difference in plasma or hair LPV or EFV levels ante and postpartum will also assessed with the Wilcoxon signed-rank test. Gandhi et al found a strong correlation between ART exposure as measured by hair levels and treatment efficacy as measured by virologic response in 70 participants in the WIHS observational cohort on LPV/r and 68 participants on EFV^226^. We will therefore use a sample size of 80 infants and 80 women in the remainder of the pharmacokinetic evaluations to evaluate the relationship between hair exposure and LPV or EFV toxicity or efficacy. We plan to collect hair samples on all consenting women and infants but will initially only analyze samples sequentially from the first 50 women (25 in each arm) and first 80 infants (40 in each arm).

**9.2 Outcome measures**

**9.2.1 Primary outcome measure**

The prevalence of placental malaria at the time of birth in each treatment group. Placental malaria will be defined as positive parasitemia on Giemsa-stained thick smears or PCR from placental blood.

- - 1. **Secondary outcome measures**

1. The prevalence of placental malaria at the time of birth in each treatment group using alternate diagnostic modalities:

Malaria Rapid Diagnostic Tests (RDTs)

Histopathology

1. The prevalence of a composite clinical outcome defined by low birth weight, stillbirth (intrauterine fetal demise > 20 weeks gestational age), late spontaneous abortion (miscarriage between 12-20 weeks gestational age), preterm delivery (<37 weeks gestation), and neonatal death (death of a liveborn infant within first 28 days of life) in each treatment group.

We will also examine rates of PTD< 34 weeks gestational age, and gestational age at delivery as a continuous variable.

1. The rate of maternal malaria in each treatment group. Defined as # episodes of malaria per person-years in each arm of the study.
   1. Symptomatic infection, defined by fever and positive peripheral blood smear.
   2. Asymptomatic infection defined by positive peripheral blood smear but no fever.
2. The prevalence of severe maternal anemia, defined by hemoglobin < 8g/dl at any point during the trial in each treatment group.
3. The prevalence of Grade 3 or 4 toxicity at any point during the trial in the two treatment groups:

In women

In infants

1. Incidence of preeclampsia in each treatment group:

Preeclampsia, defined by hypertension > 140/90 on two occasions measured > 6 hours apart with ≥1+ proteinuria on clean catch urine dipstick

1. Virologic and immunologic efficacy of PI versus non-PI ART in pregnant and breastfeeding women:

Rates of maternal HIV RNA suppression to <400 copies/mL at delivery and at 24 weeks after the start of ART using the Roche Amplicor assay. Will also perform a separate analysis using <50 copies/mL as the cut-off for HIV RNA suppression.

Change in maternal CD4 lymphocyte count and %CD4 at week 24 of ART, from enrollment to delivery, and delivery to 24 weeks postpartum.

The presence of one or more new HIV ART resistance mutations in each treatment group using the 2008 IAS-USA panel genotypic classification, tested at time of delivery and at 24 weeks and 1 year postpartum, and at any point after treatment initiation if HIV RNA rebounds from <1000 to > 1000 copies/mL. HIV genotype will be measured by FDA-approved Trugene assay (Bayer Healthcare, Tarrytown NY) at the UCSF CFAR Virology Core Lab.

The incidence of perinatal HIV infection in each treatment group, measured by HIV DNA testing of infants at delivery, at follow-up visits and at 6 weeks following the cessation of breastfeeding.

- - 1. **Pharmacokinetics in women and infants**

1. Plasma and hair concentrations (in ng/mg) of LPV or EFV in 50 HIV-infected women (approximately 25 in PI-based arm and 25 in non-PI based arm) with samples collected during the third trimester (at least 4 weeks after starting on study medications) and concentrations of LPV or EFV in the same women approximately 6-12 weeks after delivery. The laboratory performing the plasma and hair analyses will be blinded to the pregnancy status of the participants.

Plasma and hair concentrations of LPV (ng/mg) in 80 infants whose mothers were on LPV-based regimens and plasma and hair concentrations of EFV (ng/mg) in 80 infants whose mothers were on EFV-based regimens from hair samples collected from infants at delivery

Plasma and hair concentrations of LPV (ng/mg) in 80 breastfeeding infants whose mother were on LPV-based regimens during lactation and plasma and hair concentrations of EFV (ng/mg) in 80 infants whose mothers were on EFV-based regimens during lactation collected from infants at 12 weeks and 24 weeks of life, or at the visit closest to the cessation of breastfeeding if that occurs prior to 24 weeks.

2) In a subset of patients we will also determine the drug concentrations of nevirapine and lopinavir using routinely collected DBS, and analyze PK exposure in relation to measures of nutritional status such as height and weight.

**9.3 Randomization and stratification**

Randomization will occur for eligible patients and be stratified by gravidity (G1 vs. G2+) and gestational age (<24 weeks vs. ≥ 24 weeks) to ensure balance between the intervention and control arms. To protect against temporal changes in either subject referral or study procedures, randomization will be blocked with a randomly permuted block size of 2 or 4. Thus, as enrollment progresses there will be approximately the same number of subjects in each arm regardless of gravidity and gestational age. To achieve this, two sets of sealed sequentially numbered envelopes will contain the treatment assignment and be prepared in the central administrative data offices by a staff member not in contact with study subjects.

**9.4 Sample size and accrual**

**Sample Size:**

As discussed in section 9.2.1, the primary analysis will be based on the prevalence of placental malaria in each study arm. We would like to show an effect size of 48.5% reduction in placental malaria between the two study arms, based on previously observed 50% reduction in placental malaria among pregnant women receiving two-dose IPT-SP. We have preliminary data from a cohort of HIV-infected pregnant women both on HAART and abbreviated PMTCT regimens who have delivered at Tororo District Hospital. The prevalence of placental malaria among these women by either PCR or blood smear of placental blood is 20%. In order to detect a 50.7% reduction in placental malaria between treatment arms with a significance level of 0.05 (two-sided) and power of 80%, we need 273 subjects in each arm with evaluable placental malaria data. We estimate that in our study population, 3% will have a late spontaneous abortion (not evaluable for primary outcome), 4% will be lost to follow-up, and 8% will not have a placenta specimen available. We will thus need to enroll approximately 250 women in each arm, in order to have evaluable data on at least 215 subjects per arm. Table 8 shows a range of placental malaria prevalence in the non-PI arm, from very conservative low values to more realistic values given the malaria intensity in Tororo, and the corresponding detectable reduction in placental malaria prevalence in the intervention arm.

**Table 8. Range of protective efficacies detectable given an evaluable sample size of 215 per arm**

| Estimated prevalence of placental malaria in NNRTI-based ART arm | 10% | **15%** | 20% | 25% |
| --- | --- | --- | --- | --- |
| Reduced prevalence of placental malaria detectable in PI-based ART arm | 3.3% | **6.6%** | 10.3% | 14.3% |
| Minimum percentage reduction in placental malaria prevalence detectable | 67.0% | **56.0%** | 48.5% | 42.8% |

**Accrual:**

We will recruit HIV-infected women from the Tororo Hospital PMTCT program. The PMTCT program cares for approximately 380 HIV-infected pregnant women per year. Less than 1% of these women have prior ART exposure, and we do not anticipate this proportion will increase dramatically prior to study implementation. Approximately half of the 380 HIV-infected pregnant women who newly present each year initiate prenatal care prior to 26 weeks gestation. We thus estimate that with intensified outreach efforts, we will be able to recruit the 500 subjects needed over a 3 year period (see table 9).

Table 9. Estimated accrual per year

|  | Year 1 | Year 2 | Year 3 | *Total* |
| --- | --- | --- | --- | --- |
| *Pregnant women* | *83* | *200* | *217* | *500* |

**9.5 Monitoring**

**9.5.1 Data Safety Monitoring Board**

Pursuant to the NICHD policy for Data and Safety Monitoring: (http://www.nichd.nih.gov/funding/polices/datasafety.cfm?redorforprint=1) a Data Safety and Monitoring Board (DSMB) will be convened to provide oversight of the clinical trial. The role of the DSMB will be to review implementation and progress of the trial and to review the accumulating data from the study to detect early, significant benefit or harm for patients while the trial is in progress. In consultation with NICHD, the study team will convene the DSMB consisting of at least 5 members with expertise in the following 5 areas: 1) the specific disease(s) under study, 2) biostatistics, 3) epidemiology, 4) ethics/patient advocacy, and 5) clinical trials. Board membership should consist of persons completely independent of the investigators who have no financial, scientific, or other conflict of interest with the trial. The DSMB will convene annually to review study progress and safety and may be called into ad hoc sessions as the Board sees fit or at the request of the study Principal Investigator or NICHD. At the annual meeting, the study Statistician and Principal Investigator will present the trial progress and safety and efficacy data, including the results of any planned interim analyses, to the DSMB for consideration. Following its meetings, the DSMB will present its recommendations in writing to continue, modify, or terminate the trial to NICHD and the study Principal Investigator.

**9.5.2 Interim analysis and stopping guidelines**

Over the course of the trial, we will perform two interim analyses in addition to the final analysis for a total of three sequential evaluations of study outcomes. The interim analyses will be performed when the study has accrued approximately 1/3 (n=167) and 2/3 (n=334) of the study subjects. For each interim analysis, a standardized test statistic will be calculated for the proportion of poor maternal or fetal/infant outcomes based on accrued data. If this statistic exceeds the nominal critical value calculated using the error spending function (see table below), then a statistically significant result will have been achieved at the time of that analysis. In that event, the sponsor will be notified and a report submitted for review by the DSMB. In each interim analysis, the study team will present information on recruitment and the results of the interim analyses to the DSMB, which will review the data and recommend a course of action.

Table 10. Schedule of 2 interim analyses and final analyses: boundaries to monitor study outcomes*

| Number of Evaluable Subjects Accrued | Test Statistic Lower Bound | Test Statistic Upper Bound | Alpha | Cumulative Alpha |
| --- | --- | --- | --- | --- |
| 167 | -3.71 | 3.71 | 0.00010 | 0.00021 |
| 334 | -2.51 | 2.51 | 0.00601 | 0.01210 |
| 500 | -1.99 | 1.99 | 0.02313 | 0.05000 |

*This analysis assumes alpha = 0.05 (two-sided test), O’Brien-Fleming boundaries (DeMets error-spending function) and 500 trial participants. We will utilize Programs for computing Group Sequential Boundaries Using the Lan-DeMets Method [212].

**9.6 Analysis**

**9.6.1 Primary analysis**

Using an intent-to-treat approach, we will statistically compare the prevalence of placental malaria (positive thick smear or PCR of placental blood) at the time of birth between the study arms using a Chi-Square test of two proportions. The study compares the standard of care treatment of HIV-infected pregnant women (non-PI-based ART control arm) to the study intervention (PI-based ART intervention arm). Our focus is to detect any improvement over standard of care so we will use a two-sided statistical test to determine if the intervention arm is superior to the control arm. Regarding evaluation of the primary endpoint, study participants who enter the study will fall into one of the following categories: late spontaneous abortion (miscarriage from 12-20 weeks gestation), stillbirth (intrauterine fetal demise > 20 weeks gestation), or live birth. Women who have a late spontaneous abortion will not be evaluable for the primary study analysis. However, they will be evaluable for other secondary analyses. Consideration of these possible outcomes, of logistical barriers in collecting placental blood on all stillbirths or deliveries and loss to follow-up have all been considered in our analytic strategy.

**9.6.2 Secondary analysis**

In addition to evaluating placental blood smear and PCR for malaria, we will evaluate the effect of the intervention on the prevalence of placental malaria using alternate diagnostic modalities. Using an intent-to-treat approach, we will statistically compare the prevalence of placental malaria as defined by at the time of birth between the study arms using a Chi-Square test of two proportions. To test for significant differences in the distribution of histopathologically defined placental malaria by treatment arm, we will use a 2x5 Fishers Exact test. To assess the relative effect of the intervention on poor obstetrical/fetal/neonatal outcomes we will statistically compare between study arms the prevalence of a composite clinical outcome (late spontaneous abortion, stillbirth, LBW, preterm delivery and neonatal death) occurring from study enrollment until 28 days following the birth. Rationales for this composite outcome measure are that placental malaria has been associated with each of these poor obstetrical, fetal and neonatal outcomes independently, and that these poor outcomes lie on the chronological spectrum of placental malaria infection. We will compare the 2 arms of the study using a Chi-Square test of two proportions. Given the approximate frequency of LBW, still birth, spontaneous abortion, preterm delivery and neonatal death likely to be observed in this population of ~19% based on data from the CDC sponsored PMTCT program in Tororo and the Tororo District Hospital prenatal care program, and the multi-factorial nature of this outcome, comparisons are likely to be underpowered. However the relationships still bear examination. Per design, the study relies on randomization to equally distribute pregnant women with variable gestational age at study entry and the prevalence of multiple gestation (twinning). If a significant imbalance in gestational age or multiple gestation distribution exists between the study arms, we will employ multiple logistic regression with gestational age or multiple gestation as a covariate in addition to study arm to control for the potential imbalance.

As part of our analytic plan, we will conduct a mediation analysis of placental malaria as a mediator of the association between PI-based ART and the combined poor obstetrical/fetal/infant outcome measure to evaluate the relative influence of a reduction in the prevalence of placental malaria on the prevalence of poor obstetrical/fetal/infant outcome. We will structure the mediation analysis using the intervening variable model for binary outcomes as put forth by MacKinnon [202]. As the prevalence of placental malaria is also a binary measure, we will use the suggested extension of the model to account for binary outcome and mediator variable structures. To test for significant evidence of mediation, we will employ the product of coefficients tests approach using the asymmetric distribution of the product method [203]. To determine the confidence interval we will utilize the PRODCLIN software program which estimates the interval by numerical integration [204, 205]. This approach has been shown through comparison with 11 other mediation analytic methods to provide the best combination of low type I error rate, statistical power, and coverage [206]. To estimate the relative influence of placental malaria on infant low birth weight, stillbirth, late spontaneous abortion, preterm delivery and neonatal death, we will use a difference in coefficients method with standardization of coefficients and apply the formula to estimate the proportion of the total effect accounted for by the mediating/intervening variable of maternal/placental malaria. If we note maldistribution in the prevalence of histopathologically-defined placental malaria between the 2 arms, we will control for this variable in the mediation analysis. [207, 208]

Comparison of the prevalence of maternal anemia and maternal asymptomatic parasitemia will be done using repeated measures logistic regression to model the influence of the treatment arm on the probability of the outcome of interest being observed. Comparison of the incidence of maternal malaria will be done using Poisson regression, the standard approach for count data. Testing for overdispersion in the Poisson regression will be done and variances adjusted accordingly to produce valid p-values and confidence intervals.

To test the hypothesis that maternal and infant safety and tolerance of PI versus non-PI based ART during pregnancy and breastfeeding are not significantly different, we will measure the incidence of significant adverse events among women and infants during pregnancy and breastfeeding. We will define a significant adverse event as the occurrence of any grade 3 or 4 toxicity. We anticipate that our study will not be adequately powered to test for equivalence for this aim. Nonetheless, these outcomes are clinically relevant and warrant evaluation given the unique opportunity to study these questions in the setting of a randomized-controlled trial.

To test for significant difference in adverse event incidence, using an intent-to-treat analysis we will use Poisson regression to model the independent effect of PI-based ART, accounting for observation time. These models will include the logarithm of the follow-up time as an offset (an offset is similar to a predictor variable, but its coefficient is set to 1.0 instead of being estimated). Because the outcome variable is implicitly logarithmically transformed in Poisson regression, this will have the net effect of modeling log (treatments/follow-up), as desired. We will translate the fitted coefficients and their confidence bounds into percentage effects with the formula 100* [exp(coefficient)-1]. We will initially model a composite incidence density measure combining maternal and infant significant adverse events, and subsequently model events during pregnancy and events following delivery separately, and perform a sub-analysis of infants only.

We will also perform additional analyses comparing a number of measures of tolerability and safety between the PI-based ART and non PI-based ART arms, including treatment limiting toxicity, preterm delivery and, preeclampsia. For continuous outcomes measured at a single time point where the underlying distributions are unknown, we will use the non-parametric Wilcoxon 2-sample test. For continuous outcomes measured at multiple time points, we will employ generalized linear mixed modeling allowing for non-normal response data and random subject effects where indicated.

A review of three studies of pregnant women receiving ART indicates that significant grade 3 or 4 toxicity occurs in approximately 3 to 19% of ART treated pregnant women. [181, 182, 209, 210] Additionally, between 8 and 30% of women discontinue ART for medical reasons including intolerable side-effects. Assuming a large amount of overlap between these adverse outcomes, we anticipate that approximately 20% of women will experience one or more significant adverse events during pregnancy or discontinue an ART regimen due to side-effects. Given 250 subjects in each treatment arm and assuming near normally distributed data for the log of {(1 + #AE events)/follow-up time} and a slightly smaller standard deviation than is observed for malaria events (sd=.18), this study will have at least 80% power to detect a difference between the two treatment arms of approximately 11% in grade 3 or 4 toxicity incidence density with a 5% type-I error rate and a two-sided test.

To compare the virologic efficacy of PI vs. non-PI ART, we will test for differences in the proportion of pregnant women who achieve HIV viral RNA suppression at the time of delivery and at 24 weeks after the start of ART Initially, we will define HIV RNA suppression as an HIV RNA copy number of <400, followed by a separate analysis using <50 copies as indicating virologic suppression. To evaluate the statistical significance of observed differences in the proportion virologically suppressed, we will use a two group test of equivalence of proportions using standard equations. [211]

To compare immunologic efficacy of PI versus non-PI based ART in pregnant and breastfeeding women, we will test for equivalence in the change from baseline in maternal CD4 cell count at two time points, delivery and the cessation of breastfeeding. We will test for significant differences in change in CD4 cell count from baseline between the PI-based and non-PI based arms using multivariate repeated measures modeling on change in CD4 cell count, with treatment arm as the predictor and indicators for calendar time between observations and a random (individual) effect to allow for different individual CD4 count trajectories. This type of within-person modeling has the advantage of a reduction in the large variance often observed in between-subjects comparisons traditionally used in analysis of change in CD4 cell counts between groups.

We will also perform additional analyses comparing the duration of HIV viral RNA suppression between the PI-based ART and non PI-based ART arms using survival analysis and Cox proportional hazards regression to identify multivariate predictors of time to failure of viral suppression. To evaluate similarity in the proportion of vertically HIV-infected infants between the PI-based and non-PI based arms, we will compare the proportion of infants with a positive HIV DNA PCR test at delivery and at 58 weeks post partum using a two group test of equivalence of proportions (two separate tests). Likewise we will compare the proportion of mothers with one or more new HIV ART resistance mutations identified at delivery and at 58 weeks postpartum.

We will not be adequately powered to test for equivalence for HIV RNA suppression or change in CD4 cell count. Nonetheless, these outcomes are clinically relevant and warrant evaluation given the unique opportunity to study these questions in the setting of a randomized-controlled trial. Data from the DREAM cohort in Mozambique suggest that approximately 75% of HIV-infected pregnant women receiving optimal care will have an undetectable viral load at delivery and approximately 60% will be undetectable at the cessation of breastfeeding. [209] When the sample size in each group is 247, a two-group large-sample normal approximation test of proportions with a one-sided 0.05 significance level will have 80% power to reject the null hypothesis that the test and the standard are not equivalent (the difference in proportions, pT - pS, is 0.097 or farther from zero in the same direction), in favor of the alternative hypothesis that the proportions in the two groups are equivalent, assuming that the expected difference in proportions is 0 and the proportion in the standard group is 0.75. Based on a simple comparison of mean change in CD4 cell count from baseline to 2 months after the start of ART, we estimate the standard deviation for change in CD4 cell count is approximately 288. When the sample size in each group is 250, a two group 0.05 one-sided t-test will have 80% power to reject the null hypothesis that the two study arms are not equivalent (the difference in means, mT - mS, is 64.136 cells or farther from zero in the same direction) in favor of the alternative hypothesis that the means of the two groups are equivalent, assuming that the expected difference in means is 0 and the common standard deviation is 288. Regarding vertical transmission, we estimate that the proportion of vertically infected infants by 58 weeks will be approximately 5%. With our study, two-group large-sample normal approximation test of proportions with a one-sided 0.05 significance level will have 80% power to reject the null hypothesis that the two arms are not equivalent (the difference in proportions, pT - pS, is 0.049 or farther from zero in the same direction) in favor of the alternative hypothesis that the proportions in the two groups are equivalent, assuming that the expected difference in proportions is 0 and the proportion in the standard group is 0.05. This translates to a detectable difference when one arm has a 5% vertical transmission prevalence and the other arm is 1/10 of a percent (other arm is lower) or 9.9% (other arm is higher). Currently we are unable to estimate power for the comparison of the proportion of subjects with new resistance mutations secondary to the unavailability of resistance data in this population.

We will investigate differences in ART levels (by both plasma and hair) found in samples from woman and infants at selected time-points by calculating a 90% confidence interval on the geometric mean of the ratio (the antilog of the true mean of the log ratios) for ART levels within subjects and determining if the confidence interval contains the value 1 (indicating no difference in ART levels between time-points).

**10.0 DATA COLLECTION AND MONITORING**

**10.1 Record Keeping**

All clinical data will be recorded onto standardized case record forms (CRFs) by study physicians. Laboratory data will be recorded in a laboratory record book by the study laboratory technologists and then transferred to the case record forms by study coordinators, who will review the case record forms frequently for completeness and accuracy. Data will be entered directly from CRFs into a computerized database or transferred from the CRFs onto standardized data extraction forms and then into a computerized database. All computerized data will be double entered to verify accuracy of entry. Electronic data including all study databases and supporting electronic documentation will be archived to large-scale digital tape on a daily basis. On a monthly basis, a complete backup tape will be transported off-site to the Kampala Data Management Center (DMC) for rotating secure storage. In addition, the database from the backup will be placed onto one of the Kampala DMC servers as a data mirror for read-onl y access in the event that the Tororo web-site becomes temporary unavailable.

**10.2 Data Quality Assurance and Monitoring**

In order to insure data quality, the study Data Manager will perform a quarterly data quality audit. For this audit a 1% random sample of study forms entered into the data management system from the previous 2 weeks will be selected and compared for accuracy with the original case-report forms and source documents. In addition the study the Data Manager will perform monthly reviews of the 100% double data entry data verification logs and the data management system audit trail log to identify potential data quality issues**.**

**11.0 HUMAN SUBJECTS**

**11.1 Treatment and Compensation for Injury**

If the participant is injured as a result of being in this study, treatment will be available through Tororo District Hospital. Makerere University, UCSF, and NICHD do not normally provide any other form of compensation for injury.

**11.2 Costs to the Subjects**

There will be no cost to the participant or their parents/guardians for participation in this study.

**11.3 Reimbursement of Subjects**

Participants will not be paid for their participation in the study. We will provide all routine medical care, including evaluations, medications available in our clinic, and cost of any transportation free of charge. In addition, we will reimburse the cost of consultation for referrals made by study physicians to other clinics and services within Tororo District Hospital and visits the cost of most diagnostic tests (including laboratory test, X-rays, and ultrasounds) and medications resulting from referrals by the study team, using available funds. However, reimbursement of all diagnostic tests and treatment recommended outside the study clinic cannot be guaranteed in all circumstances.

**11.4 Institutional Review Board (IRB) Review and Informed Consent**

This protocol, all procedures and consent forms, and any subsequent modifications must be reviewed and approved by the IRBs of all the participating institutions in both the U.S. and in Uganda. This includes the UCSF Committee on Human Research (CHR), the MU School of Medicine - Research and Ethics Committee (SOM-REC), and the Uganda National Council of Science and Technology (UNCST).

All consent forms will be translated into the local language (Jopadhola, Teso, Swahili, Luganda, and English) and back-translated into English to ensure correct use of language. Consent forms will be read aloud to parents by trained study interviewers. The informed consent will describe the purpose of the study, all the procedures involved, and the risks and benefits of participation. Interviewers will ask parents/guardians of study participants to summarize the study and explain the reasons why they want to participate. Either a signature or a thumbprint (for parents/guardians who cannot read) will be acceptable to confirm informed consent for participation in the study.

**11.5 Study Discontinuation**

This study may be discontinued at any time by the NIH, respective IRBs or other Governmental agencies in the United States or Uganda as part of their duties to ensure that research subjects are protected. Furthermore, a Data Safety and Monitoring Board (DSMB) will be established by the study team in cooperation with the NIH to assess at specified intervals the progress of the clinical trial, safety data and critical efficacy variables, and will recommend to the NIH whether to continue, modify or terminate the trial. Interim analysis reports will be submitted to the DSMB for review of the data and to recommend a course of action. The DSMB will determine whether to stop the study for early evidence of intervention inferiority or safety concerns after a thorough review of interim data.

**12.0 PUBLICATION OF RESEARCH FINDINGS**

The findings from this study may be published in a medical journal. No individual identities will be used in any reports or publications resulting from the study. The researchers will publish results of the trial in accordance with NICHD, UNCST, UCSF, and Makerere University guidelines.

**13.0 BIOHAZARD CONTAINMENT**

As the transmission of HIV and other blood-borne pathogens can occur through contact with contaminated needles, blood, and blood products, appropriate blood and secretion precautions will be employed by all personnel involved in the drawing of blood, exposure to blood and secretions, and shipping and handling of all specimens for this study. We will follow the current guidelines set forth by the Centers for Disease Control and Prevention and the NIH. All infectious specimens will be transported using packaging mandated in the Federal Code of Regulations, CDC 42 CFR Part 72.

**14.0** **REFERENCES**

1. ter Kuile FO, Parise ME, Verhoeff FH, Udhayakumar V, Newman RD, van Eijk AM, et al. The burden of co-infection with human immunodeficiency virus type 1 and malaria in pregnant women in sub-saharan Africa. *Am J Trop Med Hyg* 2004;71(2 Suppl):41-54.

2. UNAIDS. AIDS Epidemic Update: December 2006. Geneva: UNAIDS, 2006.

3. Bansil P, Jamieson DJ, Posner SF, Kourtis AP. Hospitalizations of pregnant HIV-infected women in the United States in the era of highly active antiretroviral therapy (HAART). *J Womens Health (Larchmt)* 2007;16(2):159-62.

4. Kourtis AP, Bansil P, McPheeters M, Meikle SF, Posner SF, Jamieson DJ. Hospitalizations of pregnant HIV-infected women in the USA prior to and during the era of HAART, 1994-2003. *Aids* 2006;20(14):1823-31.

5. Brocklehurst P, French R. The association between maternal HIV infection and perinatal outcome: a systematic review of the literature and meta-analysis. *Br J Obstet Gynaecol* 1998;105(8):836-48.

6. Dairo MD, Lawoyin TO, Onadeko MO, Asekun-Olarinmoye EO, Adeniji AO. HIV as an additional risk factors for anaemia in pregnancy: evidence from primary care level in Ibadan, Southwestern Nigeria. *Afr J Med Med Sci* 2005;34(3):275-9.

7. Ryder RW, Temmerman M. The effect of HIV-1 infection during pregnancy and the perinatal period on maternal and child health in Africa. *Aids* 1991;5 Suppl 1:S75-85.

8. Zvandasara P, Hargrove JW, Ntozini R, Chidawanyika H, Mutasa K, Iliff PJ, et al. Mortality and morbidity among postpartum HIV-positive and HIV-negative women in Zimbabwe: risk factors, causes, and impact of single-dose postpartum vitamin A supplementation. *J Acquir Immune Defic Syndr* 2006;43(1):107-16.

9. Stratton P, Tuomala RE, Abboud R, Rodriguez E, Rich K, Pitt J, et al. Obstetric and newborn outcomes in a cohort of HIV-infected pregnant women: a report of the women and infants transmission study. *J Acquir Immune Defic Syndr Hum Retrovirol* 1999;20(2):179-86.

10. Leroy V, Ladner J, Nyiraziraje M, De Clercq A, Bazubagira A, Van de Perre P, et al. Effect of HIV-1 infection on pregnancy outcome in women in Kigali, Rwanda, 1992-1994. Pregnancy and HIV Study Group. *Aids* 1998;12(6):643-50.

11. Connor EM, Sperling RS, Gelber R, Kiselev P, Scott G, O'Sullivan MJ, et al. Reduction of maternal-infant transmission of human immunodeficiency virus type 1 with zidovudine treatment. Pediatric AIDS Clinical Trials Group Protocol 076 Study Group. *N Engl J Med* 1994;331(18):1173-80.

12. Shaffer N, Chuachoowong R, Mock PA, Bhadrakom C, Siriwasin W, Young NL, et al. Short-course zidovudine for perinatal HIV-1 transmission in Bangkok, Thailand: a randomised controlled trial. Bangkok Collaborative Perinatal HIV Transmission Study Group. *Lancet* 1999;353(9155):773-80.

13. Wiktor SZ, Ekpini E, Karon JM, Nkengasong J, Maurice C, Severin ST, et al. Short-course oral zidovudine for prevention of mother-to-child transmission of HIV-1 in Abidjan, Cote d'Ivoire: a randomised trial. *Lancet* 1999;353(9155):781-5.

14. Kourtis AP, Lee FK, Abrams EJ, Jamieson DJ, Bulterys M. Mother-to-child transmission of HIV-1: timing and implications for prevention. *Lancet Infect Dis* 2006;6(11):726-32.

15. Kourtis AP, Butera S, Ibegbu C, Beled L, Duerr A. Breast milk and HIV-1: vector of transmission or vehicle of protection? *Lancet Infect Dis* 2003;3(12):786-93.

16. Fawzi W, Msamanga G, Spiegelman D, Renjifo B, Bang H, Kapiga S, et al. Transmission of HIV-1 through breastfeeding among women in Dar es Salaam, Tanzania. *J Acquir Immune Defic Syndr* 2002;31(3):331-8.

17. Miotti PG, Taha TE, Kumwenda NI, Broadhead R, Mtimavalye LA, Van der Hoeven L, et al. HIV transmission through breastfeeding: a study in Malawi. *Jama* 1999;282(8):744-9.

18. Leroy V, Newell ML, Dabis F, Peckham C, Van de Perre P, Bulterys M, et al. International multicentre pooled analysis of late postnatal mother-to-child transmission of HIV-1 infection. Ghent International Working Group on Mother-to-Child Transmission of HIV. *Lancet* 1998;352(9128):597-600.

19. Coutsoudis A, Dabis F, Fawzi W, Gaillard P, Haverkamp G, Harris DR, et al. Late postnatal transmission of HIV-1 in breast-fed children: an individual patient data meta-analysis. *J Infect Dis* 2004;189(12):2154-66.

20. Taha TE, Hoover DR, Kumwenda NI, Fiscus SA, Kafulafula G, Nkhoma C, et al. Late postnatal transmission of HIV-1 and associated factors. *J Infect Dis* 2007;196(1):10-4.

21. John-Stewart GC. Breast-feeding and HIV-1 transmission: how risky for how long? *J Infect Dis* 2007;196(1):1-3.

22. Marinda E, Humphrey JH, Iliff PJ, Mutasa K, Nathoo KJ, Piwoz EG, et al. Child mortality according to maternal and infant HIV status in Zimbabwe. *Pediatr Infect Dis J* 2007;26(6):519-26.

23. Shapiro RL, Lockman S, Kim S, Smeaton L, Rahkola JT, Thior I, et al. Infant morbidity, mortality, and breast milk immunologic profiles among breast-feeding HIV-infected and HIV-uninfected women in Botswana. *J Infect Dis* 2007;196(4):562-9.

24. Snow RW, Guerra CA, Noor AM, Myint HY, Hay SI. The global distribution of clinical episodes of Plasmodium falciparum malaria. *Nature* 2005;434(7030):214-7.

25. WHO. Roll Back Malaria: Malaria in Africa.

26. Desai M, ter Kuile FO, Nosten F, McGready R, Asamoa K, Brabin B, et al. Epidemiology and burden of malaria in pregnancy. *Lancet Infect Dis* 2007;7(2):93-104.

27. Steketee RW, Nahlen BL, Parise ME, Menendez C. The burden of malaria in pregnancy in malaria-endemic areas. *American Journal of Tropical Medicine & Hygiene* 2001;64(1-2 Suppl):28-35.

28. Guyatt HL, Snow RW. The epidemiology and burden of Plasmodium falciparum-related anemia among pregnant women in sub-Saharan Africa. *American Journal of Tropical Medicine & Hygiene* 2001;64(1-2 Suppl):36-44.

29. Guyatt HL, Snow RW. Impact of malaria during pregnancy on low birth weight in sub-Saharan Africa. *Clin Microbiol Rev* 2004;17(4):760-9, table of contents.

30. WHO. Roll Back Malaria: Malaria in pregnancy.

31. WHO. WHO Global Malaria Programme: Pregnant women and infants, 2007.

32. Whitworth J, Morgan D, Quigley M, Smith A, Mayanja B, Eotu H, et al. Effect of HIV-1 and increasing immunosuppression on malaria parasitaemia and clinical episodes in adults in rural Uganda: a cohort study. *Lancet* 2000;356(9235):1051-6.

33. Leaver RJ, Haile Z, Watters DA. HIV and cerebral malaria. *Trans R Soc Trop Med Hyg* 1990;84(2):201.

34. Niyongabo T, Deloron P, Aubry P, Ndarugirire F, Manirakiza F, Muhirwa G, et al. Prognostic indicators in adult cerebral malaria: a study in Burundi, an area of high prevalence of HIV infection. *Acta Trop* 1994;56(4):299-305.

35. Brentlinger PE, Behrens CB, Micek MA. Challenges in the concurrent management of malaria and HIV in pregnancy in sub-Saharan Africa. *Lancet Infect Dis* 2006;6(2):100-11.

36. Duffy PE, Fried M. Malaria in the pregnant woman. *Curr Top Microbiol Immunol* 2005;295:169-200.

37. Mbonye AK, Asimwe JB, Kabarangira J, Nanda G, Orinda V. Emergency obstetric care as the priority intervention to reduce maternal mortality in Uganda. *Int J Gynaecol Obstet* 2007;96(3):220-5.

38. Kiwanuka GN. Malaria morbidity and mortality in Uganda. *J Vector Borne Dis* 2003;40(1-2):16-9.

39. Ndyomugyenyi R, Magnussen P. Anaemia in pregnancy: Plasmodium falciparum infection is an important cause in primigravidae in Hoima district, western Uganda. *Ann Trop Med Parasitol* 1999;93(5):457-65.

40. Ndyomugyenyi R, Magnussen P. Malaria morbidity, mortality and pregnancy outcome in areas with different levels of malaria transmission in Uganda: a hospital record-based study. *Trans R Soc Trop Med Hyg* 2001;95(5):463-8.

41. Cottrell G, Deloron P, Fievet N, Sow S, Gaye O, Le Hesran JY. Prediction of Plasmodium falciparum placental infection according to the time of infection during pregnancy. *Acta Trop* 2006;98(3):255-60.

42. Ladner J, Leroy V, Simonon A, Karita E, Bogaerts J, De Clercq A, et al. HIV infection, malaria, and pregnancy: a prospective cohort study in Kigali, Rwanda. *Am J Trop Med Hyg* 2002;66(1):56-60.

43. Briand V, Cottrell G, Massougbodji A, Cot M. Intermittent preventive treatment for the prevention of malaria during pregnancy in high transmission areas. *Malar J* 2007;6(1):160.

44. Ticconi C, Mapfumo M, Dorrucci M, Naha N, Tarira E, Pietropolli A, et al. Effect of maternal HIV and malaria infection on pregnancy and perinatal outcome in Zimbabwe. *J Acquir Immune Defic Syndr* 2003;34(3):289-94.

45. McCormick MC. The contribution of low birth weight to infant mortality and childhood morbidity. *New England Journal of Medicine* 1985;312(2):82-90.

46. Murphy SC, Breman JG. Gaps in the childhood malaria burden in Africa: cerebral malaria, neurological sequelae, anemia, respiratory distress, hypoglycemia, and complications of pregnancy. *American Journal of Tropical Medicine & Hygiene* 2001;64(1-2 Suppl):57-67.

47. Steketee RW, Wirima JJ, Campbell CC. Developing effective strategies for malaria prevention programs for pregnant African women. *American Journal of Tropical Medicine & Hygiene* 1996;55(1 Suppl):95-100.

48. Reed SC, Wirima JJ, Steketee RW. Risk factors for anemia in young children in rural Malawi. *Am J Trop Med Hyg* 1994;51(2):170-4.

49. van Eijk AM, Ayisi JG, Ter Kuile FO, Misore AO, Otieno JA, Kolczak MS, et al. Malaria and human immunodeficiency virus infection as risk factors for anemia in infants in Kisumu, western Kenya. *Am J Trop Med Hyg* 2002;67(1):44-53.

50. Cornet M, Le Hesran JY, Fievet N, Cot M, Personne P, Gounoue R, et al. Prevalence of and risk factors for anemia in young children in southern Cameroon. *Am J Trop Med Hyg* 1998;58(5):606-11.

51. Cumberland P, Shulman CE, Maple PA, Bulmer JN, Dorman EK, Kawuondo K, et al. Maternal HIV infection and placental malaria reduce transplacental antibody transfer and tetanus antibody levels in newborns in Kenya. *J Infect Dis* 2007;196(4):550-7.

52. de Moraes-Pinto MI, Verhoeff F, Chimsuku L, Milligan PJ, Wesumperuma L, Broadhead RL, et al. Placental antibody transfer: influence of maternal HIV infection and placental malaria. *Archives of Disease in Childhood: Fetal & Neonatal Edition* 1998;79(3):F202-5.

53. Farquhar C, Nduati R, Haigwood N, Sutton W, Mbori-Ngacha D, Richardson B, et al. High maternal HIV-1 viral load during pregnancy is associated with reduced placental transfer of measles IgG antibody. *J Acquir Immune Defic Syndr* 2005;40(4):494-7.

54. Scott S, Cumberland P, Shulman CE, Cousens S, Cohen BJ, Brown DW, et al. Neonatal measles immunity in rural Kenya: the influence of HIV and placental malaria infections on placental transfer of antibodies and levels of antibody in maternal and cord serum samples. *J Infect Dis* 2005;191(11):1854-60.

55. Guyatt HL, Snow RW. Malaria in pregnancy as an indirect cause of infant mortality in sub-Saharan Africa. *Trans R Soc Trop Med Hyg* 2001;95(6):569-76.

56. van Geertruyden JP, Thomas F, Erhart A, D'Alessandro U. The contribution of malaria in pregnancy to perinatal mortality. *Am J Trop Med Hyg* 2004;71(2 Suppl):35-40.

57. Mutabingwa TK, Bolla MC, Li JL, Domingo GJ, Li X, Fried M, et al. Maternal malaria and gravidity interact to modify infant susceptibility to malaria. *PLoS Med* 2005;2(12):e407.

58. Whitty CJ, Edmonds S, Mutabingwa TK. Malaria in pregnancy. *Bjog* 2005;112(9):1189-95.

59. Bonner PC, Zhou Z, Mirel LB, Ayisi JG, Shi YP, van Eijk AM, et al. Placental malaria diminishes development of antibody responses to Plasmodium falciparum epitopes in infants residing in an area of western Kenya where P. falciparum is endemic. *Clin Diagn Lab Immunol* 2005;12(3):375-9.

60. Brustoski K, Kramer M, Moller U, Kremsner PG, Luty AJ. Neonatal and maternal immunological responses to conserved epitopes within the DBL-gamma3 chondroitin sulfate A-binding domain of Plasmodium falciparum erythrocyte membrane protein 1. *Infect Immun* 2005;73(12):7988-95.

61. Brustoski K, Moller U, Kramer M, Hartgers FC, Kremsner PG, Krzych U, et al. Reduced cord blood immune effector-cell responsiveness mediated by CD4+ cells induced in utero as a consequence of placental Plasmodium falciparum infection. *J Infect Dis* 2006;193(1):146-54.

62. Brustoski K, Moller U, Kramer M, Petelski A, Brenner S, Palmer DR, et al. IFN-gamma and IL-10 mediate parasite-specific immune responses of cord blood cells induced by pregnancy-associated Plasmodium falciparum malaria. *J Immunol* 2005;174(3):1738-45.

63. Broen K, Brustoski K, Engelmann I, Luty AJ. Placental Plasmodium falciparum infection: causes and consequences of in utero sensitization to parasite antigens. *Mol Biochem Parasitol* 2007;151(1):1-8.

64. Mockenhaupt FP, Bedu-Addo G, von Gaertner C, Boye R, Fricke K, Hannibal I, et al. Detection and clinical manifestation of placental malaria in southern Ghana. *Malar J* 2006;5:119.

65. Mockenhaupt FP, Rong B, Till H, Eggelte TA, Beck S, Gyasi-Sarpong C, et al. Submicroscopic Plasmodium falciparum infections in pregnancy in Ghana. *Tropical Medicine & International Health* 2000;5(3):167-73.

66. Mockenhaupt FP, Ulmen U, von Gaertner C, Bedu-Addo G, Bienzle U. Diagnosis of placental malaria. *J Clin Microbiol* 2002;40(1):306-8.

67. Rogerson SJ, Hviid L, Duffy PE, Leke RF, Taylor DW. Malaria in pregnancy: pathogenesis and immunity. *Lancet Infect Dis* 2007;7(2):105-17.

68. Rogerson SJ, Pollina E, Getachew A, Tadesse E, Lema VM, Molyneux ME. Placental monocyte infiltrates in response to Plasmodium falciparum malaria infection and their association with adverse pregnancy outcomes. *Am J Trop Med Hyg* 2003;68(1):115-9.

69. Menendez C, Ordi J, Ismail MR, Ventura PJ, Aponte JJ, Kahigwa E, et al. The impact of placental malaria on gestational age and birth weight. *J Infect Dis* 2000;181(5):1740-5.

70. Tako EA, Zhou A, Lohoue J, Leke R, Taylor DW, Leke RF. Risk factors for placental malaria and its effect on pregnancy outcome in Yaounde, Cameroon. *Am J Trop Med Hyg* 2005;72(3):236-42.

71. Adegnika AA, Verweij JJ, Agnandji ST, Chai SK, Breitling LP, Ramharter M, et al. Microscopic and sub-microscopic Plasmodium falciparum infection, but not inflammation caused by infection, is associated with low birth weight. *Am J Trop Med Hyg* 2006;75(5):798-803.

72. McGready R, Davison BB, Stepniewska K, Cho T, Shee H, Brockman A, et al. The effects of Plasmodium falciparum and P. vivax infections on placental histopathology in an area of low malaria transmission. *Am J Trop Med Hyg* 2004;70(4):398-407.

73. Menendez C, Ordi J, Ismail MR, Ventura PJ, Aponte JJ, Kahigwa E, et al. The impact of placental malaria on gestational age and birth weight. *Journal of Infectious Diseases* 2000;181(5):1740-5.

74. Mount AM, Mwapasa V, Elliott SR, Beeson JG, Tadesse E, Lema VM, et al. Impairment of humoral immunity to Plasmodium falciparum malaria in pregnancy by HIV infection. *Lancet* 2004;363(9424):1860-7.

75. Ned RM, Moore JM, Chaisavaneeyakorn S, Udhayakumar V. Modulation of immune responses during HIV-malaria co-infection in pregnancy. *Trends Parasitol* 2005;21(6):284-91.

76. Abrams ET, Kwiek JJ, Mwapasa V, Kamwendo DD, Tadesse E, Lema VM, et al. Malaria during pregnancy and foetal haematological status in Blantyre, Malawi. *Malar J* 2005;4:39.

77. Ayisi JG, van Eijk AM, ter Kuile FO, Kolczak MS, Otieno JA, Misore AO, et al. The effect of dual infection with HIV and malaria on pregnancy outcome in western Kenya. *Aids* 2003;17(4):585-94.

78. van Eijk AM, Ayisi JG, ter Kuile FO, Misore A, Otieno JA, Kolczak MS, et al. Human immunodeficiency virus seropositivity and malaria as risk factors for third-trimester anemia in asymptomatic pregnant women in western Kenya. *American Journal of Tropical Medicine & Hygiene* 2001;65(5):623-30.

79. Antelman G, Msamanga GI, Spiegelman D, Urassa EJ, Narh R, Hunter DJ, et al. Nutritional factors and infectious disease contribute to anemia among pregnant women with human immunodeficiency virus in Tanzania. *Journal of Nutrition* 2000;130(8):1950-7.

80. Nosten F, ter Kuile F, Maelankirri L, Decludt B, White NJ. Malaria during pregnancy in an area of unstable endemicity. *Transactions of the Royal Society of Tropical Medicine & Hygiene* 1991;85(4):424-9.

81. Villamor E, Msamanga G, Aboud S, Urassa W, Hunter DJ, Fawzi WW. Adverse perinatal outcomes of HIV-1-infected women in relation to malaria parasitemia in maternal and umbilical cord blood. *Am J Trop Med Hyg* 2005;73(4):694-7.

82. Dreyfuss ML, Msamanga GI, Spiegelman D, Hunter DJ, Urassa EJ, Hertzmark E, et al. Determinants of low birth weight among HIV-infected pregnant women in Tanzania. *Am J Clin Nutr* 2001;74(6):814-26.

83. Bloland PB, Wirima JJ, Steketee RW, Chilima B, Hightower A, Breman JG. Maternal HIV infection and infant mortality in Malawi: evidence for increased mortality due to placental malaria infection. *AIDS* 1995;9(7):721-6.

84. Ayisi JG, van Eijk AM, Newman RD, ter Kuile FO, Shi YP, Yang C, et al. Maternal malaria and perinatal HIV transmission, western Kenya. *Emerg Infect Dis* 2004;10(4):643-52.

85. Brahmbhatt H, Kigozi G, Wabwire-Mangen F, Serwadda D, Sewankambo N, Lutalo T, et al. The effects of placental malaria on mother-to-child HIV transmission in Rakai, Uganda. *Aids* 2003;17(17):2539-41.

86. Gallagher M, Malhotra I, Mungai PL, Wamachi AN, Kioko JM, Ouma JH, et al. The effects of maternal helminth and malaria infections on mother-to-child HIV transmission. *Aids* 2005;19(16):1849-55.

87. Inion I, Mwanyumba F, Gaillard P, Chohan V, Verhofstede C, Claeys P, et al. Placental malaria and perinatal transmission of human immunodeficiency virus type 1. *J Infect Dis* 2003;188(11):1675-8.

88. Kapiga SH, Bang H, Spiegelman D, Msamanga GI, Coley J, Hunter DJ, et al. Correlates of plasma HIV-1 RNA viral load among HIV-1-seropositive women in Dar es Salaam, Tanzania. *JAIDS Journal of Acquired Immune Deficiency Syndromes* 2002;30(3):316-23.

89. Mwapasa V, Rogerson SJ, Molyneux ME, Abrams ET, Kamwendo DD, Lema VM, et al. The effect of Plasmodium falciparum malaria on peripheral and placental HIV-1 RNA concentrations in pregnant Malawian women. *Aids* 2004;18(7):1051-9.

90. Morley D, Woodland M, Cuthbertson WFJ. Controlled trial of pyrimethamine in pregnant women in an African village. *British Medical Journal* 1964;1:667-668.

91. Ladner J, Leroy V, Simonon A, Karita E, Bogaerts J, De Clercq A, et al. HIV infection, malaria, and pregnancy: a prospective cohort study in Kigali, Rwanda. *American Journal of Tropical Medicine & Hygiene* 2002;66(1):56-60.

92. Diagne N, Rogier C, Sokhna CS, Tall A, Fontenille D, Roussilhon C, et al. Increased susceptibility to malaria during the early postpartum period. [see comments]. *New England Journal of Medicine* 2000;343(9):598-603.

93. Helitzer-Allen DL, Macheso A, Wirima J, Kendall C. Testing strategies to increase use of chloroquine chemoprophylaxis during pregnancy in Malawi. *Acta Tropica* 1994;58(3-4):255-66.

94. Steketee RW, Wirima JJ, Slutsker L, Heymann DL, Breman JG. The problem of malaria and malaria control in pregnancy in sub-Saharan Africa. *American Journal of Tropical Medicine & Hygiene* 1996;55(1 Suppl):2-7.

95. Spencer HC, Kaseje DC, Sempebwa EK, Huong AY, Roberts JM. Malaria chemoprophylaxis to pregnant women provided by community health workers in Saradidi, Kenya. II. Effect on parasitaemia and haemoglobin levels. *Annals of Tropical Medicine & Parasitology* 1987;81 Suppl 1:83-9.

96. Schultz LJ, Ettling M, Chitsulo L, Steketee RW, Nyasulu Y, Macheso A, et al. A nation-wide malaria knowledge, attitudes and practices survey in Malawi: objectives and methodology. *Tropical Medicine & Parasitology* 1994;45(1):54-6.

97. Schultz LJ, Steketee RW, Chitsulo L, Macheso A, Nyasulu Y, Ettling M. Malaria and childbearing women in Malawi: knowledge, attitudes and practices. *Tropical Medicine & Parasitology* 1994;45(1):65-9.

98. Nyirjesy P, Kavasya T, Axelrod P, Fischer PR. Malaria during pregnancy: neonatal morbidity and mortality and the efficacy of chloroquine chemoprophylaxis. *Clin Infect Dis* 1993;16(1):127-32.

99. Cot M, Roisin A, Barro D, Yada A, Verhave JP, Carnevale P, et al. Effect of chloroquine chemoprophylaxis during pregnancy on birth weight: results of a randomized trial. *Am J Trop Med Hyg* 1992;46(1):21-7.

100. Schultz LJ, Steketee RW, Macheso A, Kazembe P, Chitsulo L, Wirima JJ. The efficacy of antimalarial regimens containing sulfadoxine-pyrimethamine and/or chloroquine in preventing peripheral and placental *Plasmodium falciparum* infection among pregnant women in Malawi. *American Journal of Tropical Medicine & Hygiene* 1994;51(5):515-22.

101. Schultz LJ, Steketee RW, Chitsulo L, Macheso A, Kazembe P, Wirima JJ. Evaluation of maternal practices, efficacy, and cost-effectiveness of alternative antimalarial regimens for use in pregnancy: chloroquine and sulfadoxine-pyrimethamine. *American Journal of Tropical Medicine & Hygiene* 1996;55(1 Suppl):87-94.

102. Schultz LJ, Steketee RW, Chitsulo L, Wirima JJ. Antimalarials during pregnancy: a cost-effectiveness analysis. *Bull World Health Organ* 1995;73(2):207-14.

103. Wolfe EB, Parise ME, Haddix AC, Nahlen BL, Ayisi JG, Misore A, et al. Cost-effectiveness of sulfadoxine-pyrimethamine for the prevention of malaria-associated low birth weight. *American Journal of Tropical Medicine & Hygiene* 2001;64(3-4):178-86.

104. Verhoeff FH, Brabin BJ, Chimsuku L, Kazembe P, Russell WB, Broadhead RL. An evaluation of the effects of intermittent sulfadoxine-pyrimethamine treatment in pregnancy on parasite clearance and risk of low birthweight in rural Malawi. *Annals of Tropical Medicine & Parasitology* 1998;92(2):141-50.

105. Hommerich L, von Oertzen C, Bedu-Addo G, Holmberg V, Acquah PA, Eggelte TA, et al. Decline of placental malaria in southern Ghana after the implementation of intermittent preventive treatment in pregnancy. *Malar J* 2007;6(1):144.

106. Ramharter M, Schuster K, Bouyou-Akotet MK, Adegnika AA, Schmits K, Mombo-Ngoma G, et al. Malaria in pregnancy before and after the implementation of a national IPTp program in Gabon. *Am J Trop Med Hyg* 2007;77(3):418-22.

107. Falade CO, Yusuf BO, Fadero FF, Mokuolu OA, Hamer DH, Salako LA. Intermittent preventive treatment with sulphadoxine-pyrimethamine is effective in preventing maternal and placental malaria in Ibadan, south-western Nigeria. *Malar J* 2007;6:88.

108. Tukur IU, Thacher TD, Sagay AS, Madaki JK. A comparison of sulfadoxine-pyrimethamine with chloroquine and pyrimethamine for prevention of malaria in pregnant Nigerian women. *Am J Trop Med Hyg* 2007;76(6):1019-23.

109. Sirima SB, Cotte AH, Konate A, Moran AC, Asamoa K, Bougouma EC, et al. Malaria prevention during pregnancy: assessing the disease burden one year after implementing a program of intermittent preventive treatment in Koupela District, Burkina Faso. *Am J Trop Med Hyg* 2006;75(2):205-11.

110. Morel CM, Lauer JA, Evans DB. Cost effectiveness analysis of strategies to combat malaria in developing countries. *Bmj* 2005;331(7528):1299.

111. Kayentao K, Kodio M, Newman RD, Maiga H, Doumtabe D, Ongoiba A, et al. Comparison of intermittent preventive treatment with chemoprophylaxis for the prevention of malaria during pregnancy in Mali. *J Infect Dis* 2005;191(1):109-16.

112. van Eijk AM, Ayisi JG, ter Kuile FO, Otieno JA, Misore AO, Odondi JO, et al. Effectiveness of intermittent preventive treatment with sulphadoxine-pyrimethamine for control of malaria in pregnancy in western Kenya: a hospital-based study. *Trop Med Int Health* 2004;9(3):351-60.

113. Shulman CE, Dorman EK, Cutts F, Kawuondo K, Bulmer JN, Peshu N, et al. Intermittent sulphadoxine-pyrimethamine to prevent severe anaemia secondary to malaria in pregnancy: a randomised placebo-controlled trial. *Lancet* 1999;353(9153):632-6.

114. Garner P, Brabin B. A review of randomized controlled trials of routine antimalarial drug prophylaxis during pregnancy in endemic malarious areas. *Bull World Health Organ* 1994;72(1):89-99.

115. Gamble C, Ekwaru PJ, Garner P, ter Kuile FO. Insecticide-treated nets for the prevention of malaria in pregnancy: a systematic review of randomised controlled trials. *PLoS Med* 2007;4(3):e107.

116. Njagi JK, Magnussen P, Estambale B, Ouma J, Mugo B. Prevention of anaemia in pregnancy using insecticide-treated bednets and sulfadoxine-pyrimethamine in a highly malarious area of Kenya: a randomized controlled trial. *Trans R Soc Trop Med Hyg* 2003;97(3):277-82.

117. Filler SJ, Kazembe P, Thigpen M, Macheso A, Parise ME, Newman RD, et al. Randomized Trial of 2-Dose versus Monthly Sulfadoxine-Pyrimethamine Intermittent Preventive Treatment for Malaria in HIV-Positive and HIV-Negative Pregnant Women in Malawi. *J Infect Dis* 2006;194(3):286-93.

118. Parise ME, Ayisi JG, Nahlen BL, Schultz LJ, Roberts JM, Misore A, et al. Efficacy of sulfadoxine-pyrimethamine for prevention of placental malaria in an area of Kenya with a high prevalence of malaria and human immunodeficiency virus infection. *American Journal of Tropical Medicine & Hygiene* 1998;59(5):813-22.

119. Hamer DH, Mwanakasale V, MacLeod WB, Chalwe V, Mukwamataba D, Champo D, et al. Two-Dose versus Monthly Intermittent Preventive Treatment of Malaria with Sulfadoxine-Pyrimethamine in HIV-Seropositive Pregnant Zambian Women. *J Infect Dis* 2007;196(11):000-000.

120. Kalilani L, Mofolo I, Chaponda M, Rogerson SJ, Alker AP, Kwiek JJ, et al. A randomized controlled pilot trial of azithromycin or artesunate added to sulfadoxine-pyrimethamine as treatment for malaria in pregnant women. *PLoS ONE* 2007;2(11):e1166.

121. Walter J, Mwiya M, Scott N, Kasonde P, Sinkala M, Kankasa C, et al. Reduction in preterm delivery and neonatal mortality after the introduction of antenatal cotrimoxazole prophylaxis among HIV-infected women with low CD4 cell counts. *J Infect Dis* 2006;194(11):1510-8.

122. Mermin J, Lule J, Ekwaru JP, Malamba S, Downing R, Ransom R, et al. Effect of co-trimoxazole prophylaxis on morbidity, mortality, CD4-cell count, and viral load in HIV infection in rural Uganda. *Lancet* 2004;364(9443):1428-34.

123. Watera C, Todd J, Muwonge R, Whitworth J, Nakiyingi-Miiro J, Brink A, et al. Feasibility and effectiveness of cotrimoxazole prophylaxis for HIV-1-infected adults attending an HIV/AIDS clinic in Uganda. *J Acquir Immune Defic Syndr* 2006;42(3):373-8.

124. Anglaret X, Chene G, Attia A, Toure S, Lafont S, Combe P, et al. Early chemoprophylaxis with trimethoprim-sulphamethoxazole for HIV-1-infected adults in Abidjan, Cote d'Ivoire: a randomised trial. Cotrimo-CI Study Group. *Lancet* 1999;353(9163):1463-8.

125. Wiktor SZ, Sassan-Morokro M, Grant AD, Abouya L, Karon JM, Maurice C, et al. Efficacy of trimethoprim-sulphamethoxazole prophylaxis to decrease morbidity and mortality in HIV-1-infected patients with tuberculosis in Abidjan, Cote d'Ivoire: a randomised controlled trial. *Lancet* 1999;353(9163):1469-75.

126. Badri M, Ehrlich R, Wood R, Maartens G. Initiating co-trimoxazole prophylaxis in HIV-infected patients in Africa: an evaluation of the provisional WHO/UNAIDS recommendations. *Aids* 2001;15(9):1143-8.

127. Yazdanpanah Y, Losina E, Anglaret X, Goldie SJ, Walensky RP, Weinstein MC, et al. Clinical impact and cost-effectiveness of co-trimoxazole prophylaxis in patients with HIV/AIDS in Cote d'Ivoire: a trial-based analysis. *Aids* 2005;19(12):1299-308.

128. Policy guidelines for prevention of mother-to-child transmission of HIV: Republic of Uganda Ministry of Health, 2006.

129. WHO. Guidelines for the treatment of malaria, 2006.

130. Watkins WM, Sixsmith DG, Chulay JD, Spencer HC. Antagonism of sulfadoxine and pyrimethamine antimalarial activity in vitro by p-aminobenzoic acid, p-aminobenzoylglutamic acid and folic acid. *Molecular & Biochemical Parasitology* 1985;14(1):55-61.

131. Safrin S, Lee BL, Sande MA. Adjunctive folinic acid with trimethoprim-sulfamethoxazole for Pneumocystis carinii pneumonia in AIDS patients is associated with an increased risk of therapeutic failure and death. *J Infect Dis* 1994;170(4):912-7.

132. Forna F, McConnell M, Kitabire FN, Homsy J, Brooks JT, Mermin J, et al. Systematic review of the safety of trimethoprim-sulfamethoxazole for prophylaxis in HIV-infected pregnant women: implications for resource-limited settings. *AIDS Rev* 2006;8(1):24-36.

133. Parikh S, Gut J, Istvan E, Goldberg DE, Havlir DV, Rosenthal PJ. Antimalarial activity of human immunodeficiency virus type 1 protease inhibitors. *Antimicrob Agents Chemother* 2005;49(7):2983-5.

134. Nathoo S, Serghides L, Kain KC. Effect of HIV-1 antiretroviral drugs on cytoadherence and phagocytic clearance of Plasmodium falciparum-parasitised erythrocytes. *Lancet* 2003;362(9389):1039-41.

135. Redmond AM, Skinner-Adams T, Andrews KT, Gardiner DL, Ray J, Kelly M, et al. Antimalarial activity of sera from subjects taking HIV protease inhibitors. *Aids* 2007;21(6):763-5.

136. Andrews KT, Fairlie DP, Madala PK, Ray J, Wyatt DM, Hilton PM, et al. Potencies of human immunodeficiency virus protease inhibitors in vitro against Plasmodium falciparum and in vivo against murine malaria. *Antimicrob Agents Chemother* 2006;50(2):639-48.

137. Skinner-Adams TS, Andrews KT, Melville L, McCarthy J, Gardiner DL. Synergistic interactions of the antiretroviral protease inhibitors saquinavir and ritonavir with chloroquine and mefloquine against Plasmodium falciparum in vitro. *Antimicrob Agents Chemother* 2007;51(2):759-62.

138. Skinner-Adams TS, McCarthy JS, Gardiner DL, Hilton PM, Andrews KT. Antiretrovirals as antimalarial agents. *J Infect Dis* 2004;190(11):1998-2000.

139. Kaletra in Pregnancy: A Retrospective Study of 104 Women. 13th British HIV Association Conference; 2007; Edinburgh, UK 23 - 25 April 2007.

140. Group. PHGW. Public Health Service Task Force Recommendations for Use of Antiretroviral Drugs in Pregnant HIV-1 Infected Women for Maternal Health and Interventions to Reduce Perinatal HIV-1 Transmission in the United States. October 12, 2006 1-65.

141. Association BH. Guidelines for the management of HIV infection in pregnant women and the prevention of mother-to-child transmission of HIV, 2005.

142. Rapid advice: use of antiretroviral drugs for treating pregnant women and preventing HIV infection in infants -- November 2009, 2009.

143. Tuomala RE, Shapiro DE, Mofenson LM, Bryson Y, Culnane M, Hughes MD, et al. Antiretroviral therapy during pregnancy and the risk of an adverse outcome. *N Engl J Med* 2002;346(24):1863-70.

144. Cotter AM, Garcia AG, Duthely ML, Luke B, O'Sullivan MJ. Is antiretroviral therapy during pregnancy associated with an increased risk of preterm delivery, low birth weight, or stillbirth? *J Infect Dis* 2006;193(9):1195-201.

145. Combination antiretroviral therapy and duration of pregnancy. *Aids* 2000;14(18):2913-20.

146. Thorne C, Patel D, Newell ML. Increased risk of adverse pregnancy outcomes in HIV-infected women treated with highly active antiretroviral therapy in Europe. *Aids* 2004;18(17):2337-9.

147. Martin F, Taylor GP. Increased rates of preterm delivery are associated with the initiation of highly active antiretrovial therapy during pregnancy: a single-center cohort study. *J Infect Dis* 2007;196(4):558-61.

148. Boer K, Nellen JF, Patel D, Timmermans S, Tempelman C, Wibaut M, et al. The AmRo study: pregnancy outcome in HIV-1-infected women under effective highly active antiretroviral therapy and a policy of vaginal delivery. *Bjog* 2007;114(2):148-55.

149. Townsend CL, Cortina-Borja M, Peckham CS, Tookey PA. Antiretroviral therapy and premature delivery in diagnosed HIV-infected women in the United Kingdom and Ireland. *Aids* 2007;21(8):1019-26.

150. Szyld EG, Warley EM, Freimanis L, Gonin R, Cahn PE, Calvet GA, et al. Maternal antiretroviral drugs during pregnancy and infant low birth weight and preterm birth. *Aids* 2006;20(18):2345-53.

151. Antepartum antiretroviral therapy and pregnancy outcomes in 462 HIV-infected women in 1998-1999 (PACTG 367). (abstract no. 664). 7th Conference of Retroviruses and Opportunistic Infections; 2000.

152. Tuomala RE, Watts DH, Li D, Vajaranant M, Pitt J, Hammill H, et al. Improved obstetric outcomes and few maternal toxicities are associated with antiretroviral therapy, including highly active antiretroviral therapy during pregnancy. *J Acquir Immune Defic Syndr* 2005;38(4):449-73.

153. Schulte J, Dominguez K, Sukalac T, Bohannon B, Fowler MG. Declines in low birth weight and preterm birth among infants who were born to HIV-infected women during an era of increased use of maternal antiretroviral drugs: Pediatric Spectrum of HIV Disease, 1989-2004. *Pediatrics* 2007;119(4):e900-6.

154. Kourtis AP, Schmid CH, Jamieson DJ, Lau J. Use of antiretroviral therapy in pregnant HIV-infected women and the risk of premature delivery: a meta-analysis. *Aids* 2007;21(5):607-15.

155. Hitti J, Andersen J, McComsey G, Liu T, Melvin A, Smith L, et al. Protease inhibitor-based antiretroviral therapy and glucose tolerance in pregnancy: AIDS Clinical Trials Group A5084. *Am J Obstet Gynecol* 2007;196(4):331 e1-7.

156. Wimalasundera RC, Larbalestier N, Smith JH, de Ruiter A, Mc GTSA, Hughes AD, et al. Pre-eclampsia, antiretroviral therapy, and immune reconstitution. *Lancet* 2002;360(9340):1152-4.

157. Suy A, Martinez E, Coll O, Lonca M, Palacio M, de Lazzari E, et al. Increased risk of pre-eclampsia and fetal death in HIV-infected pregnant women receiving highly active antiretroviral therapy. *Aids* 2006;20(1):59-66.

158. Frank KA, Buchmann EJ, Schackis RC. Does human immunodeficiency virus infection protect against preeclampsia-eclampsia? *Obstet Gynecol* 2004;104(2):238-42.

159. Bodkin C, Klopper H, Langley G. A comparison of HIV positive and negative pregnant women at a public sector hospital in South Africa. *J Clin Nurs* 2006;15(6):735-41.

160. Mattar R, Amed AM, Lindsey PC, Sass N, Daher S. Preeclampsia and HIV infection. *Eur J Obstet Gynecol Reprod Biol* 2004;117(2):240-1.

161. de Groot MR, Corporaal LJ, Cronjé HS, Joubert G. HIV infection in critically ill obstetrical patients. *International Journal of Gynecology & Obstetrics* 2003;81(1):9-16.

162. WHO. WHO HIV and Infant Feeding Technical Consultation -- Consensus Statement, Geneva, October 25-27, 2006, 2006.

163. Rapid advice: revised WHO principles and recommendations on infant feeding in the context of HIV – November 2009, 2009.

164. Palombi L, Marazzi MC, Voetberg A, Magid NA. Treatment acceleration program and the experience of the DREAM program in prevention of mother-to-child transmission of HIV. *Aids* 2007;21 Suppl 4:S65-71.

165. Prevention of mother-to-child transmission of HIV-1 through breastfeeding by treating mothers prophylactically with triple antiretroviral therapy in Dar es Salaam, Tanzania - the MITRA PLUS study. International AIDS Society; 2007; Syndey, Australia.

166. Schneider S, Peltier A, Gras A, Arendt V, Karasi-Omes C, Mujawamariwa A, et al. Efavirenz in human breast milk, mothers', and newborns' plasma. *J Acquir Immune Defic Syndr* 2008;48(4):450-4.

167. Shapiro RatMBST. The Mma Bana Study. A Randomized Trial Comparing Highly Active Antiretroviral Therapy Regimens for Virologic Efficacy and the Prevention of Mother-to-Child HIV Transmission among Breastfeeding Women in Botswana; Abstract WELBB101. *International AIDS Society*. Cape Town, 2009.

168. Sustiva [package insert]. Bristol-Myers Squibb Company. Princeton, NJ, 2007.

169. Effect of efavirenz on the pharmacokinetics of ethinyl estradiol and norgestimate in healthy female subjects [abstract A-958]. 48th ICAAC/IDSA Annual Meeting 2008; Washington, D.C.

170. Kaletra [package insert]. Abbott Laboratories. North Chicago, IL, 2005.

171. Cohn SE, Park JG, Watts DH, Stek A, Hitti J, Clax PA, et al. Depo-medroxyprogesterone in women on antiretroviral therapy: effective contraception and lack of clinically significant interactions. *Clin Pharmacol Ther* 2007;81(2):222-7.

172. Watts DH, Park JG, Cohn SE, Yu S, Hitti J, Stek A, et al. Safety and tolerability of depot medroxyprogesterone acetate among HIV-infected women on antiretroviral therapy: ACTG A5093. *Contraception* 2008;77(2):84-90.

173. Stek AM, Mirochnick M, Capparelli E, Best BM, Hu C, Burchett SK, et al. Reduced lopinavir exposure during pregnancy. *Aids* 2006;20(15):1931-9.

174. Baroncelli S, Villani P, Floridia M, Pirillo MF, Galluzzo CM, Cusato M, et al. Trough Concentrations of Lopinavir, Nelfinavir, and Nevirapine With Standard Dosing in Human Immunodeficiency Virus-Infected Pregnant Women Receiving 3-Drug Combination Regimens. *Ther Drug Monit* 2008.

175. Beumer JH, Bosman IJ, Maes RA. Hair as a biological specimen for therapeutic drug monitoring. *Int J Clin Pract* 2001;55(6):353-7.

176. Berg KM, Arnsten JH. Practical and conceptual challenges in measuring antiretroviral adherence. *J Acquir Immune Defic Syndr* 2006;43 Suppl 1:S79-87.

177. Back D, Khoo S, Gibbons S, Merry C. The role of therapeutic drug monitoring in treatment of HIV infection. *Br J Clin Pharmacol* 2001;52:89S-96S.

178. Marzolini C, Buclin T, Decosterd L, Biollaz J, Telenti A. Nelfinavir plasma levels under twice-daily and three-times-daily regimens: high interpatient and low intrapatient variability. *Ther Drug Monit* 2001;23:394-398.

179. Stahle L, Moberg L, Svensson J-O, Sonnerborg A. Efavirenz plasma concentrations in HIV-infected patients: inter- and intraindividual variability and clinical effects. *Ther Drug Monit* 2004;26:267-270.

180. Clevenbergh P, Boulme R, Kirstetter M, Dellamonica P. Efficacy, safety and predictive factors of virological success of a boosted amprenavir-based salvage regimen in heavily antiretroviral-experienced HIV-1-infected patients. *HIV Med* 2004;5(4):284-8.

181. Clevenbergh P, Garaffo R, Durant J, Dellamonica P. PharmAdapt: a randomized prospective study to evaluate the benefit of therapeutic monitoring of protease inhibitors: 12 week results. *AIDS* 2002;16:2311-2315.

182. Wertheimer BZ, Freedberg KA, Walensky RP, Yazdanapah Y, Losina E. Therapeutic drug monitoring in HIV treatment: a literature review. *HIV Clin Trials* 2006;7(2):59-69.

183. Demeter L, Jiang H, Mukherjee L, Morse G, DiFrancesco R, Klingman K, et al. A Prospective, Randomized, Controlled, Open-label Trial Evaluating the Effect of Therapeutic Drug Monitoring and Protease Inhibitor Dose Escalation on Viral Load Responses in Antiretroviral-experienced, HIV-infected Patients with a Normalized Inhibitory Quotient. *15th Conference on Retroviruses and Opportunistic Infections, February 3-6, Boston, MA* 2008.

184. Nettles RE, Kieffer TL, Parsons T, Johnson J, Cofrancesco J, Jr., Gallant JE, et al. Marked intraindividual variability in antiretroviral concentrations may limit the utility of therapeutic drug monitoring. *Clin Infect Dis* 2006;42(8):1189-96.

185. Podsadecki T, Vrijens B, Touseet E, Rode R, Hanna G. White Coat Compliance Patterns Make Therapeutic Drug Monitoring (TDM) a Potentially Unreliable Tool for Assessing Long-Term Drug Exposure. *13th Conference on Retroviruses and Opportunistic Infections, Denver, February 5-8* 2006.

186. Pepin G, Gaillard Y. Concordance between self-reported drug use and findings in hair about cocaine and heroin. *Forensic Sci Int* 1997;84(1-3):37-41.

187. Nakahara Y. Hair analysis for abused and therapeutic drugs. *Journal of Chromatography. B, Biomedical Sciences and Applications* 1999;733(1-2):161-80.

188. Tsatsakis AM, Psillakis T, Paritsis N. Phenytoin concentration in head hair sections: a method to evaluate the history of drug use. *Journal of Clinical Psychopharmacology* 2000;20(5):560-73.

189. Bernard L, Vuagnat A, Peytavin G, Hallouin MC, Bouhour D, Nguyen TH, et al. Relationship between levels of indinavir in hair and virologic response to highly active antiretroviral therapy. *Annals of Internal Medicine* 2002;137(8):656-9.

190. Bernard L, Peytavin G, Vuagnat A, de Truchis P, Perronne C. Indinavir concentrations in hair from patients receiving highly active antiretroviral therapy. *Lancet* 1998;352:1757-1758.

191. Servais J, Peytavin G, Arendt V, Staub T, Schneider F, Hemmer R, et al. Indinavir hair concentration in highly active antiretroviral therapy-treated patients: association with viral load and drug resistance. *Aids* 2001;15(7):941-3.

192. Duval X, Peytavin G, Breton G, Ecobichon JL, Descamps D, Thabut G, et al. Hair versus plasma concentrations as indicator of indinavir exposure in HIV-1-infected patients treated with indinavir/ritonavir combination. *Aids* 2007;21(1):106-8.

193. Huang Y, Gandhi M, Greenblatt RM, Gee W, Lin ET, Messenkoff N. Sensitive analysis of anti-HIV drugs, efavirenz, lopinavir and ritonavir, in human hair by liquid chromatography coupled with tandem mass spectrometry. *Rapid Commun Mass Spectrom* 2008;22(21):3401-9.

194. Gandhi M, Ameli N, Bacchetti P, Huang Y, Anastos K, Cohen M, et al. Concentrations of Lopinavir and Ritonavir in Hair Are Strongly Correlated with Virologic Success (oral presentation). *14th Conference on Retroviruses and Opportunistic Infections, February 25-28, Los Angeles, CA* 2007.

195. Gandhi M, Ameli N, Bacchetti P, Gange SJ, Anastos K, Levine A, et al. Protease inhibitor levels in hair strongly predict virologic response to treatment. *AIDS* 2009;23(4):471-8.

196. Gandhi M AN, Bacchetti P, Huang Y, Gange SJ, Anastos K, Levine A, Cohen M, Young M, Greenblatt RM. Concentrations of Efavirenz in Hair Are Strongly Correlated with Virologic Response (abstract 692). *16th Conference on Retroviruses and Opportunistic Infections (CROI)* Montreal, Canada, 2009.

197. MOH. National Antiretroviral Treatment Guidelines for Adults, Adolescents, and Children

2nd Edition ed, 2008.

198. Watts DH. Teratogenicity risk of antiretroviral therapy in pregnancy. *Curr HIV/AIDS Rep* 2007;4(3):135-40.

199. Bussmann H, Wester CW, Wester CN, Lekoko B, Okezie O, Thomas AM, et al. Pregnancy rates and birth outcomes among women on efavirenz-containing highly active antiretroviral therapy in Botswana. *J Acquir Immune Defic Syndr* 2007;45(3):269-73.

200. Jeantils V, Khuong MA, Delassus JL, Honore P, Taverne B, Uzan M, et al. [Efavirenz (Sustiva) in pregnancy: a study about 12 HIV patients]. *Gynecol Obstet Fertil* 2006;34(7-8):593-6.

201. Saitoh A, Hull AD, Franklin P, Spector SA. Myelomeningocele in an infant with intrauterine exposure to efavirenz. *J Perinatol* 2005;25(8):555-6.

202. Mofenson LM. Efavirenz reclassified as FDA pregnancy category D. *AIDS Clin Care* 2005;17(2):17.

203. De Santis M, Carducci B, De Santis L, Cavaliere AF, Straface G. Periconceptional exposure to efavirenz and neural tube defects. *Arch Intern Med* 2002;162(3):355.

204. Fundaro C, Genovese O, Rendeli C, Tamburrini E, Salvaggio E. Myelomeningocele in a child with intrauterine exposure to efavirenz. *Aids* 2002;16(2):299-300.

205. Baker R. Safety alert issued on efavirenz (Sustiva). *Beta* 1998:6-7.

206. Public Health Service Task Force Recommendations for Use of Antiretroviral Drugs in Pregnant HIV-Infected Women for Maternal Health and Interventions to Reduce Perinatal HIV

Transmission in the United States, 2008.

207. Walmsley S BB, King M, Arribas J, Beall G, Ruane P, Johnson M, Johnson D, Lalonde R, Japour A, Brun S, Sun E. . Lopinavir-ritonavir versus nelfinavir for the initial treatment of HIV infection. . *N Engl J Med.* 2002;346((26)):2039-46.

208. Eron J Jr YP, Gathe J Jr, Estrada V, DeJesus E, Staszewski S, Lackey P, Katlama C, Young B, Yau L, Sutherland-Phillips D, Wannamaker P, Vavro C, Patel L, Yeo J, Shaefer M; KLEAN study team. . The KLEAN study of fosamprenavir-ritonavir versus lopinavir-ritonavir, each in combination with abacavir-lamivudine, for initial treatment of HIV infection over 48 weeks: a randomised non-inferiority trial. . *Lancet.* 2006;368((9534)):476-82

209. A prospective, randomized, phase III trial of NRTI-, PI-, and NNRTI-sparing regimens for initial treatment of HIV-1 infection: ACTG 5142. . In: Program and abstracts of the XVI International AIDS Conference 2006 August 13-18; Toronto.

210. Pollard RB, Tierney C, Havlir D, Tebas P, Fox L, Smeaton L, et al. A phase II randomized study of the virologic and immunologic effect of zidovudine + stavudine versus stavudine alone and zidovudine + lamivudine in patients with >300 CD4 cells who were antiretroviral naive (ACTG 298). *AIDS Res Hum Retroviruses* 2002;18(10):699-704.

211. Havlir DV, Tierney C, Friedland GH, Pollard RB, Smeaton L, Sommadossi JP, et al. In vivo antagonism with zidovudine plus stavudine combination therapy. *J Infect Dis* 2000;182(1):321-5.

212. Noguera A, Fortuny C, Munoz-Almagro C, Sanchez E, Vilaseca MA, Artuch R, et al. Hyperlactatemia in human immunodeficiency virus-uninfected infants who are exposed to antiretrovirals. *Pediatrics* 2004;114(5):e598-603.

213. Antiretroviral therapy, fat redistribution and hyperlipidaemia in HIV-infected children in Europe. *Aids* 2004;18(10):1443-51.

214. Mongelli M, Wilcox M, Gardosi J. Estimating the date of confinement: ultrasonographic biometry versus certain menstrual dates. *Am J Obstet Gynecol* 1996;174(1 Pt 1):278-81.

215. Hutagalung R, Paiphun L, Ashley EA, McGready R, Brockman A, Thwai KL, et al. A randomized trial of artemether-lumefantrine versus mefloquine-artesunate for the treatment of uncomplicated multi-drug resistant Plasmodium falciparum on the western border of Thailand. *Malar J* 2005;4:46.

216. McGready R, Brockman A, Cho T, Cho D, van Vugt M, Luxemburger C, et al. Randomized comparison of mefloquine-artesunate versus quinine in the treatment of multidrug-resistant falciparum malaria in pregnancy. *Trans R Soc Trop Med Hyg* 2000;94(6):689-93.

217. Makanga M, Premji Z, Falade C, Karbwang J, Mueller EA, Andriano K, et al. Efficacy and safety of the six-dose regimen of artemether-lumefantrine in pediatrics with uncomplicated Plasmodium falciparum malaria: a pooled analysis of individual patient data. *Am J Trop Med Hyg* 2006;74(6):991-8.

218. Meremikwu M, Alaribe A, Ejemot R, Oyo-Ita A, Ekenjoku J, Nwachukwu C, et al. Artemether-lumefantrine versus artesunate plus amodiaquine for treating uncomplicated childhood malaria in Nigeria: randomized controlled trial. *Malar J* 2006;5:43.

219. Sagara I, Dicko A, Djimde A, Guindo O, Kone M, Tolo Y, et al. A randomized trial of artesunate-sulfamethoxypyrazine-pyrimethamine versus artemether-lumefantrine for the treatment of uncomplicated Plasmodium falciparum malaria in Mali. *Am J Trop Med Hyg* 2006;75(4):630-6.

220. van den Broek NR, White SA, Flowers C, Cook JD, Letsky EA, Tanumihardjo SA, et al. Randomised trial of vitamin A supplementation in pregnant women in rural Malawi found to be anaemic on screening by HemoCue. *Bjog* 2006;113(5):569-76.

221. Dorsey G, Staedke S, Clark TD, Njama-Meya D, Nzarubara B, Maiteki-Sebuguzi C, et al. Combination therapy for uncomplicated falciparum malaria in Ugandan children: a randomized trial. *Jama* 2007;297(20):2210-9.

222. Faye B, Ndiaye JL, Ndiaye D, Dieng Y, Faye O, Gaye O. Efficacy and tolerability of four antimalarial combinations in the treatment of uncomplicated Plasmodium falciparum malaria in Senegal. *Malar J* 2007;6:80.

223. Greenhouse B, Myrick A, Dokomajilar C, Woo JM, Carlson EJ, Rosenthal PJ, et al. Validation of microsatellite markers for use in genotyping polyclonal Plasmodium falciparum infections. *Am J Trop Med Hyg* 2006;75(5):836-42.

224. Langston C, Kaplan C, Macpherson T, Manci E, Peevy K, Clark B, et al. Practice guideline for examination of the placenta: developed by the Placental Pathology Practice Guideline Development Task Force of the College of American Pathologists. *Arch Pathol Lab Med* 1997;121(5):449-76.

225. WHO. ADDENDUM TO 2006 WHO GUIDELINES ON ANTIRETROVIRAL THERAPY FOR HIV INFECTION IN ADULTS AND ADOLESCENTS.

226. Gandhi M, Ameli N, Bacchetti P, French A, Young M, Anastos K, et al. Protease Inhibitor Levels in Hair Samples Strongly Predict Virologic Responses to HIV Treatment. *(submitted, October 2008)* 2008.

227. National Policy Guidelines and Service Standards for Sexual and Reproductive Health and Rights. In: Health GoUMo, editor, 2006.

228. Coovadia HM, Rollins NC, Bland RM, Little K, Coutsoudis A, Bennish ML, et al. Mother-to-child transmission of HIV-1 infection during exclusive breastfeeding in the first 6 months of life: an intervention cohort study. *Lancet* 2007;369(9567):1107-16.

229. WHO. WHO HIV and Infant Feeding Technical Consultation: Consensus Statement. Geneva: Inter-agency Task Team on Prevention of HIV Infections in Pregnant Women, Mothers, and their Infants, 2006.

230. Egge-Jacobsen W, Unger M, Niemann CU, Baluom M, Hirai S, Benet LZ, et al. Automated, fast, and sensitive quantification of drugs in human plasma by LC/LC-MS: quantification of 6 protease inhibitors and 3 nonnucleoside transcriptase inhibitors. *Ther Drug Monit* 2004;26(5):546-62.

**APPENDICES**

**Appendix I. Schedule of Clinical and Laboratory Evaluations for Women**

| **Evaluations and Interventions** | **Screen Visit** | **Enrol Visit** | **Study trt initia-tion**** | **Antepartum visits in weeks**  **since study treatment initiation^20^** | | | | | | | | **Delivery** | **Postpartum (PP) visits in weeks since delivery** | | | | | | | | **After 24 wks postpartum to 52 wks** | | | **58 wk PP visit** | **Option-al 18 & 24 mth PP visit** |
| --- | --- | --- | --- | --- | --- | --- | --- | --- | --- | --- | --- | --- | --- | --- | --- | --- | --- | --- | --- | --- | --- | --- | --- | --- | --- |
|  |  |  |  | **4** | **8** | **12** | **16** | **20** | **24** | **28** | **30-34 GA^19^** |  | **2** | **4** | **6** | **8** | **12** | **16** | **20** | **24** | **Q4 wks** | **Q12 wks** | **Q24 wks** |  |  |
| Screening consent | X |  |  |  |  |  |  |  |  |  |  |  |  |  |  |  |  |  |  |  |  |  |  |  |  |
| Study consent |  | X |  |  |  |  |  |  |  |  |  |  |  |  |  |  |  |  |  |  |  |  |  |  |  |
| Future use of specimen consent |  | X |  |  |  |  |  |  |  |  |  |  |  |  |  |  |  |  |  |  |  |  |  |  |  |
| HIV documentation^1^ | X |  |  |  |  |  |  |  |  |  |  |  |  |  |  |  |  |  |  |  |  |  |  |  |  |
| HIV & ART counseling | X | X | X |  |  |  |  |  |  |  |  |  | X |  |  |  |  |  |  |  |  |  |  |  |  |
| History^2^ |  | X |  |  |  |  |  |  |  |  |  |  |  |  |  |  |  |  |  |  |  |  |  |  | X |
| Pregnancy confirmation^3^ | X |  |  |  |  |  |  |  |  |  |  |  |  |  |  | X | X | X | X | X | X | X |  |  |  |
| Food insecurity /sanitation/hygiene questionnaire ^23^ |  |  |  | X | | | | | | | | | | | | | | | | | | | |  |  |
| Targeted physical exam^4^ |  |  |  | X | X | X | X | X | X | X | X | X | X | X |  | X | X | X | X | X | X | X |  |  | X |
| Complete physical exam |  | X |  |  |  |  |  |  |  |  |  |  |  |  | X |  |  |  |  |  |  |  |  |  |  |
| Obstetrical ultrasound^5^ | X |  |  |  |  |  |  |  |  |  |  |  |  |  |  |  |  |  |  |  |  |  |  |  |  |
| Antiretroviral therapy dispensed^6^ |  |  | X | X | X | X | X | X | X | X | X |  | X | X | X | X | X | X | X | X | X |  |  |  |  |
| Antiretroviral pregnancy registry^7^ |  | X |  |  |  |  |  |  |  |  |  |  |  |  | X |  |  |  |  |  |  |  |  |  |  |
| Adherence Questionnaires |  |  |  | X | X | X | X | X | X | X | X |  | X | X | X | X | X | X | X | X | X | X |  | X | X |
| Labor and Delivery Abstraction^8^ |  |  |  |  |  |  |  |  |  |  |  | X |  |  |  |  |  |  |  |  |  |  |  |  |  |
| **STANDARD PRENATAL CARE** |  |  |  |  |  |  |  |  |  |  |  |  |  |  |  |  |  |  |  |  |  |  |  |  |  |
| Obstetrical exam^9^ |  | X | X | X | X | X | X | X | X | X | X | X |  |  |  |  |  |  |  |  |  |  |  |  |  |
| Syphilis screen^ |  | X |  |  |  |  |  |  |  |  |  |  |  |  |  |  |  |  |  |  |  |  |  |  |  |
| Gonorrhea & Chlamydia tests^ |  | X |  |  |  |  |  |  |  |  |  |  |  |  |  |  |  |  |  |  |  |  |  |  |  |
| Iron + Folic acid |  | X |  | X | X | X | X | X | X | X | X |  | X | X | X | X | X | X | X | X |  |  |  |  |  |
| Pre-natal multivitamins |  | X |  |  |  |  |  |  |  |  |  |  | X |  |  |  |  |  |  |  |  |  |  |  |  |
| Mebendezole^10^ |  | X |  |  |  |  |  |  |  |  |  |  |  |  |  |  |  |  |  |  |  |  |  |  |  |
| Trimethoprim-sulfamethoxazole^11^ |  | X |  | X | X | X | X | X | X | X | X |  | X | X | X | X | X | X | X | X | X | X |  |  |  |
| Vitamin A^12^ |  |  |  |  |  |  |  |  |  |  |  | X |  |  |  |  |  |  |  |  |  |  |  |  |  |
| Insecticide-treated net^13^ |  | X |  |  |  |  |  |  |  |  |  |  |  |  |  |  |  |  |  |  |  |  |  |  |  |
| **LABORATORY EVALUATIONS** |  |  |  |  |  |  |  |  |  |  |  |  |  |  |  |  |  |  |  |  |  |  |  |  |  |
| CBC (2mL) | X | X* |  |  | X |  | X |  | X |  |  | X | X |  |  |  | X |  |  | X |  |  | X |  | X |
| ALT (2mL) | X | X* |  |  | X |  |  |  | X |  |  | X |  |  |  | X |  |  |  | X |  |  | X |  |  |
| Serum creatinine ^21^, bilirubin | X | X* |  |  |  |  |  |  |  |  |  |  |  |  |  |  |  |  |  |  |  |  |  |  |  |
| Urine pregnancy testing | X^3^ |  |  |  |  |  |  |  |  |  |  |  |  |  |  | X | X | X | X | X | X |  |  |  |  |
| Thick smear and filter paper ^14^ |  |  | X | X | X | X | X | X | X | X |  | X | X | X | X | X | X | X | X | X |  | X |  |  | X |
| **METABOLIC/ PHARMACOKINETIC** |  |  |  |  |  |  |  |  |  |  |  |  |  |  |  |  |  |  |  |  |  |  |  |  |  |
| Hair and plasma collection (5mL)^16~~5~~^ |  |  |  |  |  |  |  |  |  |  | X |  |  |  |  |  | X |  |  |  |  |  |  |  |  |
| Fasting cholesterol, triglycerides, glucose, insulin^1~~4~~5^ (2mL) |  |  | X |  |  |  |  |  |  |  | X |  |  |  |  |  |  |  |  |  |  |  |  |  |  |
| **VIROLOGY** |  |  |  |  |  |  |  |  |  |  |  |  |  |  |  |  |  |  |  |  |  |  |  |  |  |
| HIV RNA RT-PCR (5mL) ^22^ | X |  | X | X | X | X |  | X |  | X |  | X |  |  |  | X |  | X |  | X |  |  | X | X | X |
| IMMUNOLOGY |  |  |  |  |  |  |  |  |  |  |  |  |  |  |  |  |  |  |  |  |  |  |  |  |  |
| CD4/CD8 lymphocyte subsets (1mL) | X |  |  |  |  | X |  |  | X |  |  | X |  |  |  |  |  |  |  | X |  |  | X |  | X |
| **DELIVERY SPECIMEN COLLECTION** |  |  |  |  |  |  |  |  |  |  |  |  |  |  |  |  |  |  |  |  |  |  |  |  |  |
| Cord blood collection |  |  |  |  |  |  |  |  |  |  |  | X |  |  |  |  |  |  |  |  |  |  |  |  |  |
| Placental blood collection ^17^ ^~~6~~^ (smear, RDT, filter paper) |  |  |  |  |  |  |  |  |  |  |  | X |  |  |  |  |  |  |  |  |  |  |  |  |  |
| Placenta biopsy |  |  |  |  |  |  |  |  |  |  |  | X |  |  |  |  |  |  |  |  |  |  |  |  |  |
| Stored whole blood^18^ (1mL) |  |  | X | X | X | X | X | X | X | X |  | X |  |  |  |  |  |  |  |  |  |  | X |  | X |
| Stored plasma ^18^ (5mL) |  |  | X | X | X | X | X | X | X | X |  | X | X | X | X | X | X | X | X | X |  |  | X |  | X |
| **TOTAL VOLUME (mL)** | **6** | **0-6*** | **15** | **14** | **14** | **13** | **9** | **12** | **10** | **12** | **4** | **15** | **7** | **9** | **7** | **14** | **13** | **9** | **7** | **16** | **0** | **0** | **16** | **5** | **12** |

* Labs done at enrollment visit only if this visit is greater than 2 weeks since screening visit; ^ Tests done at enrollment when available **M**ay be same day as Enroll visit**

**Explanation of maternal schedule of events (Appendix I)**:

1. HIV-1 will be documented with a positive rapid HIV test plus confirmatory Western Blot or HIV RNA.
2. History will include SES indicators, general medical history, diagnoses, medication (including ingestion of herbs), obstetrical history, STDs, allergies, and current symptoms.
3. Pregnancy confirmation by positive serum or urine pregnancy test, or confirmed intrauterine pregnancy by ultrasound. A pregnancy test may be skipped if an intrauterine pregnancy has been noted on ultrasound at the screening visit.
4. Targeted physical exam will include anthropometric measurements (e.g. weight and mid-upper arm circumference) and vital signs (i.e. temperature, pulse, blood pressure, respiratory rate). Measurement of height at the entry visit only.
5. Ultrasound will be done to confirm intrauterine pregnancy and estimate gestational age at enrollment. See Appendix V for dating criteria.
6. Antiretroviral therapy will be dispensed by the study pharmacist at every 4 week visit. A 5 week supply will be dispensed at each visit, with a home visit to ensure consistent supply of medication. Women will continue on antiretroviral medication for 1 week following the cessation of breastfeeding.
7. Information on all pregnant women enrolled will be sent anonymously to the Antiretroviral Pregnancy Registry: [www.apregistry.com](http://www.apregistry.com/). Information will be sent within 1 month of enrollment and follow-up data will be sent following delivery.
8. Labor & Delivery documentation will include: Peripartum history, mode of delivery, Apgar scores (when available), weight, length, and head circumference at birth, approximate gestational age, duration of labor, signs of fetal distress (presence of meconium), summary of events in first days of life (including feeding, breathing patterns, jaundice, lethargy, or any additional abnormal findings), duration of admission if delivered in hospital.
9. Obstetrical exam includes estimation of gestational age at study entry, fundal height measurement, fetal heart tones and urine dipstick for protein. A cervical exam will also be performed at screening and during antepartum study visits as clinically indicated.
10. Mebendazole is typically given as 200mg twice a day for 3 days or 500mg as a single dose as early as possible after the 1^st^ trimester.
11. Trimethoprim-sulfamethoxazole will be dispensed at each 4 week visit and dosed as 960 mg/day.
12. Vitamin A supplementation is dosed as 200,000 IU.
13. All women will receive an insecticide-treated net if not already received as part of routine prenatal care prior to study entry.
14. At each of the antepartum, delivery and postpartum visits, blood will be used to make thick smears and saved on filter paper. For women without symptoms of malaria, these specimens will be stored for evaluation at a later time.
15. These metabolic labs will be performed on stored samples and not run in real time. If women arrive for their study initiation visit and have not fasted overnight, they may still start on antiretroviral therapy. They may return for fasting labs within 7 days of initiating ARVs.
16. See Appendix XII. For the pharmacokinetic portion of the study, we will initially evaluate only a subset of hair and plasma ART levels in women and their infants, although samples will be collected on all of the study participants.
17. Placental Rapid Diagnostic Testing, placental blood smear and filter paper will be performed using a blood specimen obtained within 30 minutes of delivery.
18. Blood will be collected and stored for future evaluation. HIV genotyping may also be performed on stored samples from all or a subset of participants. These assays will not be performed in real time and therefore the results will not be used in clinical management.
19. All women will undergo a study visit between 30 and 34 weeks gestation (i.e. not 30-34 weeks since enrollment). This 30-34 week gestational age (GA) visit will be combined with the q4week antepartum study visit that occurs during this gestational age range. Because hair collection will occur at this visit, the visit should occur at least 4 weeks after the onset of ART.
20. If a woman presents to the study clinic for an unscheduled visit within 7 days of a subsequently scheduled routine study visit, this unscheduled visit may count as a routine study visit as long as all study activities are performed.
21. For women receiving tenofovir, creatinine will be checked at initiation of tenofovir and then every 12 weeks.
22. HIV RNA will be measured in real time at screening, antenatal week 8, delivery, postnatal week 8, 24, and every 24 weeks thereafter.
23. Women will also undergo an assessment of food insecurity and sanitation (see Appendix XIV) once during study participation. This may occur during a routine study visit, either antepartum or postpartum.

For insufficient blood draws, priorities are as follows:

1. Safety 2. Virology 3. Metabolic studies

**Appendix II. Schedule of Clinical and Laboratory Evaluations for Infants^8^**

| **Evaluations and Interventions** | **Delivery** | **2** | **6** | **8** | **10** | **12** | **14** | **16** | **24** | **36** | **48** | **58** | **After 52 wks (separ-ate consent)**  **18 and 24 months of life** |
| --- | --- | --- | --- | --- | --- | --- | --- | --- | --- | --- | --- | --- | --- |
| CLINICAL EVALUATIONS |  |  |  |  |  |  |  |  |  |  |  |  |  |
| History^1^ | X | X | X | X |  | X |  | X | X | X |  |  | X |
| Physical exam^2^ | X | X | X | X |  | X |  | X | X | X |  |  | X |
| Nutrition and development assessment |  |  |  |  |  |  |  |  |  |  |  |  | X |
| LABORATORY EVALUATIONS |  |  |  |  |  |  |  |  |  |  |  |  |  |
| HIV DNA PCR^3^ | X |  |  |  |  |  |  |  | X |  |  | X |  |
| Hematology | 1cc |  |  | 1cc |  |  |  |  | 1cc |  |  |  |  |
| ALT | 2cc |  |  | 2cc |  |  |  |  | 2cc |  |  |  |  |
| Hair collection^4^ | X |  |  |  |  | X |  |  | X |  |  |  |  |
| Plasma for ART levels | 2cc |  |  |  |  | 2cc |  |  | 2cc |  |  |  |  |
| Thick smear/Filter paper | X | X |  | X |  |  |  | X | X | X |  |  | X |
| INTERVENTIONS |  |  |  |  |  |  |  |  |  |  |  |  |  |
| Trimethoprim-sulfamethoxazole^5^ |  |  | X | X |  | X |  | X | X | X^5^ |  |  |  |
| Insecticide-treated net^6^ | X |  |  |  |  |  |  |  |  |  |  |  |  |
| Nevirapine prophylaxis^7^ | X | X | X |  |  |  |  |  |  |  |  |  |  |
| Routine immunizations |  |  | X |  | X |  | X |  | X | X* |  |  |  |
| STORED PLASMA | X | X |  | X |  |  |  | X | X | X |  |  | X |

***According to local immunization schedule**

**Explanation of infant schedule of events** **(Appendix II)**:

1. History will include peripartum history, mode of delivery, Apgar scores (when available), weight, length, and head circumference at birth, approximate gestational age, duration of labor, signs of fetal distress (presence of meconium), summary of events in first days of life (including feeding, breathing patterns, jaundice, lethargy, or any additional abnormal findings), duration of admission if delivered in hospital. Additional history will include feeding practices, symptoms, medical events since last visit, allergic reactions, medication update, and immunizations obtained since last visit. Additionally, study staff will administer an adherence questionnaire to the infant’s caregiver assessing adherence to NVP prophylaxis, TS prophylaxis, and insecticide-treated net. History will also include a verbal autopsy if the baby has died since the last visit.

2. Physical exam will include temperature and if requested by the study physician: pulse, blood pressure, and respiratory rate, assessment for congenital anomalies, lymphadenopathy, hepatomegaly, splenomegaly, infections of the ears, mouth, pharynx, or skin, and pulmonary, cardiac, neurologic, or skeletal abnormalities. Anthropometric measurements will be obtained at each study visit. A developmental exam will be performed at each study visit.

3. HIV DNA PCR: Whole blood will be collected on filter paper (Guthrie Cards). HIV positive status will be defined as 2 positive DNA PCR samples taken on 2 different dates. Any infant found to have a positive DNA PCR will return within 48 hours for repeat DNA-PCR and RNA-PCR testing. The final DNA PCR test will be done on the infant 6 weeks following weaning, but no later than 58 weeks of life, as weaning will be encouraged at 1 year of life. HIV DNA will be measured in real time at delivery and at weeks 24 and 58 of life.

4. See Appendix XII. For the pharmacokinetic portion of the study, we will initially evaluate only a subset of hair and plasma ART levels in infants, although samples will be collected on all of the study participants. Hair and plasma samples for ARV levels will be collected from infants at birth, 12-14 weeks and again at the 24 week visit.

5. Trimethoprim- sulfamethoxazole will be started at 6 weeks of age and continued until 6 weeks following the cessation of breastfeeding as per MOH guidelines. Infants will be dosed as 2.5cc of 40mg(trimethoprim)/200mg(sulfamethoxazole)/5ml suspension. Mothers/caregivers will receive a 5 week supply of TS from the pharmacy each month. Between 24 and 58 weeks of life, monthly dispensing of TS from the pharmacy will continue even though laboratory evaluations will occur every 12 weeks during this time period.

6. Insecticide-treated nets (ITN) will be provided to all women enrolled in the study. An additional ITN will be given following delivery for the infant if the mother and infant do not co-sleep.

7. Infants will be given NVP (10mg/ml) prophylaxis for the first 6 weeks of life as per updated Ugandan MOH guidelines:

1. birth weight 2.0-2.5 kg -> 1 ml once daily
2. birth weight >2.5kg -> 1.5 ml once daily

8. If an infant presents to the study clinic for an unscheduled visit within 7 days of a subsequently

scheduled routine study visit, this unscheduled visit may count as a routine study visit as long as all study activities are performed.

**Appendix III. Precautionary Medications**

The following agents must be used with caution for subjects receiving a protease inhibitor or an NNRTI and may require additional monitoring of drug levels or for adverse events. These PRECAUTIONARY MEDICATIONS include, but may not be limited to:

| **Agent by Class** | **Precautionary Concomitant Medications** |
| --- | --- |
|  |  |
| Analgesics | Codeine |
|  |  |
| Antiarrhythmics | disopyramide (Norpace)  lidocaine (Xylocaine)  mexiletine (Mexitil) |
|  |  |
| Antihistamines | loratidine (Claritin) |
|  |  |
| Anti-infectives | clindamycin (Cleocin)  dapsone  fluconazole (Diflucan)  miconazole (Monistat)  rifabutin (Mycobutin)* |
|  |  |
| Antipsychotic agents | risperidone (Risperdal) |
|  |  |
| Beta Blockers  (use caution with entire class) | atenolol (Tenormin)  bisopropol (Zebeta)  carvediol (Coreg)  esmolol (Brevibloc)  labetolol (Normodyne)  metoprolol (Lopressor and Toprol)  nadolol (Corgard)  pindolol (Visken)  propranolol (Inderal)  timolol (Blocadren) |
|  |  |
| Calcium Channel Blockers | amlodipine (Norvasc)  diltiazem (Cardiazem and others)  isradipine (Dynacirc)  nicardipine (Cardene)  nifedipine (Adalat and Procardia)  nimodipine (Nimotop)  nisoldipine (Sular)  verapamil (Calan and Isoptin) |
|  |  |
| HMG Co Reductase Inhibitors except pravastatin** | atorvastatin (Lipitor)  cerivastatin (Baycol)  fluvastatin (Lescol) |
| Hormonal Agents | estrogens  glucocorticoids  progesterones |
|  |  |
| Hypoglycemics | pioglitazone (Actos) |
|  |  |
| Psychiatric Medications | bupropion (Wellbutrin, Zyban)  clozapine (Clozaril)  fluoxetine (Prozac and others)  nefazadone (Serzone)  paroxetine (Paxil)  venlafexine (Effexor) |
|  |  |
| Tricyclic Antidepressants, including but not limited to: | amitriptyline (Elavil and others)  desipramine (Norpramin and others)  imipramine (Tofranil and others)  nortriptyline (Pamelor and others) |
|  |  |
| Sedative/Hypnotics | All benzodiazepines: ***  alprazolam (Xanax)  diazepam (Valium)  estazolam (ProSom)  flurazepam (Dalmane)  midazolam (Versed)****  oxazepam (Serax)  temazepam (Restoril)  buspirone (BuSpar)  zaleplon (Sonata)  zolpidem (Ambien) |
|  |  |
| Other | sildenafil (Viagra)  theophylline  warfarin (Coumadin)  Ergot alkaloids |

*The growing use of rifabutin for mycobacterial infections in subjects with HIV has warranted concern regarding optimum dosing. As the magnitude of the interactions with antiretrovirals is variable, dose adjustments are in turn drug-specific and not all dose modifications have been fully evaluated in HIV-infected individuals.

**Lovastatin (Mevacor) and simvastatin (Zocor) are prohibited.

***Triazolam (Halcion) is prohibited.

****Midazolam is allowed only as a single dose given under monitored conditions for procedures.

**Appendix IV.** **Patient Information Sheet**

**A STUDY ON PREVENTING MALARIA IN**

**HIV+ PREGNANT WOMEN**

Makerere University and the University of California, San Francisco in the United States are combining efforts in Tororo to study new ways of using HIV drugs to prevent malaria in HIV+ pregnant women and their babies.

- HIV drugs are recommended for the prevention of HIV among babies born to women with HIV. We want to study 2 different combinations of HIV drugs to see if they can be used to prevent malaria as well.
- Our study clinic is located at Tororo District Hospital next to the antenatal clinic and is open every day from 8:00 am to 5:00 pm
- We want to enroll women at least 16 years old and follow them during pregnancy. We will then follow the women for 12 months post-partum and their babies until 58 weeks of age.
- Women and babies in this study will receive free medical care
- We shall also give reimbursement for transport to and from our study clinic

For more information, please come to our study clinic where our doctors will be happy to talk to you and see if you and your baby can be in the study.

**Appendix V. Determination of Gestational Age**

Gestational age will be based on the first day of the last menstrual period (LMP) and the earliest available ultrasound performed at > 6 weeks gestation. The estimated due date (EDD) is calculated as 280 days following the LMP.

If the first available ultrasound is consistent with a gestational age of 6 to 12 weeks, and the ultrasound gestational age is within 7 days of that given LMP, then the LMP is used to determine gestational age. However, if the ultrasound gestational age differs from the LMP gestational age by more than 7 days, then the ultrasound is used to determine gestational age.

If the first available ultrasound is consistent with a gestational age of 13-24 weeks, and the ultrasound gestational age is within 14 days of that given by the LMP, then the LMP is used to determine gestational age. However, if the ultrasound gestational age differs from the LMP by more than 14 days, then the ultrasound is used to determine gestational age.

If the first available ultrasound is consistent with a gestational age of 25 weeks or more, and the ultrasound gestational age is within 21 days of that given by the LMP, then the LMP is used to determine gestational age. However, if the ultrasound gestational age differs from the LMP by more than 21 days, then the ultrasound is used to determine gestational age. Care here should be taken to rule out intrauterine growth restriction (IUGR).

If there is more than one ultrasound, and the results disagree, the earliest ultrasound performed at > 6 weeks should be used to determine gestational age.

If the LMP is not known, then the earliest ultrasound performed at > 6 weeks should be used to determine gestational age.

**Appendix VI. Informed Consent to be Screened for a Clinical Study**

**Protocol Title:** Protease Inhibitors to Reduce Malaria

Morbidity in HIV-Infected Pregnant Women

**Funding Source**: NIH/NICHD (NICHD P01 HD059454)

**UCSF-CHR Number**: H5741-34342

**FOM-REC Number:** 2009-141

**UNCST Number:** HS-670

**Site of Research**: Tororo District Hospital

Tororo, Uganda

**U.S. Principal Investigator**: Diane Havlir, MD

**Ugandan Principal Investigator**: Moses Kamya, MBChB, MMed, MPH, PhD

**Version Date**: August 18, 2010

**INTRODUCTION:**

You are being asked to participate in screening for a research study which will involve doing a urine pregnancy test and testing your blood for HIV and complete blood count, liver and kidney blood tests to decide whether participation in a research study would be appropriate for your care. This is a consent form which gives information about what is involved in the screening process for a research study. If it is decided that participation in a research study would be right for you, the study staff will talk more about the study in detail for you to decide. At this time you are not being asked to agree to be enrolled in a study, only for testing whether you are eligible for a research study. You can ask questions about this screening at any time. If you agree to be screened for a research study, we will ask you to sign this consent form. You will get a copy of this form to keep.

**PURPOSE:**

If you agree, we will withdraw about 1 tablespoon of blood drawn from a vein in your arm. This sample will be tested for HIV and a complete blood count, and liver and kidney blood tests. If your HIV test is found to be positive then you will be asked to give 2 teaspoons of blood for a repeat test to confirm this finding. If you have already been tested for HIV and found to be positive and brought your test results with you, we will still complete a repeat testing to confirm this finding. If your repeat test is proved to be positive for HIV, it means that you are a carrier of HIV. It also means that you can pass the virus to your baby and to others by intimate sexual contact, by sharing needles, and through donating blood. A negative HIV test means that at this time, no antibody to HIV was found in your blood sample based on the result of the initial screening test.

There can be individuals who have HIV test results that are called “false positive,” meaning for some reason, the test indicates that HIV is present in the blood when it is not. There can also be “false negative” results which can mean the person has been infected with HIV, but the test has failed to detect it.

You may also get a urine pregnancy test to confirm that you are pregnant. You may also get a cervical exam if there is any concern that you may be in labor. We will also do an ultrasound, which is a safe way of taking pictures of your baby, to estimate how far along you are in pregnancy. A home visitor will escort you home to record where you live.

You will be asked to return to the study clinic in 3 to 7 days when your test results are complete to learn about your laboratory and HIV test results. If your HIV test is found positive, you will also be counseled as to the risks for transmitting HIV to others including your baby, risks for developing AIDS, and the available treatments for HIV infection. You will be given a date to return to the clinic to receive results from the repeat test. You will be referred, if you choose, for proper medical care.

**BENEFITS OF THIS SCREENING:**

The benefit of screening for this study is that you will be tested for HIV infection and counseled regarding HIV infection at no cost to you. Also, you will be given the results of your other laboratory tests including a complete blood count and liver and kidney tests and referred for proper medical care if you or the study staff think it is needed.

**RISKS OF THIS SCREENING:**

The risks of screening for this study are minimal. The needle stick may hurt. There is a small risk of bruising, and a rare risk of infection. There may be emotional discomfort or stress associated with knowledge of the results of the HIV test. The ultrasound test is painless. There is no risk to you or to your baby.

**COSTS AND PAYMENTS:**

There will be no costs for participating in laboratory testing or HIV counseling. The blood tests will be free of charge. Also, you will receive no payment.

**CONFIDENTIALITY:**

Your name will not be recorded anywhere in the files for this screening where your laboratory and HIV test results will be kept. Any information obtained from this testing will be stored in locked files. You will not be identified in any publication. All information will be handled in compliance with U.S. and Uganda law on confidential information.

**RIGHT TO REFUSE:**

You do not have to take part in this testing. Counseling on these laboratory and HIV tests is available to you if you choose, before you make the decision to participate in this testing.

**COMPENSATION FOR INJURY:**

If you believe that the test procedures have resulted in an injury to you, immediately contact Dr. Kamya at Mulago Hospital (mobile: 0712-520469). If you have been injured or become ill, please contact the doctors at the study clinic. If you have questions about injuries as a result of being in the study, contact the doctors at the study clinic. You can get health care services at Tororo District Hospital in case of such injuries. If funds provided by the study sponsors to carry out this research project are available, you will not have to pay for care for study-related injuries.

**CONSENT: WHAT YOUR SIGNATURE OR THUMBPRINT MEANS**

PARTICIPATION IN RESEARCH IS VOLUNTARY. You have the right to decline to participate in the screening for this clinical study. If you are eligible for a clinical study, the study staff will discuss the study with you in more detail in a separate consent for you to decide if you would like to participant. A copy of this consent form will be given to you. Your signature or thumbprint below means that you have had this study explained to you. Your signature or thumbprint below means you have had the opportunity to ask questions. If you wish to participate in the screening process for a clinical study, you should sign or place your thumbprint below.

Name of Participant (printed)

Signature or Thumbprint * of Participant Date

Name of Study Staff Administering Consent (printed) Position/Title

Signature of Study Staff Administering Consent Date

Name of Translator (if necessary)

Signature of Translator Date

*If the participant is unable to read and/or write, an impartial witness must be present during the informed consent discussion. After the written informed consent form is read and explained to the participant, and after he or she has orally consented to participation in this screening, and has either signed the consent form or provided his or her fingerprint, the witness should sign and personally date the consent form. By signing the consent form, the witness attests that the information in the consent form and any other written information was accurately explained to, and apparently understood by, the participant, and that informed consent was freely given by the participant.

Name of Person Witnessing Consent (printed)

Signature of Person Witnessing Consent Date

**Appendix VII. Research Participation Informed Consent Form**

**Protocol Title: PROMOTE-PIs:** Protease Inhibitors to Reduce Malaria

Morbidity in HIV-Infected Pregnant Women

**Funding Source**: NIH/NICHD (NICHD P01 HD059454)

**UCSF-CHR Number**: 10-02958

**FOM-REC Number:** 2009-141

**UNCST Number:** HS-670

**Site of Research**: Tororo District Hospital

Tororo, Uganda

**U.S. Principal Investigator**: Diane Havlir, MD

**Ugandan Principal Investigator**: Moses Kamya, MBChB, MMed, MPH, PhD

**Version Date**: March 31, 2011

**INTRODUCTION**

You are being asked to participate in this research study because you are infected with the Human Immunodeficiency Virus (HIV) and are pregnant. This study is sponsored by the National Institute of Child Health and Human Development (NICHD) in the U.S. Other support comes from a pharmaceutical company, called Abbott Laboratories, which makes one of the study drugs. Before you decide if you want to participate in this study, we want you to know as much as possible about the study.

This is a consent form. It gives information about this study. The study staff is available to talk more about the study. You can ask questions about this study at any time. If you agree to participate during pregnancy and allow your baby to participate after delivery, we will ask you to sign the consent form. You will get a copy of this form to keep.

**WHY IS THIS STUDY BEING DONE?**

HIV-infected pregnant women are given anti-HIV drugs for their own health and/or to decrease the chances of passing HIV to the baby. This study will compare how well different combinations of anti-HIV medicines control the risk of malaria during pregnancy and breastfeeding. This study will also compare how well the anti-HIV medicines control HIV in pregnancy and after delivery, and whether these combinations of drugs work well in preventing HIV from being passed to your baby. Depending on your laboratory tests results done during screening for this study, you may not need to take these anti-HIV medications for your own health. If you were not pregnant, you might not need to take anti-HIV medicine at all. Since you are pregnant, anti-HIV medicines are necessary to decrease the risk of your child from getting the virus. By being part of this research study, you may receive combinations of anti-HIV medication that would not usually be available to you.

The combinations of anti-HIV drugs used in this study reduce the amount of HIV but have not been fully studied in pregnant women. This study will look at the safety of these drugs for women who take them during pregnancy and while breastfeeding. This study will also look at the safety of the study drugs for babies who will be exposed to the drugs during pregnancy or through breast milk.

All drugs used in this study are approved by the National Drug Authority (NDA) in Uganda and by the U.S. Food and Drug Administration (FDA) in the U.S. One drug, called Aluvia, which will be used in this study is investigational. It is a higher dose than what has been approved by the NDA in Uganda and by the U.S. FDA for people who are not pregnant. This higher dose of Aluvia is being looked at in this study and in several other studies because early studies showed that the approved lower doses may not be enough of the drug for pregnant women.

The anti-HIV drug combinations are:

- zidovudine (AZT), lamivudine ( 3TC), and lopinavir/ritonavir (LPV/RTV, also called Aluvia)

OR

- zidovudine (AZT), lamivudine ( 3TC), and efavirenz (EFV)

If you get severe anemia during this study, you will take the anti-HIV drug medication tenofovir (TDF) or stavudine (d4T) instead of zidovudine (AZT).

In this study, you will be required to stay on study drugs during pregnancy and for either 12 months after you have your baby or until 1 week after you stop breastfeeding, whichever comes first. Depending on your health, your health care provider may advise you to stay on anti-HIV drugs after you stop breastfeeding. If you continue any anti-HIV drugs after breastfeeding, that will not be done as part of this study and those drugs will not be provided by the study. We will refer you to a local medical provider who can continue anti-HIV drugs if you need them for your own health once you are done with the study.

**HOW MANY WOMEN WILL TAKE PART IN THIS STUDY?**

About 500 women will take part in this study. We will follow the babies born to all the women in the study.

**What will happen if i agree to participate in the study?**

If you agree to be in this study, the following will happen:

**1. Study Entry (today or another day of your choosing; the total time needed for this visit is about 2 hours):**

a. If you agree to join this study, study staff will review your health history and will give you a physical exam. Study staff will also do an obstetrical exam (measure the size of your uterus). If it has been more than 2 weeks since you were screened for this study, study staff will draw about 1 tablespoon of blood from a vein in your arm to make sure that it is safe for you to take the anti-HIV drugs. We will also test you for sexually transmitted infections by taking a vaginal or cervical swab. You will be told the results of the blood and sexually transmitted infection tests soon after they are available. We will give you treatment if you are found to have a sexually transmitted infection. If you are found to have a sexually transmitted infection, we will also help with telling your partner and will recommend and provide treatment for your partner. We will also store some of your blood to do tests later. One of these tests is HIV genotyping. HIV genotyping shows how well different anti-HIV drugs work against different kinds of HIV. Because we will not perform these tests right away, we will not be able to give you the results of this test. You will receive counseling today about the anti-HIV drugs and HIV infection and be given a date to return to the study clinic for more counseling if needed.

b. If the tests show it is safe for you to join the study and the study counselors have met with you about anti-HIV drugs, we will ask you to come to the study clinic “fasting” in order to prepare for some special additional blood tests. Fasting means that you may not eat or drink anything (except water, and medications you need to take with water) for 8 hours before you come to the clinic.

c. At this visit you will be put into a treatment group that has one of two different drug combinations. You cannot choose which combination you will take. You will be assigned by chance, like flipping a coin. You will have an equal chance of receiving either treatment. You and your doctors will know which drugs you will take.

You will be assigned to:

Group A: zidovudine, lamivudine and Aluvia. You will take 1 pill of zidovudine and lamivudine twice each day, and 2 pills of Aluvia twice a day. When you are 30 weeks pregnant, your dose of Aluvia will increase to 3 pills twice a day. After delivery, your dose of Aluvia will go back down to 2 pills twice a day.

OR

Group B: zidovudine, lamivudine and efavirenz. You will take zidovudine and lamivudine twice a day and efavirenz once a day.

If you develop severe anaemia, you will be given an anti-HIV medication called tenofovir (TDF) or stavudine (d4T) instead of zidovudine (AZT). Anaemia is a decrease in the number of red blood cells that may cause weakness, dizziness, tiredness, and shortness of breath.

d. We will give you a supply of the study drugs and ask that you start taking study drugs. On the day that you start your study drugs, you will be asked to complete a questionnaire about how you are feeling and about how you usually take medications. These questionnaires will take about 30 minutes. You will also have a complete physical examination and an obstetrical exam. We will draw about 2 teaspoons of blood for laboratory tests.

e. Your information will be entered into a registry (information list) that includes pregnant

women who have HIV and are taking anti-HIV drugs by using a number but not your name. Without using your name, information about your health will be sent to this registry during the study. This registry was set up to help health care providers learn about how to best take care of pregnant women who have HIV.

f. A home visitor will escort you home to record where you live.

**3. During Pregnancy (the total time needed for each visit is about 1 hour):**

- 1. You will be seen in the study clinic every 4 weeks until delivery. Depending on where you were in your pregnancy when you entered this study, you will also have a visit at 30-34 weeks of your pregnancy. At each visit, you will have a medical history, physical and obstetrical (pregnancy) exam. We will ask you questions about your medications and your health. Depending on the study visit, we will also draw about 1 – 6 teaspoons of blood to see how your body is responding to the study drugs. We will give you the results of these tests soon after the results are available. Some of this blood will be stored and, at a later date, we will measure the amount of your HIV viral load. Because we will be running this viral load test at a later date, we will not be able to give you these results. We will also ask to take a tiny sample of your hair (about 20 strands) twice during the study (once during pregnancy and once at about 12 weeks after delivery). This tiny amount of hair will be cut from the back of your head.
  2. At each study visit, the pharmacist will give you enough study drugs for about 5 weeks. If you miss a study visit, study staff will come to your home to do a study visit within 7 days after your missed appointment. It will be very important to not miss any study visits, because we want to check the health of all study participants very closely and make sure participants get all study drugs on time.
  3. When you are between 30 and 34 weeks into your pregnancy, we will ask you to you come to the study clinic in the morning after fasting for at least 8 hours overnight. We will draw 2-4 teaspoons of blood that will be stored for later testing that is a part of this study.

d. We ask that you to come to the study clinic if you have a fever or other illness, even if it happens at a time when a study visit is not scheduled. There will be someone at the study clinic every day from 8:00 am to 5:00 pm. The clinic will be open on weekends and holidays. If you become sick after 5:00 pm, please go to Tororo District Hospital. You should tell the hospital staff that you are a part of this study. We will give you a study card that tells the doctors some information about your participation in the study. You need to keep this card with you at all times or in a safe place. Make sure that you take it with you to the hospital whenever you get sick. If you choose to go to another clinic or hospital for medical care, we will not cause any problems for you, but we cannot guarantee you will be reimbursed for care given outside of the study.

**4. Labor and Delivery:**

a. We encourage all women participating in this study to deliver at Tororo District Hospital. When you are in the hospital to have your baby, study staff will get an update on your health, and you will have a physical and an obstetrical exam. About 6 teaspoons of blood will be drawn for laboratory testing. You will continue to take all your other study medicines by mouth on your usual schedule. Study staff will look at the medical records about your delivery and your baby’s health. We will also take samples of your placenta after delivery. If you are unable to come to Tororo District Hospital for your delivery, we will send a study staff member to your home to assist with collection of your placenta required for the study. If you deliver at home, we will encourage you and your baby to come to Tororo District Hospital on the day you deliver in order to check on you and your baby and do necessary tests for the study.

**5. After Delivery Visits – Mother (the total time needed for each visit is about 1 ½ hours):**

a. After delivery, you will continue to take your study drugs. You will be seen in the clinic at 2, 4, 6, 8, 12, 16, 20, and 24 weeks after delivery of your baby. These visits will include a health history update, physical exam and blood tests. After 24 weeks after delivery, you will come to the clinic every 4 weeks for pregnancy testing and an abbreviated health history, and to get your HIV medicine and every 3 months for a health history update, physical exam and blood tests. About 2 to 4 teaspoons of blood will be drawn at most visits. This blood will be used to find out about your overall health and to see how your body is responding to the HIV infection. You will be told the results of the blood tests after they are available. Some of your blood will be stored and used to perform tests later, including a test that will see if your HIV levels have changed as a result of taking study drugs. You will have a pregnancy test done each month after delivery and at any point after delivery if you, your medical provider or study staff think you may be pregnant.

**6. After Delivery Visits – Baby (the total time needed for each visit is about 1 ½ hours):**

a. After delivery, your baby will have a regular newborn physical examination. Within 72 hours of birth, about 2 teaspoons of blood will be drawn and tested for HIV infection and to find out if the anti-HIV drugs you took during pregnancy were safe for the baby. You will be given the results of your baby’s tests when they are available.

b. As soon as your baby is taking liquids by mouth, usually beginning 8-12 hours after delivery, he or she will receive nevirapine syrup by mouth. You will give nevirapine syrup to your baby once each day until the baby is 6 weeks old. This is standard therapy for babies who are born to mothers with HIV in Uganda.

c. Your baby will have physical exams in the clinic at 2, 6, 8, 12, 16, and 24 weeks after birth. Between 24 weeks and 1 year of life, you baby will have a physical exam every 3 months. Your baby will also have blood tests at delivery, 2, 8, 16 and 24 weeks of life. Between 24 weeks and 1 year of life, your baby will have blood tests every 3 months. Your baby will have a small amount of blood taken 6 weeks after you stop breastfeeding to do a final HIV test. About 1 to 1 and ½ teaspoons of blood will be drawn at each visit to check for HIV infection and the safety of anti-HIV drugs (the study drugs that you take during pregnancy and while breastfeeding and the zidovudine that the baby gets the first week after birth). Your baby will have a study visit at 10 and 14 weeks of life to receive standard vaccines. We will also ask for a small sample of the baby’s hair (about 20 strands) at birth, 12 weeks and 24 weeks of life. If your baby becomes infected with HIV, study staff will talk with you about options concerning treatment or enrollment of your baby into other anti-HIV drug studies if and when they become available.

**7. Additional Pregnancies:**

a. After your baby is born and while you are still in the study, we require you and your partner to use reliable birth control that you discuss with the study staff. One of the study medications (efavirenz) has been associated with serious back problems among babies exposed to efavirenz in the first trimester of pregnancy, including a problem called myelomeningocele (when part of the spinal cord is open to the air at birth). It is very important to avoid pregnancy if you take efavirenz. Because efavirenz can stay in your blood for awhile even after you have stopped taking the medicine, we recommend that you continue to use birth control for 3 months after stopping study drugs.

Some of the drugs in this study may make some birth control drugs that contain hormones (given by pills or placed under or on the skin) less effective. You cannot depend on hormone-type methods of birth control alone to prevent pregnancy.

Because of these different issues with the study drugs, in order to participate in this study, we require that after you deliver you:

1. obtain an intra-uterine device (IUD) or tubal ligation (permanent sterilization)

or

2. use 2 forms of birth control, with at least one form not containing hormones.

We will provide birth control to you or refer you locally to get birth control as part of being in this study.

b. If you think you may be pregnant again during this study, tell the study staff right away. They will talk to you about your choices. We will ask you to continue to be part of the study for 1 year after your initial delivery and to bring your baby for scheduled visits up to 58 weeks of life.

**8. Early Discontinuation from the Study:**

a. If you stop taking study anti-HIV drugs for any reason, you will be asked to continue to be followed on the study for 1 year after your delivery. You will also be asked to bring your baby for scheduled visits up to 24 weeks of age. If you decide not to return for these follow-up visits, you will be asked to come to the clinic with your baby for one final visit to answer some questions about your and your baby’s health, have laboratory blood tests and additional testing of the HIV virus in your blood (less than 4 teaspoons will be drawn). Some of your blood taken at this visit may be stored for future testing that is a part of this study. Your baby will have a physical exam and blood tests (about 2 teaspoons). Some of your baby’s blood taken at this visit may also be stored for future testing as a part of this study.

**9. Amount of Blood to be Collected Each Year**

a. Each year we expect to collect from you about 40-50 teaspoons of blood. While your baby is in the study, we expect to collect from him or her about 5-10 teaspoons of blood.

**HOW LONG WILL MY BABY AND I BE IN THIS STUDY?**

You will be in this study from the time you enter through pregnancy and delivery, and for 1 year after the birth of your baby. Your baby will be in this study until about 58 weeks of life (6 weeks after his or her 1^st^ birthday or 6 weeks after you stop breastfeeding, whichever is first). We recommend that you exclusively breastfeed your baby until the baby is 6 months (24 weeks) of age. After your baby is 6 months of age, we recommend that you begin giving your baby food and continue breastfeeding until your baby is 1 year old. We then recommend that your baby get tested for HIV 6 weeks after you wean from breastfeeding.

**WHY WOULD THE DOCTOR TAKE ME OR MY BABY OFF THIS STUDY EARLY?**

The study doctor may need to take you or your baby off the study early without your permission if:

- The study is cancelled by the UCSF or Makerere University, the Ugandan Ministry of Health, the Uganda National Council of Science and Technology, the U.S. National Institute of Child Health and Human Development (NICHD), or the U.S. Office for Human Research Protections (OHRP).
- A Data Safety Monitoring Board (DSMB) recommends that the study be stopped early. (A DSMB is an outside group of experts who monitor the study.)
- You or your baby are not able to attend the study visits as required by the study.
- Study participation is no longer in the best interest of you or your baby.

**CAN I STOP BEING IN THE STUDY?**

Yes. You can decide to stop at any time. Tell the study doctor or nurse if you are thinking about stopping or deciding to stop. He or she will tell you how to stop your participation safely. It is important that you tell the study doctor if you are thinking about stopping so that your doctor can evaluate any risks and discuss what alternative follow-up care and testing could be most helpful to you.

What side effects or risks can I expect me or my child to have from being in the study?

Risks to You: There are potential risks of each of these anti-HIV drug combinations when taken by pregnant and breastfeeding women.

- The study drugs might worsen some of the side effects of pregnancy, such as nausea or fatigue.
- Drugs you are given during this study might not work as well against HIV if you take them in the future.
- It is unknown if some of the rare side effects of pregnancy, such as diabetes, could worsen by taking the study anti-HIV drugs.
- It is unknown if stopping the HIV drugs during pregnancy (if you have side effects) increases the risk of HIV transmission to your unborn baby.

Risks to Your Baby: It is unknown whether the drugs used in this study can cause illness in your baby, but small studies done so far suggest that these drugs do not cause babies to get sick.

Non-study Medications and Other Clinical Studies: You must tell your doctor or study nurse about all other medicines and herbs that you are taking to make sure that your study drugs are safe to take. You must also tell your study nurse and doctor if you join any other research studies.

Risks of study drugs: The drugs used in this study may have side effects, some of which are listed below. These lists do not include all the side effects seen with these drugs. These lists include the more serious side effects or common side effects. If you have any questions about the drugs that you are taking as a part of this study, please ask the study doctors or nurses. Some side effects of drugs are “serious”. “Serious” means they might require hospitalization. They might also be long-term or never go away, life-threatening or fatal. Other side effects are “mild”.

- “Likely” side effects happen to more than half of the people who take a drug.
- “Frequent” side effects happen to less than half of people who take a drug.
- “Occasional” side effects happen to one out of 10 people at most who take a drug.
- “Rare” side effects happen to very few people who take a drug.

Risks of Nucleoside Analogues: Nucleoside analogues are a certain type of anti-HIV drug. Two of the study drugs, lamivudine and zidovudine, are nucleoside analogues. Lactic acidosis (elevated lactic acid levels in the blood) and severe hepatomegaly (enlarged liver) with steatosis (fatty liver) that may result in liver failure, other complications and death have been reported with the use of anti-HIV nucleoside analogues alone or in combination with other drugs. The liver complications and death have been seen more often in women on these drug regimens. Some symptoms that might indicate lactic acidosis include unexplained weight loss, stomach discomfort, nausea, vomiting, fatigue, weakness, and shortness of breath.

Risks of Lamivudine (3TC*)*: The following side effects have been found in HIV-infected individuals who take lamivudine:

Frequent serious side effects include:

- Decrease in the number of white blood cells that help fight infection

Frequent mild side effects include:

1. Headache • Feeling of vague overall discomfort
2. Feeling tired • Vomiting
3. Loose or watery stools (diarrhea) • Sleeplessness

Occasional serious side effects include:

1. Increase in a substance in the blood (amylase, a type of a pancreatic enzyme), which could mean a problem with the pancreas
2. Numbness tingling, and pain in the hands or feet (peripheral neuropathy)
3. Increased liver function levels, which could mean liver damage.
4. Depression

Occasional mild side effects include:

1. Dizziness • Upset stomach
2. Decrease in appetite • Abdominal cramps
3. Rash

The following serious side effect has been seen in children: inflammation of the pancreas with abdominal pain.

Zidovudine (ZDV): The following side effects have been found in HIV-infected individuals who take zidovudine:

Frequent serious side effects, include:

1. Decrease in the number of white blood cells that help fight infection
2. Decrease in the number of red blood cells, which may cause weakness, dizziness, and fatigue

Frequent mild side effects include:

1. Headache • Upset stomach
2. Vomiting • Decrease in appetite
3. Vague overall feeling of discomfort • Lack of energy
4. Feeling tired • Sleeplessness
5. Heartburn

Occasional mild side effects include:

1. Muscle aches, weakness, and decrease in the amount of muscle
2. Wasting
3. Darkening of the nails (with long-term use of zidovudine)

Rare serious side effects include:

1. Elevated liver enzymes (measurements of your liver function which may indicate possible liver damage)
2. Infiltration of the liver with fat (fatty liver which may result in liver failure, other complications and possibly death), fever, and rash

Emtricitabine/Tenofovir Disoproxil Fumarate (FTC/TDF)

No new or unexpected side effects are observed with the FTC 200 mg/TDF 300 mg combination tablet than those observed when each drug is given separately (see below).

Emtricitabine and tenofovir are nucleoside analogues or closely related drugs.

Emtricitabine/Tenofovir Disoproxil Fumarate (FTC/TDF):

No new or unexpected side effects are observed with the Emtricitabine 200 mg/Tenofovir 300 mg combination tablet than those observed when each drug is given separately (see below).

Emtricitabine (FTC): The following side effects have been associated with the use of emtricitabine:

Frequent side effects, which are not as serious, include:

• Tiredness, weakness                               • Loose or watery stools (diarrhea)

• Upset stomach (nausea)

• Rash, itching, which sometimes can be a sign of an allergic reaction

• Increased creatine phosphokinase (CPK), which could mean muscle damage

 • Depression

Occasional side effects which are serious include:

•  Abnormal liver function tests, which could mean liver damage

•  Abnormal glucose (blood sugar) levels

•   Increased triglycerides (fatty acids in the blood) seen in up to 10% of people taking the drug, and may be elevated as high at >700 mg/dl.

Occasional side effects which are not as serious, include:

•  Headache                                              •  Dizziness

•  Inability to sleep, unusual dreams

• Vomiting                                                •  Abdominal pain

• Skin darkening of the palms and/or soles

• Increased cough                                     •  Runny nose

Rare side effects which are serious, include:

• Increases in pancreatic enzyme (substances in the blood), which could mean a problem with the pancreas

NOTE:  If you are infected with both Hepatitis B and HIV, you should be aware that your liver function tests may increase, and symptoms associated with hepatitis (an acute inflammation of the liver) may worsen if emtricitabine is stopped.

Tenofovir Disoproxil Fumarate (TDF): The following side effects have been associated with the use of tenofovir:

Occasional side effects which are not as serious, include:

• upset stomach                                         • vomiting

• gas                                                          • loose or watery stools (diarrhea)

• dizziness                                                 • abdominal pain

• lack of energy                                         • shortness of breath

• rash

Rare side effects which are serious, include:

• inflammation or swelling and possible damage to the pancreas

• increase of creatinine or decrease of phosphate in blood, which could possibly mean kidney damage or kidney failure

• Kidney damage or failure – which has rarely been associated with death

• increase of liver functions tests in children

• allergic reaction, which may include fever, rash, upset stomach, vomiting, loose or watery stools, abdominal pain, achiness, shortness of breath or a general feeling of illness.

Changes in bone growth and strength were seen in study animals given tenofovir.  Bone thinning has been seen in adults and children taking tenofovir.

NOTE A:  If you are infected with both Hepatitis B and HIV, you should be aware that your liver function test values may increase, and symptoms associated with hepatitis (an acute inflammation of the liver) may worsen if tenofovir is stopped.

NOTE B: Studies in pregnant animals using much higher doses of tenofovir than used in humans have found lower infant birth weights and bone changes. Studies in pregnant women taking tenofovir have not found an increased risk of birth defects and have not shown an increased risk of bad outcomes in their babies, but there is not a lot of experience with this drug in humans.

Stavudine (d4T): The following side-effects have been found in HIV-infected individuals who take stavudine:

Frequent serious side effects include:

1. Numbness, tingling, and pain in your hands or feet. This might not go away when the drug is stopped.
2. Abnormal liver function tests (increased liver enzymes, which may show damage to the liver)
3. Abnormal pancreatic function tests

Frequent mild side effects include:

- Rash • Upset stomach and vomiting

1. Abdominal pain • Muscle aches and pain
2. Headache • Fever/chills
3. Diarrhea • Insomnia (sleeplessness)
4. Loss of appetite

Occasional serious side effects include:

- Decrease in the red blood cells that may cause fatigue, weakness, lightheadedness, dizziness, and shortness of breath
- Decrease in the number of white blood cells that help fight infection
- Low platelet count (which can lead to serious bleeding)

Rare serious side effects include:

1. Pancreatitis (inflammation of the pancreas), which may cause death. If you develop pancreatitis, you may have one or more of the following: stomach pain, nausea, and vomiting.
2. Cases of muscle weakness that may turn into paralysis and inability to breathe and may be associated with elevation of lactic acid in your blood.

When stavudine is used with other medicines with similar side effects, these side effects may be seen more often, and may be more severe, than when stavudine is used alone.

Protease Inhibitors: Protease inhibitors are a certain type of anti-HIV drug. The use of anti-HIV drug combinations (which commonly include a protease inhibitor) may be associated with an abnormal placement of body fat and wasting. Some of the body changes include:

- Increase in fat around the waist and stomach area
- Increase in fat on the back of the neck
- Thinning of the face, legs, and arms
- Breast enlargement

The following side-effects have been found in HIV-infected individuals taking protease inhibitors:

 • Increases in the amount of triglycerides (triglycerides are fatty acids in the blood) and/or cholesterol in the blood

• Development of diabetes or worsening of high blood sugar

There have been reports of increased bleeding in HIV-infected persons with hemophilia who were treated with protease inhibitors. Hemophilia is an inherited bleeding problem. It is not known if protease inhibitors were the cause of these bleeding episodes.

Lopinavir/ritonavir (Aluvia, LPV/r): The following side-effects have been found in HIV-infected individuals taking lopinavir/ritonavir:

Frequent mild side effects include:

• Abnormal bowel movements (stools), including loose or watery stools (diarrhea), upset stomach, and stomach pain

• Large increases in triglycerides (fatty acid in the blood) and cholesterol in the blood

Occasional serious side effects include:

• Pancreatitis (inflammation of the pancreas) which may cause death. If you develop pancreatitis, you may have one or more of the following: stomach pain, nausea, vomiting or abnormal pancreatic function blood tests.

• Liver problems and worsening liver disease, which may result in death. People with these conditions may have abnormal liver function tests.

Occasional mild side effects include:

• Feeling weak and tired • Headache

• Rash (seen in children)

Rare serious side effects include:

- Cardiac rhythm disturbances

Efavirenz (EFV): The following side effects have been associated with the use of efavirenz:

Frequent serious side effects include problems associated with the central nervous system or mental function. These frequent serious side effects include:

1. Trouble sleeping such as inability to sleep, abnormal dreams, and drowsiness
2. Confusion
3. Difficulty concentrating
4. Hallucinations
5. A feeling of strangeness and losing touch with reality
6. An exaggerated feeling of well-being
7. Agitation or anxiety

Frequent mild side effects that are associated with the central nervous system or mental function include:

1. Dizziness or lightheadedness
2. Abnormal dreams
3. Drowsiness

If alcohol or mind- or mood-altering drugs are used with efavirenz, it is possible that the central nervous system side effects could become worse.

Rare and serious psychiatric problems include:

1. Suicidal thoughts or attempts
2. Aggressive behavior
3. Psychosis-like symptoms, such as abnormal thinking, paranoia, and delusions
4. Severe depression

Rare mild psychiatric problems include:

- Depression

People with a history of psychiatric problems may be at greater risk for these serious psychiatric problems.

People taking efavirenz who experience central nervous system symptoms such as dizziness, impaired concentration and/or drowsiness should avoid potentially hazardous tasks such as driving an automobile or operating machinery.

Other frequent mild side effects include:

1. Rash • Upset stomach
2. Loose or watery stools (diarrhea) • Headache
3. Sleepiness
4. Increase in cholesterol

Other occasional serious side effects include:

1. Abnormal increases in pancreatic and liver enzyme levels in the blood.
2. Pancreatitis (inflammation of the pancreas), with one or more of the following: stomach pain, nausea or vomiting

• Abnormal increase in triglycerides (fatty acids in the blood)

1. Inflammation of the liver (hepatitis)

Other occasional mild side effects include:

1. Abnormal vision • Fever
2. Abnormal or unusual distribution of body fat
3. Vomiting • Fatigue

**ARE THERE RISKS RELATED TO PREGNANCY?**

It is not known for certain if the drug combinations in this study harm unborn babies, though several studies suggest that they are safe. There have been 4 babies with serious back problems who were exposed to efavirenz in the first trimester of pregnancy. These back problems include a condition called myelomeningocele when part of the spinal cord is open to the air at birth. As far as we know, there is no increased risk of birth defects among babies exposed to efavirenz after the first trimester, similar to how efavirenz would be used in this study. The ultrasound we do at the beginning of the study will help make sure that you do not receive any study medication during the first trimester of your pregnancy. The study medications are used routinely in pregnant women in the United States.

The drug or drug combinations in this study may be unsafe for unborn babies. The risks to unborn babies for each drug are listed in the section called “Risks and/or Discomforts.” If after you deliver, you are having sex that could lead to pregnancy, you must agree not to become pregnant.

As mentioned above, we require that after you deliver you:

1. obtain an intra-uterine device (IUD) or get a tubal ligation (permanent sterilization)

or

- use 2 forms of birth control, with at least one form not containing hormones.

We will provide these forms of birth control to you or refer you locally.

We recommend that you continue birth control for at least 3 months after the study ends, especially if you receive efavirenz as part of the study.

If you become pregnant during the follow-up period after you give birth, you should tell your study doctor or nurse right away. Depending on which medications you are taking, your treatment may be stopped or changed, and the study doctor will discuss your choices with you. Your choice will not affect your usual medical care provided by your doctor. You will be allowed to join future research studies, if you qualify.

Risks of Taking Combination Therapy: There is an increased risk of side effects when taking combinations of drugs, and there may be unknown risks from taking these drugs in combination. The effects that the study drugs may have on the unborn infant are unknown. A possible increase in premature births has been reported in some women receiving combination HIV therapy with and without protease inhibitor drugs. Other poor outcomes, including deaths, have been reported. The relationship of protease inhibitor therapy to the mother’s own health is unknown at this time, but these medications are used routinely in pregnant women around the world.

Risks of Breastfeeding: A mother who is infected with HIV may infect her baby through breast milk. Anti-HIV drugs can decrease the chance that HIV can pass from your breast milk to your baby. Some anti-HIV drugs you are taking may pass through your breast milk, but it is not known whether these anti-HIV drugs can cause harm to your infant. We will watch your baby closely for signs of harm.

Risks of Blood Draws: There may be some discomfort when blood is drawn. Other risks include bleeding or bruising where the needle enters the body. A small blood clot may form where the needle enters the body or swelling of the surrounding skin may occur. There is also a small risk of minor infection at the blood draw site.

Questionnaires: Answering some questions may make you feel uncomfortable. You may refuse to answer any question or stop at any time.

**ARE THERE BENEFITS TO TAKING PART IN THIS STUDY?**

1) If you and your baby take part in this study, there may be a direct benefit to both of you, but no guarantee can be made. It is also possible that you and your baby may receive no benefit from being in this study.

2) Taking the study drugs may help you feel better, slow the HIV infection, and decrease the chance that your baby will get HIV, but no guarantees can be made. By taking combination anti-HIV drugs during pregnancy and breastfeeding, you and your baby may be less likely to experience effects of malaria, but no guarantees can be made. By participating in this study, you may have access to certain anti-HIV drugs that you would not otherwise have access to.

- You will be assigned to a treatment program by chance, and the treatment you receive may prove to be more effective than the other study treatments for the prevention of malaria, but this cannot be guaranteed.

4) Knowledge gained from this study may help other pregnant women with HIV and/or malaria in the future.

What other choices do I have if I do not want to take part in this study?

Instead of being in this study, you have the choice of:

- treatment with anti-HIV prescription drugs available to you or your baby
- treatment with simpler regimens and/or fewer drugs
- no treatment

You are free to decide whether or not you want to be in this study. If you decide you do not want to be in this study or decide to stop being in the study at any time and for any reason, this will not affect your or your baby’s care at Tororo District Hospital. All study procedures and study drugs are available outside this study. If you decide not to take part, you and your baby may still get medical care and get any of the study drugs if your doctor thinks they are needed.

Please talk to your doctor about these and other choices available to you and your baby. Your doctor will explain the risks and benefits of these choices.

Will my (AND MY BABY’S) medical information be kept private?

Other people may learn that you and your baby are part of this study because you will get medical care at the study clinic; however, we will not allow people who are not working for the study to see any medical information about you or your child. Medical information about HIV, malaria and other diseases will be collected on you and your baby, but only the people working on the study will see it. Only people involved in this study will be able to link your and your child’s medical records to your and your baby’s study number. The universities and research organizations running this study are not allowed to let others know the identity of the people in the study. The medical records for the study will be kept in a locked office and will only be able to be seen by study workers. Your name or your baby’s name will not be written in any reports based on this research. People or organizations which may review your records include: the Uganda Ministry of Health, the U.S. Food and Drug Administration (FDA), UCSF institutional review board or Makerere University research ethics committee, study staff, study monitors, and Abbott Laboratories (a drug company supporting this study).

Your data will be sent to the pregnancy registry. This is a database that tracks anti-HIV drug treatment taken during pregnancy. All identifying information about you will be removed.

**WHAT ARE THE COSTS TO ME?**

There will be no cost for you or your baby to take part in this study.

Will I be paid for taking part in this study?

You will not be paid for participation in the study. We will be giving you money for transport to and from your home to the study clinic for all study visits.

**WHAT HAPPENS IF I (OR MY BABY) AM INJURED BECAUSE OF TAKING PART IN THIS STUDY?**

If you (or your baby) are injured as a result of being in this study, you will be given immediate treatment for any injuries, and will be referred for further treatment, if necessary. The cost for this treatment may be charged to you (or to the program which normally pays for your medical care). There is no program for compensation either through Makerere University or the University of California, San Francisco, or the U.S. National Institutes of Health (NIH).

What are my (and my baby’s) rights if I take part in this study?

You are free to decide whether or not you want yourself and your child to be in this study. You have the right to stop your or your baby’s participation in the study at any time without penalty or loss of benefits to which you are otherwise entitled.

You are also free to talk with relatives, friends, health care providers and other people you know before you decide if you want to be in the study or not. Depending how long you have been pregnant, you have between 1 and 4 weeks to decide if you want to be in the study.

We will tell you about new information from this or other studies that may affect your (or your baby’s) health, welfare, or willingness to stay in this study.

Who can answer my questions about the study?

Dr. Kamya and the study staff can answer questions you have about the study. You may call Dr. Kamya at Mulago Hospital (mobile: 0712-520469). We will give you free access to a telephone line. You may also contact Dr. Charles Ibingira (0772-437351) at the Makerere University Faculty of Medicine - Research and Ethics Committee which approved this study for questions about participants’ rights and research-related harm.

**CONSENT: WHAT YOUR SIGNATURE OR THUMBPRINT MEANS**

PARTICIPATION IN RESEARCH IS VOLUNTARY. You have the right to decline to participate or to withdraw from the study at any point in this study without penalty or loss of benefits to which you or your baby are otherwise entitled. A copy of this consent form will be given to you. Your signature or thumbprint below means that you have had this study explained to you. Your signature or thumbprint below means you have had the opportunity to ask questions and to get answers. If you wish to participate in this study, you should sign or place your thumbprint below. You will also be asked to sign another form if you agree to allow left-over blood specimens and hair to be used for future studies.

Name of Participant (printed)

Participant’s Signature or Thumbprint Date

Name of Study Staff Administering Consent (printed) Position/Title

Signature of Study Staff Administering Consent Date

Name of Translator (if necessary)

Signature of Translator Date

*If the participant or parent or guardian is unable to read and/or write, an impartial witness must be present during the informed consent discussion. After the written informed consent form is read and explained to the participant, parent or guardian, after oral consent to the participation in the trial, and after the participant, parent or guardian has signed the consent form or provided her or his fingerprint, the witness should sign and personally date the consent form. By signing the consent form, the witness attests that the information in the consent form and any other written information was accurately explained to, and apparently understood by, the participant and parent or guardian, and that informed consent was freely given by the participant and parent or guardian.

Name of Person Witnessing Consent (printed)

Signature of Person Witnessing Consent Date

**Appendix VIII. Future Use of Biological Specimens Informed Consent**

**Protocol Title:** Protease Inhibitors to Reduce Malaria

Morbidity in HIV-Infected Pregnant Women

**Funding Source**: NIH/NICHD (NICHD P01 HD059454)

**UCSF-CHR Number**: H5741-34342

**FOM-REC Number:** 2009-141

**UNCST Number:** HS-670

**Site of Research**: Tororo District Hospital

Tororo, Uganda

**U.S. Principal Investigator**: Diane Havlir, MD

**Ugandan Principal Investigator**: Moses Kamya, MBChB, MMed, MPH, PhD

**Version Date**: April 5, 2010

**INTRODUCTION**

While you and your child are in this study, there may be blood samples taken from you and your child that may be useful for future research. These samples will be stored for a long time at one of the universities or research organizations which are running this study. These are: Infectious Disease Research Collaboration and the University of California, San Francisco. Samples may also be shared with investigators at other institutions.

**WHAT SAMPLES WILL BE USED FOR**

Your or your child’s blood will be used to study HIV and malaria and the response of these diseases to treatment. The information we get from these studies will not affect your or your child’s care.

1. These samples will be used for future research to learn more about HIV and malaria and other diseases.
2. Your or your child’s samples will be used only for research. They will not be sold or used for the production of commercial products.
3. We may perform genetic research on these samples. However, no genetic information obtained from this research will be placed in your or your child’s medical records. These samples will be identified only by codes so that they cannot be readily identified with you or your child.

**LEVEL OF IDENTIFICATION**

We will code your and your child’s samples using numbers. Therefore, the study workers will not be able to easily find out your name or your child’s name. We will not put reports about research done with your or your child’s samples into your or your child’s medical record. We will not allow researchers using your or your child’s samples to know your name or your child’s name to the best of our ability.

Researchers who study your or your child’s samples in the future may need to know more about you or your child, such as age, gender and race. We may give this information to a researcher if it is available from your or your child’s participation in this study. We will not give your name or your child’s name for anything that might identify you or your child personally. We will not ask you to sign another consent form.

**RISKS**

There are few risks to you or your child from future use of your child’s samples. A potential risk might be the release of information from your or your child’s health or study records. We will not put reports about research done with your or your child’s samples into health records. We will keep these reports with study records. We will keep study records as private and safe as possible.

**BENEFITS**

There will be no direct benefit to you or your child from this future research on stored samples. From studying your or your child’s samples we may learn more about malaria or other diseases. We may learn how to prevent them, how to treat them, or how to cure them.

**RESEARCH RESULTS/MEDICAL RECORDS**

1. We may present results from future research using your or your child’s samples in publications and meetings. If this happens, we will not identify your or your child’s name.
2. We will not give reports from future research done with you or your child’s samples to you or your doctor. We will not put these reports in your or your child’s medical record.

**QUESTIONS**

Dr. Moses Kamya and staff are available to explain this study to you and answer your questions. If you have any other questions about the information here, you may call Dr. Kamya at (mobile: 0712-520469).

**FREEDOM TO REFUSE**

You can change your mind at any time about allowing your samples or your child’s samples to be used for future research. If you change your mind, contact Dr. Moses Kamya (mobile: 0712-520469) or the study team at Tororo District Hospital (telephone: 0454-448840). Our study team will make sure either your samples or your child’s samples or both will no longer be made available for research by destroying the samples. If you decide for us to destroy samples for future research, we still want you and your child to stay in the study. We will still allow you and your child to participate in future studies, if you want.

**WHAT YOUR SIGNATURE OR THUMBPRINT MEANS**

**A copy of this consent form will be given to you.** Your signature or thumbprint below means that use of your and your child’s specimens for future research has been explained to you. It also means that you have had the opportunity to ask questions. You have also had time to think about if you want your or your child’s samples to be used for future research.

**Please indicate if you agree to allow your samples or your child’s samples to be stored and used for future studies:**

I agree to allow **my** samples to be stored and used for future studies: 🞎 Yes 🞎 No

I agree to allow my placental samples to be stored and used for future studies: 🞎 Yes 🞎 No

I agree to allow **my child’s** samples to be stored and used for future studies: 🞎 Yes 🞎 No

Name of Participant (printed)

Participant’s Signature or Thumbprint Date

Name of Study Staff Administering Consent (printed) Position/Title

Signature of Study Staff Administering Consent Date

Name of Translator (if necessary)

Signature of Translator Date

*If the participant or parent or guardian is unable to read and/or write, an impartial witness must be present during the informed consent discussion. After the written informed consent form is read and explained to the participant, parent or guardian, after oral consent to the participation in the trial, and after the participant, parent or guardian has signed the consent form or provided her or his fingerprint, the witness should sign and personally date the consent form. By signing the consent form, the witness attests that the information in the consent form and any other written information was accurately explained to, and apparently understood by, the participant and parent or guardian, and that informed consent was freely given by the participant and parent or guardian.

Name of Person Witnessing Consent (printed)

Signature of Person Witnessing Consent Date

**Appendix IX. Uganda Ministry of Health Guidelines for Routine Care of Pregnant and Postpartum Women, and Newborns**

In addition to receiving medical care as described above in the protocol, women and infants enrolled in the study will receive standard routine prenatal and postpartum care according to Uganda Ministry of Health guidelines. These standard procedures are subject to currently availability at Tororo District Hospital.

**Routine antenatal care**. Women enrolled in the study will receive routine care as designated in the Uganda Ministry of Health Guidelines. Routine antenatal care includes screening and treating for syphilis and syndromic management of sexually transmitted infections (STIs). Pregnant women will receive iron and folic acid supplementation. At the first antenatal visit, women initiate 2 weeks of multivitamins twice a day. In addition, women receive Mebendazole (either 200mg twice a day for 3 days or 500mg as a single dose) as early as possible after the 1^st^ trimester. Each antenatal visit also includes blood pressure assessment and urine dip for proteinuria. We will also recommend HIV testing for participants’ partners. All partners found to be HIV-infected will be referred locally for care. In addition, study staff will conduct on-going safer sex counseling for discordant couples.

**Routine intrapartum/delivery care.** Routine delivery care for in-hospital births will include labor management by the midwifery staff and management of obstetrical complications as per Ministry of Health guidelines. ^227^ Immediate postpartum infant care will include polio and BCG immunization, ophthalmic tetracycline, vitamin K, and initiation of neonatal prophylactic zidovudine.

**Routine postpartum care.** All postpartum women will receive vitamin A supplementation (200,000 IU) immediately following delivery. ^227^ Depending on clinical circumstances and based on local standard of care, women may receive 2 weeks of multivitamins twice a day. Common indications for postpartum multivitamins include anemia, postpartum hemorrhage and prolonged labor. Women typically have a postpartum visit 24 hours and 1 week after delivery as per Ugandan standards of care. Women also undergo a 6 weeks postpartum visit as part of routine care. These visits include an abdominal and pelvic exam, breast exam as well as syndromic management of STIs, evaluation for opportunistic infections, and follow-up on any obstetrical complications that occurred. In addition, women receive vitamin A at this visit, if not given immediately postpartum, and are continued on iron and folic acid supplementation. Contraceptive counseling is performed at this visit as is a nutritional assessment and infant feeding and support. Screening for cervical cancer will be performed postpartum by clinical TDH staff if available at Tororo District Hospital.

**Routine infant care.** As per the updated WHO HIV and infant feeding recommendations, HIV-infected women will receive extensive counseling in support of exclusive breastfeeding for 6 months. ^128 163^ Women will be counseled to begin introducing complementary foods at 6 months of life and then continue breastfeeding until the baby reaches 1 year of age. ^163 228 229^ Women should wean slowly over the course of a month. Women with infants found to be infected with HIV will be encouraged to continue breastfeeding for as long as possible. These HIV+ infants will be referred for ART. Infant HIV prophylaxis will follow Ugandan Ministry of Health guidelines. Infants will get 6 weeks of NVP syrup (10mg/ml). Additional routine infant care will include the receipt of TS starting at 6 weeks of life until confirmation of negative HIV status, at least 6 weeks after complete weaning from breastfeeding. This 6 week visit also includes evaluation of growth and age-appropriate immunizations. Infants undergo additional immunization visits at 10 weeks, 14 weeks, 6 and 9 months of life. Infants will be referred to a local health facility for additional routine health care.

**Appendix X. DAIDS Toxicity Table (Dec 2004, clarification Aug 2009)**

**Division of AIDS Table for Grading the Severity of ADULT AND PEDIATRIC Adverse Events Version 1.0, December, 2004; clarification AUGUST 2009**

The Division of AIDS Table for Grading the Severity of Adult and Pediatric Adverse Events (“DAIDS AE Grading Table”) is a descriptive terminology which can be utilized for Adverse Event (AE) reporting. A grading (severity) scale is provided for each AE term.

This clarification of the DAIDS Table for Grading the Severity of Adult and Pediatric AE’s provides additional explanation of the DAIDS AE Grading Table and clarifies some of the parameters.

**I. Instructions and Clarifications**

**Grading Adult and Pediatric AEs**

The DAIDS AE Grading Table includes parameters for grading both Adult and Pediatric AEs. When a single set of parameters is not appropriate for grading specific types of AEs for both Adult and Pediatric populations, separate sets of parameters for Adult and/or Pediatric populations (with specified respective age ranges) are given in the Table. If there is no distinction in the Table between Adult and Pediatric values for a type of AE, then the single set of parameters listed is to be used for grading the severity of both Adult and Pediatric events of that type.

**Note:** In the classification of adverse events, the term “**severe**” is not the same as “**serious**.” Severity is an indication of the intensity of a specific event (as in mild, moderate, or severe chest pain). The term “**serious**” relates to a participant/event outcome or action criteria, usually associated with events that pose a threat to a participant’s life or functioning.

Addenda 1-3 Grading Tables for Microbicide Studies

For protocols involving topical application of products to the female genital tract, male genital area or rectum, strong consideration should be given to using Appendices I-III as the primary grading scales for these areas. The protocol would need to specifically state that one or more of the Appendices would be primary (and thus take precedence over the main Grading Table) for items that are listed in both the Appendix and the main Grading Table.

- Addendum 1 - Female Genital Grading Table for Use in Microbicide Studies - [PDF](http://rcc.tech-res.com/DAIDS RCC Forms/DAIDS_AE_Grading_Table_Ver_1_0_Nov_2007_Addendum_1.pdf)
- Addendum 2 - Male Genital Grading Table for Use in Microbicide Studies - [PDF](http://rcc.tech-res.com/DAIDS RCC Forms/DAIDS_AE_Grading_Table_Ver_1_0_Nov_2007_Addendum_2.pdf)
- Addendum 3 - Rectal Grading Table for Use in Microbicide Studies - [PDF](http://rcc.tech-res.com/DAIDS RCC Forms/DAIDS_AE_Grading_Table_Ver_1_0_Nov_2007_Addendum_3.pdf)

Grade 5

For any AE where the outcome is death, the severity of the AE is classified as Grade 5.

Estimating Severity Grade for Parameters Not Identified in the Table

In order to grade a clinical AE that is not identified in the DAIDS AE grading table, use the category “Estimating Severity Grade” located on Page 3.

Determining Severity Grade for Parameters “Between Grades”

If the severity of a clinical AE could fall under either one of two grades (e.g., the severity of an AE could be either Grade 2 or Grade 3), select the higher of the two grades for the AE. If a laboratory value that is graded as a multiple of the ULN or LLN falls between two grades, select the higher of the two grades for the AE. For example, Grade 1 is 2.5 x ULN and Grade 2 is 2.6 x ULN for a parameter. If the lab value is 2.53 x ULN (which is between the two grades), the severity of this AE would be Grade 2, the higher of the two grades.

Values Below Grade 1

Any laboratory value that is between either the LLN or ULN and Grade 1 should not be graded.

Determining Severity Grade when Local Laboratory Normal Values Overlap with Grade 1 Ranges

In these situations, the severity grading is based on the ranges in the DAIDS AE Grading Table, even when there is a reference to the local lab LLN.

*For example: Phosphate, Serum, Low, Adult and Pediatric > 14 years (Page 20) Grade 1 range is 2.50 mg/dL - < LLN.* A particular laboratory’s normal range for Phosphate is 2.1 – 3.8 mg/dL. A participant’s actual lab value is 2.5. In this case, the value of 2.5 exceeds the LLN for the local lab, but will be graded as Grade 1 per DAIDS AE Grading Table.

.

**II. Definitions of terms used in the Table:**

| Basic Self-care Functions | Adult  Activities such as bathing, dressing, toileting, transfer/movement, continence, and feeding.  Young Children  Activities that are age and culturally appropriate (e.g., feeding self with culturally appropriate eating implement). |
| --- | --- |
| LLN | Lower limit of normal |
| Medical Intervention | Use of pharmacologic or biologic agent(s) for treatment of an AE. |
| NA | Not Applicable |
| Operative Intervention | Surgical OR other invasive mechanical procedures. |
| ULN | Upper limit of normal |
| Usual Social & Functional Activities | Adult  Adaptive tasks and desirable activities, such as going to work, shopping, cooking, use of transportation, pursuing a hobby, etc.  Young Children  Activities that are age and culturally appropriate (e.g., social interactions, play activities, learning tasks, etc.). |

| PARAMETER | | | | | | GRADE 1 MILD | | | GRADE 2 MODERATE | | | | GRADE 3 SEVERE | | | GRADE 4 POTENTIALLY LIFE-THREATENING | | |
| --- | --- | --- | --- | --- | --- | --- | --- | --- | --- | --- | --- | --- | --- | --- | --- | --- | --- | --- |
| ESTIMATING SEVERITY GRADE | | | | | | | | | | | | | | | | | | |
| Clinical adverse event NOT identified elsewhere in this DAIDS AE Grading Table | | | | | | Symptoms causing no or minimal interference with usual social & functional activities | | | Symptoms causing greater than minimal interference with usual social & functional activities | | | | Symptoms causing inability to perform usual social & functional activities | | | Symptoms causing inability to perform basic self-care functions OR Medical or operative intervention indicated to prevent permanent impairment, persistent disability, or death | | |
| SYSTEMIC | | | | | | | | | | | | | | | | | | |
| Acute systemic allergic reaction | | | | | | Localized urticaria (wheals) with no medical intervention indicated | | | Localized urticaria with medical intervention indicated OR Mild angioedema with no medical intervention indicated | | | | Generalized urticaria OR Angioedema with medical intervention indicated OR Symptomatic mild bronchospasm | | | Acute anaphylaxis OR Life-threatening bronchospasm OR laryngeal edema | | |
| Chills | | | | | | Symptoms causing no or minimal interference with usual social & functional activities | | | Symptoms causing greater than minimal interference with usual social & functional activities | | | | Symptoms causing inability to perform usual social & functional activities | | | NA | | |
| Fatigue  Malaise | | | | | | Symptoms causing no or minimal interference with usual social & functional activities | | | Symptoms causing greater than minimal interference with usual social & functional activities | | | | Symptoms causing inability to perform usual social & functional activities | | | Incapacitating fatigue/ malaise symptoms causing inability to perform basic self-care functions | | |
| Fever (nonaxillary) | | | | | | 37.7 – 38.6°C | | | 38.7 – 39.3°C | | | | 39.4 – 40.5°C | | | > 40.5°C | | |
| Pain (indicate body site)  DO NOT use for pain due to injection (See Injection Site Reactions: Injection site pain)  See also Headache, Arthralgia, and Myalgia | | | | | | Pain causing no or minimal interference with usual social & functional activities | | | Pain causing greater than minimal interference with usual social & functional activities | | | | Pain causing inability to perform usual social & functional activities | | | Disabling pain causing inability to perform basic self-care functions OR Hospitalization (other than emergency room visit) indicated | | |
| Unintentional weight loss | | | | | | NA | | | 5 – 9% loss in body weight from baseline | | | | 10 – 19% loss in body weight from baseline | | | ≥ 20% loss in body weight from baseline OR Aggressive intervention indicated [e.g., tube feeding or total parenteral nutrition (TPN)] | | |
| INFECTION | | | | | | | | | | | | | | | | | | |
| Infection (any other than HIV infection) | | | | | | Localized, no systemic antimicrobial treatment indicated AND Symptoms causing no or minimal interference with usual social & functional activities | | | Systemic antimicrobial treatment indicated OR Symptoms causing greater than minimal interference with usual social & functional activities | | | | Systemic antimicrobial treatment indicated AND Symptoms causing inability to perform usual social & functional activities OR Operative intervention (other than simple incision and drainage) indicated | | | Life-threatening consequences (e.g., septic shock) | | |
| **INJECTION SITE REACTIONS** | | | | | | | | | | | | | | | | | | |
| Injection site pain (pain without touching)  Or  Tenderness (pain when area is touched) | | | | | | Pain/tenderness causing no or minimal limitation of use of limb | | | Pain/tenderness limiting use of limb OR Pain/tenderness causing greater than minimal interference with usual social & functional activities | | | | Pain/tenderness causing inability to perform usual social & functional activities | | | Pain/tenderness causing inability to perform basic self-care function OR Hospitalization (other than emergency room visit) indicated for management of pain/tenderness | | |
| Injection site reaction (localized) | | | | | | | | | | | | | | | | | | |
|  | | **Adult > 15 years** | | | | | Erythema OR Induration of 5x5 cm – 9x9 cm (or 25 cm^2^ – 81cm^2^) | | | Erythema OR Induration OR Edema  > 9 cm any diameter (or > 81 cm^2^) | Ulceration OR Secondary infection OR Phlebitis OR Sterile abscess OR Drainage | | | | | | | Necrosis (involving dermis and deeper tissue) |
|  | | **Pediatric ≤ 15 years** | | | | | Erythema OR Induration OR Edema present but ≤ 2.5 cm diameter | | | Erythema OR Induration OR Edema > 2.5 cm diameter but < 50% surface area of the extremity segment (e.g., upper arm/thigh) | Erythema OR Induration OR Edema involving  ≥ 50% surface area of the extremity segment (e.g., upper arm/thigh) OR Ulceration OR Secondary infection OR Phlebitis OR Sterile abscess OR Drainage | | | | | | | Necrosis (involving dermis and deeper tissue) |
| Pruritis associated with injection  See also Skin: Pruritis (itching - no skin lesions) | | | | | | Itching localized to injection site AND Relieved spontaneously or with < 48 hours treatment | | | Itching beyond the injection site but not generalized OR Itching localized to injection site requiring ≥ 48 hours treatment | | | | Generalized itching causing inability to perform usual social & functional activities | | | NA | | |
| SKIN – DERMATOLOGICAL | | | | | | | | | | | | | | | | | | |
| Alopecia | | | | | | Thinning detectable by study participant (or by caregiver for young children and disabled adults) | | | Thinning or patchy hair loss detectable by health care provider | | | | Complete hair loss | | | NA | | |
| Cutaneous reaction – rash | | | | | | Localized macular rash | | | Diffuse macular, maculopapular, or morbilliform rash OR Target lesions | | | | Diffuse macular, maculopapular, or morbilliform rash with vesicles or limited number of bullae OR Superficial ulcerations of mucous membrane limited to one site | | | Extensive or generalized bullous lesions OR Stevens-Johnson syndrome OR Ulceration of mucous membrane involving two or more distinct mucosal sites OR Toxic epidermal necrolysis (TEN) | | |
| Hyperpigmentation | | | | | | Slight or localized | | | Marked or generalized | | | | NA | | | NA | | |
| Hypopigmentation | | | | | | Slight or localized | | | Marked or generalized | | | | NA | | | NA | | |
| Pruritis (itching – no skin lesions)  (See also Injection Site Reactions: Pruritis associated with injection) | | | | | | Itching causing no or minimal interference with usual social & functional activities | | | Itching causing greater than minimal interference with usual social & functional activities | | | | Itching causing inability to perform usual social & functional activities | | | NA | | |
| CARDIOVASCULAR | | | | | | | | | | | | | | | | | | |
| Cardiac arrhythmia (general)  (By ECG or physical exam) | | | | | | | Asymptomatic AND No intervention indicated | | | Asymptomatic AND Non-urgent medical intervention indicated | Symptomatic, non-life-threatening AND Non-urgent medical intervention indicated | | | | | | | Life-threatening arrhythmia OR Urgent intervention indicated |
| Cardiac-ischemia/infarction | | | | | | NA | | | NA | | | | Symptomatic ischemia (stable angina) OR Testing consistent with ischemia | | | Unstable angina OR Acute myocardial infarction | | |
| Hemorrhage (significant acute blood loss) | | | | | | NA | | | Symptomatic AND No transfusion indicated | | | | Symptomatic AND Transfusion of ≤ 2 units packed RBCs (for children ≤ 10 cc/kg) indicated | | | Life-threatening hypotension OR Transfusion of > 2 units packed RBCs (for children > 10 cc/kg) indicated | | |
| Hypertension | | | | | | | | | | | | | | | | | | |
|  | | | **Adult > 17 years**  (with repeat testing at same visit) | | | | 140 – 159 mmHg systolic  OR  90 – 99 mmHg diastolic | | | 160 – 179 mmHg systolic  OR  100 – 109 mmHg diastolic | | | | **≥** 180 mmHg systolic  OR  **≥** 110 mmHg diastolic | | | | Life-threatening consequences (e.g., malignant hypertension) OR Hospitalization indicated (other than emergency room visit) |
|  | | | **Correction**: in Grade 2 to 160 - 179 from > 160-179 (systolic) and to ≥ 100 -109 from > 100-109 (diastolic) and  in Grade 3 to  **≥** 180 from > 180 (systolic) and to  **≥** 110 from > 110 (diastolic). | | | | | | | | | | | | | | | |
|  | | | **Pediatric ≤ 17 years**  (with repeat testing at same visit) | | | | NA | | | 91^st^ – 94^th^ percentile adjusted for age, height, and gender (systolic and/or diastolic) | | | | ≥ 95^th^ percentile adjusted for age, height, and gender (systolic and/or diastolic) | | | | Life-threatening consequences (e.g., malignant hypertension) OR Hospitalization indicated (other than emergency room visit) |
| Hypotension | | | | | | NA | | | Symptomatic, corrected with oral fluid replacement | | | | Symptomatic, IV fluids indicated | | | Shock requiring use of vasopressors or mechanical assistance to maintain blood pressure | | |
| Pericardial effusion | | | | | | Asymptomatic, small effusion requiring no intervention | | | Asymptomatic, moderate or larger effusion requiring no intervention | | | | Effusion with non-life threatening physiologic consequences OR Effusion with non-urgent intervention indicated | | | Life-threatening consequences (e.g., tamponade) OR Urgent intervention indicated | | |
| Prolonged PR interval | | | | | | | | | | | | | | | | | | |
|  | | | | **Adult > 16 years** | | PR interval  0.21 – 0.25 sec | | | PR interval > 0.25 sec | | | | Type II 2^nd^ degree AV block OR Ventricular pause > 3.0 sec | | | Complete AV block | | |
|  | | | | **Pediatric ≤ 16 years** | | 1^st^ degree AV block (PR > normal for age and rate) | | | Type I 2^nd^ degree AV block | | | | Type II 2^nd^ degree AV block | | | Complete AV block | | |
| Prolonged QTc | | | | | | | | | | | | | | | | | | |
|  | | **Adult > 16 years** | | | | | Asymptomatic, QTc interval 0.45 – 0.47 sec OR Increase interval < 0.03 sec above baseline | | | Asymptomatic, QTc interval 0.48 – 0.49 sec OR Increase in interval 0.03 – 0.05 sec above baseline | Asymptomatic, QTc interval ≥ 0.50 sec OR Increase in interval  ≥ 0.06 sec above baseline | | | | | | Life-threatening consequences, e.g. Torsade de pointes or other associated serious ventricular dysrhythmia | |
|  | | **Pediatric ≤ 16 years** | | | | | Asymptomatic, QTc interval 0.450 –  0.464 sec | | | Asymptomatic, QTc interval 0.465 –  0.479 sec | Asymptomatic, QTc interval ≥ 0.480 sec | | | | | | Life-threatening consequences, e.g. Torsade de pointes or other associated serious ventricular dysrhythmia | |
| Thrombosis/embolism | | | | | | NA | | | Deep vein thrombosis AND No intervention indicated (e.g., anticoagulation, lysis filter, invasive procedure) | | | | Deep vein thrombosis AND Intervention indicated (e.g., anticoagulation, lysis filter, invasive procedure) | | | Embolic event (e.g., pulmonary embolism, life-threatening thrombus) | | |
| Vasovagal episode (associated with a procedure of any kind) | | | | | | Present without loss of consciousness | | | Present with transient loss of consciousness | | | | NA | | | NA | | |
| Ventricular dysfunction (congestive heart failure) | | | | | | NA | | | Asymptomatic diagnostic finding AND intervention indicated | | | | New onset with symptoms OR Worsening symptomatic congestive heart failure | | | Life-threatening congestive heart failure | | |
| GASTROINTESTINAL | | | | | | | | | | | | | | | | | | |
| Anorexia | | | | | | Loss of appetite without decreased oral intake | | | Loss of appetite associated with decreased oral intake without significant weight loss | | | | Loss of appetite associated with significant weight loss | | | Life-threatening consequences OR Aggressive intervention indicated [e.g., tube feeding or total parenteral nutrition (TPN)] | | |
| **Comment:** Please note that, while the grading scale provided for Unintentional Weight Loss may be used as a guideline when grading anorexia, this is not a requirement and should not be used as a substitute for clinical judgment. | | | | | | | | | | | | | | | | | | |
| Ascites | | | | | | Asymptomatic | | | Symptomatic AND Intervention indicated (e.g., diuretics or therapeutic paracentesis) | | | | Symptomatic despite intervention | | | Life-threatening consequences | | |
| Cholecystitis | | | | | | NA | | | Symptomatic AND Medical intervention indicated | | | | Radiologic, endoscopic, or operative intervention indicated | | | Life-threatening consequences (e.g., sepsis or perforation) | | |
| Constipation | | | | | | NA | | | Persistent constipation requiring regular use of dietary modifications, laxatives, or enemas | | | | Obstipation with manual evacuation indicated | | | Life-threatening consequences (e.g., obstruction) | | |
| Diarrhea | | | | | | | | | | | | | | | | | | |
|  | | **Adult and Pediatric ≥ 1 year** | | | | | Transient or intermittent episodes of unformed stools OR Increase of ≤ 3 stools over baseline per 24-hour period | | | Persistent episodes of unformed to watery stools OR Increase of 4 – 6 stools over baseline per 24-hour period | Bloody diarrhea OR Increase of ≥ 7 stools per 24-hour period OR IV fluid replacement indicated | | | | | | | Life-threatening consequences (e.g., hypotensive shock) |
|  | | **Pediatric < 1 year** | | | | | Liquid stools (more unformed than usual) but usual number of stools | | | Liquid stools with increased number of stools OR Mild dehydration | Liquid stools with moderate dehydration | | | | | | | Liquid stools resulting in severe dehydration with aggressive rehydration indicated OR Hypotensive shock |
| Dysphagia-Odynophagia | | | | | | Symptomatic but able to eat usual diet | | | Symptoms causing altered dietary intake without medical intervention indicated | | | | Symptoms causing severely altered dietary intake with medical intervention indicated | | | Life-threatening reduction in oral intake | | |
| Mucositis/stomatitis  (clinical exam)  Indicate site (e.g., larynx, oral)  See Genitourinary for Vulvovaginitis  See also Dysphagia-Odynophagia and Proctitis | | | | | | Erythema of the mucosa | | | Patchy pseudomembranes or ulcerations | | | | Confluent pseudomembranes or ulcerations OR Mucosal bleeding with minor trauma | | | Tissue necrosis OR Diffuse spontaneous mucosal bleeding OR Life-threatening consequences (e.g., aspiration, choking) | | |
| Nausea | | | | | | Transient (< 24 hours) or intermittent nausea with no or minimal interference with oral intake | | | Persistent nausea resulting in decreased oral intake for 24 – 48 hours | | | | Persistent nausea resulting in minimal oral intake for > 48 hours OR Aggressive rehydration indicated (e.g., IV fluids) | | | Life-threatening consequences (e.g., hypotensive shock) | | |
| Pancreatitis | | | | | | NA | | | Symptomatic AND Hospitalization not indicated (other than emergency room visit) | | | | Symptomatic AND Hospitalization indicated (other than emergency room visit) | | | Life-threatening consequences (e.g., circulatory failure, hemorrhage, sepsis) | | |
| Proctitis (functional- symptomatic)  Also see Mucositis/stomatitis  for clinical exam | | | | | | Rectal discomfort AND No intervention indicated | | | Symptoms causing greater than minimal interference with usual social & functional activities OR Medical intervention indicated | | | | Symptoms causing inability to perform usual social & functional activities OR Operative intervention indicated | | | Life-threatening consequences (e.g., perforation) | | |
| Vomiting | | | | | | Transient or intermittent vomiting with no or minimal interference with oral intake | | | Frequent episodes of vomiting with no or mild dehydration | | | | Persistent vomiting resulting in orthostatic hypotension OR Aggressive rehydration indicated (e.g., IV fluids) | | | Life-threatening consequences (e.g., hypotensive shock) | | |
| NEUROLOGIC | | | | | | | | | | | | | | | | | | |
| Alteration in personality-behavior or in mood (e.g., agitation, anxiety, depression, mania, psychosis) | | | | | | Alteration causing no or minimal interference with usual social & functional activities | | | Alteration causing greater than minimal interference with usual social & functional activities | | | | Alteration causing inability to perform usual social & functional activities | | | Behavior potentially harmful to self or others (e.g., suicidal and homicidal ideation or attempt, acute psychosis) OR Causing inability to perform basic self-care functions | | |
| Altered Mental Status  For Dementia, see Cognitive and behavioral/attentional disturbance (including dementia and attention deficit disorder) | | | | | | Changes causing no or minimal interference with usual social & functional activities | | | Mild lethargy or somnolence causing greater than minimal interference with usual social & functional activities | | | | Confusion, memory impairment, lethargy, or somnolence causing inability to perform usual social & functional activities | | | Delirium OR obtundation, OR coma | | |
| Ataxia | | | | | | Asymptomatic ataxia detectable on exam OR Minimal ataxia causing no or minimal interference with usual social & functional activities | | | Symptomatic ataxia causing greater than minimal interference with usual social & functional activities | | | | Symptomatic ataxia causing inability to perform usual social & functional activities | | | Disabling ataxia causing inability to perform basic self-care functions | | |
| Cognitive and behavioral/attentional disturbance (including dementia and attention deficit disorder) | | | | | | Disability causing no or minimal interference with usual social & functional activities OR Specialized resources not indicated | | | Disability causing greater than minimal interference with usual social & functional activities OR Specialized resources on part-time basis indicated | | | | Disability causing inability to perform usual social & functional activities OR Specialized resources on a full-time basis indicated | | | Disability causing inability to perform basic self-care functions OR Institutionalization indicated | | |
| CNS ischemia (acute) | | | | | | NA | | | NA | | | | Transient ischemic attack | | | Cerebral vascular accident (CVA, stroke) with neurological deficit | | |
| Developmental delay – **Pediatric ≤ 16 years** | | | | | | Mild developmental delay, either motor or cognitive, as determined by comparison with a developmental screening tool appropriate for the setting | | | Moderate developmental delay, either motor or cognitive, as determined by comparison with a developmental screening tool appropriate for the setting | | | | Severe developmental delay, either motor or cognitive, as determined by comparison with a developmental screening tool appropriate for the setting | | | Developmental regression, either motor or cognitive, as determined by comparison with a developmental screening tool appropriate for the setting | | |
| Headache | | | | | | Symptoms causing no or minimal interference with usual social & functional activities | | | Symptoms causing greater than minimal interference with usual social & functional activities | | | | Symptoms causing inability to perform usual social & functional activities | | | Symptoms causing inability to perform basic self-care functions OR Hospitalization indicated (other than emergency room visit) OR Headache with significant impairment of alertness or other neurologic function | | |
| Insomnia | | | | | | NA | | | Difficulty sleeping causing greater than minimal interference with usual social & functional activities | | | | Difficulty sleeping causing inability to perform usual social & functional activities | | | Disabling insomnia causing inability to perform basic self-care functions | | |
| Neuromuscular weakness  (including myopathy & neuropathy) | | | | | | Asymptomatic with decreased strength on exam OR Minimal muscle weakness causing no or minimal interference with usual social & functional activities | | | Muscle weakness causing greater than minimal interference with usual social & functional activities | | | | Muscle weakness causing inability to perform usual social & functional activities | | | Disabling muscle weakness causing inability to perform basic self-care functions OR Respiratory muscle weakness impairing ventilation | | |
| Neurosensory alteration (including paresthesia and painful neuropathy) | | | | | | Asymptomatic with sensory alteration on exam or minimal paresthesia causing no or minimal interference with usual social & functional activities | | | Sensory alteration or paresthesia causing greater than minimal interference with usual social & functional activities | | | | Sensory alteration or paresthesia causing inability to perform usual social & functional activities | | | Disabling sensory alteration or paresthesia causing inability to perform basic self-care functions | | |
| Seizure: (new onset)  **– Adult ≥ 18 years**  See also Seizure: (known pre-existing seizure disorder) | | | | | | NA | | | 1 seizure | | | | 2 **–** 4 seizures | | | Seizures of any kind which are prolonged, repetitive (e.g., status epilepticus), or difficult to control (e.g., refractory epilepsy) | | |
| Seizure: (known pre-existing seizure disorder)  **– Adult ≥ 18 years**  For worsening of existing epilepsy the grades should be based on an increase from previous level of control to any of these levels. | | | | | | NA | | | Increased frequency of pre-existing seizures (non-repetitive) without change in seizure character OR Infrequent break-through seizures while on stable medication in a previously controlled seizure disorder | | | | Change in seizure character from baseline either in duration or quality (e.g., severity or focality) | | | Seizures of any kind which are prolonged, repetitive (e.g., status epilepticus), or difficult to control (e.g., refractory epilepsy) | | |
| Seizure  **– Pediatric < 18 years** | | | | | | Seizure, generalized onset with or without secondary generalization, lasting < 5 minutes with < 24 hours post ictal state | | | Seizure, generalized onset with or without secondary generalization, lasting 5 – 20 minutes with  < 24 hours post ictal state | | | | Seizure, generalized onset with or without secondary generalization, lasting  > 20 minutes | | | Seizure, generalized onset with or without secondary generalization, requiring intubation and sedation | | |
| Syncope (not associated with a procedure) | | | | | | NA | | | Present | | | | NA | | | NA | | |
| Vertigo | | | | | | Vertigo causing no or minimal interference with usual social & functional activities | | | Vertigo causing greater than minimal interference with usual social & functional activities | | | | Vertigo causing inability to perform usual social & functional activities | | | Disabling vertigo causing inability to perform basic self-care functions | | |
| RESPIRATORY | | | | | | | | | | | | | | | | | | |
| Bronchospasm (acute) | | | | | | FEV1 or peak flow reduced to  70 **–** 80% | | | FEV1 or peak flow  50 **–** 69% | | | | FEV1 or peak flow  25 **–** 49% | | | Cyanosis OR FEV1 or peak flow < 25% OR Intubation | | |
| Dyspnea or respiratory distress | | | | | | | | | | | | | | | | | | |
|  | | **Adult ≥ 14 years** | | | | | Dyspnea on exertion with no or minimal interference with usual social & functional activities | | | Dyspnea on exertion causing greater than minimal interference with usual social & functional activities | | | | Dyspnea at rest causing inability to perform usual social & functional activities | | | Respiratory failure with ventilatory support indicated | |
|  | | **Pediatric < 14 years** | | | | | Wheezing OR minimal increase in respiratory rate for age | | | Nasal flaring OR Intercostal retractions OR Pulse oximetry 90 – 95% | | | | Dyspnea at rest causing inability to perform usual social & functional activities OR Pulse oximetry < 90% | | | Respiratory failure with ventilatory support indicated | |
| MUSCULOSKELETAL | | | | | | | | | | | | | | | | | | |
| Arthralgia  See also Arthritis | | | | | | Joint pain causing no or minimal interference with usual social & functional activities | | | Joint pain causing greater than minimal interference with usual social & functional activities | | | | Joint pain causing inability to perform usual social & functional activities | | | Disabling joint pain causing inability to perform basic self-care functions | | |
| Arthritis  See also Arthralgia | | | | | | Stiffness or joint swelling causing no or minimal interference with usual social & functional activities | | | Stiffness or joint swelling causing greater than minimal interference with usual social & functional activities | | | | Stiffness or joint swelling causing inability to perform usual social & functional activities | | | Disabling joint stiffness or swelling causing inability to perform basic self-care functions | | |
| Bone Mineral Loss | | | | | | | | | | | | | | | | | | |
|  | **Adult ≥ 21 years** | | | | BMD t-score  -2.5 to -1.0 | | | BMD t-score < -2.5 | | | | Pathological fracture (including loss of vertebral height) | | | Pathologic fracture causing life-threatening consequences | | | |
|  | **Pediatric < 21 years** | | | | BMD z-score  -2.5 to -1.0 | | | BMD z-score < -2.5 | | | | Pathological fracture (including loss of vertebral height) | | | Pathologic fracture causing life-threatening consequences | | | |
| Myalgia (non-injection site) | | | | | | Muscle pain causing no or minimal interference with usual social & functional activities | | | Muscle pain causing greater than minimal interference with usual social & functional activities | | | | Muscle pain causing inability to perform usual social & functional activities | | | Disabling muscle pain causing inability to perform basic self-care functions | | |
| Osteonecrosis | | | | | | NA | | | Asymptomatic with radiographic findings AND No operative intervention indicated | | | | Symptomatic bone pain with radiographic findings OR Operative intervention indicated | | | Disabling bone pain with radiographic findings causing inability to perform basic self-care functions | | |
| GENITOURINARY | | | | | | | | | | | | | | | | | | |
| Cervicitis  (symptoms)  (For use in studies evaluating topical study agents)  For other cervicitis see Infection: Infection (any other than HIV infection) | | | | | | Symptoms causing no or minimal interference with usual social & functional activities | | | Symptoms causing greater than minimal interference with usual social & functional activities | | | | Symptoms causing inability to perform usual social & functional activities | | | Symptoms causing inability to perform basic self-care functions | | |
| Cervicitis  (clinical exam)  (For use in studies evaluating topical study agents)  For other cervicitis see Infection: Infection (any other than HIV infection) | | | | | | Minimal cervical abnormalities on examination (erythema, mucopurulent discharge, or friability) OR Epithelial disruption  < 25% of total surface | | | Moderate cervical abnormalities on examination (erythema, mucopurulent discharge, or friability) OR Epithelial disruption of 25 – 49% total surface | | | | Severe cervical abnormalities on examination (erythema, mucopurulent discharge, or friability) OR Epithelial disruption  50 – 75% total surface | | | Epithelial disruption  > 75% total surface | | |
| Inter-menstrual bleeding (IMB) | | | | | | Spotting observed by participant OR Minimal blood observed during clinical or colposcopic examination | | | Inter-menstrual bleeding not greater in duration or amount than usual menstrual cycle | | | | Inter-menstrual bleeding greater in duration or amount than usual menstrual cycle | | | Hemorrhage with life-threatening hypotension OR Operative intervention indicated | | |
| Urinary tract obstruction (e.g., stone) | | | | | | NA | | | Signs or symptoms of urinary tract obstruction without hydronephrosis or renal dysfunction | | | | Signs or symptoms of urinary tract obstruction with hydronephrosis or renal dysfunction | | | Obstruction causing life-threatening consequences | | |
| Vulvovaginitis  (symptoms)  (Use in studies evaluating topical study agents)  For other vulvovaginitis see Infection: Infection (any other than HIV infection) | | | | | | Symptoms causing no or minimal interference with usual social & functional activities | | | Symptoms causing greater than minimal interference with usual social & functional activities | | | | Symptoms causing inability to perform usual social & functional activities | | | Symptoms causing inability to perform basic self-care functions | | |
| Vulvovaginitis  (clinical exam)  (Use in studies evaluating topical study agents)  For other vulvovaginitis see Infection: Infection (any other than HIV infection) | | | | | | Minimal vaginal abnormalities on examination OR Epithelial disruption  < 25% of total surface | | | Moderate vaginal abnormalities on examination OR Epithelial disruption of 25 - 49% total surface | | | | Severe vaginal abnormalities on examination OR Epithelial disruption  50 - 75% total surface | | | Vaginal perforation OR Epithelial disruption > 75% total surface | | |
| OCULAR/VISUAL | | | | | | | | | | | | | | | | | | |
| Uveitis | | | | | | Asymptomatic but detectable on exam | | | Symptomatic anterior uveitis OR Medical intervention indicated | | | | Posterior or pan-uveitis OR Operative intervention indicated | | | Disabling visual loss in affected eye(s) | | |
| Visual changes (from baseline) | | | | | | Visual changes causing no or minimal interference with usual social & functional activities | | | Visual changes causing greater than minimal interference with usual social & functional activities | | | | Visual changes causing inability to perform usual social & functional activities | | | Disabling visual loss in affected eye(s) | | |
| ENDOCRINE/METABOLIC | | | | | | | | | | | | | | | | | | |
| Abnormal fat accumulation (e.g., back of neck, breasts, abdomen) | | | | | | Detectable by study participant (or by caregiver for young children and disabled adults) | | | Detectable on physical exam by health care provider | | | | Disfiguring OR Obvious changes on casual visual inspection | | | NA | | |
| Diabetes mellitus | | | | | | NA | | | New onset without need to initiate medication OR Modification of current medications to regain glucose control | | | | New onset with initiation of medication indicated OR Diabetes uncontrolled despite treatment modification | | | Life-threatening consequences (e.g., ketoacidosis, hyperosmolar non-ketotic coma) | | |
| Gynecomastia | | | | | | Detectable by study participant or caregiver (for young children and disabled adults) | | | Detectable on physical exam by health care provider | | | | Disfiguring OR Obvious on casual visual inspection | | | NA | | |
| Hyperthyroidism | | | | | | Asymptomatic | | | Symptomatic causing greater than minimal interference with usual social & functional activities OR Thyroid suppression therapy indicated | | | | Symptoms causing inability to perform usual social & functional activities OR Uncontrolled despite treatment modification | | | Life-threatening consequences (e.g., thyroid storm) | | |
| Hypothyroidism | | | | | | Asymptomatic | | | Symptomatic causing greater than minimal interference with usual social & functional activities OR Thyroid replacement therapy indicated | | | | Symptoms causing inability to perform usual social & functional activities OR Uncontrolled despite treatment modification | | | Life-threatening consequences (e.g., myxedema coma) | | |
| Lipoatrophy (e.g., fat loss from the face, extremities, buttocks) | | | | | | Detectable by study participant (or by caregiver for young children and disabled adults) | | | Detectable on physical exam by health care provider | | | | Disfiguring OR Obvious on casual visual inspection | | | NA | | |

| Laboratory | | | | | |
| --- | --- | --- | --- | --- | --- |
| PARAMETER | | GRADE 1 MILD | GRADE 2 MODERATE | GRADE 3 SEVERE | GRADE 4 POTENTIALLY LIFE-THREATENING |
| HEMATOLOGY *Standard International Units are listed in italics* | | | | | |
| Absolute CD4+ count  **–** **Adult and Pediatric  > 13 years** (HIV negative only) | | 300 – 400/mm^3^ *300 – 400/µL* | 200 – 299/mm^3^ *200 – 299/µL* | 100 – 199/mm^3^ *100 – 199/µL* | < 100/mm^3^ *< 100/µL* |
| Absolute lymphocyte count  **–** **Adult and Pediatric**  **> 13 years** (HIV negative only) | | 600 – 650/mm^3^ *0.600 x 10^9^ – 0.650 x 10^9^/L* | 500 – 599/mm^3^ *0.500 x 10^9^ –  0.599 x 10^9^/L* | 350 – 499/mm^3^ *0.350 x 10^9^ –*   *0.499 x 10^9^/L* | < 350/mm^3^ *<* *0.350 x 10^9^/L* |
| **Comment:** Values in children ≤ 13 years are not given for the two parameters above because the absolute counts are variable. | | | | | |
| Absolute neutrophil count (ANC) * | | | | | |
|  | **Adult *** | 1,000 – 1,300/mm^3^ *1.000 x 10^9^ – 1.300 x 10^9^/L* | 750 – 999/mm^3^ *0.750 x 10^9^ –*  *0.999 x 10^9^/L* | 500 – 749/mm^3^ *0.500 x 10^9^ –  0.749 x 10^9^/L* | < 500/mm^3^ < *0.500 x 10^9^/L* |
| **Comment:**  Parameter changed from “Infant, *<* 1 day” to “Infant, **≤**1 day” | | | | | |
| Fibrinogen, decreased | | 100 – 200 mg/dL *1.00 – 2.00 g/L* OR 0.75 – 0.99 x LLN | 75 – 99 mg/dL *0.75* – *0.99 g/L* OR 0.50 – 0.74 x LLN | 50 – 74 mg/dL *0.50* – *0.74 g/L* OR 0.25 – 0.49 x LLN | < 50 mg/dL  *< 0.50 g/L* OR < 0.25 x LLN OR  Associated with gross bleeding |

**See Appendix X(a) for pediatric ANC scales*

| **LABORATORY** | | | | | |
| --- | --- | --- | --- | --- | --- |
| PARAMETER | | GRADE 1 MILD | GRADE 2 MODERATE | GRADE 3 SEVERE | GRADE 4 POTENTIALLY LIFE-THREATENING |
| Hemoglobin (Hgb) | | | | | |
| **Comment:** The Hgb values in mmol/L have changed because the conversion factor used to convert g/dL to mmol/L has been changed from 0.155 to 0.6206 (the most commonly used conversion factor).  For grading Hgb results obtained by an analytic method with a conversion factor other than 0.6206, the result must be converted to g/dL using the appropriate conversion factor for that lab. | | | | | |
|  | **Adult and Pediatric ≥ 57 days**  (HIV positive only) | 8.5 – 10.0 g/dL  *5.24 – 6.23 mmol/L* | 7.5 – 8.4 g/dL  *4.62–5.23 mmol/L* | 6.50 – 7.4 g/dL  *4.03–4.61 mmol/L* | < 6.5 g/dL  *< 4.03 mmol/L* |
|  | **Adult and Pediatric  ≥ 57 days** (HIV negative only) | 10.0 – 10.9 g/dL *6.18* – 6.79 *mmol/L*  OR Any decrease  2.5 – 3.4 g/dL *1.58 – 2.13 mmol/L* | 9.0 – 9.9 g/dL *5.55 - 6.17 mmol/L*  OR Any decrease  3.5 – 4.4 g/dL *2.14 – 2.78 mmol/L* | 7.0 – 8.9 g/dL *4.34 - 5.54 mmol/L*  OR Any decrease  ≥ 4.5 g/dL *> 2.79 mmol/L* | < 7.0 g/dL *< 4.34 mmol/L* |
| **Comment:** The decrease is a decrease from baseline | | | | | |
|  | **Infant^*^**^†^**, 36 – 56 days** (HIV positive or negative) | 8.5 – 9.4 g/dL *5.24 – 5.86 mmol/L* | 7.0 – 8.4 g/dL *4.31 – 5.23 mmol/L* | 6.0 – 6.9 g/dL *3.72 – 4.30 mmol/L* | < 6.00 g/dL *< 3.72 mmol/L* |
|  | **Infant^*^**^†^**, 22 – 35 days** (HIV positive or negative) | 9.5 – 10.5 g/dL *5.87 - 6.54 mmol/L* | 8.0 – 9.4 g/dL *4.93 – 5.86 mmol/L* | 7.0 – 7.9 g/dL *4.34 – 4.92 mmol/L* | < 7.00 g/dL *< 4.34 mmol/L* |
|  | **Infant^*^**^†^**, ≤ 21 days** (HIV positive or negative) | 12.0 – 13.0 g/dL *7.42 – 8.09 mmol/L* | 10.0 – 11.9 g/dL *6.18 – 7.41 mmol/L* | 9.0 – 9.9 g/dL *5.59- 6.17 mmol/L* | < 9.0 g/dL *< 5.59 mmol/L* |
| **Correction**: Parameter changed from “Infant *<* 21 days” to “Infant ≤ 21 days” | | | | | |
| International Normalized Ratio of prothrombin time (INR) | | 1.1 – 1.5 x ULN | 1.6 – 2.0 x ULN | 2.1 – 3.0 x ULN | > 3.0 x ULN |
| Methemoglobin | | 5.0 – 10.0% | 10.1 – 15.0% | 15.1 – 20.0% | > 20.0% |
| Prothrombin Time (PT) | | 1.1 – 1.25 x ULN | 1.26 – 1.50 x ULN | 1.51 – 3.00 x ULN | > 3.00 x ULN |
| Partial Thromboplastin Time (PTT) | | 1.1 – 1.66 x ULN | 1.67 – 2.33 x ULN | 2.34 – 3.00 x ULN | > 3.00 x ULN |
| Platelets, decreased | | 100,000 –  124,999/mm^3^ *100.000 x 10^9^ – 124.999 x 10^9^/L* | 50,000 –  99,999/mm^3^ *50.000 x 10^9^ – 99.999 x 10^9^/L* | 25,000 –  49,999/mm^3^ *25.000 x 10^9^ – 49.999 x 10^9^/L* | < 25,000/mm^3^ *< 25.000 x 10^9^/L* |
| WBC, decreased | | 2,000 – 2,500/mm^3^ *2.000 x 10^9^ –  2.500 x 10^9^/L* | 1,500 – 1,999/mm^3^ *1.500 x 10^9^ –  1.999 x 10^9^/L* | 1,000 – 1,499/mm^3^ *1.000 x 10^9^ –  1.499 x 10^9^/L* | < 1,000/mm^3^ *< 1.000 x 10^9^/L* |

| LABORATORY | | | | | |
| --- | --- | --- | --- | --- | --- |
| PARAMETER | | GRADE 1 MILD | GRADE 2 MODERATE | GRADE 3 SEVERE | GRADE 4 POTENTIALLY LIFE-THREATENING |
| CHEMISTRIES *Standard International Units are listed in italics* | | | | | |
| Acidosis | | NA | pH < normal, but ≥ 7.3 | pH < 7.3 without life-threatening consequences | pH < 7.3 with life-threatening consequences |
| Albumin, serum, low | | 3.0 g/dL – < LLN *30 g/L* – *< LLN* | 2.0 – 2.9 g/dL *20* – *29 g/L* | < 2.0 g/dL *< 20 g/L* | NA |
| Alkaline Phosphatase | | 1.25 – 2.5 x ULN^†^ | 2.6 – 5.0 x ULN^†^ | 5.1 – 10.0 x ULN^†^ | > 10.0 x ULN^†^ |
| Alkalosis | | NA | pH > normal, but ≤ 7.5 | pH > 7.5 without life-threatening consequences | pH > 7.5 with life-threatening consequences |
| ALT (SGPT) | | 1.25 – 2.5 x ULN  56 – 113 U/L | 2.6 – 5.0 x ULN  114 – 225 U/L | 5.1 – 10.0 x ULN  226 – 450 U/L | > 10.0 x ULN  > 450 U/L |
| AST (SGOT) | | 1.25 – 2.5 x ULN  56 – 113 U/L | 2.6 – 5.0 x ULN  114 – 225 U/L | 5.1 – 10.0 x ULN  226 – 450 U/L | - - 10.0 x ULN   > 450 U/L |
| **Comment:** Some laboratories will report this value as Bicarbonate (HCO_3_) and others as Total Carbon Dioxide (CO_2_). These are the same tests; values should be graded according to the ranges for Bicarbonate as listed above. | | | | | |
| Bilirubin (Total) | | | | | |
|  | **Adult and Pediatric > 14 days** | 1.1 – 1.5 x ULN | 1.6 – 2.5 x ULN | 2.6 – 5.0 x ULN | > 5.0 x ULN |
|  | **Infant^*^**^†^**, ≤ 14 days** (non-hemolytic) | NA | 20.0 – 25.0 mg/dL *342 – 428 µmol/L* | 25.1 – 30.0 mg/dL *429 – 513 µmol/L* | > 30.0 mg/dL *>* *513.0 µmol/L* |
|  | **Infant^*^**^†^**, ≤ 14 days** (hemolytic) | NA | NA | 20.0 – 25.0 mg/dL *342 – 428 µmol/L* | > 25.0 mg/dL > *428 µmol/L* |
| Calcium, serum, high | | | | | |
|  | **Adult and Pediatric  ≥ 7 days** | 10.6 – 11.5 mg/dL *2.65 – 2.88 mmol/L* | 11.6 – 12.5 mg/dL *2.89* – *3.13 mmol/L* | 12.6 – 13.5 mg/dL *3.14 – 3.38 mmol/L* | > 13.5 mg/dL *> 3.38 mmol/L* |
|  | **Infant^*^**^†^**, < 7 days** | 11.5 – 12.4 mg/dL *2.88* – *3.10 mmol/L* | 12.5 – 12.9 mg/dL *3.11* – *3.23 mmol/L* | 13.0 – 13.5 mg/dL *3.245 – 3.38 mmol/L* | > 13.5 mg/dL *> 3.38 mmol/L* |
| Calcium, serum, low | | | | | |
|  | **Adult and Pediatric  ≥ 7 days** | 7.8 – 8.4 mg/dL *1.95 – 2.10 mmol/L* | 7.0 – 7.7 mg/dL *1.75* – *1.94 mmol/L* | 6.1 – 6.9 mg/dL *1.53* – *1.74 mmol/L* | < 6.1 mg/dL *< 1.53 mmol/L* |
|  | **Infant^*^**^†^**, < 7 days** | 6.5 – 7.5 mg/dL *1.63 – 1.88 mmol/L* | 6.0 – 6.4 mg/dL *1.50* – *1.62 mmol/L* | 5.50 – 5.90 mg/dL *1.38* – *1.51 mmol/L* | < 5.50 mg/dL *< 1.38 mmol/L* |
|  | **Comment:** Do not adjust Calcium, serum, low or Calcium, serum, high for albumin | | | | |

| **LABORATORY** | | | | | |
| --- | --- | --- | --- | --- | --- |
| PARAMETER | | GRADE 1 MILD | GRADE 2 MODERATE | GRADE 3 SEVERE | GRADE 4 POTENTIALLY LIFE-THREATENING |
| Cardiac troponin I (cTnI) | | NA | NA | NA | Levels consistent with myocardial infarction or unstable angina as defined by the manufacturer |
| Cardiac troponin T (cTnT) | | NA | NA | NA | ≥ 0.20 ng/mL OR Levels consistent with myocardial infarction or unstable angina as defined by the manufacturer |
| Cholesterol (fasting) | | | | | |
|  | **Adult ≥ 18 years** | 200 – 239 mg/dL *5.18 – 6.19 mmol/L* | 240 – 300 mg/dL *6.20 – 7.77 mmol/L* | > 300 mg/dL > *7.77 mmol/L* | NA |
|  | **Pediatric < 18 years** | 170 – 199 mg/dL *4.40 – 5.15 mmol/L* | 200 – 300 mg/dL *5.16 – 7.77 mmol/L* | > 300 mg/dL *> 7.77 mmol/L* | NA |
| Creatine Kinase | | 3.0 – 5.9 x ULN^†^ | 6.0 – 9.9 x ULN^†^ | 10.0 – 19.9 x ULN^†^ | ≥ 20.0 x ULN^†^ |
| Creatinine | | 1.1 – 1.3 x ULN^†^ | 1.4 – 1.8 x ULN^†^ | 1.9 – 3.4 x ULN^†^ | ≥ 3.5 x ULN^†^ |
| Glucose, serum, high | | | | | |
|  | Nonfasting | 116 – 160 mg/dL *6.44* – *8.88 mmol/L* | 161 – 250 mg/dL *8.89 – 13.88 mmol/L* | 251 – 500 mg/dL *13.89 – 27.75 mmol/L* | > 500 mg/dL *> 27.75 mmol/L* |
|  | Fasting | 110 – 125 mg/dL *6.11* – *6.94 mmol/L* | 126 – 250 mg/dL *6.95* – *13.88 mmol/L* | 251 – 500 mg/dL *13.89 – 27.75 mmol/L* | > 500 mg/dL *> 27.75 mmol/L* |
| Glucose, serum, low | | | | | |
|  | **Adult and Pediatric  ≥ 1 month** | 55 – 64 mg/dL *3.05 – 3.55 mmol/L* | 40 – 54 mg/dL *2.22 – 3.06 mmol/L* | 30 – 39 mg/dL *1.67* – *2.23 mmol/L* | < 30 mg/dL *< 1.67 mmol/L* |
|  | **Infant^*^**^†^**, < 1 month** | 50 – 54 mg/dL *2.78 – 3.00 mmol/L* | 40 – 49 mg/dL *2.22 – 2.77 mmol/L* | 30 – 39 mg/dL *1.67* – *2.21 mmol/L* | < 30 mg/dL *< 1.67 mmol/L* |
| Lactate | | ULN - < 2.0 x ULN without acidosis | ≥ 2.0 x ULN without acidosis | Increased lactate with pH < 7.3 without life-threatening consequences | Increased lactate with pH < 7.3 with life-threatening consequences |
| **Comment:** Added ULN to Grade 1 parameter | | | | | |
| LDL cholesterol (fasting) | | | | | |
|  | **Adult ≥ 18 years** | 130 – 159 mg/dL *3.37* **–** *4.12 mmol/L* | 160 – 190 mg/dL *4.13* **–** *4.90 mmol/L* | ≥ 190 mg/dL *≥ 4.91 mmol/L* | NA |
|  | **Pediatric > 2 - < 18 years** | 110 – 129 mg/dL  *2.85 – 3.34 mmol/L* | 130 – 189 mg/dL *3.35 – 4.90 mmol/L* | ≥ 190 mg/dL *≥ 4.91 mmol/L* | NA |

| **LABORATORY** | | | | | |
| --- | --- | --- | --- | --- | --- |
| PARAMETER | | GRADE 1 MILD | GRADE 2 MODERATE | GRADE 3 SEVERE | GRADE 4 POTENTIALLY LIFE-THREATENING |
| Lipase | | 1.1 – 1.5 x ULN | 1.6 – 3.0 x ULN | 3.1 – 5.0 x ULN | > 5.0 x ULN |
| Magnesium, serum, low | | 1.2 – 1.4 mEq/L *0.60 – 0.70 mmol/L* | 0.9 – 1.1 mEq/L *0.45* – *0.59 mmol/L* | 0.6 – 0.8 mEq/L *0.30* – *0.44 mmol/L* | < 0.60 mEq/L *< 0.30 mmol/L* |
| Pancreatic amylase | | 1.1 – 1.5 x ULN | 1.6 – 2.0 x ULN | 2.1 – 5.0 x ULN | > 5.0 x ULN |
| Phosphate, serum, low | | | | | |
|  | **Adult and Pediatric  > 14 years** | 2.5 mg/dL – < LLN *0.81 mmol/L* – *< LLN* | 2.0 – 2.4 mg/dL *0.65* – *0.80 mmol/L* | 1.0 – 1.9 mg/dL *0.32* – *0.64 mmol/L* | < 1.00 mg/dL *< 0.32 mmol/L* |
|  | **Pediatric 1 year – 14 years** | 3.0 – 3.5 mg/dL *0.97 – 1.13 mmol/L* | 2.5 – 2.9 mg/dL *0.81* – *0.96 mmol/L* | 1.5 – 2.4 mg/dL *0.48* – *0.80 mmol/L* | < 1.50 mg/dL *< 0.48 mmol/L* |
|  | **Pediatric < 1 year** | 3.5 – 4.5 mg/dL *1.13 – 1.45 mmol/L* | 2.5 – 3.4 mg/dL *0.81* – *1.12 mmol/L* | 1.5 – 2.4 mg/dL *0.48* – *0.80 mmol/L* | < 1.50 mg/dL *< 0.48 mmol/L* |
| Potassium, serum, high | | 5.6 – 6.0 mEq/L *5.6* – *6.0 mmol/L* | 6.1 – 6.5 mEq/L *6.1* – *6.5 mmol/L* | 6.6 – 7.0 mEq/L *6.6 – 7.0 mmol/L* | > 7.0 mEq/L *> 7.0 mmol/L* |
| Potassium, serum, low | | 3.0 – 3.4 mEq/L *3.0 – 3.4 mmol/L* | 2.5 – 2.9 mEq/L *2.5* – *2.9 mmol/L* | 2.0 – 2.4 mEq/L *2.0* – *2.4 mmol/L* | < 2.0 mEq/L *< 2.0 mmol/L* |
| Sodium, serum, high | | 146 – 150 mEq/L *146* – *150 mmol/L* | 151 – 154 mEq/L *151* – *154 mmol/L* | 155 – 159 mEq/L *155* – *159 mmol/L* | ≥ 160 mEq/L *≥ 160 mmol/L* |
| Sodium, serum, low | | 130 – 135 mEq/L *130 – 135 mmol/L* | 125 – 129 mEq/L *125* – *129 mmol/L* | 121 – 124 mEq/L *121* – *124 mmol/L* | ≤ 120 mEq/L *≤ 120 mmol/L* |
| Triglycerides (fasting) | | NA | 500 – 750 mg/dL *5.65* – *8.48 mmol/L* | 751 – 1,200 mg/dL *8.49 – 13.56 mmol/L* | > 1,200 mg/dL *> 13.56 mmol/L* |
| Uric acid | | 7.5 – 10.0 mg/dL 0.45 – 0.59 mmol/L | 10.1 – 12.0 mg/dL 0.60 – 0.71 mmol/L | 12.1 – 15.0 mg/dL 0.72 – 0.89 mmol/L | > 15.0 mg/dL > 0.89 mmol/L |
| URINALYSIS *Standard International Units are listed in italics* | | | | | |
| Hematuria (microscopic) | | 6 – 10 RBC/HPF | > 10 RBC/HPF | Gross, with or without clots OR with RBC casts | Transfusion indicated |
| Proteinuria, random collection | | 1 + (30 mg/dL) | 2 – 3 + (100-300 mg/dL) | 4 + (2000 mg/dL) | NA |
| Proteinuria, 24 hour collection | | | | | |
|  | **Adult and Pediatric  ≥ 10 years** | 200 – 999 mg/24 h *0.200 – 0.999 g/d* | 1,000 – 1,999 mg/24 h *1.000 – 1.999 g/d* | 2,000 – 3,500 mg/24 h *2.000 – 3.500 g/d* | > 3,500 mg/24 h *> 3.500 g/d* |
|  | **Pediatric > 3 mo -  < 10 years** | 201 – 499 mg/m^2^/24 h *0.201 – 0.499 g/d* | 500 – 799 mg/m^2^/24 h *0.500 – 0.799 g/d* | 800 – 1,000  mg/m^2^/24 h *0.800 – 1.000 g/d* | > 1,000 mg/ m^2^/24 h *> 1.000 g/d* |

Appendix XI. Supplemental Toxicity Table for Grading Severity of Adult and Pediatric Cutaneous/Skin Rash/Dermatitis Adverse Experiences

| GRADE 1 | GRADE 2 | GRADE 3 | GRADE 4 |
| --- | --- | --- | --- |
| Erythema, with or without pruritis | A. Diffuse erythematous macular or maculopapular cutaneous eruption or dry desquamation with or without pruritis (without the presence of any additional constitutional findings as described in Grade 3 of DAIDS Toxicity tables); OR typical target lesions without blistering, vesicles, or ulcerations in the lesions.  B. Urticaria | A. Diffuse erythematous macular or maculopapular cutaneous eruption or moist desquamation with or without pruritis together with any of the following constitutional findings considered related to study drug:  1. 5 x ULN AST, ALT or 2 x baseline if baseline > ULN.  2. fever, >39^o^C  3. blistering and/or vesiculation of cutaneous eruptions  4. any site of mucosal lesions; OR  B. angioedema; OR  C. exfoliative dermatitis defined as severe widespread erythema and dry scaling of the skin, with generalized superficial lymphadenopathy, and with other constitutional findings such as fever, weight loss, hypoproteinemia possibly related to study drug; OR  D. diffuse rash and serum sickness-like reactions defined as a clinical symptom complex manifested as fever, lymphadenopathy, edema, myalgia, and/or arthralgia; OR  E. diffuse cutaneous eruptions, usually starting on the face, trunk or back, often with prodromal symptoms plus one of the following:  1. cutaneous bullae, sometimes confluent with widespread sheet-like detachment of skin (<10% body surface area), (Nikolski's sign), (Stevens Johnson Syndrome (SJS))  2. two or more anatomically distinct sites of mucosal erosion or ulceration not due to another cause. | Diffuse cutaneous eruptions, usually starting on the face, trunk or back, often with prodromal symptoms plus cutaneous bullae with widespread sheet-like detachment of skin (>10% of body surface area), (Nikolski's sign), (SJS/Toxic Epidermal Necrolysis (TEN), overlap syndrome) |

**Appendix XII. Pharmacokinetics using Plasma and Hair Collection from Woman and Infants**

PLASMA COLLECTION PROCEDURE

A. Draw 5ml of blood into an EDTA tube for adults; 2ml for infants. EDTA tubes are typically lavender-topped and should be large enough to contain 5ml. All EDTA tubes gently inverted several times immediately after blood collection to ensure that the anticoagulant mixes evenly with the blood. Place each EDTA tube on ice.

B. Specimens can be processed locally if lab services are available; otherwise they should be delivered to the local processing lab within six hours of the blood draw and kept on ice (4°C) during transport. Steps for processing are as follows:

Specimen processing and storage

1. Centrifuge EDTA tubes at 1,200 x g for 10 minutes (can be performed at room temperature or at 4^o^C).

2. Sub-aliquot 1.2 ml of plasma into 2 ml cryotubes for a total of two aliquots in two different tubes. Screw-cap crytotubes are preferred to snap-top cryotubes.

3. Sub-aliquot remaining plasma in 1 ml or 0.5 ml aliquots, depending on the amount remaining, into cryotubes (Example: if 2 ml remain, aliquot 2 x 1 ml; if 1.5 ml remain, aliquot 1 x 1ml and 1 x 0.5 ml.) The aliquots should be labeled the same as the EDTA tubes used in collection.

4. Polyester tape should then be placed over the labels.

5. Freeze plasma at –70°C. Plasma for pharmacokinetic testing is to be stored at the local lab until shipment to Dr. Monica Gandhi’s laboratory as in the next section. Methods for the plasma determination of antiretroviral levels are well-established and described^230^

HAIR COLLECTION PROCEDURE

1. Clean the blades of a pair of scissors with an alcohol pad and allow blades to completely dry prior to use
2. Lift up the top layer of hair from the occipital region of the scalp. Isolate a small thatch of hair (~20 fibers of hair) from *underneath* this top layer of hair from the occipital region (can use a hair clip to keep the top layer of hair away).
3. Place a small label with the patient’s STUDY ID over the distal end of the hair thatch.
4. Cut the small hair sample off the patient’s head *as close to the scalp as possible*
5. Unfold the piece of aluminum foil and place the cut thatch of hair inside the piece of foil Refold the foil over to completely enclose the thatch of hair.
6. Place a STUDY ID label on the folded piece of foil
7. Place the folded piece of foil inside the plastic (e.g. Ziplock®) bag (each Ziplock bag will have a desiccant in it) and seal the bag
8. Hair samples should be kept at room temperature and in a dark place at each site prior to shipment (can be shipped without biohazard restrictions)

TIMING OF HAIR COLLECTION

Women will undergo hair sampling for pharmacokinetic evaluation at the 30-34 week visit. Women should be on study medication for at least 4 weeks prior to the hair collection visit. Women will undergo additional hair collection at 6-12 weeks postpartum. Hair will also be collected from infants at delivery and again at the 12 and 24 week visits if still breastfeeding (otherwise, at the visit following the cessation of breastfeeding)

ANALYSIS OF LPV AND EFV IN HAIR SAMPLES:

Hair samples will be shipped to the hair analysis laboratory at UCSF at the following address:

Monica Gandhi MD, MPH

405 Irving Street, 2^nd^ floor

San Francisco, CA 94122 USA

In brief, the methods established for lopinavir, ritonavir and efavirenz extraction and analysis are as follows^193^: About 2 mg of cut hair is placed into a test tube and 1 ml of methanol is added. The test tubes are incubated at 37°C overnight (>12h). Samples in methanol extraction tubes are evaporated by N_2_ gas at room temperature, then 1 ml of 50 mM ammonium acetate (pH, 8.5) and 20 µl of IS (ritonavir-d6, 10 µg/ml) is added. Three ml of methyl tert-butyl ether/ethyl acetate (1/1) is then added to the samples and each is vortexed for 1 minute x 3. Each tube is then centrifuged at 3000 rpm for 10 min. The samples are then frozen in a methanol/dry ice bath; the organic solvent is poured into fresh test tubes; and the solvent is evaporated by N_2_ gas. The samples are reconstituted with 0.2 ml of 50% methanol and 10 µl is injected into the liquid chromatography/tandem mass spectrometer (LC/MS/MS) system for analyzing drug concentrations.

The LC/MS/MS conditions are as follows. The LC/MS/MS system consists of Shimadzu LC-10 AD pumps, a Waters intelligent Sample Processor 717 Plus autosampler, a Shimadzu SPD10A UV detector and a Micromass Quattro LC Ultima triple quadrupole tandem mass spectrometer. The mass spectrometer is set to electrospray ionization in positive ion mode for lopinavir/ritonavir and in negative ion mode for efavirenz.

Good linearities of spiked drug concentrations from 0.01 to 4.0 ng/mg hair for ritonavir and 0.05 to 20 ng/mg hair for lopinavir and efavirenz versus the drug peak area ratio are obtained using our methods. The regression coefficients are more than 0.99 for all three ARVs. Using 2.0mg human cut hair, the lowest quantification limit (LOQ) is 0.01ng/mg hair for ritonavir and 0.05ng/mg hair for lopinavir and efavirenz. The accuracy of low, medium and high concentrations in the 3 drugs is 88.6-109% with coefficients of variation (CV) of less than 17%. The mean extraction recoveries of spiked drugs to hair samples at low, medium and high concentrations are 90.0% for ritonavir, 91.4% for lopinavir and 87.2% for efavirenz, respectively. The matrix ionization suppressions of these drugs are assessed by comparing them with the mass response from extracted blank hair samples spiked with drug in pure solution. The matrix ionization suppressions for these drugs are insignificant at less than 5%.

**Appendix XIII. Radio Announcement Script**

Version December 8, 2010

This is to inform all health workers of Tororo District and the General Public that there is an on-going study at Tororo District Hospital at the malaria clinic.

This study aims at studying the role of anti-AIDs medication in the prevention of malaria in pregnant women.

Pregnant mothers not less than 16 years of age living in Tororo District, who are HIV positive and not yet taking HIV medicine are eligible for the study.

This study is funded by collaboration between Makerere University and University of California, San Francisco.

## Appendix XIV. Food insecurity, and water and sanitation questions for Nutrition Interview (Version March 31, 2011)

*I’m going to ask you some questions about the circumstances in your household. By “household” we mean those of you that sleep under the same roof and take meals together at least four days a week.*

*I’m going to begin by asking you 9 questions about food security in your household over the last four weeks (30 days)[[1]](#footnote-2)*. I will be asking whether the condition ever happened, or if it rarely (once or twice), sometimes (three to ten times) or often (more than ten times). Remember, these are only questions about the last 4 weeks.

| 1. Did you worry that your household would not have enough food? | - 0. Never - 1. Rarely (1 – 2 times in the last 4 weeks) - 2. Sometimes (3 – 10 times in the last 4 weeks) - 3. Often (More than 10 times in the last 4 weeks) - 8. Don’t know |
| --- | --- |
|  |  |
| 2. Was there any time when you or any of your household members could not eat the food you wanted because you didn’t have resources? | - 0. Never - 1. Rarely (1 – 2 times in the last 4 weeks) - 2. Sometimes (3 – 10 times in the last 4 weeks) - 3. Often (More than 10 times in the last 4 weeks) - 8. Don’t know |
|  |  |
| 3. Was there any time when you or any of your household members could not eat the food you wanted because you didn’t have resources? | - 0. Never - 1. Rarely (1 – 2 times in the last 4 weeks) - 2. Sometimes (3 – 10 times in the last 4 weeks) - 3. Often (More than 10 times in the last 4 weeks) - 8. Don’t know |
|  |  |
| 4. Was there ever a time when you or a household member had to eat a limited variety of foods due to a lack of resources? | - 0. Never - 1. Rarely (1 – 2 times in the last 4 weeks) - 2. Sometimes (3 – 10 times in the last 4 weeks) - 3. Often (More than 10 times in the last 4 weeks) - 8. Don’t know |
|  |  |
| 5. Did you or a household member have to eat some foods that you didn’t want to eat because of lack of resources to obtain other foods? | - 0. Never - 1. Rarely (1 – 2 times in the last 4 weeks) - 2. Sometimes (3 – 10 times in the last 4 weeks) - 3. Often (More than 10 times in the last 4 weeks) - 8. Don’t know |
|  |  |
| 6. Did you or a household member ever compromise on the portion of food you ate because there was not enough? | - 0. Never - 1. Rarely (1 – 2 times in the last 4 weeks) - 2. Sometimes (3 – 10 times in the last 4 weeks) - 3. Often (More than 10 times in the last 4 weeks) - 8. Don’t know |
|  |  |
| 7. Was there any point where you or your household member had no food in the household because of lack of resources? | - 0. Never - 1. Rarely (1 – 2 times in the last 4 weeks) - 2. Sometimes (3 – 10 times in the last 4 weeks) - 3. Often (More than 10 times in the last 4 weeks) - 8. Don’t know |
|  |  |
| 8. Did you or a household member sleep hungry at night because of food shortages in the house? | - 0. Never - 1. Rarely (1 – 2 times in the last 4 weeks) - 2. Sometimes (3 – 10 times in the last 4 weeks) - 3. Often (More than 10 times in the last 4 weeks) - 8. Don’t know |
|  |  |
| 9. Did you or a household member go hungry in day and night because of limited food in the house? | - 0. Never - 1. Rarely (1 – 2 times in the last 4 weeks) - 2. Sometimes (3 – 10 times in the last 4 weeks) - 3. Often (More than 10 times in the last 4 weeks) - 8. Don’t know |

*Now I’m going to ask you 9 questions about water and sanitation in your household.[[2]](#footnote-3)*

Q1. What is the main source of drinking-water for members of your household?

Piped water into dwelling >>Q4

Piped water to yard/plot >>Q4

Public tap/standpipe >>Q2

Tubewell/borehole >>Q2

Protected dug well >>Q2

Unprotected dug well >>Q2

Protected spring >>Q2

Unprotected spring >>Q2

Rainwater collection >>Q2

Bottled water >>Q1A

Cart with small tank/drum >>Q2

Tanker-truck >>Q2

Surface water (river, dam, lake, pond, stream, canal, irrigation channels)>>Q2

Other (specify)>>Q2

Q1A. What is the main source of water used by your household for other purposes, such as cooking and hand washing?

Piped water into dwelling >>Q4

Piped water to yard/plot >>Q4

Public tap/standpipe

Tubewell/borehole

Protected dug well

Unprotected dug well

Protected spring

Unprotected spring

Rainwater collection

Cart with small tank/drum

Tanker-truck

Surface water (river, dam, lake,pond, stream, canal, irrigation channels)

Other (specify)

Q2. How long does it take to go there, get water, and come back?

No. of minutes >>Q3

Water on premises >>Q4

DK >>Q3

Q3. Who usually goes to this source to fetch the water for your household?

Probe: Is this person under age 15 years? What sex? Circle the code that best describes this person.

Adult woman >>Q4

Adult man >>Q4

Female child (under 15 years) >>Q4

Male child (under 15 years) >>Q4

DK >>Q4

Q4. Do you treat your water in any way to make it safer to drink?

Yes >>Q5

No >>Q6

DK >>Q6

Q5. What do you usually do to the water to make it safer to drink?

Prompt: Anything else? Record all items mentioned

Boil >>Q6

Add bleach/chlorine >>Q6

Strain it through a cloth >>Q6

Use a water filter (ceramic, sand, composite, etc.)>>Q6

Solar disinfection >>Q6

Let it stand and settle >>Q6

Other (specify) >>Q6

DK >>Q6

Q6. What kind of toilet facility do members of your household usually use?

*If “flush” or “pour flush” probe: Where does it flush to?*

Flush/pour flush to: >>Q7

piped sewer system >>Q7

septic tank >>Q7

pit latrine >>Q7

elsewhere >>Q7

unknown place/not sure/DK where>>Q7

Ventilated improved pit latrine (VIP) >>Q7

Pit latrine with slab >>Q7

Pit latrine without slab/open pit >>Q7

Composting toilet >>Q7

Bucket >>Q7

Hanging toilet/hanging latrine >>Q7

No facilities or bush or field >>Q9

Other (specify) >>Q7

Q7. Do you share this facility with other households?

Yes >>Q8

No >>Q9

Q8. How many households use this toilet facility?

How many other households share this toilet? >>Q9

Can any member of the public use this toilet? >>Q9

DK >>Q9

Q9. The last time your youngest child in the household passed stools, what was done to dispose of the stools?

Child used toilet/latrine Left in the open

Put/rinsed into toilet or latrine Other (specify)

Put/rinsed into drain or ditch DK

Thrown into garbage

Buried

**Appendix XV. Nutrition questions for focus group discussion or open-ended interviews (Version March 31, 2011)**

***Introduction***

*Thank you for taking the time to participate today. I’d like to talk with you to learn more about your nutritional experiences, what you eat, what you don’t eat, what you wish you did eat, etc. There are no right or wrong answers. You should consider yourself/ves to be like the teacher, and I’m the student. Please teach me about your food and nutrition! You should know that the things you say today are confidential, they will stay between us only. Do you have any questions for me before we begin?*

*1. Okay, let’s start with questions about the diet of non-pregnant adults*

What are your favorite things to eat?

Why do you like them so much?

How frequently do you eat them?

What are the main foods you typically eat?

Are there things that you don’t like to eat that you regularly do eat? What are they

Which foods do you spend the most money on every month?

Would you say you grow most of your food, or buy most of it, or somewhere in the middle?

How many meals per day do you eat?

In a typical household, who is responsible for making sure there is food to eat?

Are there things that aren’t food that are sometimes eaten , like earth or charcoal? What about other things like that?

*2. Questions about foods for pregnant women*

What are the best foods for pregnant women to eat? Why?

Do pregnant women here eat them with any regularity? Why or why not?

In your opinion, are there any foods that pregnant women should NOT eat? What are they? What is the reason?

*3. Questions about foods for infants and young children*

What do you think the ideal length of time for feeding only breast milk to children is? Are there any circumstances when you think breastmilk should not be given? Could prompt with e.g. any illness on the part of the child or mother, after the mother has been traveling, when baby has diarrhea?

What is the best age to continue feeding children any breast milk?

In your opinion, what are the best foods to feed children as soon as they are not drinking only breast milk? Why?

In your village, are these foods regularly given to children? If no, why not?

What are the foods that are regularly given to these very young children?

Which foods do you think should not be fed to very young children, children that are less than one year? Why not?

What do you think are the best foods to give to children once they get a little older, say 1 or 2 years old?

What are the foods that are regularly given to these slightly older children?

When do children start eating the same food as the adults?

Should children be given snacks? What kind? When?

How many meals should they eat?

How many meals do they typically eat in your village?

*4. Nutritional challenges*

(Besides what we discussed above) what are the biggest challenges with food that you face?

Do you see consequences of these problems in your health? Or in your children’s health?

How do you try to solve these problems? Could prompt with growing other food, borrowing food from neighbors, feeding smaller meals, skipping meals, etc.

*5. Questions about potential supplemental foods*

We may have potential supplemental foods on display and for sampling. These could include simsim (sesame) paste, odi (groundnut/peanut paste), mukene (whitebait), fresh milk, millet flour, micronutrient sprinkles (encapsulated micronutrients that can be mixed into porridgey type of foods), micronutrient tablets, and micronutrient syrups.

Which of these foods are good to give children between 6 and 12 months? Why?

Which are good to give children > 12 months? Why?

Which are good for pregnant women? Why?

Explain how each of the micronutrient supplements work.

Can you imagine eating any of these regularly? What would be difficult about it?

## Appendix XVI. Informed Consent to Participate in a Nutrition Interview or Focus Group

**Protocol Title: PROMOTE:** Protease Inhibitors to Reduce Malaria Morbidity in HIV-Infected Pregnant Women

**Funding Source**: NICHD P01 HD059454

**UCSF-CHR Number**: 10-02958

**FOM-REC Number:** 2009-141

**UNCST Number:** HS-670

**Site of Research**: Tororo District Hospital, Tororo, Uganda

**U.S. Principal Investigator**: Diane Havlir, MD, Moses Kamya, MBChB, MMed, MPH, PhD

**Version Date**: March 31, 2011
[truncated: 18,840 more chars]
